# Anti-Atopic Effect of *Scutellaria baicalensis* and *Raphanus sativus* on Atopic Dermatitis-like Lesions in Mice by Experimental Verification and Compound-Target Prediction

**DOI:** 10.3390/ph17030269

**Published:** 2024-02-20

**Authors:** Jeongmin Lee, Yun-Soo Seo, A Yeong Lee, Hyeon-Hwa Nam, Kon-Young Ji, Taesoo Kim, Sanghyun Lee, Jin Won Hyun, Changjong Moon, Yongho Cho, Bokyung Jung, Joong Sun Kim, Sungwook Chae

**Affiliations:** 1 BK21 FOUR Program, College of Veterinary Medicine, Chonnam National University, Gwangju 61186, Republic of Korea; 216392@jnu.ac.kr (J.L.); moonc@jnu.ac.kr (C.M.); yh01290@naver.com (Y.C.); 96bkjung@gmail.com (B.J.); 2 Center for Companion Animal New Drug Development, Jeonbuk Branch, Korea Institute of Toxicology, Jeongeup 56212, Republic of Korea; sys0109@kiom.re.kr (Y.-S.S.); namhh@kiom.re.kr (H.-H.N.); jky8387@kiom.re.kr (K.-Y.J.); 3 Herbal Medicine Resources Research Center, Korea Institute of Oriental Medicine, 111, Geonjae-ro, Naju-si 58245, Republic of Korea; lay7709@kiom.re.kr; 4 KM Convergence Research Division, Korea Institute of Oriental Medicine, 1672 Yuseongdae-ro, Yuseong-gu, Daejeon 34054, Republic of Korea; xotn91@kiom.re.kr; 5 Department of Plant Science and Technology, Chung-Ang University, Anseong 17546, Republic of Korea; slee@cau.ac.kr; 6 Jeju Research Center for Natural Medicine, Department of Biochemistry, College of Medicine, Jeju National University, Jeju 63243, Republic of Korea; jinwonh@jejunu.ac.kr

**Keywords:** *Scutellaria baicalensis*, *Raphanus sativus*, atopic dermatitis, T lymphocyte differentiation, skin, network pharmacology

## Abstract

*Scutellaria baicalensis* Georgi and *Raphanus Sativus* Linne herbal mixture (SRE) is a Chinese herbal medicine. In this study, we aimed to evaluate the therapeutic efficacy of SRE as an active ingredient for 2,4-dinitrochlorobenzene (DNCB)-induced atopic dermatitis (AD) and to predict the underlying therapeutic mechanisms and involved pathways using network pharmacological analysis. Treatment with SRE accelerated the development of AD-like lesions, improving thickness and edema of the epidermis. Moreover, administering the SRE to AD-like mice suppressed immunoglobulin E and interleukin-4 cytokine and reduced T lymphocyte differentiation. In silico, network analysis was used to predict the exact genes, proteins, and pathways responsible for the therapeutic effect of the SRE against DNCB-induced AD. These results indicated that the SRE exerted protective effects on the DNCB-induced AD-like model by attenuating histopathological changes and suppressing the levels of inflammatory mediators. Therefore, the SRE can potentially be a new remedy for improving AD and other inflammatory diseases and predicting the intracellular signaling pathways and target genes involved. This therapeutic effect of the SRE on AD can be used to treat DNCB-induced AD and its associated symptoms.

## 1. Introduction

Atopic dermatitis (AD) is the most common chronic inflammatory skin disease with severe symptoms such as pruritus, epidermal hyperplasia, edema, erythema and erythematous plaque, and erythematous plaque [1,2]. Some factors, such as genetic, immune function imbalance, and environmental factors, are known to cause AD. However, the precise mechanism of AD has not yet been demonstrated [3,4]. Previous reports suggested that AD affects approximately 10–20% of the total population. Some therapeutic drugs exist to treat its occurrence. Recently, anti-inflammatory drugs such as topical corticosteroids, PDE-4 inhibitors, topical calcineurin inhibitors, biologics, JAK-STAT inhibitors, and antihistamines are the mainstay of AD treatment. The primary role of these therapeutics is to preserve the skin barrier function. However, due to the limitations such as high relapse of AD and side-effects of long-term corticosteroids of current drugs, new treatments still need to be developed [5].

However, despite its common occurrence, no precise therapeutic treatment exists. Immunologically, the activation of T lymphocytes is a major mediator in the allergic inflammatory response. Moreover, an abnormal cytokine system reduces cell-mediated immunity and induces immunoglobulin E, which is essential in AD pathology [6].

Diverse inflammatory cytokines such as interleukin-4 (IL-4), IL-5, and IL-13 are regulated to atopic skin. Epidemiology studies showed that more inflammatory cytokines were produced in AD patients than in non-AD individuals. Moreover, various studies indicated that IL-4 and interferon-γ are increased in acute AD [7,8]. Ointments and oral medicines are currently used to treat atopic dermatitis to reduce inflammation and itching [9]. Drugs, such as steroids, antihistamines, immunosuppressants, and calcineurin inhibitors, are also commonly used [9,10,11]. However, side effects, including extreme skin atrophy, adrenal suppression, and susceptibility to infection, occur with the long-term use of these drugs [10,11]. Therefore, research is focused on discovering treatments for AD with reduced side effects. Consequently, our research focuses on developing new natural compounds with reduced side effects.

Although traditional medicines have been used for a long time, their pharmacological activity has not been clearly identified because of their multiple targets and compounds. However, many studies have been performed in many countries and have confirmed the efficacy of traditional medicines. As technological advancements have progressed, bioinformatical methods, such as network pharmacology, have been widely used to create “ambiguous” mechanisms of conventional medicine more explicitly [12]. Network pharmacology uses extensive databases to systematically determine the effects and mechanisms of traditional medicine prescriptions in treating complex diseases [13]. This method enables broad predictions of potential mechanisms of action, corresponding component targets and disease targets for treating disease [14].

*Scutellaria baicalensis* Georgi is a perennial herbaceous plant that includes the Lamiaceae family. It re-seeds in China and Northeast Asia, including Siberia and north of the Yangtze River [15]. The pharmacological effects of SBG protection from UV damage in aging skin provide anti-allergic [16] and anti-inflammatory effects in AD [17]. Moreover, *Raphanus sativus* Linne is a Chinese medicine made from the seeds of cruciferous or congenital plants that have been re-seeded in the Mediterranean region. RS is known for its effects on the lungs, spleen, and gastric meridians. Its pharmacological effects include inhibiting Staphylococcus aureus and skin fungus, lowering blood pressure, anti-inflammatory, antioxidant, and antidiabetic effects, and inhibiting vascular smooth muscle proliferation [18,19].

A previous study showed that *Scutellaria baicalensis* and *Raphanus sativus* mixtures may improve ultraviolet B-induced skin damage and wrinkles in mice models [20]. As mentioned above, the effects of SBG and RS have been reported, yet there is no experimental report on AD following the administration of their compound drugs. Therefore, we aimed to improve the skin inflammation response in AD by using a complex extract containing two medicinal materials to provide a synergistic effect.

## 2. Results

### 2.1. Protective Effects of SRE on DNCB-Induced Atopic Dermatitis on Mice Skin

**Figure 1:**
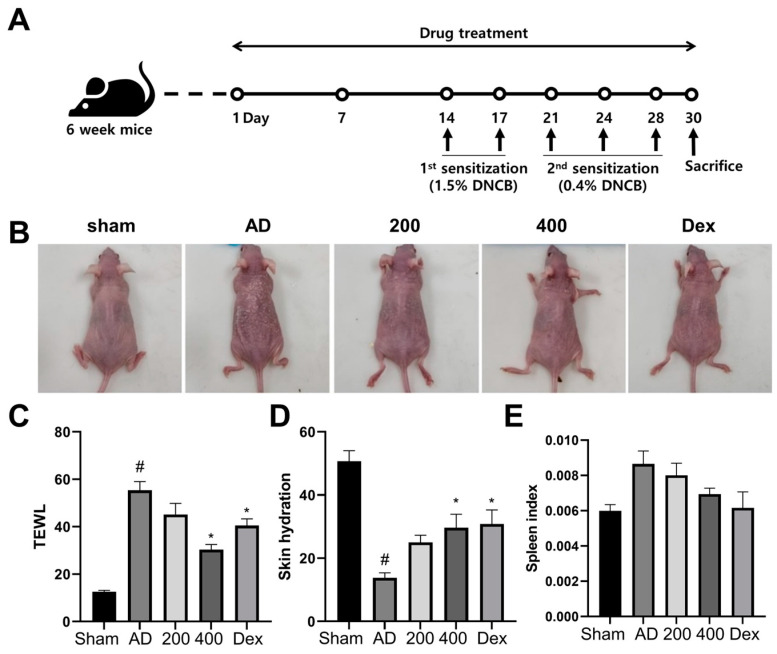
Effects of SRE on atopic dermatitis-like symptoms in hairless mice. (**A**) Schematics of experiment design. (**B**) After 30 days, images of skin lesions from the groups were taken on the last day of treatment. (**C**) Value of transepidermal water loss (TEWL). (**D**) Level of skin hydration. (**E**) Spleen weight in hairless mice (*n* = 7). All data are presented as mean ± SEs; * *p* < 0.05 compared with the AD group, and # *p* < 0.05 compared with the sham group—AD: atopic dermatitis; Dex: dexamethasone; DNCB: 2,4-dinitrochlorobenzene.

To study the therapeutic effects of SRE, AD-like skin lesions were induced by DNCB treatment in SKH-1 hairless mice, as indicated in Figure 1A. After the DNCB treatment was applied, the AD-like model group showed severe dermatitis accompanied by erythema, excoriation, scarring, and erosion. However, the AD-like mice in the SRE- and Dex-treated groups exhibited suppressed dermatitis phenotypes (Figure 1B). Moreover, as in previous studies, increased TEWL values and decreased skin hydration levels were observed in the AD-like model [21,22,23]. In this study, the SRE-treated group significantly improved the TEWL values and skin hydration levels in the SRE high-dose treated group, and the Dex group, the positive control, also significantly enhanced the TEWL values and skin hydration levels in SKH-1 hairless mice (*p* < 0.05) (Figure 1C,D). For neutralization, spleen size was calculated to reduce alongside body weight. The AD-like model tended to have increased spleen weights, while the mice treated with SRE and Dex had decreased spleen weights. However, these changes were not significant (Figure 1E). These results implicated that SRE treatment suppressed the phenotype of AD-like skin lesions, decreased the TEWL level, and increased skin hydration, like Dex—the positive control.

### 2.2. Protective Effects of SRE on DNCB-Induced Histological Changes of Atopic Dermatitis Mice Skin

**Figure 2:**
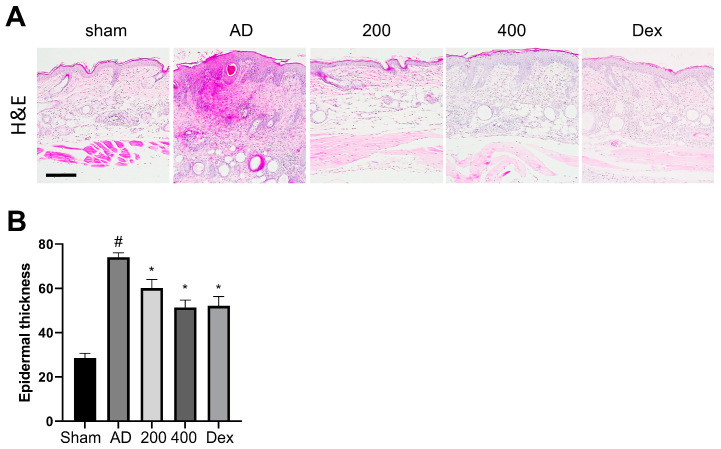
Effects of SRE on atopic dermatitis-like histological changes in hairless mice. (**A**) H&E staining of the skin lesion; scale bar = 200 μm (**B**) Epidermal thickness was analyzed in H&E-stained sections (n = 3). All data are presented as mean ± SEs; * *p* < 0.05 compared with the AD group, and # *p* < 0.05 compared with the sham group. AD: atopic dermatitis; Dex: dexamethasone.

H&E was performed to reveal the epidermal hyperplasia and inflammatory cell infiltration into the epidermal layer from the dermal skin layer in the SRE-treated AD-like model mice (Figure 2A). A previous study showed that the epidermal thickness of AD-like mice was significantly increased compared to the sham group [21,22,23]. However, SRE treatment significantly suppressed epidermal thickness in the AD-like models in an SRE dose-dependent manner (*p* < 0.05) (Figure 2B). These findings revealed that SRE treatment suppressed epidermal thickness and mast cell infiltration in AD-like skin lesions. Overall, SRE treatment showed potential protective effects against skin dysfunction and abnormal immune responses in AD skin.

### 2.3. SRE Treatment Decreased the Production of Lymphocyte Cells, IgE, and IL-4 in Mice with DNCB-Induced Atopic Dermatitis

**Figure 3:**
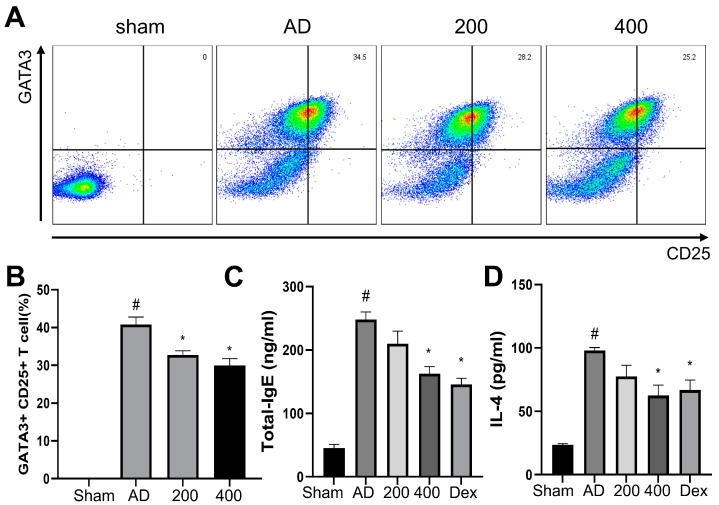
Effects of SRE on lymphocyte cells—IgE and IL-4 levels in atopic dermatitis-like hairless mice. (**A**) Dot plot of representative fluorescence-activated cell sorting analysis in the spleen. (**B**) The percentage of GATA3+ and CD25+. (**C**) Level of IgE in serum. (**D**) Level of IL-4 in hairless mice serum (n = 7). All data are presented as mean ± SEs; * *p* < 0.05 compared with the AD group, and # *p* < 0.05 compared with the sham group. AD: atopic dermatitis; IgE: immunoglobulin E; IL-4: interleukin-4; CD: cluster of differentiation; GATA: GATA binding protein 3; Dex: dexamethasone.

Flow cytometry was performed in the spleens of DNCB-induced AD mice to investigate the production of T cells, using both GATA3 and CD25 as markers. GATA3+ was expressed in Th2 cells and induced the proinflammatory cytokine IL-4 [24]. CD25+ was expressed in Treg cells [25]. As shown in Figure 3A,B, the population of GATA3+ and CD25+ in cells was significantly increased in the AD-like models. However, the expression of GATA3+ and CD25+ was significantly reduced in cells from the SRE-treated group. Moreover, the IgE and IL-4 levels in the mice serum were evaluated by ELISA. As shown in a previous study, IgE and IL-4 were significantly increased in the AD-like model group compared to the sham group [26]. In contrast, the SRE treatment significantly decreased the level of IgE and IL-4 compared to the AD-like model group (Figure 3C,D). These results showed the SRE treatment reduced lymphocyte cells, including Th2 and Treg cells in the spleen, and suppressed immunoglobulin-like IgE and proinflammatory cytokine (IL-4) levels in the serum.

### 2.4. Active Small Molecules and SRE Target Genes

A total of 185 active small molecules were searched for in TCMSP, of which 143 and 52 small molecules of *Scutellaria baicalensis* and *Raphanus sativus* were found, respectively (Supplementary Table S1). After ADME screening with OB ≥ 20% and DL ≥ 0.1, according to TCMSP, 66 active small molecules were selected; among them, 16 small molecules were related to 444 genes (Supplementary Table S2). The family of 16 small molecules were flavonoids (acacetin, apigenin, baicalein, baicalin, chrysin, oroxylin A, panicolin, and wogonin), alkaloid (coptisine), unsaturated fatty acids (erucic acid, linoleic acid, and linolenic acid), steroids (sitogluside, sitosterol, and stigmasterol), and triterpenoid (supraene). Eight genes (CAPS3, CYP1A1, CYP1A2, CYP1B1, CYP3A4, MAPK1, MAPK3, and PTGS2) among the 444 were linked to the five or more active small molecules (Supplementary Table S2).

### 2.5. Potential Target Genes and PPI

**Figure 4:**
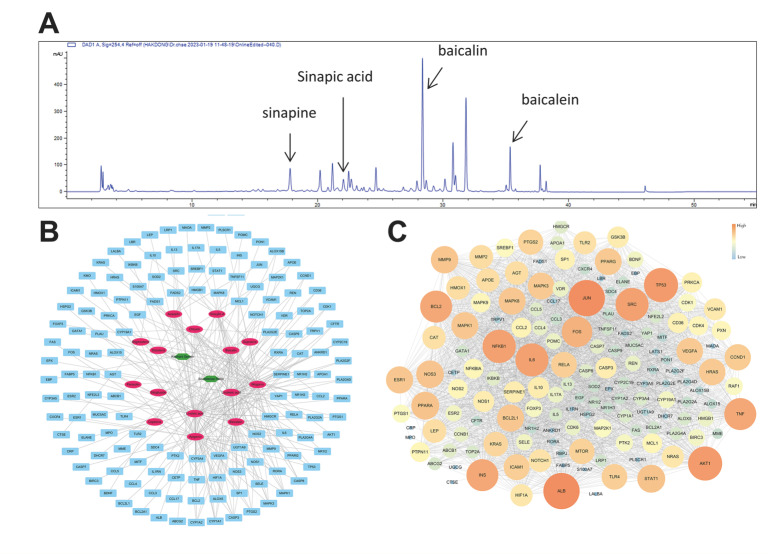
Target and potential genes of SRE. (**A**) HPLC-DAD chromatogram of the sample (254 nm) (**B**) Network analysis of herbs–small molecules–genes; the green hexagon is an herb, the pink oval is the small molecule, and the cyan rectangle is a gene. (**C**) Protein–protein interactions (PPIs): the core gene is the larger size of the circle and the more orange color in SRE.

A total of 1567 human genes related to AD (Supplementary Table S3) were searched in the GeneCards database, and 141 genes overlapped with the target gene searched above. Thus, fifteen active small molecules were deemed related to this disease and investigated. Among 141 genes, 7 were associated with five or more active small molecules: CASP3, CYP1A1, CYP1A2, CYP3A4, MAPK1, MAPK3, and PTGS2 (Figure 4B).

The protein–protein interaction (PPI) analysis utilized the STITCH database, which is very frequently used [27]. Cytoscape 3.7.2 visualized the PPI network and calculated its topological characteristics using the “network analyzer” function of this software [28,29]. High degree, closeness centrality, and betweenness centrality genes were found to be ALB, JUN, TP53, AKT1, NFKB1, IL6, SRC, INS, TNF, and BCL2 (Figure 4C).

### 2.6. Pathway Analysis Related to Atopic Dermatitis

The signaling pathways and functions of genes were analyzed using the DAVID and KEGG database with the *p*-value (*p* < 0.05) correction algorithm [30]. A total of 19 pathways were related to AD disease: TNF signaling pathway, apoptosis, IL-17 signaling pathway, VEGF signaling pathway, Toll-like receptor signaling pathway, HIF-1 signaling pathway, T cell receptor signaling pathway, PI3K–Akt signaling pathway, Ras signaling pathway, inflammatory bowel disease, chemokine signaling pathway, MAPK signaling pathway, NF-κB signaling pathway, Th17 signaling pathway, FoxO signaling pathway, NOD-like receptor signaling pathway, Th1 and Th2 cell differentiation, apoptosis-multi species, and inflammatory mediator regulation of TRP channels.

**Figure 5:**
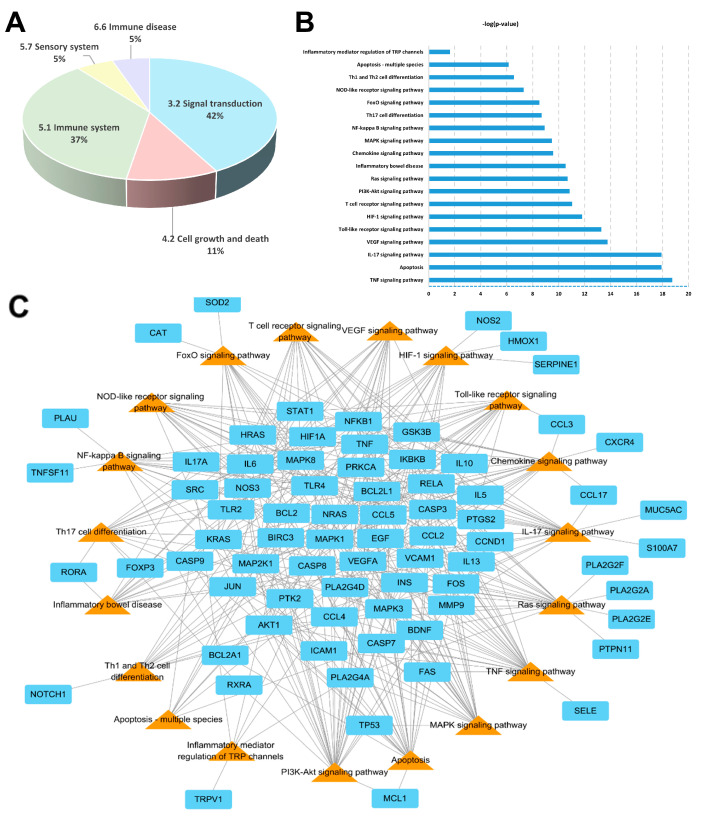
Pathway analysis of SRE: (**A**) KEGG classification of pathways. (**B**) Rank according to the *p*-value. (**C**) Pathways (triangle orange color) and their corresponding genes (rectangle cyan color) for SRE.

Among these 19 pathways, the KEGG categories accounting for 79% were signal transduction (42%) and immune system (37%) (Figure 5A). Based on the *p*-value, the top five pathways were the TNF signaling pathway, apoptosis, IL-17 signaling pathway, VEG signaling pathway, and Toll-like receptor signaling pathway (Figure 5B). A total of 75 potential target genes were involved in the aforementioned 19 pathways. Among these 75 genes, there were a total of 9 associated with more than 10 pathways: AKT1, IKBKB, JUN, MAP2K1, MAPK1, MAPK3, MAPK8, NFKB1, and RELA (Figure 5C). In this experiment, the highest related pathway to AD was Th1 and Th2 cell differentiation, which involved 12 genes.

## 3. Discussion

In this study, we investigated the protective effect of SRE by regulating lymphocytes through DNCB-induced AD-like skin lesions in SKH-1 hairless mice. The DNCB-induced model is commonly used to mimic human AD [31]. Following the previous studies, repeated exposure to DNCB causes chronic inflammation accompanied by the infiltration of lymphocytes and mast cells, such as human AD symptoms [32,33]. Our results showed that SRE treatment improved DNCB-induced AD-like skin lesions in SKH-1 hairless mice.

Treatment with SRE, an herbal mixture of SRG and RS, suppressed the clinical symptoms, phenotypes, and histopathological changes associated with the AD-like model, such as erythema, edema, erosion, skin hydration, epidermal thickness, and infiltration of mast cell in DNCB-treated SKH-1 hairless mice (Figure 1, Figure 2 and Figure 3). However, our study showed that the spleen size did not significantly change, unlike in previous reports [34]. This result in the spleen size is expected to be due to the short induction period of the AD-like skin lesions with DNCB treatment. Nevertheless, the tendency for the spleen size to change appeared to increase in the AD-like group, whereas SRE treatment decreased the spleen size in the DNCB-treated AD-like model (Figure 1E). In addition, we also observed that SRE treatment regulated the Th1/Th2 immune response and remarkably decreased serum Ig E and IL-4 levels in SKH-1 hairless mice (Figure 3). GATA3 is known to be precisely expressed in Th2 cells and differentiate into Th2 cells. This expression of GATA3 in Th2 cells mediates cytokines, such as IL-4, IL-5, and IL-13, which cause allergic inflammation, such as AD [35]. Moreover, the expression of CD25+ was established in Treg cells, suppressing the proliferative function of autologous effector T cells (Teffs) in AD. Following a previous report, the attenuated ability of Teffs to induce proliferation exacerbated AD in humans [36]. Thus, our study observed the suppressed differentiation of Th2 and Threg cells by SRE treatment, which aggravated AD phenotypes in the DNCB-induced AD-like SKH-1 skin hairless model. These results indicated a potential therapeutic effect of SRE to alleviate human AD through its ability to balance between Th1/Th2 cells.

To identify the mechanisms of SRE, we performed HPLC (Figure 4). The 15 components were classified into eight flavonoids (acacetin, apigenin, baicalein, baicalin, chrysin, wogonin, oroxylin A, and panicolin), two fatty acids (linoleic acid and linolenic acid), three steroids (sitosterol, stigmasterol, and sitoglucoside), one terpenoid (supraene), and one alkaloid (coptisine). Subsequently, as a result of the network analysis, seven small molecules, which were linoleic acid, apigenin, baicalein, linolenic acid, wogonin, baicalin, and chrysin, had high values for both degree, closeness centrality, and betweenness centrality. Baicalin and baicalein were active small molecules of *Scutellaria baicalensis* with a flavonoid structure. In previous reports, baicalin improved skin lesions by controlling the Th1/Th2 balance, improved skin barrier function, regulated intestinal bacterial imbalance, and suppressed inflammation by inhibiting the activation of the NF-κB and JAK/STAT pathways [37]. Baicalein could modulate the balance between Th1 and Th2 cells by inhibiting the production of IL-4, IL-6, and TNF-α and inducing the expression of IFN-γ [38]. *Raphanus Sativus* contains sinapic acid, a small molecular compound. Sinapic acid upregulates immunosuppression in RAW264.7 and oxazolone-induced AD-like model [39,40]. In addition, sinapic acid is known to modulate the Th1/Th2 cell differentiation [41] and alleviate the symptoms by suppressing the Th2 cell in allergic asthma. The SRE effect in AD could be improved by these immunosuppressive effects of sinapic acid [42].

Wogonin induces HO1 expression, and HO1 and/or CO suppress TARC expression in human HaCaT cells induced by tick antigens [43]. Chrysin plays a role in reducing mast cell infiltration and serum histamine levels and suppresses AD by inhibiting the inflammatory response of Th1, Th2, and Th17 cells in the ear [44]. Apigenin significantly reduced inflammatory and allergic response factors in RAW264.7 and RBL cells, thereby alleviating skin disease [45]. Polyenoic acids, such as linoleic and linolenic acid, are components that are mainly contained in plant seeds [46]. Linoleic acid inhibited IL-6, IL-1β, TNF-α, and iNOS in a dose-dependent manner, showing a potentially protective effect against AD-like lesioned skin caused by an inflammatory response [47]. An appropriate ratio of linoleic acid and linolenic acid significantly suppressed T cell proliferation and invasion and the production of Th1, Th2, and Th17 cytokines in mice skin and serum [48]. In mouse models, sitosterol and stigmasterol decreased inflammatory cell infiltration and ear edema [49]. Moreover, sitosterol inhibited NF-κB and suppressed the expression of CAM-1 and ICAM-1 stimulated by TNF-α [50]. The suparen, squalene, is a highly unsaturated hydrocarbon from the triterpenoid family and is used as an antioxidant and moisturizer for seborrheic dermatitis and AD [51].

KEGG pathway analysis associated Th1 and Th2 cell differentiation with 19 pathways. Among them, the TNF signaling pathway, apoptosis, IL-17 signaling pathway, VEG signaling pathway, and Toll-like receptor signaling pathway were closely correlated with the pathogenesis of atopic skin dermatitis [52,53,54,55].

In the network analysis, there were ten core genes, which were ALB, JUN, TP53, AKT1, NFKB1, IL6, SRC, INS, TNF, and BCL2, which are genes with a high degree of closeness centrality and betweenness centrality genes. The genes important to AD disease are Th1 and Th2 cell differentiation [56,57,58,59,60].

As a result of pathway analysis, the most critical pathway was Th1/Th2 cell differentiation, which was consistent with the efficacy of key small molecules. Additionally, the genes involved in this pathway were FOS, JUN, RELA, IKBKB, IL13, IL5, MAPK1, MAPK3, MAPK8, NOTCH1, NFKB1, and STAT1.

This study confirms the protective effects of SRE in a DNCB-induced AD mouse model by attenuating skin damage. In addition, using a network pharmacological approach to predict the protective effects of SRE, we investigated the anti-inflammatory effects of SRE on various factors that cause AD. The results suggest that SRE has potential as a treatment for AD. Moreover, it will contribute to the validation of herbal medicine for treating inflammation and skin dermatitis. However, experimental verification is still needed, as informatics is primarily theoretical. Therefore, the mechanism underlying the effects of SRE on skin damage and the signaling pathways regulated by its active constituents should be elucidated in future studies.

## 4. Materials and Methods

### 4.1. Preparation of the Extract

*Scutellaria baicalensis* and *Raphanus sativus* extracts were prepared in a previous study [20]. Dried medicinal materials *Scutellaria baicalensis* and *Raphanus sativus* were purchased from Kwangmyung-Dang (Ulsan, Republic of Korea) and used as research samples, and all voucher specimens (2015SC and 2015 SR) were stored in the herb bank of the Korea Institute of Oriental Medicine. The dried herbs of *Scutellaria baicalensis* and *Raphanus sativus* were combined, extracted using a process known as water under reflux, and filtered to provide a sample ready for examination. An herbal product in powder was obtained by evaporating the filtrate, and this product was utilized for experimental verification.

### 4.2. Preparation of the Animals

Five-week-old female SKH-1 hairless mice were used in this experiment after a week of animal laboratory adaptation, purchased from Dooyeol-Biotech, Inc. (Seoul, Republic of Korea). Experimental animals could freely consume sterile distilled water and solid feed. A total of 4–5 animals were housed in a cage with an environment of 150–300 Lux at 22 ± 2 °C, 55 ± 15% humidity, for 12 h light–dark cycles. All experimental protocols pertained to Chonnam University’s Institutional Animal Care and Use Committee (CNU IACUC-YB-2022-50).

### 4.3. Treatment of Mice

For induced AD, the back of each mouse was stimulated by 200 μL of a 1.5% 1-chloro-2,4-dini-trobenzene (DNCB, acetone:olive oil = 3:1, Sigma-Aldrich, St. Louis, MO, USA) solution two times and 200 μL of 0.4% DNCB solution, which were both applied evenly to the same area every two days until the end of the experiment, to ensure that AD persisted. To evaluate the protective effect of SBG with the RS herbal mixture (SRE) in AD, SRE was orally administered every day for 1 month. Pretreatment was performed two weeks prior to AD induction, and SRE was administered for two weeks during AD induction. A total of 5 mg/mL of dexamethasone (Dex, Sigma-Aldrich) was administered as the positive control. Figure 1A shows the schematics of the experimental design. There were six groups, with seven mice in each group: (1) PBS stimulated + PBS treatment group, (2) PBS stimulated + SRE treatment group (400 mg/kg), (3) DNCB stimulated + PBS treatment group, (4) DNCB stimulated + Dex 5 mg/mL, (5) DNCB stimulated + SRE treatment group (200 mg/kg), and (6) DNCB stimulated + SRE treatment group (400 mg/kg).

### 4.4. Transepidermal Water Loss Assessment

After the sample treatment, the amount of transepidermal water loss (TEWL) from the epidermis, which changes in AD, was measured using a measuring instrument on the mouse back. The measurement was conducted at a constant room temperature of 24–25 °C and humidity of 50–60%.

### 4.5. Histological Analysis

The skin of the sacrificed mice was fixed in a 10% neutral buffered formalin solution (NBF). After fixing, the skin tissue in each experimental group was added to 10% NBF. Dehydration processing was performed, and paraffin blocks were made for sectioning. All tissue slides were sectioned into 3 μm thicknesses. Hematoxylin and eosin (H&E) staining was performed for the histopathological analysis.

### 4.6. Flow Cytometry

After the sacrifice, the spleen was dissected, and the cells were collected. Spleen cells were stained using fluorochrome-conjugated antibodies to analyze the T helper 2 (Th2) and T regulatory (Treg) cell activation and populations. For T cell analysis, anti-GATA3 (PE, eBioscience, San Diego, CA, USA, 12-9966-42) and anti-CD25 (APC-Cy7, BioLegend, San Diego, CA, USA, 102026) antibodies were used to stain the cells. A cytoFLEX Flow Cytometer (BeckmanCoulter, Brea, CA, USA) and FlowJo version 10.6 (TreeStar, Ashland, OR, USA) were used to evaluate T cell activation. All flow cytometry procedures were performed as previously described [61].

### 4.7. Enzyme-Linked Immunosorbent Assay (ELISA)

IgE and IL-4 levels were measured using an enzyme-linked immunosorbent assay (ELISA) kit (mouse IgE Quantikine ELISA Kit and mouse IL-4 Quantikine ELISA Kit; R&D Systems, Minneapolis, MN, USA). All procedures were performed accurately, according to the manufacturer’s instructions.

### 4.8. HPLC

A total of 100.00 mg of extract was dissolved in methanol to achieve a concentration of 50 mg/mL. Subsequently, the solution was filtered through a 0.2 μm PVDF membrane filter before the HPLC analysis. All standard compounds were dissolved in methanol. The HPLC system utilized for this analysis comprised the Agilent 1260 Infinity II Quat Pump from CA, USA, integrated with a DAD WR detector. The flow rate and injection volume were 10 mL/min and 1.0 μL, respectively. The monitored wavelength range for PDA detection was 200 to 420 nm, with 254 nm being the detection wavelength of the specifically targeted peaks. The mobile phase comprised 0.1% aqueous TFA (A) and acetonitrile (B). The specific conditions are outlined in the following Table 1.

### 4.9. Network Pharmacology Analysis

#### 4.9.1. Active Small Molecules Screening and Target Genes

A public database, Traditional Chinese Medicine Systems Pharmacology (TCMSP; https://tcmsp-e.com/browse.php?qc=herbs, version 2.3, accessed on 10 May 2022), was used to search for active small molecules in the seeds of *Raphanus sativus* Linné and roots of *Scutellaria baicalensis*. Common information on small molecules was confirmed using ChEMBL (https://www.ebi.ac.uk/chembl/, accessed on 10 May 2022), ChemSpider (https://www.chemspider.com/, accessed on 10 May 2022), National Institute of Standards and Technology (NIST; https://www.nist.gov/, accessed on 10 May 2022), and PubChem (https://pubchem.ncbi.nlm.nih.gov/, accessed on 10 May 2022). All small molecules were selected using the in silico integrative ADME model, which was screened by the index of Oral bioavailability (OB) ≥ 20% and drug-likeness (DL) ≥ 0.1, according to the TCMSP website.

Target genes linked to active small molecules in *Raphanus sativus* and *Scutellaria baicalensis* were searched for using the Search Tool in the Interactions of Chemicals and Proteins (STITCH) database (http://stitch.embl.de/, ver. 5.0, accessed on 12 May 2022) with ‘*Homo sapiens*’ selected as the organism [62]. Gene information was verified in the UniProt database (https://www.uniprot.org/, accessed on 12 May 2022), and active small molecule–protein interactions with an interaction score ≥ 0.400 (as medium confidence) were selected [63].

#### 4.9.2. Potential Target Genes and Protein–Protein Interaction (PPI)

As potential target genes, only genes overlapping with the aforementioned target genes and atopic dermatitis-related genes were searched for in the GeneCards: The Human Gene Database (https://www.genecards.org/, version 5.9, accessed on 13 May 2022) with ‘*Homo sapiens*’ and with a similarity search value ≥ 0.700 (high confidence score according to STITCH database) [64]. The protein–protein interaction (PPI) network was searched to identify the STITCH database (medium confidence score ≥ 0.400), and topology, including degree, closeness, and betweenness centrality of PPI, was calculated by Cytoscape version 3.7.2 (https://cytoscape.org/, accessed on 13 May 2022) [65].

#### 4.9.3. Signaling Pathway Analysis

Signaling pathways were analyzed using the Database for Annotation, Visualization, and Integrated Discovery (DAVID; https://david.ncifcrf.gov/, version 6.8, accessed on 13 May 2022) and KEGG: Kyoto Encyclopedia of Genes and Genomes (https://www.genome.jp/kegg/, accessed on 13 May 2022) with *p* > 0.05. The network was visualized using Cytoscape version 3.7.2 (Cytoscape, Boston, MA, USA) [66].

### 4.10. Statistical Analysis

GraphPad Prism (version 9.3.1, GraphPad Software, San Diego, CA, USA) was used for statistical analysis. One-way ANOVA followed by Tukey’s post hoc test was used to determine the statistical significance of the data. Data are expressed as mean ± SEs. For all analyses, a *p*-value of less than 0.05 was considered statistically significant.

## 5. Conclusions

This study shows that by regulating lymphocytes, the SRE application significantly suppressed DNCB-induced AD-like symptoms such as edema, inflammatory infiltration, skin barrier damage, and serum IgE levels. In addition, our network pharmacology analysis suggested the potential available pathway of SRE. Our study demonstrated SRE’s therapeutic effect on AD-like skin diseases in a mouse model. In further studies, it will be worthwhile to explore the mechanism of SRE by suggesting net-work pharmacology analysis, which could be a potential complementary candidate for AD patients.

## Figures and Tables

**Table 1:** Information of HPLC time reports.

Time (min)	Solvent	
	A (%)	B (%)
0	90	10
5	90	10
20	75	25
30	60	40
35	30	70
37	0	100
42	0	100
45	90	10
55	90	10

## Data Availability

Data are contained within the article.

## Supplementary Materials

The following supporting information can be downloaded at: https://www.mdpi.com/article/10.3390/ph17030269/s1, Table S1: List of small molecules information included in herbs of TCMSP database. Table S2: Values of small molecules and genes using the STITCH database (score ≥ 0.400). Table S3: Atopic dermatitis disease of human genes using GeneCards database.
