# Supplementary material for: Anti-Atopic Effect of Scutellaria baicalensis and Raphanus sativus on Atopic Dermatitis-like Lesions in Mice by Experimental Verification and Compound-Target Prediction
Source: Pharmaceuticals (Basel). 2024 Feb 20;17(3):269. doi: 10.3390/ph17030269 (PMC10974632; doi:10.3390/ph17030269)
Supplement: Supplementary file 1 [file pharmaceuticals-17-00269-s001.zip › pharmaceuticals-2857855-supplementary.pdf]

Supplementary Table S1. List of small molecules information included in herbs of TCMSP database

Herbs	Mol ID	Molecule Name	MW	AlogP	Hdon	Hacc	OB (%)	Caco-2	BBB	DL	FASA-	HL
Scutellariae Radix	MOL000612	(-)-alpha-Cedrene	204.39	4.12	0	0	55.56	1.81	2.16	0.1	0.24	4.82
Scutellariae Radix	MOL000228	(2R)-7-Hydroxy-5-methoxy-2-phenylchroman-4-one	270.3	2.82	1	4	55.23	0.87	0.26	0.2	0.34	17.02
Scutellariae Radix	MOL010415	11,13-Eicosadienoic acid, methyl ester	322.59	7.55	0	2	39.28	1.46	1.24	0.23	0.21	5.44
Scutellariae Radix	MOL005272	13-Tetradecenyl acetate	254.46	5.52	0	2	36.76	1.36	1.06	0.1	0.2	5.71
Scutellariae Radix	MOL012240	2',3',5,7-Tetrahydroxyflavone	286.25	2.07	4	6	25.75	0.36	-0.6	0.24	0.38	
Scutellariae Radix	MOL002911	2,6,2',4'-Tetrahydroxy-6'-methoxychaleone	302.3	2.62	4	6	69.04	-0.07	-0.32	0.22	0.34	21.89
Scutellariae Radix	MOL002917	5,2',6'-Trihydroxy-7,8-dimethoxyflavone	330.31	2.3	3	7	45.05	0.48	-0.11	0.33	0.25	16.37
Scutellariae Radix	MOL000552	5,2'-Dihydroxy-6,7,8-trimethoxyflavone	344.34	2.55	2	7	31.71	0.93	0	0.35	0.22	16.47
Scutellariae Radix	MOL002909	5,7,2,5-Tetrahydroxy-8,6-dimethoxyflavone	376.34	2.02	4	9	33.82	0.35	-0.59	0.45	0.19	15.94
Scutellariae Radix	MOL002925	5,7,2',6'-Tetrahydroxyflavone	286.25	2.07	4	6	37.01	0.18	-0.56	0.24	0.38	18
Scutellariae Radix	MOL012245	6-Methoxynaringenin	302.3	2.28	3	6	36.63	0.43	-0.32	0.27	0.28	16.12
Scutellariae Radix	MOL012246	5,7,4'-Trihydroxy-8-methoxyflavanone	302.3	2.28	3	6	74.24	0.37	-0.43	0.26	0.31	16.85
Scutellariae Radix	MOL002933	8-Methoxyapigenin	300.28	2.32	3	6	36.56	0.46	-0.4	0.27	0.31	16.93
Scutellariae Radix	MOL002908	5,8,2'-Trihydroxy-7-methoxyflavone	300.28	2.32	3	6	37.01	0.76	-0.07	0.27	0.28	16.17
Scutellariae Radix	MOL003475	9-Cedranone	220.39	3.02	0	1	67.6	1.26	1.47	0.12	0.27	4.99
Scutellariae Radix	MOL001689	Acacetin	284.28	2.59	2	5	34.97	0.67	-0.05	0.24	0.35	17.25
Scutellariae Radix	MOL000008	Apigenin	270.25	2.33	3	5	23.06	0.43	-0.61	0.21	0.41	
Scutellariae Radix	MOL002714	Baicalein	270.25	2.33	3	5	33.52	0.63	-0.05	0.21	0.36	16.25
Scutellariae Radix	MOL002935	Baicalin	460.42	0.84	6	11	29.53	-1.1	-1.97	0.77	0.37	
Scutellariae Radix	MOL000358	beta-Sitosterol	414.79	8.08	1	1	36.91	1.32	0.99	0.75	0.23	5.36
Scutellariae Radix	MOL001490	bis[(2S)-2-Ethylhexyl] benzene-1,2-dicarboxylate	390.62	7.57	0	4	43.59	0.98	0.68	0.35	0.28	3.02
Scutellariae Radix	MOL002910	Carthamidin	288.27	2.03	4	6	41.15	0.16	-0.42	0.24	0.36	15.81
Scutellariae Radix	MOL002560	Chrysin	254.25	2.6	2	4	22.61	0.7	0.01	0.18	0.42	
Scutellariae Radix	MOL001458	Coptisine	320.34	3.25	0	4	30.67	1.21	0.32	0.86	0.26	9.33
Raphani Semen	MOL000676	Dibutyl benzene-1,2-dicarboxylate	278.38	4.2	0	4	64.54	0.8	0.56	0.13	0.34	5.41
Scutellariae Radix	MOL000676	Dibutyl benzene-1,2-dicarboxylate	278.38	4.2	0	4	64.54	0.8	0.56	0.13	0.34	5.41
Scutellariae Radix	MOL002912	Dihydrobaicalin	448.41	0.6	6	11	20.85	-0.84	-1.42	0.75	0.34	
Scutellariae Radix	MOL002913	Dihydrobaicalin_qt	272.27	2.3	3	5	40.04	0.56	0.18	0.21	0.36	16.13
Scutellariae Radix	MOL002937	Dihydrooroxylin	286.3	2.55	2	5	66.06	0.67	0.13	0.23	0.31	17.17
Scutellariae Radix	MOL002926	Dihydrooroxylin A	286.3	2.55	2	5	38.72	0.71	0.03	0.23	0.29	17.58
Scutellariae Radix	MOL002879	Diisocapryl phthalate	390.62	7.44	0	4	43.59	0.79	0.26	0.39	0.28	3.6
Raphani Semen	MOL000131	Linoleic acid	280.5	6.39	1	2	41.9	1.16	0.9	0.14	0.25	7.5
Scutellariae Radix	MOL000131	Linoleic acid	280.5	6.39	1	2	41.9	1.16	0.9	0.14	0.25	7.5
Scutellariae Radix	MOL000073	ent-Epicatechin	290.29	1.92	5	6	48.96	0.02	-0.64	0.24	0.34	0.63
Scutellariae Radix	MOL002897	Epiberberine	336.39	3.45	0	4	43.09	1.17	0.4	0.78	0.19	6.1
Scutellariae Radix	MOL002914	Eriodyctiol (flavanone)	288.27	2.03	4	6	41.35	0.05	-0.66	0.24	0.39	15.88
Raphani Semen	MOL001631	Erucic acid	338.64	8.66	1	2	28.56	1.2	0.85	0.26	0.2	
Raphani Semen	MOL002203	Exceparl M-OL	296.55	7.09	0	2	31.9	1.39	1.08	0.16	0.17	5.43

Raphani Semen	MOL003975	Icosa-11,14,17-trienoic acid methyl ester	320.57	7.11	0	2	44.81	1.52	1.07	0.23	0.21	5.39
Raphani Semen	MOL010672	Icosa-8,11,14-trienoic acid methyl ester	320.57	7.11	0	2	44.81	1.49	1.09	0.23	0.23	6.16
Raphani Semen	MOL000432	Linolenic acid	278.48	5.95	1	2	45.01	1.21	0.84	0.15	0	5.54
Scutellariae Radix	MOL011081	Linolenic acid methyl ester	292.51	6.2	0	2	46.15	1.51	1.18	0.17	0.23	5.9
Raphani Semen	MOL010671	Methyl (E)-hexadec-7-enoate	268.49	6.17	0	2	34.61	1.4	1.17	0.12	0.19	5.67
Scutellariae Radix	MOL004684	Methyl (E)-octadec-2-enoate	296.55	7.51	0	2	29.84	1.42	1.4	0.17	0.18	
Raphani Semen	MOL010677	Methyl erucate	352.67	8.91	0	2	27.42	1.43	1.14	0.3	0.18	
Raphani Semen	MOL009730	Methyl icos-11-enoate	324.61	8	0	2	29.49	1.43	1.19	0.23	0.18	
Scutellariae Radix	MOL009730	Methyl icos-11-enoate	324.61	8	0	2	29.49	1.43	1.19	0.23	0.18	
Scutellariae Radix	MOL010206	Methyl isoheptadecanoate	284.54	6.87	0	2	21.72	1.39	1.28	0.14	0.16	
Raphani Semen	MOL001641	Methyl linoleate	294.53	6.64	0	2	41.93	1.44	1.08	0.17	0.21	6.05
Scutellariae Radix	MOL001889	Methyl linolelaidate	294.53	6.64	0	2	41.93	1.46	1.11	0.17	0.2	5.78
Scutellariae Radix	MOL001818	Methyl palmitelaidate	268.49	6.17	0	2	34.61	1.4	1.22	0.12	0.17	5.58
Raphani Semen	MOL010682	Methyl tetracos-15-enoate	380.73	9.82	0	2	25.62	1.5	1.17	0.37	0.17	
Raphani Semen	MOL001398	Methyl linolenate	292.51	6.2	0	2	46.15	1.48	1.09	0.17	0.24	5.95
Scutellariae Radix	MOL008206	Moslosooflavone	298.31	2.84	1	5	44.09	1.01	0.54	0.25	0.26	17.02
Scutellariae Radix	MOL002934	Neobaicalein	374.37	2.54	2	8	104.34	0.74	-0.19	0.44	0.18	16.5
Scutellariae Radix	MOL000525	Norwogonin	270.25	2.33	3	5	39.4	0.6	-0.17	0.21	0.39	16.93
Scutellariae Radix	MOL002928	Oroxylin a	284.28	2.59	2	5	41.37	0.76	0.13	0.23	0.29	17.15
Scutellariae Radix	MOL002932	Panicolin	314.31	2.57	2	6	76.26	0.84	0.31	0.29	0.24	16.78
Scutellariae Radix	MOL003568	Patchoulene	204.39	4.32	0	0	49.06	1.82	2.17	0.11	0	14.1
Scutellariae Radix	MOL012266	Rivularin	344.34	2.55	2	7	37.94	0.65	-0.13	0.37	0.21	16.25
Scutellariae Radix	MOL002915	Salvigenin	328.34	2.82	1	6	49.07	0.86	-0.03	0.33	0.21	15.87
Scutellariae Radix	MOL012267	Scutevulin	300.28	2.32	3	6	20.67	0.64	-0.13	0.27	0.29	
Scutellariae Radix	MOL000357	Sitogluside	576.95	6.34	4	6	20.63	-0.14	-0.93	0.62	0.23	
Raphani Semen	MOL000359	Sitosterol	414.79	8.08	1	1	36.91	1.32	0.87	0.75	0.22	5.37
Scutellariae Radix	MOL000359	Sitosterol	414.79	8.08	1	1	36.91	1.32	0.87	0.75	0.22	5.37
Scutellariae Radix	MOL002927	Skullcapflavone II	374.37	2.54	2	8	69.51	0.68	-0.07	0.44	0.2	16.14
Scutellariae Radix	MOL000449	Stigmasterol	412.77	7.64	1	1	43.83	1.44	1	0.76	0.22	5.57
Scutellariae Radix	MOL001506	Supraene	410.8	11.33	0	0	33.55	2.08	1.73	0.42	0.27	2.72
Scutellariae Radix	MOL000173	Wogonin	284.28	2.59	2	5	30.68	0.79	0.04	0.23	0.32	17.75
Scutellariae Radix	MOL002573	β-Patchoulene	204.39	4.32	0	0	50.69	1.79	2.16	0.11	0.22	14.26
Scutellariae Radix	MOL000396	(+)-Syringaresinol	418.48	2.1	2	8	3.29	0.47	-0.34	0.72	0	
Scutellariae Radix	MOL000018	(+/-)-Isoborneol	154.28	1.98	1	1	86.98	1.27	1.6	0.05	0	11.36
Scutellariae Radix	MOL003393	(1S,4S)-7-Isopropylidene-1,4-dimethyl-2,3,4,5,6,8-hexahydro-1H-azulene	204.39	5.13	0	0	24.38	1.86	2.07	0.07	0.23	
Raphani Semen	MOL002771	(2R)-2,5,7,8-Tetramethyl-2-[(4R,8R)-4,8,12-trimethyltridecyl]-6-chromanol	430.79	10.42	1	2	14.26	1.7	1.67	0.55	0.24	
Scutellariae Radix	MOL002921	(2S,3R,4R,5R,6S)-2-[(2R,3R,4S,5R,6R)-3,5-Dihydroxy-2-[2-(3-hydroxy-4-methoxy-phenyl)ethoxy]-6-methylol-tetrahydropyran-4-yl]oxy-6-methyl-tetrahydropyran-3,4,5-triol	476.53	-1.34	7	12	12.69	-1.62	-2.27	0.67	0.26	
Scutellariae Radix	MOL000198	(R)-Linalool	154.28	2.74	1	1	39.8	1.33	1.36	0.02	0.32	6.48
Scutellariae Radix	MOL000669	(S)-Camphor	152.26	1.94	0	1	21.68	1.28	1.74	0.05	0.28	
Scutellariae Radix	MOL000709	(S)-Matsutake alcohol	128.24	2.53	1	1	40.11	1.19	1.35	0.01	0.28	5.62
Scutellariae Radix	MOL003535	1,1,6-Trimethyl-2H-naphthalene	172.29	3.84	0	0	24.94	1.88	1.99	0.06	0.34	

Raphani Semen	MOL010666	1,1-Dimethoxy-2-methylpropane	118.2	1.18	0	2	84.11	1.22	1.45	0.01	0.16	11.26
Raphani Semen	MOL010667	1,3-Dimethyl cyclohexane (trans)	112.24	3.24	0	0	44.58	1.77	2.16	0.01	0.2	10.95
Scutellariae Radix	MOL000122	1,8-Cineole	154.28	2.15	0	1	39.73	1.57	2.06	0.05	0.24	11.29
Scutellariae Radix	MOL002916	2-(2,6-Dihydroxyphenyl)-3,5,7-trihydroxy-chromone	302.25	1.5	5	7	16.44	0.07	-0.42	0.27	0.43	
Raphani Semen	MOL010684	2-Methylhexadecanoic acid methyl ester	284.54	7.08	0	2	18.99	1.25	1.08	0.13	0.23	
Scutellariae Radix	MOL001300	2-Phenylethanol	122.18	1.55	1	1	44.03	1.11	1.13	0.02	0.37	-2.41
Scutellariae Radix	MOL012564	3,7-Dimethylnonane	156.35	4.98	0	0	15.31	1.8	2.12	0.02	0.21	
Scutellariae Radix	MOL009520	3,8-Dimethylundecane	184.41	5.89	0	0	4.72	1.81	2.02	0.03	0.2	
Raphani Semen	MOL010685	3-Methyl-5-ethylheptane	142.32	4.53	0	0	32.39	1.81	2.22	0.01	0.24	10.97
Raphani Semen	MOL005295	3-Methylundecane	170.38	5.64	0	0	6.57	1.79	1.96	0.02	0.19	
Raphani Semen	MOL010668	4-Butoxybutan-1-ol	146.26	1.48	1	2	47.43	0.75	0.49	0.02	0.17	6.16
Scutellariae Radix	MOL004464	4-Hydroxyanisole	124.15	1.55	1	2	43.98	1.27	1.33	0.02	0.32	11.69
Raphani Semen	MOL010669	4-Tridecanol	200.41	5.07	1	1	23.74	1.25	1.14	0.04	0.19	
Scutellariae Radix	MOL002922	5-(2-Hydroxyethyl)-2-methoxyphenol	168.21	1.26	2	3	31.95	0.6	0.21	0.04	0.26	4.07
Scutellariae Radix	MOL002936	5,8-Dihydroxy-6,7-dimethoxyflavone	314.31	2.57	2	6	5.74	0.97	0.09	0.29	0.28	
Raphani Semen	MOL010670	5-Methyl-2-Undecene	168.36	5.2	0	0	16.65	1.85	2.1	0.02	0.22	
Scutellariae Radix	MOL006370	5-o-Caffeoylquinic acid	354.34	-0.42	6	9	19.61	-1.39	-2.08	0.33	0.36	
Raphani Semen	MOL010673	8-Heptadecanol	256.53	6.89	1	1	14.24	1.28	0.98	0.09	0.19	
Raphani Semen	MOL010674	9-Methyl-3-Undecene	168.36	5.2	0	0	15.72	1.85	2.14	0.02	0.24	
Scutellariae Radix	MOL000169	alpha-Guaiene	204.39	4.99	0	0	25.93	1.81	2.09	0.07	0.26	
Scutellariae Radix	MOL000024	alpha-Humulene	204.39	5.04	0	0	22.98	1.88	2.08	0.06	0	
Scutellariae Radix	MOL006312	Azulol	198.33	4.91	0	0	15.15	1.94	1.83	0.07	0.38	
Scutellariae Radix	MOL000708	Benzadehyde	106.13	1.59	0	1	32.63	1.32	1.66	0.01	0.44	12.07
Scutellariae Radix	MOL000219	Benzenecarboxylic acid	121.12	0.76	0	2	31.55	0.54	0.84	0.02	0.11	12.07
Scutellariae Radix	MOL000035	beta-Selinene	204.39	4.81	0	0	24.39	1.83	2.12	0.08	0	
Raphani Semen	MOL003056	Bicyclohexane	166.34	4.69	0	0	41.3	1.79	2.19	0.04	0.16	3.53
Raphani Semen	MOL004352	Brassicasterol	398.74	7.18	1	1	14.09	1.32	0.85	0.72	0.23	
Scutellariae Radix	MOL000458	Campesterol	400.76	7.97	1	1	5.57	1.6	1.41	0.72	0.22	
Scutellariae Radix	MOL000303	Caprylic acid	144.24	2.72	1	2	16.4	0.9	1.02	0.02	0	
Scutellariae Radix	MOL002819	Catalpol	362.37	-3.77	6	10	5.07	-1.72	-2.33	0.44	0.22	
Scutellariae Radix	MOL000007	Cosmetin	432.41	0.43	6	10	9.68	-1.08	-2.26	0.74	0.32	
Raphani Semen	MOL010683	Cyclohexylethanol	128.24	2.22	1	1	53.98	1.09	1.2	0.02	0.17	-3.27
Scutellariae Radix	MOL002923	Darendoside B	476.53	-1.46	7	12	10.75	-1.59	-2	0.59	0.24	
Scutellariae Radix	MOL002924	Darendoside B_qt	330.37	-0.6	5	8	10.05	-0.74	-1.11	0.22	0.23	
Scutellariae Radix	MOL013062	Di(phenyl)methanone	182.23	3.23	0	1	58.62	1.44	1.52	0.06	0.49	21.31
Scutellariae Radix	MOL011322	Diisobutyl succinate	230.34	2.48	0	4	39.54	0.69	0.29	0.06	0.25	4.81
Scutellariae Radix	MOL005021	Dimethyl benzene-1,2-dicarboxylate	194.2	1.54	0	4	57.4	0.64	0.63	0.06	0	5.56
Raphani Semen	MOL007621	Dimethyldisulfide	94.22	1.39	0	0	39.27	2.03	2.18	0	0.51	12.5
Raphani Semen	MOL010675	Dimetilsolfato	126.15	-0.53	0	4	55.11	0.8	0.49	0.01	0.09	11.68
Scutellariae Radix	MOL007197	Diphenylamine	169.24	3.38	1	1	31.13	1.85	1.87	0.05	0.49	7.72
Scutellariae Radix	MOL000717	d-Isomenthone	154.28	2.6	0	1	61.2	1.35	1.74	0.03	0.23	10.86

Scutellariae Radix	MOL000885	Dodekan	170.38	5.85	0	0	17.74	1.79	1.96	0.02	0.16	
Scutellariae Radix	MOL000254	Eugenol	164.22	2.55	1	2	56.24	1.35	1.32	0.04	0.32	0.92
Scutellariae Radix	MOL002918	Ganhuangenin	346.31	2.03	4	8	1.34	0.28	-0.57	0.37	0.25	
Scutellariae Radix	MOL003127	Germacrene D	204.39	5.14	0	0	19.22	1.83	2.02	0.06	0	
Raphani Semen	MOL010676	Glyceron sinapate	710.74	5.23	3	15	5.18	-0.33	-1.73	0.37	0.26	
Scutellariae Radix	MOL000869	Henicosane	296.65	9.95	0	0	8.41	1.84	1.8	0.15	0.13	
Scutellariae Radix	MOL003055	Heptadecyloxirane	282.57	7.61	0	1	12.44	1.67	1.7	0.15	0.15	
Scutellariae Radix	MOL000867	Heptadekan	240.53	8.13	0	0	8.64	1.84	1.87	0.07	0.14	
Scutellariae Radix	MOL002046	Hexanoic acid	116.18	1.81	1	2	73.08	0.8	0.93	0.01	0.27	10.81
Scutellariae Radix	MOL000870	Hexatriacontane	507.1	16.8	0	0	7.95	1.99	1.61	0.41	0.11	
Scutellariae Radix	MOL000714	Hyacinthin	120.16	1.52	0	1	38.65	1.31	1.58	0.02	0.43	-2.04
Scutellariae Radix	MOL001578	Hypnon	120.16	1.57	0	1	48.19	1.36	1.54	0.02	0.45	25.2
Scutellariae Radix	MOL000868	Icosane	282.62	9.5	0	0	8.46	1.83	1.8	0.13	0.13	
Scutellariae Radix	MOL007792	Isomartynoside	652.71	0.89	7	15	13.98	-1.6	-2.44	0.56	0.29	
Scutellariae Radix	MOL000789	Jatrorrizine	338.41	3.4	1	4	19.65	1.28	0.36	0.59	0	
Raphani Semen	MOL000663	Lignoceric acid	368.72	10.02	1	2	14.9	1.24	1.01	0.33	0.17	
Scutellariae Radix	MOL000715	l-Menthone	154.28	2.6	0	1	57.9	1.35	1.7	0.03	0.24	10.57
Scutellariae Radix	MOL001132	Longipinene	204.39	4.12	0	0	17.01	1.84	2.1	0.12	0.23	
Raphani Semen	MOL010678	Methanethiol	48.12	0.61	0	0	16.71	1.39	1.72	0	0.53	
Scutellariae Radix	MOL010563	Methyl (Z)-cinnamate	162.2	2.15	0	2	37.2	1.31	1.56	0.04	0.35	7.75
Scutellariae Radix	MOL008615	Methyl 9-oxononanoate	186.28	2.17	0	3	24.02	0.62	0.17	0.04	0.2	
Raphani Semen	MOL002027	Methyl behenate	354.69	9.36	0	2	14.96	1.45	1.2	0.29	0.16	
Scutellariae Radix	MOL002027	Methyl behenate	354.69	9.36	0	2	14.96	1.45	1.2	0.29	0.16	
Scutellariae Radix	MOL006219	Methyl benzoate	136.16	1.69	0	2	45.99	1.25	1.41	0.02	0.4	-2.16
Scutellariae Radix	MOL008595	Methyl henicosanoate	340.66	8.9	0	2	15.36	1.42	1.17	0.26	0.15	
Scutellariae Radix	MOL013161	Methyl hexacosanoate	410.81	11.18	0	2	13.68	1.49	1.11	0.43	0.15	
Raphani Semen	MOL003920	Methyl icosanoate	326.63	8.44	0	2	15.79	1.43	1.12	0.22	0.15	
Scutellariae Radix	MOL003920	Methyl icosanoate	326.63	8.44	0	2	15.79	1.43	1.12	0.22	0.15	
Scutellariae Radix	MOL001386	Methyl laurate	214.39	4.79	0	2	21.75	1.32	1.24	0.05	0.15	
Scutellariae Radix	MOL009734	Methyl lignocerate	382.75	10.27	0	2	14.27	1.47	0.94	0.37	0.13	
Scutellariae Radix	MOL005402	Methyl margarate	284.54	7.08	0	2	17.41	1.37	1.25	0.14	0.17	
Scutellariae Radix	MOL000654	Methyl montanate	438.87	12.09	0	2	13.2	1.51	0.99	0.48	0.14	
Scutellariae Radix	MOL001392	Methyl myristate	242.45	5.71	0	2	19.68	1.36	1.19	0.08	0.14	
Scutellariae Radix	MOL008151	Methyl nonadecanoate	312.6	7.99	0	2	16.27	1.4	1.18	0.19	0.15	
Scutellariae Radix	MOL004682	Methyl octylate	158.27	2.97	0	2	18.71	1.23	1.32	0.02	0.18	
Raphani Semen	MOL000879	Methyl palmitate	270.51	6.62	0	2	18.09	1.37	1.18	0.12	0.14	
Scutellariae Radix	MOL000879	Methyl palmitate	270.51	6.62	0	2	18.09	1.37	1.18	0.12	0.14	
Raphani Semen	MOL001817	Methyl stearate	298.57	7.53	0	2	16.8	1.41	1.29	0.16	0.16	
Scutellariae Radix	MOL001817	Methyl stearate	298.57	7.53	0	2	16.8	1.41	1.29	0.16	0.16	
Scutellariae Radix	MOL005368	Methyl tricosanoate	368.72	9.81	0	2	14.61	1.43	1.08	0.33	0.16	
Raphani Semen	MOL007628	Methyl trisulfide	126.29	1.98	0	0	10.72	2.01	2.27	0	0.58	

Scutellariae Radix	MOL001393	Myristic acid	228.42	5.46	1	2	21.18	1.07	0.99	0.07	0.19	
Raphani Semen	MOL000116	Nonanal	142.27	3.22	0	1	40.28	1.31	1.5	0.02	0.21	6.35
Scutellariae Radix	MOL003050	Nonanoic acid	158.27	3.17	1	2	40.51	0.92	1.08	0.02	0.23	4.15
Scutellariae Radix	MOL002137	Octane	114.26	4.02	0	0	29.72	1.78	2.02	0.01	0.19	
Raphani Semen	MOL001285	Octanol	130.26	2.8	1	1	21.06	1.16	1.23	0.01	0.17	
Scutellariae Radix	MOL013068	Oroxindin	459.41	0.21	4	11	7.07	-1.68	-2.15	0.77	0.04	
Raphani Semen	MOL005120	Palmitaldehyde	240.48	6.42	0	1	16.54	1.44	1.28	0.08	0.18	
Raphani Semen	MOL000069	Palmitic acid	256.48	6.37	1	2	19.3	1.09	1	0.1	0	
Scutellariae Radix	MOL000069	Palmitic acid	256.48	6.37	1	2	19.3	1.09	1	0.1	0	
Scutellariae Radix	MOL000771	p-Coumaric acid	164.17	1.64	2	3	43.29	0.46	0.13	0.04	0.45	4.43
Scutellariae Radix	MOL000864	Pentadecane	212.47	7.22	0	0	13.98	1.81	1.92	0.05	0.15	
Raphani Semen	MOL006934	Pentane-1,5-diol	104.17	0.2	2	2	24.8	0.02	-0.56	0.01	0.17	
Scutellariae Radix	MOL001972	Pulegone	152.26	2.75	0	1	51.6	1.39	1.74	0.03	0.26	11.19
Raphani Semen	MOL010679	Raphanin	175.3	0.77	0	2	61.47	0.69	0.65	0.02	0.47	4.61
Scutellariae Radix	MOL002929	Salidroside	300.34	-0.47	5	7	7.01	-0.82	-1.41	0.2	0.3	
Scutellariae Radix	MOL002737	Scutellarein	286.25	2.07	4	6	18.97	0.31	-0.54	0.24	0.35	
Scutellariae Radix	MOL002931	Scutellarin	462.39	0.37	7	12	2.64	-1.08	-2.13	0.79	0.36	
Raphani Semen	MOL010680	Sinapine	310.41	0.64	1	5	1.21	0.69	0.2	0.18	0.19	
Raphani Semen	MOL010681	Sinapine thiocyanate	310.41	0.64	1	5	1.21	0.66	0.17	0.18	0.12	
Raphani Semen	MOL000860	Stearic acid	284.54	7.28	1	2	17.83	1.15	1.22	0.14	0.19	
Scutellariae Radix	MOL000860	Stearic acid	284.54	7.28	1	2	17.83	1.15	1.22	0.14	0.19	
Scutellariae Radix	MOL002202	Tetramethylpyrazine	136.22	0.66	0	2	20.01	1.19	1.05	0.03	0.31	
Scutellariae Radix	MOL005224	Tetratetracotane	619.34	20.44	0	0	7.82	2.04	1.22	0.25	0.16	
Raphani Semen	MOL006077	Thiamine	265.4	-0.05	3	4	19.87	-0.32	-1.14	0.11	0.15	
Raphani Semen	MOL008159	Triacontane	422.92	14.06	0	0	8.09	1.91	1.46	0.41	0.16	
Scutellariae Radix	MOL005841	Tributyl phosphate	266.36	3.64	0	4	27.76	1.18	0.9	0.06	0	
Scutellariae Radix	MOL000610	Tridecane	184.41	6.3	0	0	17.89	1.78	1.96	0.03	0.19	
Raphani Semen	MOL003527	Tyranton	116.18	-0.08	1	2	58.34	0.49	0.37	0.01	0.31	11.21
Scutellariae Radix	MOL002930	Tyrosol	138.18	1.28	2	2	33.81	0.65	0.25	0.02	0.36	-2.55
Scutellariae Radix	MOL005577	Undecanal	170.33	4.13	0	1	22.9	1.38	1.41	0.03	0.2	
Scutellariae Radix	MOL002378	Undecane	156.35	5.39	0	0	17.15	1.79	2.02	0.02	0.18	
Scutellariae Radix	MOL002919	Viscidulin III	346.31	1.74	4	8	14.36	0.27	-0.27	0.37	0.27	
Raphani Semen	MOL001691	Vitamin c	176.14	-1.76	4	6	13.34	-0.86	-1.38	0.04	0.37	
Raphani Semen	MOL002730	Vitamin- G	376.41	0.23	5	10	6.79	-1.22	-1.77	0.5	0.32	

Supplementary Table S2. Values of small molecules and genes using the STITCH database (score≥0.400)

node No.	node	accession	annotation	score
**node 1**	**Acacetin**	**5280442**	**Acacetin is an O-methylated flavone found in "Robinia pseudoacacia" (black locust), "Turnera diffusa" (damiana), "Betula pendula" (silver birch), and in the fern "Asplenium normale".**	
**node 2**	CYP1A1	ENSP00000369050	cytochrome P450, family 1, subfamily A, polypeptide 1; Cytochromes P450 are a group of heme-thiolate monooxygenases. In liver microsomes, this enzyme is involved in an NADPH-dependent electron transport pathway. It oxidizes a variety of structurally unrelated compounds, including steroids, fatty acids, and xenobiotics	0.869
	CYP1A2	ENSP00000342007	cytochrome P450, family 1, subfamily A, polypeptide 2; Cytochromes P450 are a group of heme-thiolate monooxygenases. In liver microsomes, this enzyme is involved in an NADPH-dependent electron transport pathway. It oxidizes a variety of structurally unrelated compounds, including steroids, fatty acids, and xenobiotics. Most active in catalyzing 2-hydroxylation. Caffeine is metabolized primarily by cytochrome CYP1A2 in the liver through an initial N3-demethylation. Also acts in the metabolism of aflatoxin B1 and acetaminophen. Participates in the bioactivation of carcinogenic aromatic a [...]	0.869
	CYP1B1	ENSP00000260630	cytochrome P450, family 1, subfamily B, polypeptide 1; Cytochromes P450 are a group of heme-thiolate monooxygenases. In liver microsomes, this enzyme is involved in an NADPH-dependent electron transport pathway. It oxidizes a variety of structurally unrelated compounds, including steroids, fatty acids, and xenobiotics	0.905
	IL13	ENSP00000304915	interleukin 13; Cytokine. Inhibits inflammatory cytokine production. Synergizes with IL2 in regulating interferon-gamma synthesis. May be critical in regulating inflammatory and immune responses	0.8
	IL5	ENSP00000231454	interleukin 5 (colony-stimulating factor, eosinophil); Factor that induces terminal differentiation of late- developing B-cells to immunoglobulin secreting cells	0.8
	JUN	ENSP00000360266	jun proto-oncogene; Transcription factor that recognizes and binds to the enhancer heptamer motif 5'-TGA[CG]TCA-3'. Promotes activity of NR5A1 when phosphorylated by HIPK3 leading to increased steroidogenic gene expression upon cAMP signaling pathway stimulation	0.8
	NR1I2	ENSP00000336528	nuclear receptor subfamily 1, group I, member 2	0.7
	SELE	ENSP00000331736	selectin E; Cell-surface glycoprotein having a role in immunoadhesion. Mediates in the adhesion of blood neutrophils in cytokine-activated endothelium through interaction with PSGL1/SELPLG. May have a role in capillary morphogenesis	0.8
	STAT1	ENSP00000354394	signal transducer and activator of transcription 1, 91kDa	0.82
	VEGFA	ENSP00000361125	vascular endothelial growth factor A	0.8
**node1**	**Apigenin**	**5280443**	**Apigenin (4â,5,7-trihydroxyflavone), found in many plants, is a natural product belonging to the flavone class that is the aglycone of several naturally occurring glycosides. It is a yellow crystalline solid that has been used to dye wool.**	
**node2**	ABCB1	ENSP00000265724	ATP-binding cassette, sub-family B (MDR/TAP), member 1; Energy-dependent efflux pump responsible for decreased drug accumulation in multidrug-resistant cells	0.814
	ABCC1	ENSP00000382342	ATP-binding cassette, sub-family C (CFTR/MRP), member 1	0.725
	ACHE	ENSP00000303211	acetylcholinesterase	0.651
	AKT1	ENSP00000270202	v-akt murine thymoma viral oncogene homolog 1; AKT1 is one of 3 closely related serine/threonine- protein kinases (AKT1, AKT2 and AKT3) called the AKT kinase, and which regulate many processes including metabolism, proliferation, cell survival, growth and angiogenesis. This is mediated through serine and/or threonine phosphorylation of a range of downstream substrates. Over 100 substrate candidates have been reported so far, but for most of them, no isoform specificity has been reported. AKT is responsible of the regulation of glucose uptake by mediating insulin-induced translocation o [...]	0.876
	AXL	ENSP00000301178	AXL receptor tyrosine kinase; Receptor tyrosine kinase that transduces signals from the extracellular matrix into the cytoplasm by binding growth factor GAS6 and which is thus regulating many physiological processes including cell survival, cell proliferation, migration and differentiation. Ligand binding at the cell surface induces dimerization and autophosphorylation of AXL. Following activation by ligand, ALX binds and induces tyrosine phosphorylation of PI3- kinase subunits PIK3R1, PIK3R2 and PIK3R3; but also GRB2, PLCG1, LCK and PTPN11. Other downstream substrate candidates for AX [...]	0.8
	BCL2	ENSP00000329623	B-cell CLL/lymphoma 2; Suppresses apoptosis in a variety of cell systems including factor-dependent lymphohematopoietic and neural cells. Regulates cell death by controlling the mitochondrial membrane permeability. Appears to function in a feedback loop system with caspases. Inhibits caspase activity either by preventing the release of cytochrome c from the mitochondria and/or by binding to the apoptosis-activating factor (APAF-1)	0.7
	BIRC3	ENSP00000263464	baculoviral IAP repeat containing 3; Multi-functional protein which regulates not only caspases and apoptosis, but also modulates inflammatory signaling and immunity, mitogenic kinase signaling and cell proliferation, as well as cell invasion and metastasis. Acts as an E3 ubiquitin- protein ligase regulating NF-kappa-B signaling and regulates both canonical and non-canonical NF-kappa-B signaling by acting in opposite directions- acts as a positive regulator of the canonical pathway and suppresses constitutive activation of non-canonical NF-kappa-B signaling. The target proteins for its [...]	0.7
	CASP3	ENSP00000311032	caspase 3, apoptosis-related cysteine peptidase; Involved in the activation cascade of caspases responsible for apoptosis execution. At the onset of apoptosis it proteolytically cleaves poly(ADP-ribose) polymerase (PARP) at a '216-Asp-|-Gly-217' bond. Cleaves and activates sterol regulatory element binding proteins (SREBPs) between the basic helix-loop- helix leucine zipper domain and the membrane attachment domain. Cleaves and activates caspase-6, -7 and -9. Involved in the cleavage of huntingtin. Triggers cell adhesion in sympathetic neurons through RET cleavage	0.947
	CASP7	ENSP00000358327	caspase 7, apoptosis-related cysteine peptidase	0.7
	CASP8	ENSP00000351273	caspase 8, apoptosis-related cysteine peptidase	0.734
	CASP9	ENSP00000330237	caspase 9, apoptosis-related cysteine peptidase	0.726
	CAT	ENSP00000241052	catalase; Occurs in almost all aerobically respiring organisms and serves to protect cells from the toxic effects of hydrogen peroxide. Promotes growth of cells including T-cells, B-cells, myeloid leukemia cells, melanoma cells, mastocytoma cells and normal and transformed fibroblast cells	0.822
	CCND1	ENSP00000227507	cyclin D1; Regulatory component of the cyclin D1-CDK4 (DC) complex that phosphorylates and inhibits members of the retinoblastoma (RB) protein family including RB1 and regulates the cell-cycle during G(1)/S transition. Phosphorylation of RB1 allows dissociation of the transcription factor E2F from the RB/E2F complex and the subsequent transcription of E2F target genes which are responsible for the progression through the G(1) phase. Hypophosphorylates RB1 in early G(1) phase. Cyclin D-CDK4 complexes are major integrators of various mitogenenic and antimitogenic signals. Also substrate [...]	0.838
	CD38	ENSP00000226279	CD38 molecule; Synthesizes cyclic ADP-ribose, a second messenger for glucose-induced insulin secretion. Also has cADPr hydrolase activity. Also moonlights as a receptor in cells of the immune system	0.8
	CDK1	ENSP00000378699	cyclin-dependent kinase 1; Plays a key role in the control of the eukaryotic cell cycle by modulating the centrosome cycle as well as mitotic onset; promotes G2-M transition, and regulates G1 progress and G1-S transition via association with multiple interphase cyclins. Required in higher cells for entry into S-phase and mitosis. Phosphorylates PARVA/actopaxin, APC, AMPH, APC, BARD1, Bcl- xL/BCL2L1, BRCA2, CALD1, CASP8, CDC7, CDC20, CDC25A, CDC25C, CC2D1A, CSNK2 proteins/CKII, FZR1/CDH1, CDK7, CEBPB, CHAMP1, DMD/dystrophin, EEF1 proteins/EF-1, EZH2, KIF11/EG5, EGFR, FANCG, FOS, GFAP, G [...]	0.949
	CFTR	ENSP00000003084	cystic fibrosis transmembrane conductance regulator (ATP-binding cassette sub-family C, member 7); Involved in the transport of chloride ions. May regulate bicarbonate secretion and salvage in epithelial cells by regulating the SLC4A7 transporter. Can inhibit the chloride channel activity of ANO1	0.722
	CHCHD7	ENSP00000306425	coiled-coil-helix-coiled-coil-helix domain containing 7	0.441
	COQ6	ENSP00000333946	coenzyme Q6 homolog, monooxygenase (S. cerevisiae)	0.424
	CSNK2A1	ENSP00000217244	casein kinase 2, alpha 1 polypeptide; Catalytic subunit of a constitutively active serine/threonine-protein kinase complex that phosphorylates a large number of substrates containing acidic residues C-terminal to the phosphorylated serine or threonine. Regulates numerous cellular processes, such as cell cycle progression, apoptosis and transcription, as well as viral infection. May act as a regulatory node which integrates and coordinates numerous signals leading to an appropriate cellular response. During mitosis, functions as a component of the p53/TP53-dependent spindle assembly che [...]	0.817
	CXCR4	ENSP00000386884	chemokine (C-X-C motif) receptor 4; Receptor for the C-X-C chemokine CXCL12/SDF-1 that transduces a signal by increasing intracellular calcium ion levels and enhancing MAPK1/MAPK3 activation. Acts as a receptor for extracellular ubiquitin; leading to enhanced intracellular calcium ions and reduced cellular cAMP levels. Involved in hematopoiesis and in cardiac ventricular septum formation. Also plays an essential role in vascularization of the gastrointestinal tract, probably by regulating vascular branching and/or remodeling processes in endothelial cells. Involved in cerebellar develo [...]	0.8
	CYP19A1	ENSP00000260433	cytochrome P450, family 19, subfamily A, polypeptide 1; Catalyzes the formation of aromatic C18 estrogens from C19 androgens	0.837
	CYP1A1	ENSP00000369050	cytochrome P450, family 1, subfamily A, polypeptide 1; Cytochromes P450 are a group of heme-thiolate monooxygenases. In liver microsomes, this enzyme is involved in an NADPH-dependent electron transport pathway. It oxidizes a variety of structurally unrelated compounds, including steroids, fatty acids, and xenobiotics	0.814
	CYP1A2	ENSP00000342007	cytochrome P450, family 1, subfamily A, polypeptide 2; Cytochromes P450 are a group of heme-thiolate monooxygenases. In liver microsomes, this enzyme is involved in an NADPH-dependent electron transport pathway. It oxidizes a variety of structurally unrelated compounds, including steroids, fatty acids, and xenobiotics. Most active in catalyzing 2-hydroxylation. Caffeine is metabolized primarily by cytochrome CYP1A2 in the liver through an initial N3-demethylation. Also acts in the metabolism of aflatoxin B1 and acetaminophen. Participates in the bioactivation of carcinogenic aromatic a [...]	0.807
	CYP1B1	ENSP00000260630	cytochrome P450, family 1, subfamily B, polypeptide 1; Cytochromes P450 are a group of heme-thiolate monooxygenases. In liver microsomes, this enzyme is involved in an NADPH-dependent electron transport pathway. It oxidizes a variety of structurally unrelated compounds, including steroids, fatty acids, and xenobiotics	0.876
	CYP3A4	ENSP00000337915	cytochrome P450, family 3, subfamily A, polypeptide 4; Cytochromes P450 are a group of heme-thiolate monooxygenases. In liver microsomes, this enzyme is involved in an NADPH-dependent electron transport pathway. It performs a variety of oxidation reactions (e.g. caffeine 8-oxidation, omeprazole sulphoxidation, midazolam 1'-hydroxylation and midazolam 4- hydroxylation) of structurally unrelated compounds, including steroids, fatty acids, and xenobiotics. Acts as a 1,8-cineole 2- exo-monooxygenase. The enzyme also hydroxylates etoposide	0.7

EGF	ENSP00000265171	epidermal growth factor; EGF stimulates the growth of various epidermal and epithelial tissues in vivo and in vitro and of some fibroblasts in cell culture. Magnesiotropic hormone that stimulates magnesium reabsorption in the renal distal convoluted tubule via engagement of EGFR and activation of the magnesium channel TRPM6	0.8
ELAVL1	ENSP00000385269	ELAV (embryonic lethal, abnormal vision, Drosophila)-like 1 (Hu antigen R); Involved in 3'-UTR ARE-mediated MYC stabilization. Binds avidly to the AU-rich element in FOS and IL3/interleukin-3 mRNAs. In the case of the FOS AU-rich element, HUR binds to a core element of 27 nucleotides that contain AUUUA, AUUUUA and AUUUUUA motifs. Binds preferentially to the 5'-UUUU[AG]UUU-3' motif in vitro	0.826
ESR1	ENSP00000206249	estrogen receptor 1; Nuclear hormone receptor. The steroid hormones and their receptors are involved in the regulation of eukaryotic gene expression and affect cellular proliferation and differentiation in target tissues. Ligand-dependent nuclear transactivation involves either direct homodimer binding to a palindromic estrogen response element (ERE) sequence or association with other DNA- binding transcription factors, such as AP-1/c-Jun, c-Fos, ATF-2, Sp1 and Sp3, to mediate ERE-independent signaling. Ligand binding induces a conformational change allowing subsequent or combinatorial [...]	0.961
ESR2	ENSP00000343925	estrogen receptor 2 (ER beta)	0.847
FAS	ENSP00000347979	Fas (TNF receptor superfamily, member 6)	0.786
FOXO1	ENSP00000368880	forkhead box O1; Transcription factor that is the main target of insulin signaling and regulates metabolic homeostasis in response to oxidative stress. Binds to the insulin response element (IRE) with consensus sequence 5'-TT[G/A]TTTTG-3' and the related Daf-16 family binding element (DBE) with consensus sequence 5'- TT[G/A]TTTAC-3'. Activity suppressed by insulin. Main regulator of redox balance and osteoblast numbers and controls bone mass. Orchestrates the endocrine function of the skeleton in regulating glucose metabolism. Acts syngernistically with ATF4 to suppress osteocalcin/BGL [...]	0.8
GAD1	ENSP00000350928	glutamate decarboxylase 1 (brain, 67kDa); Catalyzes the production of GABA	0.8
GMNN	ENSP00000230056	geminin, DNA replication inhibitor; Inhibits DNA replication by preventing the incorporation of MCM complex into pre-replication complex (pre-RC). It is degraded during the mitotic phase of the cell cycle. Its destruction at the metaphase-anaphase transition permits replication in the succeeding cell cycle	0.8
HIF1A	ENSP00000338018	hypoxia inducible factor 1, alpha subunit (basic helix-loop-helix transcription factor); Functions as a master transcriptional regulator of the adaptive response to hypoxia. Under hypoxic conditions, activates the transcription of over 40 genes, including erythropoietin, glucose transporters, glycolytic enzymes, vascular endothelial growth factor, HILPDA, and other genes whose protein products increase oxygen delivery or facilitate metabolic adaptation to hypoxia. Plays an essential role in embryonic vascularization, tumor angiogenesis and pathophysiology of ischemic disease. Binds to [...]	0.824
IKBKB	ENSP00000430684	inhibitor of kappa light polypeptide gene enhancer in B-cells, kinase beta; Serine kinase that plays an essential role in the NF- kappa-B signaling pathway which is activated by multiple stimuli such as inflammatory cytokines, bacterial or viral products, DNA damages or other cellular stresses. Acts as part of the canonical IKK complex in the conventional pathway of NF-kappa-B activation and phosphorylates inhibitors of NF-kappa-B on 2 critical serine residues. These modifications allow polyubiquitination of the inhibitors and subsequent degradation by the proteasome. In turn, free NF- [...]	0.7
KMO	ENSP00000355517	kynurenine 3-monooxygenase (kynurenine 3-hydroxylase); Catalyzes the hydroxylation of L-kynurenine (L-Kyn) to form 3-hydroxy-L-kynurenine (L-3OHKyn). Required for synthesis of quinolinic acid, a neurotoxic NMDA receptor antagonist and potential endogenous inhibitor of NMDA receptor signaling in axonal targeting, synaptogenesis and apoptosis during brain development. Quinolinic acid may also affect NMDA receptor signaling in pancreatic beta cells, osteoblasts, myocardial cells, and the gastrointestinal tract (By similarity)	0.424
MAOA	ENSP00000340684	monoamine oxidase A; Catalyzes the oxidative deamination of biogenic and xenobiotic amines and has important functions in the metabolism of neuroactive and vasoactive amines in the central nervous system and peripheral tissues. MAOA preferentially oxidizes biogenic amines such as 5-hydroxytryptamine (5-HT), norepinephrine and epinephrine	0.848
MAOB	ENSP00000367309	monoamine oxidase B; Catalyzes the oxidative deamination of biogenic and xenobiotic amines and has important functions in the metabolism of neuroactive and vasoactive amines in the central nervous system and peripheral tissues. MAOB preferentially degrades benzylamine and phenylethylamine	0.8
MAPK1	ENSP00000215832	mitogen-activated protein kinase 1; Serine/threonine kinase which acts as an essential component of the MAP kinase signal transduction pathway. MAPK1/ERK2 and MAPK3/ERK1 are the 2 MAPKs which play an important role in the MAPK/ERK cascade. They participate also in a signaling cascade initiated by activated KIT and KITLG/SCF. Depending on the cellular context, the MAPK/ERK cascade mediates diverse biological functions such as cell growth, adhesion, survival and differentiation through the regulation of transcription, translation, cytoskeletal rearrangements. The MAPK/ERK cascade plays a [...]	0.7
MAPK3	ENSP00000263025	mitogen-activated protein kinase 3; Serine/threonine kinase which acts as an essential component of the MAP kinase signal transduction pathway. MAPK1/ERK2 and MAPK3/ERK1 are the 2 MAPKs which play an important role in the MAPK/ERK cascade. They participate also in a signaling cascade initiated by activated KIT and KITLG/SCF. Depending on the cellular context, the MAPK/ERK cascade mediates diverse biological functions such as cell growth, adhesion, survival and differentiation through the regulation of transcription, translation, cytoskeletal rearrangements. The MAPK/ERK cascade plays a [...]	0.755
MMP9	ENSP00000361405	matrix metallopeptidase 9 (gelatinase B, 92kDa gelatinase, 92kDa type IV collagenase); May play an essential role in local proteolysis of the extracellular matrix and in leukocyte migration. Could play a role in bone osteoclastic resorption. Cleaves KiSS1 at a Gly-|-Leu bond. Cleaves type IV and type V collagen into large C-terminal three quarter fragments and shorter N-terminal one quarter fragments. Degrades fibronectin but not laminin or Pz-peptide	0.842
NOX4	ENSP00000263317	NADPH oxidase 4; Constitutive NADPH oxidase which generates superoxide intracellularly upon formation of a complex with CYBA/p22phox. Regulates signaling cascades probably through phosphatases inhibition. May function as an oxygen sensor regulating the KCNK3/TASK-1 potassium channel and HIF1A activity. May regulate insulin signaling cascade. May play a role in apoptosis, bone resorption and lipolysaccharide-mediated activation of NFKB. May produce superoxide in the nucleus and play a role in regulating gene expression upon cell stimulation. Isoform 3 is not functional. Isoform 4 displa [...]	0.411
NR1I2	ENSP00000336528	nuclear receptor subfamily 1, group I, member 2	0.7
PARP1	ENSP00000355759	poly (ADP-ribose) polymerase 1; Involved in the base excision repair (BER) pathway, by catalyzing the poly(ADP-ribosyl)ation of a limited number of acceptor proteins involved in chromatin architecture and in DNA metabolism. This modification follows DNA damages and appears as an obligatory step in a detection/signaling pathway leading to the reparation of DNA strand breaks. Mediates the poly(ADP- ribosyl)ation of APLF and CHFR. Positively regulates the transcription of MTUS1 and negatively regulates the transcription of MTUS2/TIP150. With EEF1A1 and TXK, forms a complex that acts as a [...]	0.944
POMC	ENSP00000264708	proopiomelanocortin; ACTH stimulates the adrenal glands to release cortisol	0.8
PTGS2	ENSP00000356438	prostaglandin-endoperoxide synthase 2 (prostaglandin G/H synthase and cyclooxygenase); Mediates the formation of prostaglandins from arachidonate. May have a role as a major mediator of inflammation and/or a role for prostanoid signaling in activity-dependent plasticity	0.877
PTK2	ENSP00000341189	PTK2 protein tyrosine kinase 2; Non-receptor protein-tyrosine kinase that plays an essential role in regulating cell migration, adhesion, spreading, reorganization of the actin cytoskeleton, formation and disassembly of focal adhesions and cell protrusions, cell cycle progression, cell proliferation and apoptosis. Required for early embryonic development and placenta development. Required for embryonic angiogenesis, normal cardiomyocyte migration and proliferation, and normal heart development. Regulates axon growth and neuronal cell migration, axon branching and synapse formation; req [...]	0.8
PTPN11	ENSP00000340944	protein tyrosine phosphatase, non-receptor type 11; Acts downstream of various receptor and cytoplasmic protein tyrosine kinases to participate in the signal transduction from the cell surface to the nucleus. Dephosphorylates ROCK2 at Tyr-722 resulting in stimulatation of its RhoA binding activity	0.8
SLC2A1	ENSP00000416293	solute carrier family 2 (facilitated glucose transporter), member 1; Facilitative glucose transporter. This isoform may be responsible for constitutive or basal glucose uptake. Has a very broad substrate specificity; can transport a wide range of aldoses including both pentoses and hexoses	0.8
SPAM1	ENSP00000345849	sperm adhesion molecule 1 (PH-20 hyaluronidase, zona pellucida binding); Involved in sperm-egg adhesion. Upon fertilization sperm must first penetrate a layer of cumulus cells that surrounds the egg before reaching the zona pellucida. The cumulus cells are embedded in a matrix containing hyaluronic acid which is formed prior to ovulation. This protein aids in penetrating the layer of cumulus cells by digesting hyaluronic acid	0.848
SQLE	ENSP00000265896	Supraene epoxidase; Catalyzes the first oxygenation step in sterol biosynthesis and is suggested to be one of the rate-limiting enzymes in this pathway	0.424
SRC	ENSP00000350941	v-src sarcoma (Schmidt-Ruppin A-2) viral oncogene homolog (avian); Non-receptor protein tyrosine kinase which is activated following engagement of many different classes of cellular receptors including immune response receptors, integrins and other adhesion receptors, receptor protein tyrosine kinases, G protein- coupled receptors as well as cytokine receptors. Participates in signaling pathways that control a diverse spectrum of biological activities including gene transcription, immune response, cell adhesion, cell cycle progression, apoptosis, migration, and transformation. Due to f [...]	0.8
SULT1A1	ENSP00000321988	sulfotransferase family, cytosolic, 1A, phenol-preferring, member 1; Sulfotransferase that utilizes 3'-phospho-5'-adenylyl sulfate (PAPS) as sulfonate donor to catalyze the sulfate conjugation of catecholamines, phenolic drugs and neurotransmitters. Has also estrogen sulfotransferase activity. responsible for the sulfonation and activation of minoxidil. Is Mediates the metabolic activation of carcinogenic N- hydroxyarylamines to DNA binding products and could so participate as modulating factor of cancer risk	0.7
TAGLN	ENSP00000278968	transgelin; Actin cross-linking/gelling protein (By similarity). Involved in calcium interactions and contractile properties of the cell that may contribute to replicative senescence	0.8
TNF	ENSP00000398698	tumor necrosis factor	0.692
TNFRSF10B	ENSP00000276431	tumor necrosis factor receptor superfamily, member 10b; Receptor for the cytotoxic ligand TNFSF10/TRAIL. The adapter molecule FADD recruits caspase-8 to the activated receptor. The resulting death-inducing signaling complex (DISC) performs caspase-8 proteolytic activation which initiates the subsequent cascade of caspases (aspartate-specific cysteine proteases) mediating apoptosis. Promotes the activation of NF- kappa-B. Essential for ER stress-induced apoptosis	0.819
TNKS2	ENSP00000360689	tankyrase, TRF1-interacting ankyrin-related ADP-ribose polymerase 2; Poly-ADP-ribosyltransferase involved in various processes such as Wnt signaling pathway, telomere length and vesicle trafficking. Acts as an activator of the Wnt signaling pathway by mediating poly-ADP-ribosylation of AXIN1 and AXIN2, 2 key components of the beta-catenin destruction complex- poly-ADP- ribosylated target proteins are recognized by RNF146, which mediates their ubiquitination and subsequent degradation. Also mediates poly-ADP-ribosylation of BLZF1 and CASC3, followed by recruitment of RNF146 and subseque [...]	0.8
TOP2A	ENSP00000411532	topoisomerase (DNA) II alpha 170kDa	0.471
TOP2B	ENSP00000396704	topoisomerase (DNA) II beta 180kDa; Control of topological states of DNA by transient breakage and subsequent rejoining of DNA strands. Topoisomerase II makes double-strand breaks. Indirectly involved in vitamin D- coupled transcription regulation via its association with the WINAC complex, a chromatin-remodeling complex recruited by vitamin D receptor (VDR), which is required for the ligand-bound VDR- mediated transrepression of the CYP27B1 gene	0.471
TP53	ENSP00000269305	tumor protein p53; Acts as a tumor suppressor in many tumor types; induces growth arrest or apoptosis depending on the physiological circumstances and cell type. Involved in cell cycle regulation as a trans-activator that acts to negatively regulate cell division by controlling a set of genes required for this process. One of the activated genes is an inhibitor of cyclin-dependent kinases. Apoptosis induction seems to be mediated either by stimulation of BAX and FAS antigen expression, or by repression of Bcl-2 expression (By similarity)	0.868

	UGT1A1	ENSP00000304845	UDP glucuronosyltransferase 1 family, polypeptide A1; UDPGT is of major importance in the conjugation and subsequent elimination of potentially toxic xenobiotics and endogenous compounds. This isoform glucuronidates bilirubin IX- alpha to form both the IX-alpha-C8 and IX-alpha-C12 monoconjugates and diconjugate. Is also able to catalyze the glucuronidation of 17beta-estradiol, 17alpha-ethinylestradiol, 1-hydroxypyrene, 4- methylumbelliferone, 1-naphthol, paranitrophenol, scopoletin, and umbelliferone	0.938
	UGT1A10	ENSP00000343838	UDP glucuronosyltransferase 1 family, polypeptide A10; UDPGT is of major importance in the conjugation and subsequent elimination of potentially toxic xenobiotics and endogenous compounds	0.7
	UGT1A6	ENSP00000303174	UDP glucuronosyltransferase 1 family, polypeptide A6; UDPGT is of major importance in the conjugation and subsequent elimination of potentially toxic xenobiotics and endogenous compounds. This isoform has specificity for phenols	0.8
	UGT1A7	ENSP00000362525	UDP glucuronosyltransferase 1 family, polypeptide A7; UDPGT is of major importance in the conjugation and subsequent elimination of potentially toxic xenobiotics and endogenous compounds	0.7
	UGT1A8	ENSP00000362549	UDP glucuronosyltransferase 1 family, polypeptide A8; UDPGT is of major importance in the conjugation and subsequent elimination of potentially toxic xenobiotics and endogenous compounds	0.7
	UGT1A9	ENSP00000346768	UDP glucuronosyltransferase 1 family, polypeptide A9; UDPGT is of major importance in the conjugation and subsequent elimination of potentially toxic xenobiotics and endogenous compounds. This isoform has specificity for phenols	0.7
	UGT2B15	ENSP00000341045	UDP glucuronosyltransferase 2 family, polypeptide B15; UDPGTs are of major importance in the conjugation and subsequent elimination of potentially toxic xenobiotics and endogenous compounds. This isozyme displays activity toward several classes of xenobiotic substrates, including simple phenolic compounds, 7-hydroxylated coumarins, flavonoids, anthraquinones, and certain drugs and their hydroxylated metabolites. It also catalyzes the glucuronidation of endogenous estrogens and androgens	0.7
	VDR	ENSP00000447173	vitamin D (1,25- dihydroxyvitamin D3) receptor; Nuclear hormone receptor. Transcription factor that mediates the action of vitamin D3 by controlling the expression of hormone sensitive genes. Regulates transcription of hormone sensitive genes via its association with the WINAC complex, a chromatin-remodeling complex. Recruited to promoters via its interaction with the WINAC complex subunit BAZ1B/WSTF, which mediates the interaction with acetylated histones, an essential step for VDR-promoter association. Plays a central role in calcium homeostasis	0.8
	VEGFA	ENSP00000361125	vascular endothelial growth factor A	0.845
**node1**	**Baicalein**	**5281605**	**Baicalein (5,6,7-trihydroxyflavone) is a flavone, a type of flavonoid, originally isolated from the roots of "Scutellaria baicalensis" and "Scutellaria lateriflora". It is also reported in "Oroxylum indicum" or Indian trumpetflower. It is the aglycone of Baicalin. Baicalein is one of the active ingredients of Sho-Saiko-To, a Japanese herbal supplement believed to enhance liver health.**	
**node2**	ABCC1	ENSP00000382342	ATP-binding cassette, sub-family C (CFTR/MRP), member 1	0.7
	AKT1	ENSP00000270202	v-akt murine thymoma viral oncogene homolog 1; AKT1 is one of 3 closely related serine/threonine- protein kinases (AKT1, AKT2 and AKT3) called the AKT kinase, and which regulate many processes including metabolism, proliferation, cell survival, growth and angiogenesis. This is mediated through serine and/or threonine phosphorylation of a range of downstream substrates. Over 100 substrate candidates have been reported so far, but for most of them, no isoform specificity has been reported. AKT is responsible of the regulation of glucose uptake by mediating insulin-induced translocation o [...]	0.955
	ALOX12	ENSP00000251535	arachidonate 12-lipoxygenase; Oxygenase and 14,15-leukotriene A4 synthase activity	0.981
	ALOX15	ENSP00000293761	arachidonate 15-lipoxygenase; Converts arachidonic acid to 15S- hydroperoxyeicosatetraenoic acid. Also acts on C-12 of arachidonate as well as on Linoleic acid	0.935
	ALOX15B	ENSP00000369530	arachidonate 15-lipoxygenase, type B; Converts arachidonic acid exclusively to 15S- hydroperoxyeicosatetraenoic acid, while Linoleic acid is less well metabolized	0.498
	ALOX5	ENSP00000363512	arachidonate 5-lipoxygenase; Catalyzes the first step in leukotriene biosynthesis, and thereby plays a role in inflammatory processes	0.585
	CCNB1	ENSP00000256442	cyclin B1; Essential for the control of the cell cycle at the G2/M (mitosis) transition	0.8
	CCND1	ENSP00000227507	cyclin D1; Regulatory component of the cyclin D1-CDK4 (DC) complex that phosphorylates and inhibits members of the retinoblastoma (RB) protein family including RB1 and regulates the cell-cycle during G(1)/S transition. Phosphorylation of RB1 allows dissociation of the transcription factor E2F from the RB/E2F complex and the subsequent transcription of E2F target genes which are responsible for the progression through the G(1) phase. Hypophosphorylates RB1 in early G(1) phase. Cyclin D-CDK4 complexes are major integrators of various mitogenenic and antimitogenic signals. Also substrate [...]	0.839
	CDK1	ENSP00000378699	cyclin-dependent kinase 1; Plays a key role in the control of the eukaryotic cell cycle by modulating the centrosome cycle as well as mitotic onset; promotes G2-M transition, and regulates G1 progress and G1-S transition via association with multiple interphase cyclins. Required in higher cells for entry into S-phase and mitosis. Phosphorylates PARVA/actopaxin, APC, AMPH, APC, BARD1, Bcl- xL/BCL2L1, BRCA2, CALD1, CASP8, CDC7, CDC20, CDC25A, CDC25C, CC2D1A, CSNK2 proteins/CKII, FZR1/CDH1, CDK7, CEBPB, CHAMP1, DMD/dystrophin, EEF1 proteins/EF-1, EZH2, KIF11/EG5, EGFR, FANCG, FOS, GFAP, G [...]	0.842
	CDK2	ENSP00000266970	cyclin-dependent kinase 2; Serine/threonine-protein kinase involved in the control of the cell cycle; essential for meiosis, but dispensable for mitosis. Phosphorylates CTNNB1, USP37, p53/TP53, NPM1, CDK7, RB1, BRCA2, MYC, NPAT, EZH2. Interacts with cyclins A, B1, B3, D, or E. Triggers duplication of centrosomes and DNA. Acts at the G1-S transition to promote the E2F transcriptional program and the initiation of DNA synthesis, and modulates G2 progression; controls the timing of entry into mitosis/meiosis by controlling the subsequent activation of cyclin B/CDK1 by phosphorylation, and [...]	0.843
	CDK4	ENSP00000257904	cyclin-dependent kinase 4; Ser/Thr-kinase component of cyclin D-CDK4 (DC) complexes that phosphorylate and inhibit members of the retinoblastoma (RB) protein family including RB1 and regulate the cell-cycle during G(1)/S transition. Phosphorylation of RB1 allows dissociation of the transcription factor E2F from the RB/E2F complexes and the subsequent transcription of E2F target genes which are responsible for the progression through the G(1) phase. Hypophosphorylates RB1 in early G(1) phase. Cyclin D-CDK4 complexes are major integrators of various mitogenenic and antimitogenic signals. [...]	0.862
	CFTR	ENSP00000003084	cystic fibrosis transmembrane conductance regulator (ATP-binding cassette sub-family C, member 7); Involved in the transport of chloride ions. May regulate bicarbonate secretion and salvage in epithelial cells by regulating the SLC4A7 transporter. Can inhibit the chloride channel activity of ANO1	0.7
	CYP1A1	ENSP00000369050	cytochrome P450, family 1, subfamily A, polypeptide 1; Cytochromes P450 are a group of heme-thiolate monooxygenases. In liver microsomes, this enzyme is involved in an NADPH-dependent electron transport pathway. It oxidizes a variety of structurally unrelated compounds, including steroids, fatty acids, and xenobiotics	0.734
	CYP1A2	ENSP00000342007	cytochrome P450, family 1, subfamily A, polypeptide 2; Cytochromes P450 are a group of heme-thiolate monooxygenases. In liver microsomes, this enzyme is involved in an NADPH-dependent electron transport pathway. It oxidizes a variety of structurally unrelated compounds, including steroids, fatty acids, and xenobiotics. Most active in catalyzing 2-hydroxylation. Caffeine is metabolized primarily by cytochrome CYP1A2 in the liver through an initial N3-demethylation. Also acts in the metabolism of aflatoxin B1 and acetaminophen. Participates in the bioactivation of carcinogenic aromatic a [...]	0.938
	CYP1B1	ENSP00000260630	cytochrome P450, family 1, subfamily B, polypeptide 1; Cytochromes P450 are a group of heme-thiolate monooxygenases. In liver microsomes, this enzyme is involved in an NADPH-dependent electron transport pathway. It oxidizes a variety of structurally unrelated compounds, including steroids, fatty acids, and xenobiotics	0.758
	CYP3A4	ENSP00000337915	cytochrome P450, family 3, subfamily A, polypeptide 4; Cytochromes P450 are a group of heme-thiolate monooxygenases. In liver microsomes, this enzyme is involved in an NADPH-dependent electron transport pathway. It performs a variety of oxidation reactions (e.g. caffeine 8-oxidation, omeprazole sulphoxidation, midazolam 1'-hydroxylation and midazolam 4- hydroxylation) of structurally unrelated compounds, including steroids, fatty acids, and xenobiotics. Acts as a 1,8-cineole 2- exo-monooxygenase. The enzyme also hydroxylates etoposide	0.938
	DDIT4	ENSP00000307305	DNA-damage-inducible transcript 4; Regulates cell growth, proliferation and survival via inhibition of the activity of the mammalian target of rapamycin complex 1 (mTORC1). Inhibition of mTORC1 is mediated by a pathway that involves DDIT4/REDD1, AKT1, the TSC1-TSC2 complex and the GTPase RHEB. Plays an important role in responses to cellular energy levels and cellular stress, including responses to hypoxia and DNA damage. Regulates p53/TP53-mediated apoptosis in response to DNA damage via its effect on mTORC1 activity. Its role in the response to hypoxia depends on the cell type; it me [...]	0.8
	GSK3B	ENSP00000324806	glycogen synthase kinase 3 beta; Constitutively active protein kinase that acts as a negative regulator in the hormonal control of glucose homeostasis, Wnt signaling and regulation of transcription factors and microtubules, by phosphorylating and inactivating glycogen synthase (GYS1 or GYS2), EIF2B, CTNNB1/beta-catenin, APC, AXIN1, DPYSL2/CRMP2, JUN, NFATC1/NFATC, MAPT/TAU and MACF1. Requires primed phosphorylation of the majority of its substrates. In skeletal muscle, contributes to insulin regulation of glycogen synthesis by phosphorylating and inhibiting GYS1 activity and hence glyc [...]	0.729
	HSD11B2	ENSP00000316786	hydroxysteroid (11-beta) dehydrogenase 2	0.7
	IL6	ENSP00000258743	interleukin 6 (interferon, beta 2); Cytokine with a wide variety of biological functions. It is a potent inducer of the acute phase response. Plays an essential role in the final differentiation of B-cells into Ig- secreting cells Involved in lymphocyte and monocyte differentiation. It induces myeloma and plasmacytoma growth and induces nerve cells differentiation Acts on B-cells, T-cells, hepatocytes, hematopoietic progenitor cells and cells of the CNS. Also acts as a myokine. It is discharged into the bloodstream after muscle contraction and acts to increase the breakdown of fats and [...]	0.823
	INS	ENSP00000250971	insulin; Insulin decreases blood glucose concentration. It increases cell permeability to monosaccharides, amino acids and fatty acids. It accelerates glycolysis, the pentose phosphate cycle, and glycogen synthesis in liver (By similarity)	0.7
	KEAP1	ENSP00000171111	kelch-like ECH-associated protein 1; Acts as a substrate adapter protein for the E3 ubiquitin ligase complex formed by CUL3 and RBX1 and targets NFE2L2/NRF2 for ubiquitination and degradation by the proteasome, thus resulting in the suppression of its transcriptional activity and the repression of antioxidant response element-mediated detoxifying enzyme gene expression. Retains NFE2L2/NRF2 and may also retain BPTF in the cytosol. Targets PGAM5 for ubiquitination and degradation by the proteasome	0.8
	MAP2K1	ENSP00000302486	mitogen-activated protein kinase kinase 1; Dual specificity protein kinase which acts as an essential component of the MAP kinase signal transduction pathway. Binding of extracellular ligands such as growth factors, cytokines and hormones to their cell-surface receptors activates RAS and this initiates RAF1 activation. RAF1 then further activates the dual-specificity protein kinases MAP2K1/MEK1 and MAP2K2/MEK2. Both MAP2K1/MEK1 and MAP2K2/MEK2 function specifically in the MAPK/ERK cascade, and catalyze th concomitant phosphorylation of a threonine and a tyrosine residue in a Thr-Glu-T [...]	0.816
	MAPK1	ENSP00000215832	mitogen-activated protein kinase 1; Serine/threonine kinase which acts as an essential component of the MAP kinase signal transduction pathway. MAPK1/ERK2 and MAPK3/ERK1 are the 2 MAPKs which play an important role in the MAPK/ERK cascade. They participate also in a signaling cascade initiated by activated KIT and KITLG/SCF. Depending on the cellular context, the MAPK/ERK cascade mediates diverse biological functions such as cell growth, adhesion, survival and differentiation through the regulation of transcription, translation, cytoskeletal rearrangements. The MAPK/ERK cascade plays a [...]	0.938
	MAPK3	ENSP00000263025	mitogen-activated protein kinase 3; Serine/threonine kinase which acts as an essential component of the MAP kinase signal transduction pathway. MAPK1/ERK2 and MAPK3/ERK1 are the 2 MAPKs which play an important role in the MAPK/ERK cascade. They participate also in a signaling cascade initiated by activated KIT and KITLG/SCF. Depending on the cellular context, the MAPK/ERK cascade mediates diverse biological functions such as cell growth, adhesion, survival and differentiation through the regulation of transcription, translation, cytoskeletal rearrangements. The MAPK/ERK cascade plays a [...]	0.724

	MAPK8	ENSP00000353483	mitogen-activated protein kinase 8; Serine/threonine-protein kinase involved in various processes such as cell proliferation, differentiation, migration, transformation and programmed cell death. Extracellular stimuli such as proinflammatory cytokines or physical stress stimulate the stress-activated protein kinase/c-Jun N-terminal kinase (SAP/JNK) signaling pathway. In this cascade, two dual specificity kinases MAP2K4/MKK4 and MAP2K7/MKK7 phosphorylate and activate MAPK8/JNK1. In turn, MAPK8/JNK1 phosphorylates a number of transcription factors, primarily components of AP-1 such as JU [...]	0.733
	MCL1	ENSP00000358022	myeloid cell leukemia sequence 1 (BCL2-related)	0.8
	MITF	ENSP00000295600	microphthalmia-associated transcription factor	0.8
	MMP2	ENSP00000219070	matrix metallopeptidase 2 (gelatinase A, 72kDa gelatinase, 72kDa type IV collagenase); Ubiquitinous metalloproteinase that is involved in diverse functions such as remodeling of the vasculature, angiogenesis, tissue repair, tumor invasion, inflammation, and atherosclerotic plaque rupture. As well as degrading extracellular matrix proteins, can also act on several nonmatrix proteins such as big endothelial 1 and beta-type CGRP promoting vasoconstriction. Also cleaves KISS at a Gly-|-Leu bond. Appears to have a role in myocardial cell death pathways. Contributes to myocardial oxidative s [...]	0.944
	MMP9	ENSP00000361405	matrix metallopeptidase 9 (gelatinase B, 92kDa gelatinase, 92kDa type IV collagenase); May play an essential role in local proteolysis of the extracellular matrix and in leukocyte migration. Could play a role in bone osteoclastic resorption. Cleaves KiSS1 at a Gly-|-Leu bond. Cleaves type IV and type V collagen into large C-terminal three quarter fragments and shorter N-terminal one quarter fragments. Degrades fibronectin but not laminin or Pz-peptide	0.941
	MTRR	ENSP00000264668	5-methyltetrahydrofolate-homocysteine methyltransferase reductase; Involved in the reductive regeneration of cob(I)alamin cofactor required for the maintenance of methionine synthase in a functional state	0.473
	NFE2L2	ENSP00000380252	nuclear factor (erythroid-derived 2)-like 2; Transcription activator that binds to antioxidant response (ARE) elements in the promoter regions of target genes. Important for the coordinated up-regulation of genes in response to oxidative stress. May be involved in the transcriptional activation of genes of the beta-globin cluster by mediating enhancer activity of hypersensitive site 2 of the beta-globin locus control region	0.8
	NFKB1	ENSP00000226574	nuclear factor of kappa light polypeptide gene enhancer in B-cells 1; NF-kappa-B is a pleiotropic transcription factor present in almost all cell types and is the endpoint of a series of signal transduction events that are initiated by a vast array of stimuli related to many biological processes such as inflammation, immunity, differentiation, cell growth, tumorigenesis and apoptosis. NF-kappa-B is a homo- or heterodimeric complex formed by the Rel-like domain-containing proteins RELA/p65, RELB, NFKB1/p105, NFKB1/p50, REL and NFKB2/p52 and the heterodimeric p65-p50 complex appears to b [...]	0.7
	NFKBIB	ENSP00000312988	nuclear factor of kappa light polypeptide gene enhancer in B-cells inhibitor, beta; Inhibits NF-kappa-B by complexing with and trapping it in the cytoplasm. However, the unphosphorylated form resynthesized after cell stimulation is able to bind NF-kappa-B allowing its transport to the nucleus and protecting it to further NFKBIA- dependent inactivation. Association with inhibitor kappa B- interacting NKIRAS1 and NKIRAS2 prevent its phosphorylation rendering it more resistant to degradation, explaining its slower degradation	0.7
	NOS1	ENSP00000337459	nitric oxide synthase 1 (neuronal); Produces nitric oxide (NO) which is a messenger molecule with diverse functions throughout the body. In the brain and peripheral nervous system, NO displays many properties of a neurotransmitter. Probably has nitrosylase activity and mediates cysteine S-nitrosylation of cytoplasmic target proteins such SRR	0.786
	NOS2	ENSP00000327251	nitric oxide synthase 2, inducible	0.733
	NOS3	ENSP00000297494	nitric oxide synthase 3 (endothelial cell); Produces nitric oxide (NO) (By similarity)	0.631
	NOTCH1	ENSP00000277541	notch 1; Functions as a receptor for membrane-bound ligands Jagged1, Jagged2 and Delta1 to regulate cell-fate determination. Upon ligand activation through the released notch intracellular domain (NICD) it forms a transcriptional activator complex with RBPJ/RBPSUH and activates genes of the enhancer of split locus. Affects the implementation of differentiation, proliferation and apoptotic programs. May be important for normal lymphocyte function. In altered form, may contribute to transformation or progression in some T-cell neoplasms. Involved in the maturation of both CD4+ and CD8+ c [...]	0.7
	NR1I2	ENSP00000336528	nuclear receptor subfamily 1, group I, member 2	0.8
	PKM	ENSP00000320171	pyruvate kinase, muscle	0.7
	PLAU	ENSP00000361850	plasminogen activator, urokinase; Specifically cleaves the zymogen plasminogen to form the active enzyme plasmin	0.938
	POR	ENSP00000419970	P450 (cytochrome) oxidoreductase; This enzyme is required for electron transfer from NADP to cytochrome P450 in microsomes. It can also provide electron transfer to heme oxygenase and cytochrome B5	0.473
	PRKCA	ENSP00000408695	protein kinase C, alpha; Calcium-activated, phospholipid- and diacylglycerol (DAG)-dependent serine/threonine-protein kinase that is involved in positive and negative regulation of cell proliferation, apoptosis, differentiation, migration and adhesion, tumorigenesis, cardiac hypertrophy, angiogenesis, platelet function and inflammation, by directly phosphorylating targets such as RAF1, BCL2, CSPG4, TNNT2/CTNT, or activating signaling cascade involving MAPK1/3 (ERK1/2) and RAP1GAP. Involved in cell proliferation and cell growth arrest by positive and negative regulation of the cell cycl [...]	0.723
	PTGS1	ENSP00000354612	prostaglandin-endoperoxide synthase 1 (prostaglandin G/H synthase and cyclooxygenase); May play an important role in regulating or promoting cell proliferation in some normal and neoplastically transformed cells	0.817
	PTGS2	ENSP00000356438	prostaglandin-endoperoxide synthase 2 (prostaglandin G/H synthase and cyclooxygenase); Mediates the formation of prostaglandins from arachidonate. May have a role as a major mediator of inflammation and/or a role for prostanoid signaling in activity-dependent plasticity	0.824
	RB1	ENSP00000267163	retinoblastoma 1; Key regulator of entry into cell division that acts as a tumor suppressor. Promotes G0-G1 transition when phosphorylated by CDK3/cyclin-C. Acts as a transcription repressor of E2F1 target genes. The underphosphorylated, active form of RB1 interacts with E2F1 and represses its transcription activity, leading to cell cycle arrest. Directly involved in heterochromatin formation by maintaining overall chromatin structure and, in particular, that of constitutive heterochromatin by stabilizing histone methylation. Recruits and targets histone methyltransferases SUV39H1, SUV [...]	0.7
	RELA	ENSP00000384273	v-rel reticuloendotheliosis viral oncogene homolog A (avian); NF-kappa-B is a pleiotropic transcription factor present in almost all cell types and is the endpoint of a series of signal transduction events that are initiated by a vast array of stimuli related to many biological processes such as inflammation, immunity, differentiation, cell growth, tumorigenesis and apoptosis. NF-kappa-B is a homo- or heterodimeric complex formed by the Rel-like domain-containing proteins RELA/p65, RELB, NFKB1/p105, NFKB1/p50, REL and NFKB2/p52 and the heterodimeric p65-p50 complex appears to be most a [...]	0.7
	S100A7	ENSP00000357711	S100 calcium binding protein A7	0.8
	SERPINE1	ENSP00000223095	serpin peptidase inhibitor, clade E (nexin, plasminogen activator inhibitor type 1), member 1; Serine protease inhibitor. This inhibitor acts as 'bait' for tissue plasminogen activator, urokinase, protein C and matriptase-3/TMPRSS7. Its rapid interaction with PLAT may function as a major control point in the regulation of fibrinolysis	0.8
	TNFRSF10B	ENSP00000276431	tumor necrosis factor receptor superfamily, member 10b; Receptor for the cytotoxic ligand TNFSF10/TRAIL. The adapter molecule FADD recruits caspase-8 to the activated receptor. The resulting death-inducing signaling complex (DISC) performs caspase-8 proteolytic activation which initiates the subsequent cascade of caspases (aspartate-specific cysteine proteases) mediating apoptosis. Promotes the activation of NF- kappa-B. Essential for ER stress-induced apoptosis	0.8
	TP53	ENSP00000269305	tumor protein p53; Acts as a tumor suppressor in many tumor types; induces growth arrest or apoptosis depending on the physiological circumstances and cell type. Involved in cell cycle regulation as a trans-activator that acts to negatively regulate cell division by controlling a set of genes required for this process. One of the activated genes is an inhibitor of cyclin-dependent kinases. Apoptosis induction seems to be mediated either by stimulation of BAX and FAS antigen expression, or by repression of Bcl-2 expression (By similarity)	0.844
	UGT2B15	ENSP00000341045	UDP glucuronosyltransferase 2 family, polypeptide B15; UDPGTs are of major importance in the conjugation and subsequent elimination of potentially toxic xenobiotics and endogenous compounds. This isozyme displays activity toward several classes of xenobiotic substrates, including simple phenolic compounds, 7-hydroxylated coumarins, flavonoids, anthraquinones, and certain drugs and their hydroxylated metabolites. It also catalyzes the glucuronidation of endogenous estrogens and androgens	0.722
	VEGFA	ENSP00000361125	vascular endothelial growth factor A	0.839
**node1**	**Baicalin**	**64982**	**Baicalin is a flavone, a type of flavonoid. It is found in several species in the genus "Scutellaria", including "Scutellaria baicalensis" and "Scutellaria lateriflora". There are 10 mg/g Baicalin in "Scutellaria galericulata" leaves. Baicalin is the glucuronide of Baicalein, and it is one of the chemical ingredients of Sho-Saiko-To, an herbal supplement.**	
**node2**	ALOX5	ENSP00000363512	arachidonate 5-lipoxygenase; Catalyzes the first step in leukotriene biosynthesis, and thereby plays a role in inflammatory processes	0.818
	BECN1	ENSP00000355231	beclin 1, autophagy related; Plays a central role in autophagy. Required for the abcission step in cytokinesis. May play a role in antiviral host defense. Protects against infection by a neurovirulent strain of Sindbis virus	0.832
	CASP10	ENSP00000286186	caspase 10, apoptosis-related cysteine peptidase; Involved in the activation cascade of caspases responsible for apoptosis execution. Recruited to both Fas- and TNFR-1 receptors in a FADD dependent manner. May participate in the granzyme B apoptotic pathways. Cleaves and activates caspase- 3, -4, -6, -7, -8, and -9. Hydrolyzes the small- molecule substrates, Tyr-Val-Ala-Asp-|-AMC and Asp-Glu-Val-Asp-|-AMC	0.712
	CASP3	ENSP00000311032	caspase 3, apoptosis-related cysteine peptidase; Involved in the activation cascade of caspases responsible for apoptosis execution. At the onset of apoptosis it proteolytically cleaves poly(ADP-ribose) polymerase (PARP) at a '216-Asp-|-Gly-217' bond. Cleaves and activates sterol regulatory element binding proteins (SREBPs) between the basic helix-loop- helix leucine zipper domain and the membrane attachment domain. Cleaves and activates caspase-6, -7 and -9. Involved in the cleavage of huntingtin. Triggers cell adhesion in sympathetic neurons through RET cleavage	0.829
	CASP8	ENSP00000351273	caspase 8, apoptosis-related cysteine peptidase	0.784
	CDX1	ENSP00000231656	caudal type homeobox 1; Could play a role in the terminal differentiation of the intestine	0.8
	CDX2	ENSP00000370408	caudal type homeobox 2; Involved in the transcriptional regulation of multiple genes expressed in the intestinal epithelium. Important in broad range of functions from early differentiation to maintenance of the intestinal epithelial lining of both the small and large intestine	0.8
	CYP2E1	ENSP00000252945	cytochrome P450, family 2, subfamily E, polypeptide 1; Metabolizes several precarcinogens, drugs, and solvents to reactive metabolites. Inactivates a number of drugs and xenobiotics and also bioactivates many xenobiotic substrates to their hepatotoxic or carcinogenic forms	0.8
	CYP3A4	ENSP00000337915	cytochrome P450, family 3, subfamily A, polypeptide 4; Cytochromes P450 are a group of heme-thiolate monooxygenases. In liver microsomes, this enzyme is involved in an NADPH-dependent electron transport pathway. It performs a variety of oxidation reactions (e.g. caffeine 8-oxidation, omeprazole sulphoxidation, midazolam 1'-hydroxylation and midazolam 4- hydroxylation) of structurally unrelated compounds, including steroids, fatty acids, and xenobiotics. Acts as a 1,8-cineole 2- exo-monooxygenase. The enzyme also hydroxylates etoposide	0.8

	EGF	ENSP00000265171	epidermal growth factor; EGF stimulates the growth of various epidermal and epithelial tissues in vivo and in vitro and of some fibroblasts in cell culture. Magnesiotropic hormone that stimulates magnesium reabsorption in the renal distal convoluted tubule via engagement of EGFR and activation of the magnesium channel TRPM6	0.8
	FOXP3	ENSP00000365380	forkhead box P3; Probable transcription factor. Plays a critical role in the control of immune response	0.8
	HIF1A	ENSP00000338018	hypoxia inducible factor 1, alpha subunit (basic helix-loop-helix transcription factor); Functions as a master transcriptional regulator of the adaptive response to hypoxia. Under hypoxic conditions, activates the transcription of over 40 genes, including erythropoietin, glucose transporters, glycolytic enzymes, vascular endothelial growth factor, HILPDA, and other genes whose protein products increase oxygen delivery or facilitate metabolic adaptation to hypoxia. Plays an essential role in embryonic vascularization, tumor angiogenesis and pathophysiology of ischemic disease. Binds to [...]	0.8
	IL17A	ENSP00000344192	interleukin 17A; Induces stromal cells to produce proinflammatory and hematopoietic cytokines. Enhances the surface expression of ICAM1/intracellular adhesion molecule 1 in fibroblasts	0.8
	MTRR	ENSP00000264668	5-methyltetrahydrofolate-homocysteine methyltransferase reductase; Involved in the reductive regeneration of cob(I)alamin cofactor required for the maintenance of methionine synthase in a functional state	0.457
	MYC	ENSP00000367207	v-myc myelocytomatosis viral oncogene homolog (avian); Participates in the regulation of gene transcription. Binds DNA in a non-specific manner, yet also specifically recognizes the core sequence 5'-CAC[GA]TG-3'. Seems to activate the transcription of growth-related genes	0.8
	NOS1	ENSP00000337459	nitric oxide synthase 1 (neuronal); Produces nitric oxide (NO) which is a messenger molecule with diverse functions throughout the body. In the brain and peripheral nervous system, NO displays many properties of a neurotransmitter. Probably has nitrosylase activity and mediates cysteine S-nitrosylation of cytoplasmic target proteins such SRR	0.596
	NOS2	ENSP00000327251	nitric oxide synthase 2, inducible	0.733
	NOS3	ENSP00000297494	nitric oxide synthase 3 (endothelial cell); Produces nitric oxide (NO) (By similarity)	0.596
	NOTCH1	ENSP00000277541	notch 1; Functions as a receptor for membrane-bound ligands Jagged1, Jagged2 and Delta1 to regulate cell-fate determination. Upon ligand activation through the released notch intracellular domain (NICD) it forms a transcriptional activator complex with RBPJ/RBPSUH and activates genes of the enhancer of split locus. Affects the implementation of differentiation, proliferation and apoptotic programs. May be important for normal lymphocyte function. In altered form, may contribute to transformation or progression in some T-cell neoplasms. Involved in the maturation of both CD4+ and CD8+ c [...]	0.7
	NT5E	ENSP00000257770	5'-nucleotidase, ecto (CD73); Hydrolyzes extracellular nucleotides into membrane permeable nucleosides. Exhibits AMP-, NAD-, and NMN-nucleosidase activities	0.8
	PDGFB	ENSP00000330382	platelet-derived growth factor beta polypeptide; Growth factor that plays an essential role in the regulation of embryonic development, cell proliferation, cell migration, survival and chemotaxis. Potent mitogen for cells of mesenchymal origin. Required for normal proliferation and recruitment of pericytes and vascular smooth muscle cells in the central nervous system, skin, lung, heart and placenta. Required for normal blood vessel development, and for normal development of kidney glomeruli. Plays an important role in wound healing. Signaling is modulated by the formation of heterodim [...]	0.8
	PKM	ENSP00000320171	pyruvate kinase, muscle	0.7
	POR	ENSP00000419970	P450 (cytochrome) oxidoreductase; This enzyme is required for electron transfer from NADP to cytochrome P450 in microsomes. It can also provide electron transfer to heme oxygenase and cytochrome B5	0.457
	REN	ENSP00000272190	renin; Renin is a highly specific endopeptidase, whose only known function is to generate angiotensin I from angiotensinogen in the plasma, initiating a cascade of reactions that produce an elevation of blood pressure and increased sodium retention by the kidney	0.8
	TLR2	ENSP00000260010	toll-like receptor 2; Cooperates with LY96 to mediate the innate immune response to bacterial lipoproteins and other microbial cell wall components. Cooperates with TLR1 or TLR6 to mediate the innate immune response to bacterial lipoproteins or lipopeptides. Acts via MYD88 and TRAF6, leading to NF-kappa-B activation, cytokine secretion and the inflammatory response. May also promote apoptosis in response to lipoproteins. Recognizes mycoplasmal macrophage-activating lipopeptide-2kD (MALP-2), soluble tuberculosis factor (STF), phenol-soluble modulin (PSM) and B.burgdorferi outer surface [...]	0.816
	TLR4	ENSP00000363089	toll-like receptor 4	0.816
	TNF	ENSP00000398698	tumor necrosis factor	0.697
	VEGFA	ENSP00000361125	vascular endothelial growth factor A	0.815
**node1**	**Chrysin**	**5281607**	**Chrysin is a naturally occurring flavone, a type of flavonoid. It is found in the passion flowers "Passiflora caerulea" and in honeycomb.**	
**node2**	ABCB1	ENSP00000265724	ATP-binding cassette, sub-family B (MDR/TAP), member 1; Energy-dependent efflux pump responsible for decreased drug accumulation in multidrug-resistant cells	0.7
	ABCC2	ENSP00000359478	ATP-binding cassette, sub-family C (CFTR/MRP), member 2; Mediates hepatobiliary excretion of numerous organic anions. May function as a cellular cisplatin transporter	0.7
	ABCG2	ENSP00000237612	ATP-binding cassette, sub-family G (WHITE), member 2	0.779
	AKT1	ENSP00000270202	v-akt murine thymoma viral oncogene homolog 1; AKT1 is one of 3 closely related serine/threonine- protein kinases (AKT1, AKT2 and AKT3) called the AKT kinase, and which regulate many processes including metabolism, proliferation, cell survival, growth and angiogenesis. This is mediated through serine and/or threonine phosphorylation of a range of downstream substrates. Over 100 substrate candidates have been reported so far, but for most of them, no isoform specificity has been reported. AKT is responsible of the regulation of glucose uptake by mediating insulin-induced translocation o [...]	0.7
	BCL2	ENSP00000329623	B-cell CLL/lymphoma 2; Suppresses apoptosis in a variety of cell systems including factor-dependent lymphohematopoietic and neural cells. Regulates cell death by controlling the mitochondrial membrane permeability. Appears to function in a feedback loop system with caspases. Inhibits caspase activity either by preventing the release of cytochrome c from the mitochondria and/or by binding to the apoptosis-activating factor (APAF-1)	0.7
	CASP3	ENSP00000311032	caspase 3, apoptosis-related cysteine peptidase; Involved in the activation cascade of caspases responsible for apoptosis execution. At the onset of apoptosis it proteolytically cleaves poly(ADP-ribose) polymerase (PARP) at a '216-Asp-|-Gly-217' bond. Cleaves and activates sterol regulatory element binding proteins (SREBPs) between the basic helix-loop- helix leucine zipper domain and the membrane attachment domain. Cleaves and activates caspase-6, -7 and -9. Involved in the cleavage of huntingtin. Triggers cell adhesion in sympathetic neurons through RET cleavage	0.7
	CASP8	ENSP00000351273	caspase 8, apoptosis-related cysteine peptidase	0.7
	CASP9	ENSP00000330237	caspase 9, apoptosis-related cysteine peptidase	0.7
	CBR1	ENSP00000290349	carbonyl reductase 1; NADPH-dependent reductase with broad substrate specificity. Catalyzes the reduction of a wide variety of carbonyl compounds including quinones, prostaglandins, menadione, plus various xenobiotics. Catalyzes the reduction of the antitumor anthracyclines doxorubicin and daunorubicin to the cardiotoxic compounds doxorubicinol and daunorubicinol. Can convert prostaglandin E2 to prostaglandin F2-alpha. Can bind glutathione, which explains its higher affinity for glutathione-conjugated substrates. Catalyzes the reduction of S-nitrosoglutathione	0.409
	CYP19A1	ENSP00000260433	cytochrome P450, family 19, subfamily A, polypeptide 1; Catalyzes the formation of aromatic C18 estrogens from C19 androgens	0.931
	CYP1A1	ENSP00000369050	cytochrome P450, family 1, subfamily A, polypeptide 1; Cytochromes P450 are a group of heme-thiolate monooxygenases. In liver microsomes, this enzyme is involved in an NADPH-dependent electron transport pathway. It oxidizes a variety of structurally unrelated compounds, including steroids, fatty acids, and xenobiotics	0.841
	CYP1A2	ENSP00000342007	cytochrome P450, family 1, subfamily A, polypeptide 2; Cytochromes P450 are a group of heme-thiolate monooxygenases. In liver microsomes, this enzyme is involved in an NADPH-dependent electron transport pathway. It oxidizes a variety of structurally unrelated compounds, including steroids, fatty acids, and xenobiotics. Most active in catalyzing 2-hydroxylation. Caffeine is metabolized primarily by cytochrome CYP1A2 in the liver through an initial N3-demethylation. Also acts in the metabolism of aflatoxin B1 and acetaminophen. Participates in the bioactivation of carcinogenic aromatic a [...]	0.857
	CYP1B1	ENSP00000260630	cytochrome P450, family 1, subfamily B, polypeptide 1; Cytochromes P450 are a group of heme-thiolate monooxygenases. In liver microsomes, this enzyme is involved in an NADPH-dependent electron transport pathway. It oxidizes a variety of structurally unrelated compounds, including steroids, fatty acids, and xenobiotics	0.877
	MAPK1	ENSP00000215832	mitogen-activated protein kinase 1; Serine/threonine kinase which acts as an essential component of the MAP kinase signal transduction pathway. MAPK1/ERK2 and MAPK3/ERK1 are the 2 MAPKs which play an important role in the MAPK/ERK cascade. They participate also in a signaling cascade initiated by activated KIT and KITLG/SCF. Depending on the cellular context, the MAPK/ERK cascade mediates diverse biological functions such as cell growth, adhesion, survival and differentiation through the regulation of transcription, translation, cytoskeletal rearrangements. The MAPK/ERK cascade plays a [...]	0.7
	MAPK3	ENSP00000263025	mitogen-activated protein kinase 3; Serine/threonine kinase which acts as an essential component of the MAP kinase signal transduction pathway. MAPK1/ERK2 and MAPK3/ERK1 are the 2 MAPKs which play an important role in the MAPK/ERK cascade. They participate also in a signaling cascade initiated by activated KIT and KITLG/SCF. Depending on the cellular context, the MAPK/ERK cascade mediates diverse biological functions such as cell growth, adhesion, survival and differentiation through the regulation of transcription, translation, cytoskeletal rearrangements. The MAPK/ERK cascade plays a [...]	0.7
	PARP1	ENSP00000355759	poly (ADP-ribose) polymerase 1; Involved in the base excision repair (BER) pathway, by catalyzing the poly(ADP-ribosyl)ation of a limited number of acceptor proteins involved in chromatin architecture and in DNA metabolism. This modification follows DNA damages and appears as an obligatory step in a detection/signaling pathway leading to the reparation of DNA strand breaks. Mediates the poly(ADP- ribosyl)ation of APLF and CHFR. Positively regulates the transcription of MTUS1 and negatively regulates the transcription of MTUS2/TIP150. With EEF1A1 and TXK, forms a complex that acts as a [...]	0.7
	PGD	ENSP00000270776	phosphogluconate dehydrogenase; Catalyzes the oxidative decarboxylation of 6- phosphogluconate to ribulose 5-phosphate and CO(2), with concomitant reduction of NADP to NADPH (By similarity)	0.7
	PLCG1	ENSP00000244007	phospholipase C, gamma 1; Mediates the production of the second messenger molecules diacylglycerol (DAG) and inositol 1,4,5-trisphosphate (IP3). Plays an important role in the regulation of intracellular signaling cascades. Becomes activated in response to ligand- mediated activation of receptor-type tyrosine kinases, such as PDGFRA, PDGFRB, FGFR1, FGFR2, FGFR3 and FGFR4. Plays a role in actin reorganization and cell migration	0.7
	PYGB	ENSP00000216962	phosphorylase, glycogen; brain; Phosphorylase is an important allosteric enzyme in carbohydrate metabolism. Enzymes from different sources differ in their regulatory mechanisms and in their natural substrates. However, all known phosphorylases share catalytic and structural properties (By similarity)	0.712

	PYGL	ENSP00000216392	phosphorylase, glycogen, liver; Phosphorylase is an important allosteric enzyme in carbohydrate metabolism. Enzymes from different sources differ in their regulatory mechanisms and in their natural substrates. However, all known phosphorylases share catalytic and structural properties (By similarity)	0.712
	PYGM	ENSP00000164139	phosphorylase, glycogen, muscle; Phosphorylase is an important allosteric enzyme in carbohydrate metabolism. Enzymes from different sources differ in their regulatory mechanisms and in their natural substrates. However, all known phosphorylases share catalytic and structural properties (By similarity)	0.795
	RELA	ENSP00000384273	v-rel reticuloendotheliosis viral oncogene homolog A (avian); NF-kappa-B is a pleiotropic transcription factor present in almost all cell types and is the endpoint of a series of signal transduction events that are initiated by a vast array of stimuli related to many biological processes such as inflammation, immunity, differentiation, cell growth, tumorigenesis and apoptosis. NF-kappa-B is a homo- or heterodimeric complex formed by the Rel-like domain-containing proteins RELA/p65, RELB, NFKB1/p105, NFKB1/p50, REL and NFKB2/p52 and the heterodimeric p65-p50 complex appears to be most a [...]	0.7
	SULT1A1	ENSP00000321988	sulfotransferase family, cytosolic, 1A, phenol-preferring, member 1; Sulfotransferase that utilizes 3'-phospho-5'-adenylyl sulfate (PAPS) as sulfonate donor to catalyze the sulfate conjugation of catecholamines, phenolic drugs and neurotransmitters. Has also estrogen sulfotransferase activity. responsible for the sulfonation and activation of minoxidil. Is Mediates the metabolic activation of carcinogenic N- hydroxyarylamines to DNA binding products and could so participate as modulating factor of cancer risk	0.7
	SULT1E1	ENSP00000226444	sulfotransferase family 1E, estrogen-preferring, member 1; Sulfotransferase that utilizes 3'-phospho-5'-adenylyl sulfate (PAPS) as sulfonate donor to catalyze the sulfate conjugation of estradiol and estrone. May play a role in the regulation of estrogen receptor activity by metabolizing free estradiol. Maximally sulfates beta-estradiol and estrone at concentrations of 20 nM. Also sulfates dehydroepiandrosterone, pregnenolone, ethinylestradiol, equalenin, diethylstilbesterol and 1-naphthol, at significantly higher concentrations; however, cortisol, testosterone and dopamine are not sulfated	0.7
	TBK1	ENSP00000329967	TANK-binding kinase 1; Serine/threonine kinase that plays an essential role in regulating inflammatory responses to foreign agents. Following activation of toll-like receptors by viral or bacterial components, associates with TRAF3 and TANK and phosphorylates interferon regulatory factors (IRFs) IRF3 and IRF7 as well as DDX3X. This activity allows subsequent homodimerization and nuclear translocation of the IRFs leading to transcriptional activation of pro-inflammatory and antiviral genes including IFN- alpha and IFN-beta. In order to establish such an antiviral state, TBK1 form severa [...]	0.7
	TNFSF10	ENSP00000241261	tumor necrosis factor (ligand) superfamily, member 10; Cytokine that binds to TNFRSF10A/TRAILR1, TNFRSF10B/TRAILR2, TNFRSF10C/TRAILR3, TNFRSF10D/TRAILR4 and possibly also to TNFRSF11B/OPG. Induces apoptosis. Its activity may be modulated by binding to the decoy receptors TNFRSF10C/TRAILR3, TNFRSF10D/TRAILR4 and TNFRSF11B/OPG that cannot induce apoptosis	0.7
	TP53	ENSP00000269305	tumor protein p53; Acts as a tumor suppressor in many tumor types; induces growth arrest or apoptosis depending on the physiological circumstances and cell type. Involved in cell cycle regulation as a trans-activator that acts to negatively regulate cell division by controlling a set of genes required for this process. One of the activated genes is an inhibitor of cyclin-dependent kinases. Apoptosis induction seems to be mediated either by stimulation of BAX and FAS antigen expression, or by repression of Bcl-2 expression (By similarity)	0.722
	UGT1A1	ENSP00000304845	UDP glucuronosyltransferase 1 family, polypeptide A1; UDPGT is of major importance in the conjugation and subsequent elimination of potentially toxic xenobiotics and endogenous compounds. This isoform glucuronidates bilirubin IX- alpha to form both the IX-alpha-C8 and IX-alpha-C12 monoconjugates and diconjugate. Is also able to catalyze the glucuronidation of 17beta-estradiol, 17alpha-ethinylestradiol, 1-hydroxypyrene, 4- methylumbelliferone, 1-naphthol, paranitrophenol, scopoletin, and umbelliferone	0.7
	UGT1A10	ENSP00000343838	UDP glucuronosyltransferase 1 family, polypeptide A10; UDPGT is of major importance in the conjugation and subsequent elimination of potentially toxic xenobiotics and endogenous compounds	0.7
	UGT1A7	ENSP00000362525	UDP glucuronosyltransferase 1 family, polypeptide A7; UDPGT is of major importance in the conjugation and subsequent elimination of potentially toxic xenobiotics and endogenous compounds	0.7
	UGT1A8	ENSP00000362549	UDP glucuronosyltransferase 1 family, polypeptide A8; UDPGT is of major importance in the conjugation and subsequent elimination of potentially toxic xenobiotics and endogenous compounds	0.7
	UGT1A9	ENSP00000346768	UDP glucuronosyltransferase 1 family, polypeptide A9; UDPGT is of major importance in the conjugation and subsequent elimination of potentially toxic xenobiotics and endogenous compounds. This isoform has specificity for phenols	0.7
	UGT2B15	ENSP00000341045	UDP glucuronosyltransferase 2 family, polypeptide B15; UDPGTs are of major importance in the conjugation and subsequent elimination of potentially toxic xenobiotics and endogenous compounds. This isozyme displays activity toward several classes of xenobiotic substrates, including simple phenolic compounds, 7-hydroxylated coumarins, flavonoids, anthraquinones, and certain drugs and their hydroxylated metabolites. It also catalyzes the glucuronidation of endogenous estrogens and androgens	0.7
**node1**	**Coptisine**	**72321**	**Coptisine is an alkaloid found in Chinese goldthread ("Coptis chinensis"). Famous for the bitter taste that it produces, it is used in Chinese herbal medicine along with the related compound berberine for treating digestive disorders caused by bacterial infections.**	
**node2**	ESF1	ENSP00000202816	ESF1, nucleolar pre-rRNA processing protein, homolog (S. cerevisiae); May constitute a novel regulatory system for basal transcription. Negatively regulates ABT1 (By similarity)	0.414
	PAWR	ENSP00000328088	PRKC, apoptosis, WT1, regulator; Pro-apoptotic protein capable of selectively inducing apoptosis in cancer cells, sensitizing the cells to diverse apoptotic stimuli and causing regression of tumors in animal models. Induces apoptosis in certain cancer cells by activation of the Fas prodeath pathway and coparallel inhibition of NF-kappa-B transcriptional activity. Inhibits the transcriptional activation and augments the transcriptional repression mediated by WT1. Down- regulates the anti-apoptotic protein BCL2 via its interaction with WT1. Seems also to be a transcriptional repressor by [...]	0.501
	TNFSF11	ENSP00000239849	tumor necrosis factor (ligand) superfamily, member 11; Cytokine that binds to TNFRSF11B/OPG and to TNFRSF11A/RANK. Osteoclast differentiation and activation factor. Augments the ability of dendritic cells to stimulate naive T-cell proliferation. May be an important regulator of interactions between T-cells and dendritic cells and may play a role in the regulation of the T-cell-dependent immune response. May also play an important role in enhanced bone-resorption in humoral hypercalcemia of malignancy	0.8
	XPO1	ENSP00000384863	exportin 1 (CRM1 homolog, yeast); Mediates the nuclear export of cellular proteins (cargos) bearing a leucine-rich nuclear export signal (NES) and of RNAs. In the nucleus, in association with RANBP3, binds cooperatively to the NES on its target protein and to the GTPase RAN in its active GTP-bound form (Ran-GTP). Docking of this complex to the nuclear pore complex (NPC) is mediated through binding to nucleoporins. Upon transit of a nuclear export complex into the cytoplasm, disassembling of the complex and hydrolysis of Ran-GTP to Ran-GDP (induced by RANBP1 and RANGAP1, respectively) c [...]	0.457
**node1**	**Erucic acid**	**5281116**	**Erucic acid**	
**node2**	ALDOA	ENSP00000336927	aldolase A, fructose-bisphosphate; Plays a key role in glycolysis and gluconeogenesis. In addition, may also function as scaffolding protein (By similarity)	0.589
**node1**	**Linoleic acid**	**5280450**	**Linoleic acid**	
**node2**	ABCA1	ENSP00000363868	ATP-binding cassette, sub-family A (ABC1), member 1	0.92
	ABCB4	ENSP00000265723	ATP-binding cassette, sub-family B (MDR/TAP), member 4; Mediates ATP-dependent export of organic anions and drugs from the cytoplasm. Hydrolyzes ATP with low efficiency. Not capable of conferring drug resistance. Mediates the translocation of phosphatidylcholine across the canalicular membrane of the hepatocyte	0.9
	ACADM	ENSP00000409612	acyl-CoA dehydrogenase, C-4 to C-12 straight chain; This enzyme is specific for acyl chain lengths of 4 to 16	0.9
	ACOT1	ENSP00000311224	acyl-CoA thioesterase 1; Acyl-CoA thioesterases are a group of enzymes that catalyze the hydrolysis of acyl-CoAs to the free fatty acid and coenzyme A (CoASH), providing the potential to regulate intracellular levels of acyl-CoAs, free fatty acids and CoASH. Active towards fatty acyl-CoA with chain-lengths of C12-C16 (By similarity)	0.8
	ACOT2	ENSP00000238651	acyl-CoA thioesterase 2; Acyl-CoA thioesterases are a group of enzymes that catalyze the hydrolysis of acyl-CoAs to the free fatty acid and coenzyme A (CoASH), providing the potential to regulate intracellular levels of acyl-CoAs, free fatty acids and CoASH. Displays high levels of activity on medium- and long chain acyl CoAs	0.8
	ACOT4	ENSP00000323071	acyl-CoA thioesterase 4; Acyl-CoA thioesterases are a group of enzymes that catalyze the hydrolysis of acyl-CoAs to the free fatty acid and coenzyme A (CoASH), providing the potential to regulate intracellular levels of acyl-CoAs, free fatty acids and CoASH (By similarity). Succinyl-CoA thioesterase that also hydrolyzes long chain saturated and unsaturated monocarboxylic acyl-CoAs	0.8
	ACOT7	ENSP00000367086	acyl-CoA thioesterase 7	0.8
	ACSBG1	ENSP00000258873	acyl-CoA synthetase bubblegum family member 1; Mediates activation of long-chain fatty acids for both synthesis of cellular lipids, and degradation via beta-oxidation. Able to activate long-chain fatty acids. Also able to activate very long-chain fatty acids; however, the relevance of such activity is unclear in vivo. Can activate diverse saturated, monosaturated and polyunsaturated fatty acids	0.9
	ACSBG2	ENSP00000252669	acyl-CoA synthetase bubblegum family member 2; Mediates activation of long-chain fatty acids for both synthesis of cellular lipids, and degradation via beta-oxidation. Able to activate long-chain fatty acids. Also able to activate very long-chain fatty acids; however, the relevance of such activity is unclear in vivo. Has increased ability to activate oleic and Linoleic acid. May play a role in spermatogenesis	0.9
	ACSL1	ENSP00000281455	acyl-CoA synthetase long-chain family member 1; Activation of long-chain fatty acids for both synthesis of cellular lipids, and degradation via beta-oxidation. Preferentially uses palmitoleate, oleate and linoleate	0.912
	ACSL3	ENSP00000350012	acyl-CoA synthetase long-chain family member 3; Acyl-CoA synthetases (ACSL) activates long-chain fatty acids for both synthesis of cellular lipids, and degradation via beta-oxidation. ACSL3 mediates hepatic lipogenesis (By similarity). Preferentially uses myristate, laurate, arachidonate and eicosapentaenoate as substrates (By similarity). Has mainly an anabolic role in energy metabolism. Required for the incorporation of fatty acids into phosphatidylcholine, the major phospholipid located on the surface of VLDL (very low density lipoproteins)	0.9
	ACSL4	ENSP00000339787	acyl-CoA synthetase long-chain family member 4; Activation of long-chain fatty acids for both synthesis of cellular lipids, and degradation via beta-oxidation. Preferentially uses arachidonate and eicosapentaenoate as substrates	0.979
	ACSL5	ENSP00000348429	acyl-CoA synthetase long-chain family member 5; Acyl-CoA synthetases (ACSL) activate long-chain fatty acids for both synthesis of cellular lipids, and degradation via beta-oxidation. ACSL5 may activate fatty acids from exogenous sources for the synthesis of triacylglycerol destined for intracellular storage (By similarity). Utilizes a wide range of saturated fatty acids with a preference for C16-C18 unsaturated fatty acids (By similarity). It was suggested that it may also stimulate fatty acid oxidation (By similarity). At the villus tip of the crypt-villus axis of the small intestine [...]	0.9
	ACSL6	ENSP00000296869	acyl-CoA synthetase long-chain family member 6; Activation of long-chain fatty acids for both synthesis of cellular lipids, and degradation via beta-oxidation. Plays an important role in fatty acid metabolism in brain and the acyl-CoAs produced may be utilized exclusively for the synthesis of the brain lipid	0.9
	ACSM1	ENSP00000301956	acyl-CoA synthetase medium-chain family member 1; Has medium-chain fatty acid-CoA ligase activity with broad substrate specificity (in vitro). Acts on acids from C(4) to C(11) and on the corresponding 3-hydroxy- and 2,3- or 3,4- unsaturated acids (in vitro). Functions as GTP-dependent lipoate- activating enzyme that generates the substrate for lipoyltransferase (By similarity)	0.9
	ACSM3	ENSP00000289416	acyl-CoA synthetase medium-chain family member 3; Has medium-chain fatty acid-CoA ligase activity with broad substrate specificity (in vitro). Acts on acids from C(4) to C(11) and on the corresponding 3-hydroxy- and 2,3- or 3,4- unsaturated acids (in vitro) (By similarity)	0.9
	ACSM4	ENSP00000382349	acyl-CoA synthetase medium-chain family member 4; Has medium-chain fatty acid-CoA ligase activity with broad substrate specificity (in vitro). Acts on acids from C(4) to C(11) and on the corresponding 3-hydroxy- and 2,3- or 3,4- unsaturated acids (in vitro) (By similarity)	0.9

ACSM5	ENSP00000327916	acyl-CoA synthetase medium-chain family member 5; Has medium-chain fatty acid-CoA ligase activity with broad substrate specificity (in vitro). Acts on acids from C(4) to C(11) and on the corresponding 3-hydroxy- and 2,3- or 3,4- unsaturated acids (in vitro) (By similarity)	0.9
AGRN	ENSP00000368678	agrin; Agrin N-terminal 110 kDa subunit- is involved in regulation of neurite outgrowth probably due to the presence of the glycosaminoglcan (GAG) side chains of heparan and chondroitin sulfate attached to the Ser/Thr- and Gly/Ser-rich regions. Also involved in modulation of growth factor signaling (By similarity)	0.9
AGT	ENSP00000355627	angiotensinogen (serpin peptidase inhibitor, clade A, member 8); Essential component of the renin-angiotensin system (RAS), a potent regulator of blood pressure, body fluid and electrolyte homeostasis	0.907
ALAS1	ENSP00000309259	aminolevulinate, delta-, synthase 1	0.9
ALB	ENSP00000295897	albumin	0.475
ALOX12	ENSP00000251535	arachidonate 12-lipoxygenase; Oxygenase and 14,15-leukotriene A4 synthase activity	0.657
ALOX15	ENSP00000293761	arachidonate 15-lipoxygenase; Converts arachidonic acid to 15S- hydroperoxyeicosatetraenoic acid. Also acts on C-12 of arachidonate as well as on Linoleic acid	0.977
ALOX15B	ENSP00000369530	arachidonate 15-lipoxygenase, type B; Converts arachidonic acid exclusively to 15S- hydroperoxyeicosatetraenoic acid, while Linoleic acid is less well metabolized	0.96
ALOX5	ENSP00000363512	arachidonate 5-lipoxygenase; Catalyzes the first step in leukotriene biosynthesis, and thereby plays a role in inflammatory processes	0.885
ANGPTL4	ENSP00000301455	angiopoietin-like 4; Protein with hypoxia-induced expression in endothelial cells. May act as a regulator of angiogenesis and modulate tumorigenesis. Inhibits proliferation, migration, and tubule formation of endothelial cells and reduces vascular leakage. May exert a protective function on endothelial cells through an endocrine action. It is directly involved in regulating glucose homeostasis, lipid metabolism, and insulin sensitivity. In response to hypoxia, the unprocessed form of the protein accumulates in the subendothelial extracellular matrix (ECM). The matrix-associated and imm [...]	0.981
ANKRD1	ENSP00000360762	ankyrin repeat domain 1 (cardiac muscle); May play an important role in endothelial cell activation. May act as a nuclear transcription factor that negatively regulates the expression of cardiac genes. Induction seems to be correlated with apoptotic cell death in hepatoma cells	0.9
APOA1	ENSP00000236850	apolipoprotein A-I; Participates in the reverse transport of cholesterol from tissues to the liver for excretion by promoting cholesterol efflux from tissues and by acting as a cofactor for the lecithin cholesterol acyltransferase (LCAT). As part of the SPAP complex, activates spermatozoa motility	0.981
APOA2	ENSP00000356969	apolipoprotein A-II; May stabilize HDL (high density lipoprotein) structure by its association with lipids, and affect the HDL metabolism	0.9
APOA4	ENSP00000350425	apolipoprotein A-IV; May have a role in chylomicrons and VLDL secretion and catabolism. Required for efficient activation of lipoprotein lipase by ApoC-II; potent activator of LCAT. Apoa-IV is a major component of HDL and chylomicrons	0.9
APOA5	ENSP00000227665	apolipoprotein A-V; Minor apolipoprotein mainly associated with HDL and to a lesser extent with VLDL. May also be associated with chylomicrons. Important determinant of plasma triglyceride (TG) levels by both being a potent stimulator of apo-CII lipoprotein lipase (LPL) TG hydrolysis and a inhibitor of the hepatic VLDL-TG production rate (without affecting the VLDL-apoB production rate) (By similarity). Activates poorly lecithin-cholesterol acyltransferase (LCAT) and does not enhance efflux of cholesterol from macrophages	0.9
APOB	ENSP00000233242	apolipoprotein B (including Ag(x) antigen)	0.943
APOC3	ENSP00000227667	apolipoprotein C-III; Inhibits lipoprotein lipase and hepatic lipase and decreases the uptake of lymph chylomicrons by hepatic cells. This suggests that it delays the catabolism of triglyceride-rich particles	0.908
APOE	ENSP00000252486	apolipoprotein E; Mediates the binding, internalization, and catabolism of lipoprotein particles. It can serve as a ligand for the LDL (apo B/E) receptor and for the specific apo-E receptor (chylomicron remnant) of hepatic tissues	0.916
ARNTL	ENSP00000374357	aryl hydrocarbon receptor nuclear translocator-like	0.9
BAAT	ENSP00000259407	bile acid CoA- amino acid N-acyltransferase (glycine N-choloyltransferase); Involved in bile acid metabolism. In liver hepatocytes catalyzes the second step in the conjugation of C24 bile acids (choloneates) to glycine and taurine before excretion into bile canaliculi. The major components of bile are cholic acid and chenodeoxycholic acid. In a first step the bile acids are converted to an acyl-CoA thioester, either in peroxisomes (primary bile acids deriving from the cholesterol pathway), or cytoplasmic at the endoplasmic reticulum (secondary bile acids). May catalyze the conjugation [...]	0.8
C10orf129	ENSP00000340296	chromosome 10 open reading frame 129	0.9
C2	ENSP00000299367	complement component 2	0.563
CARM1	ENSP00000325690	coactivator-associated arginine methyltransferase 1; Methylates (mono- and asymmetric dimethylation) the guanidino nitrogens of arginyl residues in several proteins involved in DNA packaging, transcription regulation, pre-mRNA splicing, and mRNA stability. Recruited to promoters upon gene activation together with histone acetyltransferases from EP300/P300 and p160 families, methylates histone H3 at 'Arg-17' (H3R17me), forming mainly asymmetric dimethylarginine (H3R17me2a), leading to activate transcription via chromatin remodeling. During nuclear hormone receptor activation and TCF7L2/ [...]	0.9
CAT	ENSP00000241052	catalase; Occurs in almost all aerobically respiring organisms and serves to protect cells from the toxic effects of hydrogen peroxide. Promotes growth of cells including T-cells, B-cells, myeloid leukemia cells, melanoma cells, mastocytoma cells and normal and transformed fibroblast cells	0.904
CCNC	ENSP00000428982	cyclin C; Component of the Mediator complex, a coactivator involved in regulated gene transcription of nearly all RNA polymerase II-dependent genes. Mediator functions as a bridge to convey information from gene-specific regulatory proteins to the basal RNA polymerase II transcription machinery. Mediator is recruited to promoters by direct interactions with regulatory proteins and serves as a scaffold for the assembly of a functional preinitiation complex with RNA polymerase II and the general transcription factors. Binds to and activates cyclin-dependent kinase CDK8 that phosphorylate [...]	0.9
CD36	ENSP00000308165	CD36 molecule (thrombospondin receptor)	0.94
CDK19	ENSP00000357907	cyclin-dependent kinase 19	0.9
CDK8	ENSP00000370938	cyclin-dependent kinase 8; Component of the Mediator complex, a coactivator involved in regulated gene transcription of nearly all RNA polymerase II-dependent genes. Mediator functions as a bridge to convey information from gene-specific regulatory proteins to the basal RNA polymerase II transcription machinery. Mediator is recruited to promoters by direct interactions with regulatory proteins and serves as a scaffold for the assembly of a functional preinitiation complex with RNA polymerase II and the general transcription factors. Phosphorylates the CTD (C-terminal domain) of the lar [...]	0.9
CETP	ENSP00000200676	cholesteryl ester transfer protein, plasma; Involved in the transfer of insoluble cholesteryl esters in the reverse transport of cholesterol	0.843
CFB	ENSP00000416561	complement factor B	0.563
CHD9	ENSP00000455307	chromodomain helicase DNA binding protein 9; Acts as a transcriptional coactivator for PPARA and possibly other nuclear receptors. Proposed to be a ATP-dependent chromatin remodeling protein. Has DNA-dependent ATPase activity and binds to A/T-rich DNA. Associates with A/T-rich regulatory regions in promoters of genes that participate in the differentiation of progenitors during osteogenesis (By similarity)	0.9
CLOCK	ENSP00000308741	clock homolog (mouse); ARNTL/2-CLOCK heterodimers activate E-box element (5'- CACGTG-3') transcription of a number of proteins of the circadian clock. Activates transcription of PER1 and PER2. This transcription is inhibited in a feedback loop by PER and CRY proteins. Has intrinsic histone acetyltransferase activity and this enzymatic function contributes to chromatin-remodeling events implicated in circadian control of gene expression (By similarity). Acetylates primarily histones H3 and H4 (By similarity). Acetylates also a non-histone substrate- ARNTL (By similarity). Plays a role i [...]	0.9
CPT1A	ENSP00000265641	carnitine palmitoyltransferase 1A (liver); Catalyzes the transfer of the acyl group of long-chain fatty acid-CoA conjugates onto carnitine, an essential step for the mitochondrial uptake of long-chain fatty acids and their subsequent beta-oxidation in the mitochondrion. Plays an important role in triglyceride metabolism	0.948
CPT2	ENSP00000360541	carnitine palmitoyltransferase 2	0.933
CREBBP	ENSP00000262367	CREB binding protein; Acetylates histones, giving a specific tag for transcriptional activation. Also acetylates non-histone proteins, like NCOA3 and FOXO1. Binds specifically to phosphorylated CREB and enhances its transcriptional activity toward cAMP-responsive genes. Acts as a coactivator of ALX1 in the presence of EP300	0.907
CTGF	ENSP00000356954	connective tissue growth factor; Major connective tissue mitoattractant secreted by vascular endothelial cells. Promotes proliferation and differentiation of chondrocytes. Mediates heparin- and divalent cation-dependent cell adhesion in many cell types including fibroblasts, myofibroblasts, endothelial and epithelial cells. Enhances fibroblast growth factor-induced DNA synthesis	0.9
CYP1A1	ENSP00000369050	cytochrome P450, family 1, subfamily A, polypeptide 1; Cytochromes P450 are a group of heme-thiolate monooxygenases. In liver microsomes, this enzyme is involved in an NADPH-dependent electron transport pathway. It oxidizes a variety of structurally unrelated compounds, including steroids, fatty acids, and xenobiotics	0.907
CYP1A2	ENSP00000342007	cytochrome P450, family 1, subfamily A, polypeptide 2; Cytochromes P450 are a group of heme-thiolate monooxygenases. In liver microsomes, this enzyme is involved in an NADPH-dependent electron transport pathway. It oxidizes a variety of structurally unrelated compounds, including steroids, fatty acids, and xenobiotics. Most active in catalyzing 2-hydroxylation. Caffeine is metabolized primarily by cytochrome CYP1A2 in the liver through an initial N3-demethylation. Also acts in the metabolism of aflatoxin B1 and acetaminophen. Participates in the bioactivation of carcinogenic aromatic a [...]	0.9
CYP1B1	ENSP00000260630	cytochrome P450, family 1, subfamily B, polypeptide 1; Cytochromes P450 are a group of heme-thiolate monooxygenases. In liver microsomes, this enzyme is involved in an NADPH-dependent electron transport pathway. It oxidizes a variety of structurally unrelated compounds, including steroids, fatty acids, and xenobiotics	0.817
CYP2A13	ENSP00000332679	cytochrome P450, family 2, subfamily A, polypeptide 13; Exhibits a coumarin 7-hydroxylase activity. Active in the metabolic activation of hexamethylphosphoramide, N,N- dimethylaniline, 2'-methoxyacetophenone, N- nitrosomethylphenylamine, and the tobacco-specific carcinogen, 4- (methylnitrosamino)-1-(3-pyridyl)-1-butanone. Possesses phenacetin O-deethylation activity	0.8
CYP2A7	ENSP00000301146	cytochrome P450, family 2, subfamily A, polypeptide 7; Cytochromes P450 are a group of heme-thiolate monooxygenases. In liver microsomes, this enzyme is involved in an NADPH-dependent electron transport pathway. It oxidizes a variety of structurally unrelated compounds, including steroids, fatty acids, and xenobiotics	0.8

CYP2C18	ENSP00000285979	cytochrome P450, family 2, subfamily C, polypeptide 18; Cytochromes P450 are a group of heme-thiolate monooxygenases. In liver microsomes, this enzyme is involved in an NADPH-dependent electron transport pathway. It oxidizes a variety of structurally unrelated compounds, including steroids, fatty acids, and xenobiotics	0.8
CYP2C19	ENSP00000360372	cytochrome P450, family 2, subfamily C, polypeptide 19; Responsible for the metabolism of a number of therapeutic agents such as the anticonvulsant drug S-mephenytoin, omeprazole, proguanil, certain barbiturates, diazepam, propranolol, citalopram and imipramine	0.9
CYP2C8	ENSP00000360317	cytochrome P450, family 2, subfamily C, polypeptide 8; Cytochromes P450 are a group of heme-thiolate monooxygenases. In liver microsomes, this enzyme is involved in an NADPH-dependent electron transport pathway. It oxidizes a variety of structurally unrelated compounds, including steroids, fatty acids, and xenobiotics. In the epoxidation of arachidonic acid it generates only 14,15- and 11,12-cis-epoxyeicosatrienoic acids. It is the principal enzyme responsible for the metabolism the anti- cancer drug paclitaxel (taxol)	0.9
CYP2C9	ENSP00000260682	cytochrome P450, family 2, subfamily C, polypeptide 9; Cytochromes P450 are a group of heme-thiolate monooxygenases. In liver microsomes, this enzyme is involved in an NADPH-dependent electron transport pathway. It oxidizes a variety of structurally unrelated compounds, including steroids, fatty acids, and xenobiotics. This enzyme contributes to the wide pharmacokinetics variability of the metabolism of drugs such as S- warfarin, diclofenac, phenytoin, tolbutamide and losartan	0.9
CYP2D6	ENSP00000353820	cytochrome P450, family 2, subfamily D, polypeptide 6; Responsible for the metabolism of many drugs and environmental chemicals that it oxidizes. It is involved in the metabolism of drugs such as antiarrhythmics, adrenoceptor antagonists, and tricyclic antidepressants	0.8
CYP2E1	ENSP00000252945	cytochrome P450, family 2, subfamily E, polypeptide 1; Metabolizes several precarcinogens, drugs, and solvents to reactive metabolites. Inactivates a number of drugs and xenobiotics and also bioactivates many xenobiotic substrates to their hepatotoxic or carcinogenic forms	0.9
CYP2F1	ENSP00000333534	cytochrome P450, family 2, subfamily F, polypeptide 1; May be involved in the metabolism of various pneumotoxicants including naphthalene. Is able to dealkylate ethoxycoumarin, propoxycoumarin, and pentoxyresorufin but possesses no activity toward ethoxyresorufin and only trace dearylation activity toward benzyloxyresorufin. Bioactivates 3- methylindole (3MI) by dehydrogenation to the putative electrophile 3-methylene-indolenine	0.8
CYP2J2	ENSP00000360247	cytochrome P450, family 2, subfamily J, polypeptide 2; This enzyme metabolizes arachidonic acid predominantly via a NADPH-dependent olefin epoxidation to all four regioisomeric cis-epoxyeicosatrienoic acids. One of the predominant enzymes responsible for the epoxidation of endogenous cardiac arachidonic acid pools	0.916
CYP2S1	ENSP00000308032	cytochrome P450, family 2, subfamily S, polypeptide 1; Has a potential importance for extrahepatic xenobiotic metabolism	0.8
CYP3A4	ENSP00000337915	cytochrome P450, family 3, subfamily A, polypeptide 4; Cytochromes P450 are a group of heme-thiolate monooxygenases. In liver microsomes, this enzyme is involved in an NADPH-dependent electron transport pathway. It performs a variety of oxidation reactions (e.g. caffeine 8-oxidation, omeprazole sulphoxidation, midazolam 1'-hydroxylation and midazolam 4- hydroxylation) of structurally unrelated compounds, including steroids, fatty acids, and xenobiotics. Acts as a 1,8-cineole 2- exo-monooxygenase. The enzyme also hydroxylates etoposide	0.9
CYP3A5	ENSP00000222982	cytochrome P450, family 3, subfamily A, polypeptide 5; Cytochromes P450 are a group of heme-thiolate monooxygenases. In liver microsomes, this enzyme is involved in an NADPH-dependent electron transport pathway. It oxidizes a variety of structurally unrelated compounds, including steroids, fatty acids, and xenobiotics	0.8
CYP3A7	ENSP00000337450	cytochrome P450, family 3, subfamily A, polypeptide 7; Cytochromes P450 are a group of heme-thiolate monooxygenases. In liver microsomes, this enzyme is involved in an NADPH-dependent electron transport pathway. It oxidizes a variety of structurally unrelated compounds, including steroids, fatty acids, and xenobiotics	0.8
CYP4A11	ENSP00000311095	cytochrome P450, family 4, subfamily A, polypeptide 11; Catalyzes the omega- and (omega-1)-hydroxylation of various fatty acids such as laurate, myristate and palmitate. Has little activity toward prostaglandins A1 and E1. Oxidizes arachidonic acid to 20-hydroxyeicosatetraenoic acid (20-HETE)	0.908
CYP4B1	ENSP00000360991	cytochrome P450, family 4, subfamily B, polypeptide 1; Cytochromes P450 are a group of heme-thiolate monooxygenases. In liver microsomes, this enzyme is involved in an NADPH-dependent electron transport pathway. It oxidizes a variety of structurally unrelated compounds, including steroids, fatty acids, and xenobiotics	0.8
CYP4F11	ENSP00000248041	cytochrome P450, family 4, subfamily F, polypeptide 11	0.8
CYP4F12	ENSP00000321821	cytochrome P450, family 4, subfamily F, polypeptide 12; Catalyzes leukotriene B4 omega-hydroxylation and arachidonic acid omega-hydroxylation but with an activity much lower than that of CYP4F2. Catalyzes the hydroxylation of the antihistamine ebastine	0.8
CYP4X1	ENSP00000360968	cytochrome P450, family 4, subfamily X, polypeptide 1	0.8
CYP4Z1	ENSP00000334246	cytochrome P450, family 4, subfamily Z, polypeptide 1	0.8
CYP7A1	ENSP00000301645	cytochrome P450, family 7, subfamily A, polypeptide 1; Catalyzes a rate-limiting step in cholesterol catabolism and bile acid biosynthesis by introducing a hydrophilic moiety at position 7 of cholesterol. Important for cholesterol homeostasis	0.933
ELANE	ENSP00000263621	elastase, neutrophil expressed; Modifies the functions of natural killer cells, monocytes and granulocytes. Inhibits C5a-dependent neutrophil enzyme release and chemotaxis	0.403
ENSG00000168970	ENSP00000371886	JMJD7-PLA2G4B readthrough	0.9
ENSG00000244255	ENSP00000410815	Complement factor B Ba fragment; Uncharacterized protein; cDNA FLJ55673, highly similar to Complement factor B	0.563
EP300	ENSP00000263253	E1A binding protein p300; Functions as histone acetyltransferase and regulates transcription via chromatin remodeling. Acetylates all four core histones in nucleosomes. Histone acetylation gives an epigenetic tag for transcriptional activation. Mediates cAMP-gene regulation by binding specifically to phosphorylated CREB protein. Also functions as acetyltransferase for nonhistone targets. Acetylates 'Lys-131' of ALX1 and acts as its coactivator in the presence of CREBBP. Acetylates SIRT2 and is proposed to indirectly increase the transcriptional activity of TP53 through acetylation and [...]	0.9
FABP1	ENSP00000295834	fatty acid binding protein 1, liver; Binds free fatty acids and their coenzyme A derivatives, bilirubin, and some other small molecules in the cytoplasm. May be involved in intracellular lipid transport	0.912
FABP4	ENSP00000256104	fatty acid binding protein 4, adipocyte; Lipid transport protein in adipocytes. Binds both long chain fatty acids and retinoic acid. Delivers long-chain fatty acids and retinoic acid to their cognate receptors in the nucleus (By similarity)	0.987
FABP5	ENSP00000297258	fatty acid binding protein 5 (psoriasis-associated); High specificity for fatty acids. Highest affinity for C18 chain length. Decreasing the chain length or introducing double bonds reduces the affinity. May be involved in keratinocyte differentiation	0.825
FADS1	ENSP00000322229	fatty acid desaturase 1	0.955
FADS2	ENSP00000278840	fatty acid desaturase 2; Component of a lipid metabolic pathway that catalyzes biosynthesis of highly unsaturated fatty acids (HUFA) from precursor essential polyunsaturated fatty acids (PUFA) Linoleic acid (LA) (18-2n-6) and alpha-Linolenic acid (ALA) (18-3n-3). Catalyzes the first and rate limiting step in this pathway which is the desaturation of LA (18-2n-6) and ALA (18-3n-3) into gamma- Linoleic acid (GLA) (18-3n-6) and stearidonic acid (18-4n-3) respectively and other desaturation steps. Highly unsaturated fatty acids (HUFA) play pivotal roles in many biological functions. It cat [...]	0.957
FADS6	ENSP00000307821	fatty acid desaturase domain family, member 6	0.9
FDFT1	ENSP00000220584	farnesyl-diphosphate farnesyltransferase 1	0.907
FFAR1	ENSP00000246553	free fatty acid receptor 1; Receptor for medium and long chain saturated and unsaturated fatty acids. Binding of the ligand increase intracellular calcium concentration and amplify glucose-stimulated insulin secretion. The activity of this receptor is mediated by G-proteins that activate phospholipase C. Seems to act through a G(q) and G(i)-mediated pathway	0.9
FGF21	ENSP00000222157	fibroblast growth factor 21; Stimulates glucose uptake in differentiated adipocytes via the induction of glucose transporter SLC2A1/GLUT1 expression (but not SLC2A4/GLUT4 expression). Activity requires the presence of KLB	0.8
FHL2	ENSP00000322909	four and a half LIM domains 2; May function as a molecular transmitter linking various signaling pathways to transcriptional regulation. Negatively regulates the transcriptional repressor E4F1 and may function in cell growth. Inhibits the transcriptional activity of FOXO1 and its apoptotic function by enhancing the interaction of FOXO1 with SIRT1 and FOXO1 deacetylation	0.9
FOS	ENSP00000306245	FBJ murine osteosarcoma viral oncogene homolog; Nuclear phosphoprotein which forms a tight but non- covalently linked complex with the JUN/AP-1 transcription factor. In the heterodimer, FOS and JUN/AP-1 basic regions each seems to interact with symmetrical DNA half sites. On TGF-beta activation, forms a multimeric SMAD3/SMAD4/JUN/FOS complex at the AP1/SMAD- binding site to regulate TGF-beta-mediated signaling. Has a critical function in regulating the development of cells destined to form and maintain the skeleton. It is thought to have an important role in signal transduction, cell p [...]	0.8
G0S2	ENSP00000355996	G0/G1switch 2; Promotes apoptosis by binding to BCL2, hence preventing the formation of protective BCL2-BAX heterodimers	0.9
GLIPR1	ENSP00000266659	GLI pathogenesis-related 1	0.9
GLTP	ENSP00000315263	glycolipid transfer protein; Accelerates the intermembrane transfer of various glycolipids. Catalyzes the transfer of various glycosphingolipids between membranes but does not catalyze the transfer of phospholipids. May be involved in the intracellular translocation of glucosylceramides	0.8
GOT2	ENSP00000245206	glutamic-oxaloacetic transaminase 2, mitochondrial (aspartate aminotransferase 2); Catalyzes the irreversible transamination of the L- tryptophan metabolite L-kynurenine to form kynurenic acid (KA). Plays a key role in amino acid metabolism. Important for metabolite exchange between mitochondria and cytosol. Facilitates cellular uptake of long-chain free fatty acids	0.44
GPC1	ENSP00000264039	glypican 1; Cell surface proteoglycan that bears heparan sulfate. Binds, via the heparan sulfate side chains, alpha-4 (V) collagen and participates in Schwann cell myelination (By similarity). May act as a catalyst in increasing the rate of conversion of prion protein PRPN(C) to PRNP(Sc) via associating (via the heparan sulfate side chains) with both forms of PRPN, targeting them to lipid rafts and facilitating their interaction. Required for proper skeletal muscle differentiation by sequestering FGF2 in lipid rafts preventing its binding to receptors (FGFRs) and inhibiting the FGF-med [...]	0.9
GPC2	ENSP00000292377	glypican 2; Cell surface proteoglycan that bears heparan sulfate. May fulfill a function related to the motile behaviors of developing neurons (By similarity)	0.9
GPC3	ENSP00000377836	glypican 3; Cell surface proteoglycan that bears heparan sulfate. Inhibits the dipeptidyl peptidase activity of DPP4. May be involved in the suppression/modulation of growth in the predominantly mesodermal tissues and organs. May play a role in the modulation of IGF2 interactions with its receptor and thereby modulate its function. May regulate growth and tumor predisposition	0.9
GPC4	ENSP00000359864	glypican 4; Cell surface proteoglycan that bears heparan sulfate. May be involved in the development of kidney tubules and of the central nervous system (By similarity)	0.909

GPC5	ENSP00000366267	glypican 5; Cell surface proteoglycan that bears heparan sulfate (By similarity)	0.9
GPC6	ENSP00000366246	glypican 6; Cell surface proteoglycan that bears heparan sulfate. Putative cell surface coreceptor for growth factors, extracellular matrix proteins, proteases and anti-proteases (By similarity). Enhances migration and invasion of cancer cells through WNT5A signaling	0.909
GRHL1	ENSP00000324693	grainyhead-like 1 (Drosophila)	0.9
HDAC3	ENSP00000302967	histone deacetylase 3; Responsible for the deacetylation of lysine residues on the N-terminal part of the core histones (H2A, H2B, H3 and H4), and some other non-histone substrates. Histone deacetylation gives a tag for epigenetic repression and plays an important role in transcriptional regulation, cell cycle progression and developmental events. Histone deacetylases act via the formation of large multiprotein complexes. Probably participates in the regulation of transcription through its binding to the zinc-finger transcription factor YY1; increases YY1 repression activity. Required [...]	0.913
HELZ2	ENSP00000417401	helicase with zinc finger 2, transcriptional coactivator; Helicase that acts as a transcriptional coactivator for a number of nuclear receptors including PPARA, PPARG, THRA, THRB and RXRA	0.9
HIF1A	ENSP00000338018	hypoxia inducible factor 1, alpha subunit (basic helix-loop-helix transcription factor); Functions as a master transcriptional regulator of the adaptive response to hypoxia. Under hypoxic conditions, activates the transcription of over 40 genes, including erythropoietin, glucose transporters, glycolytic enzymes, vascular endothelial growth factor, HILPDA, and other genes whose protein products increase oxygen delivery or facilitate metabolic adaptation to hypoxia. Plays an essential role in embryonic vascularization, tumor angiogenesis and pathophysiology of ischemic disease. Binds to [...]	0.907
HMGCR	ENSP00000287936	3-hydroxy-3-methylglutaryl-CoA reductase; Transmembrane glycoprotein that is the rate-limiting enzyme in cholesterol biosynthesis as well as in the biosynthesis of nonsterol isoprenoids that are essential for normal cell function including ubiquinone and geranylgeranyl proteins	0.945
HMGCS1	ENSP00000322706	3-hydroxy-3-methylglutaryl-CoA synthase 1 (soluble); This enzyme condenses acetyl-CoA with acetoacetyl-CoA to form HMG-CoA, which is the substrate for HMG-CoA reductase	0.909
HMGCS2	ENSP00000358414	3-hydroxy-3-methylglutaryl-CoA synthase 2 (mitochondrial); This enzyme condenses acetyl-CoA with acetoacetyl-CoA to form HMG-CoA, which is the substrate for HMG-CoA reductase	0.911
HSPG2	ENSP00000363827	heparan sulfate proteoglycan 2; Integral component of basement membranes. Component of the glomerular basement membrane (GBM), responsible for the fixed negative electrostatic membrane charge, and which provides a barrier which is both size- and charge-selective. It serves as an attachment substrate for cells. Plays essential roles in vascularization. Critical for normal heart development and for regulating the vascular response to injury. Also required for avascular cartilage development	0.9
IL6	ENSP00000258743	interleukin 6 (interferon, beta 2); Cytokine with a wide variety of biological functions. It is a potent inducer of the acute phase response. Plays an essential role in the final differentiation of B-cells into Ig- secreting cells Involved in lymphocyte and monocyte differentiation. It induces myeloma and plasmacytoma growth and induces nerve cells differentiation Acts on B-cells, T-cells, hepatocytes, hematopoietic progenitor cells and cells of the CNS. Also acts as a myokine. It is discharged into the bloodstream after muscle contraction and acts to increase the breakdown of fats and [...]	0.839
LCAT	ENSP00000264005	lecithin-cholesterol acyltransferase; Central enzyme in the extracellular metabolism of plasma lipoproteins. Synthesized mainly in the liver and secreted into plasma where it converts cholesterol and phosphatidylcholines (lecithins) to cholesteryl esters and lysophosphatidylcholines on the surface of high and low density lipoproteins (HDLs and LDLs). The cholesterol ester is then transported back to the liver. Has a preference for plasma 16-0-18-2 or 18-O-18-2 phosphatidylcholines. Also produced in the brain by primary astrocytes, and esterifies free cholesterol on nascent APOE-contain [...]	0.427
LCLAT1	ENSP00000310551	lysocardiolipin acyltransferase 1; Acyl-CoA-lysocardiolipin acyltransferase. Possesses both lysophosphatidylinositol acyltransferase (LPIAT) and lysophosphatidylglycerol acyltransferase (LPGAT) activities. Recognizes both monolysocardiolipin and dilysocardiolipin as substrates with a preference for linoleoyl-CoA and oleoyl-CoA as acyl donors. Acts as a remodeling enzyme for cardiolipin, a major membrane polyglycerophospholipid. Converts lysophosphatidic acid (LPA) into phosphatidic acid (PA) with a relatively low activity. Required for establishment of the hematopoietic and endothelial [...]	0.415
LDLR	ENSP00000454071	low density lipoprotein receptor	0.916
LEP	ENSP00000312652	leptin; May function as part of a signaling pathway that acts to regulate the size of the body fat depot. An increase in the level of LEP may act directly or indirectly on the CNS to inhibit food intake and/or regulate energy expenditure as part of a homeostatic mechanism to maintain constancy of the adipose mass	0.866
LOX	ENSP00000231004	lysyl oxidase; Responsible for the post-translational oxidative deamination of peptidyl lysine residues in precursors to fibrous collagen and elastin. In addition to cross-linking of extracellular matrix proteins, may have a direct role in tumor suppression	0.504
LPL	ENSP00000309757	lipoprotein lipase; The primary function of this lipase is the hydrolysis of triglycerides of circulating chylomicrons and very low density lipoproteins (VLDL). Binding to heparin sulfate proteogylcans at the cell surface is vital to the function. The apolipoprotein, APOC2, acts as a coactivator of LPL activity in the presence of lipids on the luminal surface of vascular endothelium (By similarity)	0.462
LRAT	ENSP00000337224	lecithin retinol acyltransferase (phosphatidylcholine--retinol O-acyltransferase); Transfers the acyl group from the sn-1 position of phosphatidylcholine to all-trans retinol, producing all-trans retinyl esters. Retinyl esters are storage forms of vitamin A. LRAT plays a critical role in vision. It provides the all-trans retinyl ester substrates for the isomerohydrolase which processes the esters into 11-cis-retinol in the retinal pigment epithelium; due to a membrane-associated alcohol dehydrogenase, 11 cis-retinol is oxidized and converted into 11-cis-retinaldehyde which is the chrom [...]	0.9
LRP1	ENSP00000243077	low density lipoprotein receptor-related protein 1; Endocytic receptor involved in endocytosis and in phagocytosis of apoptotic cells. Required for early embryonic development. Involved in cellular lipid homeostasis. Involved in the plasma clearance of chylomicron remnants and activated LRPAP1 (alpha 2-macroglobulin), as well as the local metabolism of complexes between plasminogen activators and their endogenous inhibitors. May modulate cellular events, such as APP metabolism, kinase-dependent intracellular signaling, neuronal calcium signaling as well as neurotransmission	0.9
LRP2	ENSP00000263816	low density lipoprotein receptor-related protein 2; Acts together with cubilin to mediate HDL endocytosis (By similarity). May participate in regulation of parathyroid- hormone and para-thyroid-hormone-related protein release	0.9
LRP8	ENSP00000303634	low density lipoprotein receptor-related protein 8, apolipoprotein e receptor; Cell surface receptor for Reelin (RELN) and apolipoprotein E (apoE)-containing ligands. LRP8 participates in transmitting the extracellular Reelin signal to intracellular signaling processes, by binding to DAB1 on its cytoplasmic tail. Reelin acts via both the VLDL receptor (VLDLR) and LRP8 to regulate DAB1 tyrosine phosphorylation and microtubule function in neurons. LRP8 has higher affinity for Reelin than VLDLR. LRP8 is thus a key component of the Reelin pathway which governs neuronal layering of the fore [...]	0.9
MAPK8	ENSP00000353483	mitogen-activated protein kinase 8; Serine/threonine-protein kinase involved in various processes such as cell proliferation, differentiation, migration, transformation and programmed cell death. Extracellular stimuli such as proinflammatory cytokines or physical stress stimulate the stress-activated protein kinase/c-Jun N-terminal kinase (SAP/JNK) signaling pathway. In this cascade, two dual specificity kinases MAP2K4/MKK4 and MAP2K7/MKK7 phosphorylate and activate MAPK8/JNK1. In turn, MAPK8/JNK1 phosphorylates a number of transcription factors, primarily components of AP-1 such as JU [...]	0.822
ME1	ENSP00000358719	malic enzyme 1, NADP(+)-dependent, cytosolic	0.9
MED1	ENSP00000300651	mediator complex subunit 1; Component of the Mediator complex, a coactivator involved in the regulated transcription of nearly all RNA polymerase II-dependent genes. Mediator functions as a bridge to convey information from gene-specific regulatory proteins to the basal RNA polymerase II transcription machinery. Mediator is recruited to promoters by direct interactions with regulatory proteins and serves as a scaffold for the assembly of a functional preinitiation complex with RNA polymerase II and the general transcription factors	0.913
MED10	ENSP00000255764	mediator complex subunit 10; Component of the Mediator complex, a coactivator involved in the regulated transcription of nearly all RNA polymerase II-dependent genes. Mediator functions as a bridge to convey information from gene-specific regulatory proteins to the basal RNA polymerase II transcription machinery. Mediator is recruited to promoters by direct interactions with regulatory proteins and serves as a scaffold for the assembly of a functional preinitiation complex with RNA polymerase II and the general transcription factors	0.9
MED11	ENSP00000293777	mediator complex subunit 11; Component of the Mediator complex, a coactivator involved in the regulated transcription of nearly all RNA polymerase II-dependent genes. Mediator functions as a bridge to convey information from gene-specific regulatory proteins to the basal RNA polymerase II transcription machinery. Mediator is recruited to promoters by direct interactions with regulatory proteins and serves as a scaffold for the assembly of a functional preinitiation complex with RNA polymerase II and the general transcription factors	0.9
MED12	ENSP00000363193	mediator complex subunit 12; Component of the Mediator complex, a coactivator involved in the regulated transcription of nearly all RNA polymerase II-dependent genes. Mediator functions as a bridge to convey information from gene-specific regulatory proteins to the basal RNA polymerase II transcription machinery. Mediator is recruited to promoters by direct interactions with regulatory proteins and serves as a scaffold for the assembly of a functional preinitiation complex with RNA polymerase II and the general transcription factors. This subunit may specifically regulate transcription [...]	0.9
MED13	ENSP00000380888	mediator complex subunit 13; Component of the Mediator complex, a coactivator involved in the regulated transcription of nearly all RNA polymerase II-dependent genes. Mediator functions as a bridge to convey information from gene-specific regulatory proteins to the basal RNA polymerase II transcription machinery. Mediator is recruited to promoters by direct interactions with regulatory proteins and serves as a scaffold for the assembly of a functional preinitiation complex with RNA polymerase II and the general transcription factors	0.9
MED13L	ENSP00000281928	mediator complex subunit 13-like; Component of the Mediator complex, a coactivator involved in the regulated transcription of nearly all RNA polymerase II-dependent genes. Mediator functions as a bridge to convey information from gene-specific regulatory proteins to the basal RNA polymerase II transcription machinery. Mediator is recruited to promoters by direct interactions with regulatory proteins and serves as a scaffold for the assembly of a functional preinitiation complex with RNA polymerase II and the general transcription factors. This subunit may specifically regulate transcri [...]	0.9
MED14	ENSP00000323720	mediator complex subunit 14; Component of the Mediator complex, a coactivator involved in the regulated transcription of nearly all RNA polymerase II-dependent genes. Mediator functions as a bridge to convey information from gene-specific regulatory proteins to the basal RNA polymerase II transcription machinery. Mediator is recruited to promoters by direct interactions with regulatory proteins and serves as a scaffold for the assembly of a functional preinitiation complex with RNA polymerase II and the general transcription factors	0.9
MED15	ENSP00000263205	mediator complex subunit 15	0.9
MED16	ENSP00000325612	mediator complex subunit 16; Component of the Mediator complex, a coactivator involved in the regulated transcription of nearly all RNA polymerase II-dependent genes. Mediator functions as a bridge to convey information from gene-specific regulatory proteins to the basal RNA polymerase II transcription machinery. Mediator is recruited to promoters by direct interactions with regulatory proteins and serves as a scaffold for the assembly of a functional preinitiation complex with RNA polymerase II and the general transcription factors	0.9

MED17	ENSP00000251871	mediator complex subunit 17; Component of the Mediator complex, a coactivator involved in the regulated transcription of nearly all RNA polymerase II-dependent genes. Mediator functions as a bridge to convey information from gene-specific regulatory proteins to the basal RNA polymerase II transcription machinery. Mediator is recruited to promoters by direct interactions with regulatory proteins and serves as a scaffold for the assembly of a functional preinitiation complex with RNA polymerase II and the general transcription factors	0.9
MED18	ENSP00000362948	mediator complex subunit 18; Component of the Mediator complex, a coactivator involved in the regulated transcription of nearly all RNA polymerase II-dependent genes. Mediator functions as a bridge to convey information from gene-specific regulatory proteins to the basal RNA polymerase II transcription machinery. Mediator is recruited to promoters by direct interactions with regulatory proteins and serves as a scaffold for the assembly of a functional preinitiation complex with RNA polymerase II and the general transcription factors	0.9
MED19	ENSP00000337340	mediator complex subunit 19; Component of the Mediator complex, a coactivator involved in the regulated transcription of nearly all RNA polymerase II-dependent genes. Mediator functions as a bridge to convey information from gene-specific regulatory proteins to the basal RNA polymerase II transcription machinery. Mediator is recruited to promoters by direct interactions with regulatory proteins and serves as a scaffold for the assembly of a functional preinitiation complex with RNA polymerase II and the general transcription factors	0.9
MED20	ENSP00000265350	mediator complex subunit 20; Component of the Mediator complex, a coactivator involved in the regulated transcription of nearly all RNA polymerase II-dependent genes. Mediator functions as a bridge to convey information from gene-specific regulatory proteins to the basal RNA polymerase II transcription machinery. Mediator is recruited to promoters by direct interactions with regulatory proteins and serves as a scaffold for the assembly of a functional preinitiation complex with RNA polymerase II and the general transcription factors	0.9
MED21	ENSP00000282892	mediator complex subunit 21; Component of the Mediator complex, a coactivator involved in the regulated transcription of nearly all RNA polymerase II-dependent genes. Mediator functions as a bridge to convey information from gene-specific regulatory proteins to the basal RNA polymerase II transcription machinery. Mediator is recruited to promoters by direct interactions with regulatory proteins and serves as a scaffold for the assembly of a functional preinitiation complex with RNA polymerase II and the general transcription factors	0.9
MED22	ENSP00000342343	mediator complex subunit 22; Component of the Mediator complex, a coactivator involved in the regulated transcription of nearly all RNA polymerase II-dependent genes. Mediator functions as a bridge to convey information from gene-specific regulatory proteins to the basal RNA polymerase II transcription machinery. Mediator is recruited to promoters by direct interactions with regulatory proteins and serves as a scaffold for the assembly of a functional preinitiation complex with RNA polymerase II and the general transcription factors	0.9
MED23	ENSP00000357047	mediator complex subunit 23	0.9
MED24	ENSP00000377686	mediator complex subunit 24; Component of the Mediator complex, a coactivator involved in the regulated transcription of nearly all RNA polymerase II-dependent genes. Mediator functions as a bridge to convey information from gene-specific regulatory proteins to the basal RNA polymerase II transcription machinery. Mediator is recruited to promoters by direct interactions with regulatory proteins and serves as a scaffold for the assembly of a functional preinitiation complex with RNA polymerase II and the general transcription factors	0.9
MED25	ENSP00000326767	mediator complex subunit 25	0.9
MED26	ENSP00000263390	mediator complex subunit 26; Component of the Mediator complex, a coactivator involved in the regulated transcription of nearly all RNA polymerase II-dependent genes. Mediator functions as a bridge to convey information from gene-specific regulatory proteins to the basal RNA polymerase II transcription machinery. Mediator is recruited to promoters by direct interactions with regulatory proteins and serves as a scaffold for the assembly of a functional preinitiation complex with RNA polymerase II and the general transcription factors	0.9
MED27	ENSP00000292035	mediator complex subunit 27; Component of the Mediator complex, a coactivator involved in the regulated transcription of nearly all RNA polymerase II-dependent genes. Mediator functions as a bridge to convey information from gene-specific regulatory proteins to the basal RNA polymerase II transcription machinery. Mediator is recruited to promoters by direct interactions with regulatory proteins and serves as a scaffold for the assembly of a functional preinitiation complex with RNA polymerase II and the general transcription factors	0.9
MED29	ENSP00000314343	mediator complex subunit 29; Component of the Mediator complex, a coactivator involved in the regulated transcription of nearly all RNA polymerase II-dependent genes. Mediator functions as a bridge to convey information from gene-specific regulatory proteins to the basal RNA polymerase II transcription machinery. Mediator is recruited to promoters by direct interactions with regulatory proteins and serves as a scaffold for the assembly of a functional preinitiation complex with RNA polymerase II and the general transcription factors	0.9
MED30	ENSP00000297347	mediator complex subunit 30; Component of the Mediator complex, a coactivator involved in the regulated transcription of nearly all RNA polymerase II-dependent genes. Mediator functions as a bridge to convey information from gene-specific regulatory proteins to the basal RNA polymerase II transcription machinery. Mediator is recruited to promoters by direct interactions with regulatory proteins and serves as a scaffold for the assembly of a functional preinitiation complex with RNA polymerase II and the general transcription factors	0.9
MED31	ENSP00000225728	mediator complex subunit 31; Component of the Mediator complex, a coactivator involved in the regulated transcription of nearly all RNA polymerase II-dependent genes. Mediator functions as a bridge to convey information from gene-specific regulatory proteins to the basal RNA polymerase II transcription machinery. Mediator is recruited to promoters by direct interactions with regulatory proteins and serves as a scaffold for the assembly of a functional preinitiation complex with RNA polymerase II and the general transcription factors	0.9
MED4	ENSP00000258648	mediator complex subunit 4; Component of the Mediator complex, a coactivator involved in the regulated transcription of nearly all RNA polymerase II-dependent genes. Mediator functions as a bridge to convey information from gene-specific regulatory proteins to the basal RNA polymerase II transcription machinery. Mediator is recruited to promoters by direct interactions with regulatory proteins and serves as a scaffold for the assembly of a functional preinitiation complex with RNA polymerase II and the general transcription factors	0.9
MED6	ENSP00000256379	mediator complex subunit 6; Component of the Mediator complex, a coactivator involved in the regulated transcription of nearly all RNA polymerase II-dependent genes. Mediator functions as a bridge to convey information from gene-specific regulatory proteins to the basal RNA polymerase II transcription machinery. Mediator is recruited to promoters by direct interactions with regulatory proteins and serves as a scaffold for the assembly of a functional preinitiation complex with RNA polymerase II and the general transcription factors (By similarity)	0.9
MED7	ENSP00000286317	mediator complex subunit 7; Component of the Mediator complex, a coactivator involved in the regulated transcription of nearly all RNA polymerase II-dependent genes. Mediator functions as a bridge to convey information from gene-specific regulatory proteins to the basal RNA polymerase II transcription machinery. Mediator is recruited to promoters by direct interactions with regulatory proteins and serves as a scaffold for the assembly of a functional preinitiation complex with RNA polymerase II and the general transcription factors	0.9
MED8	ENSP00000290663	mediator complex subunit 8; Component of the Mediator complex, a coactivator involved in the regulated transcription of nearly all RNA polymerase II-dependent genes. Mediator functions as a bridge to convey information from gene-specific regulatory proteins to the basal RNA polymerase II transcription machinery. Mediator is recruited to promoters by direct interactions with regulatory proteins and serves as a scaffold for the assembly of a functional preinitiation complex with RNA polymerase II and the general transcription factors. May play a role as a target recruitment subunit in E3 [...]	0.9
MED9	ENSP00000268711	mediator complex subunit 9; Component of the Mediator complex, a coactivator involved in the regulated transcription of nearly all RNA polymerase II-dependent genes. Mediator functions as a bridge to convey information from gene-specific regulatory proteins to the basal RNA polymerase II transcription machinery. Mediator is recruited to promoters by direct interactions with regulatory proteins and serves as a scaffold for the assembly of a functional preinitiation complex with RNA polymerase II and the general transcription factors	0.9
MME	ENSP00000353679	membrane metallo-endopeptidase; Thermolysin-like specificity, but is almost confined on acting on polypeptides of up to 30 amino acids. Biologically important in the destruction of opioid peptides such as Met- and Leu-enkephalins by cleavage of a Gly-Phe bond. Able to cleave angiotensin-1, angiotensin-2 and angiotensin 1-9. Involved in the degradation of atrial natriuretic factor (ANF). Displays UV- inducible elastase activity toward skin preelastic and elastic fibers	0.8
MYO7A	ENSP00000386331	myosin VIIA; Myosins are actin-based motor molecules with ATPase activity. Unconventional myosins serve in intracellular movements. Their highly divergent tails bind to membranous compartments, which are then moved relative to actin filaments. In the retina, plays an important role in the renewal of the outer photoreceptor disks. Plays an important role in the distribution and migration of retinal pigment epithelial (RPE) melanosomes and phagosomes, and in the regulation of opsin transport in retinal photoreceptors. In the inner ear, plays an important role in differentiation, morphoge [...]	0.9
NCOA1	ENSP00000320940	nuclear receptor coactivator 1	0.9
NCOA2	ENSP00000399968	nuclear receptor coactivator 2; Transcriptional coactivator for steroid receptors and nuclear receptors. Coactivator of the steroid binding domain (AF- 2) but not of the modulating N-terminal domain (AF-1). Required with NCOA1 to control energy balance between white and brown adipose tissues	0.9
NCOA3	ENSP00000361066	nuclear receptor coactivator 3	0.9
NCOA6	ENSP00000351894	nuclear receptor coactivator 6; Nuclear receptor coactivator that directly binds nuclear receptors and stimulates the transcriptional activities in a hormone-dependent fashion. Coactivates expression in an agonist- and AF2-dependent manner. Involved in the coactivation of different nuclear receptors, such as for steroids (GR and ERs), retinoids (RARs and RXRs), thyroid hormone (TRs), vitamin D3 (VDR) and prostanoids (PPARs). Probably functions as a general coactivator, rather than just a nuclear receptor coactivator. May also be involved in the coactivation of the NF-kappa-B pathway. M [...]	0.914
NCOR1	ENSP00000268712	nuclear receptor corepressor 1; Mediates transcriptional repression by certain nuclear receptors. Part of a complex which promotes histone deacetylation and the formation of repressive chromatin structures which may impede the access of basal transcription factors	0.908
NCOR2	ENSP00000384018	nuclear receptor corepressor 2; Transcriptional corepressor of NR4A2/NURR1 and acts through histone deacetylases (HDACs) to keep promoters of NR4A2/NURR1 target genes in a repressed deacetylated state (By similarity). Mediates the transcriptional repression activity of some nuclear receptors by promoting chromatin condensation, thus preventing access of the basal transcription. Isoform 1 and isoform 5 have different affinities for different nuclear receptors	0.921
NFYA	ENSP00000345702	nuclear transcription factor Y, alpha; Stimulates the transcription of various genes by recognizing and binding to a CCAAT motif in promoters, for example in type 1 collagen, albumin and beta-actin genes	0.9
NFYB	ENSP00000240055	nuclear transcription factor Y, beta; Stimulates the transcription of various genes by recognizing and binding to a CCAAT motif in promoters, for example in type 1 collagen, albumin and beta-actin genes	0.917
NFYC	ENSP00000396620	nuclear transcription factor Y, gamma	0.9
NPAS2	ENSP00000338283	neuronal PAS domain protein 2; BMAL1-NPAS2 heterodimers activate E-box element (5'- CACGTG-3') transcription of a number of proteins of the circadian clock. This transcription is inhibited in a feedback loop by PER, and also by CRY proteins (By similarity)	0.9

NRF1	ENSP00000223190	nuclear respiratory factor 1; Transcription factor that activates the expression of the EIF2S1 (EIF2-alpha) gene. Links the transcriptional modulation of key metabolic genes to cellular growth and development. Implicated in the control of nuclear genes required for respiration, heme biosynthesis, and mitochondrial DNA transcription and replication	0.9
OLR1	ENSP00000309124	oxidized low density lipoprotein (lectin-like) receptor 1; Receptor that mediates the recognition, internalization and degradation of oxidatively modified low density lipoprotein (oxLDL) by vascular endothelial cells. OxLDL is a marker of atherosclerosis that induces vascular endothelial cell activation and dysfunction, resulting in pro-inflammatory responses, pro- oxidative conditions and apoptosis. Its association with oxLDL induces the activation of NF-kappa-B through an increased production of intracellular reactive oxygen and a variety of pro- atherogenic cellular responses includ [...]	0.897
PAEP	ENSP00000277508	progestagen-associated endometrial protein; This protein is, quantitatively, the main protein synthesized and secreted in the endometrium from mid-luteal phase of the menstrual cycle and during the first semester of pregnancy	0.773
PCK1	ENSP00000319814	phosphoenolpyruvate carboxykinase 1 (soluble); Catalyzes the conversion of oxaloacetate (OAA) to phosphoenolpyruvate (PEP), the rate-limiting step in the metabolic pathway that produces glucose from lactate and other precursors derived from the citric acid cycle	0.859
PCK2	ENSP00000216780	phosphoenolpyruvate carboxykinase 2 (mitochondrial); Catalyzes the conversion of oxaloacetate (OAA) to phosphoenolpyruvate (PEP), the rate-limiting step in the metabolic pathway that produces glucose from lactate and other precursors derived from the citric acid cycle (By similarity)	0.862
PEX11A	ENSP00000300056	peroxisomal biogenesis factor 11 alpha; May be involved in peroxisomal proliferation and may regulate peroxisomes division. May mediate binding of coatomer proteins to the peroxisomal membrane	0.9
PLA2G10	ENSP00000393847	phospholipase A2, group X; PA2 catalyzes the calcium-dependent hydrolysis of the 2- acyl groups in 3-sn-phosphoglycerides. Has a powerful potency for releasing arachidonic acid from cell membrane phospholipids. Prefers phosphatidylethanolamine and phosphatidylcholine liposomes to those of phosphatidylserine	0.909
PLA2G12A	ENSP00000243501	phospholipase A2, group XIIA; PA2 catalyzes the calcium-dependent hydrolysis of the 2- acyl groups in 3-sn-phosphoglycerides. Does not exhibit detectable activity toward sn-2-arachidonoyl- or linoleoyl- phosphatidylcholine or -phosphatidylethanolamine	0.9
PLA2G12B	ENSP00000362123	phospholipase A2, group XIIB; Not known; does not seem to have catalytic activity	0.9
PLA2G16	ENSP00000320337	phospholipase A2, group XVI; Exhibits PLA1/2 activity, catalyzing the calcium- independent hydrolysis of acyl groups in various phosphatidylcholines (PC) and phosphatidylethanolamine (PE). For most substrates, PLA1 activity is much higher than PLA2 activity. Specifically catalyzes the release of fatty acids from phospholipids in adipose tissue (By similarity). N- and O- acylation activity is hardly detectable. Might decrease protein phosphatase 2A (PP2A) activity	0.9
PLA2G1B	ENSP00000312286	phospholipase A2, group IB (pancreas); PA2 catalyzes the calcium-dependent hydrolysis of the 2- acyl groups in 3-sn-phosphoglycerides, this releases glycerophospholipids and arachidonic acid that serve as the precursors of signal molecules	0.932
PLA2G2A	ENSP00000364252	phospholipase A2, group IIA (platelets, synovial fluid); Thought to participate in the regulation of the phospholipid metabolism in biomembranes including eicosanoid biosynthesis. Catalyzes the calcium-dependent hydrolysis of the 2- acyl groups in 3-sn-phosphoglycerides	0.925
PLA2G2C	ENSP00000247992	phospholipase A2, group IIC; Inactive phospholipase (Probable)	0.9
PLA2G2E	ENSP00000364257	phospholipase A2, group IIE; PA2 catalyzes the calcium-dependent hydrolysis of the 2- acyl groups in 3-sn-phosphoglycerides. Has a preference for arachidonic-containing phospholipids	0.9
PLA2G2F	ENSP00000364243	phospholipase A2, group IIF; PA2 catalyzes the calcium-dependent hydrolysis of the 2- acyl groups in 3-sn-phosphoglycerides. Hydrolyzes phosphatidylglycerol versus phosphatidylcholine with a 15-fold preference	0.9
PLA2G3	ENSP00000215885	phospholipase A2, group III; PA2 catalyzes the calcium-dependent hydrolysis of the 2- acyl groups in 3-sn-phosphoglycerides. Shows an 11-fold preference for phosphatidylglycerol over phosphatidylcholine (PC). Preferential cleavage- 1-palmitoyl-2-linoleoyl-phosphatidylethanolamine (PE) > 1-palmitoyl-2-linoleoyl-PC > 1- palmitoyl-2-arachidonoyl-PC > 1-palmitoyl-2-arachidonoyl-PE. Plays a role in ciliogenesis	0.9
PLA2G4A	ENSP00000356436	phospholipase A2, group IVA (cytosolic, calcium-dependent); Selectively hydrolyzes arachidonyl phospholipids in the sn-2 position releasing arachidonic acid. Together with its lysophospholipid activity, it is implicated in the initiation of the inflammatory response	0.926
PLA2G4B	ENSP00000396045	phospholipase A2, group IVB (cytosolic); Calcium-dependent phospholipase A2 that selectively hydrolyzes glycerophospholipids in the sn-2 position with a preference for arachidonoyl phospholipids. Has a much weaker activity than PLA2G4A. Isoform 3 has calcium-dependent activity against palmitoyl-arachidonyl-phosphatidylethanolamine and low level lysophospholipase activity but no activity against phosphatidylcholine. Isoform 5 does have activity against phosphatidylcholine	0.9
PLA2G4C	ENSP00000400036	phospholipase A2, group IVC (cytosolic, calcium-independent); Has a preference for arachidonic acid at the sn-2 position of phosphatidylcholine as compared with palmitic acid	0.9
PLA2G4D	ENSP00000290472	phospholipase A2, group IVD (cytosolic); Calcium-dependent phospholipase A2 that selectively hydrolyzes glycerophospholipids in the sn-2 position. Not arachidonic acid-specific but has Linoleic acid-specific activity. May play a role in inflammation in psoriatic lesions	0.9
PLA2G4E	ENSP00000382434	phospholipase A2, group IVE; Calcium-dependent phospholipase A2 that selectively hydrolyzes glycerophospholipids in the sn-2 position (By similarity)	0.9
PLA2G4F	ENSP00000371833	phospholipase A2, group IVF; Calcium-dependent phospholipase A2 that selectively hydrolyzes glycerophospholipids in the sn-2 position. Has higher enzyme activity for phosphatidylethanolamine than phosphatidylcholine (By similarity)	0.9
PLA2G5	ENSP00000364249	phospholipase A2, group V; PA2 catalyzes the calcium-dependent hydrolysis of the 2- acyl groups in 3-sn-phosphoglycerides. This isozyme hydrolyzes more efficiently L-alpha-1-palmitoyl-2-oleoyl phosphatidylcholine than L-alpha-1-palmitoyl-2-arachidonyl phosphatidylcholine, L- alpha-1-palmitoyl-2-arachidonyl phosphatidylethanolamine, or L- alpha-1-stearoyl-2-arachidonyl phosphatidylinositol. May be involved in the production of lung surfactant, the remodeling or regulation of cardiac muscle	0.9
PLA2G6	ENSP00000333142	phospholipase A2, group VI (cytosolic, calcium-independent); Catalyzes the release of fatty acids from phospholipids. It has been implicated in normal phospholipid remodeling, nitric oxide-induced or vasopressin-induced arachidonic acid release and in leukotriene and prostaglandin production. May participate in fas mediated apoptosis and in regulating transmembrane ion flux in glucose-stimulated B-cells. Has a role in cardiolipin (CL) deacylation. Required for both speed and directionality of monocyte MCP1/CCL2-induced chemotaxis through regulation of F- actin polymerization at the pse [...]	0.917
PLB1	ENSP00000330442	phospholipase B1; Membrane-associated phospholipase. Exhibits a calcium- independent broad substrate specificity including phospholipase A2/lysophospholipase activity. Preferential hydrolysis at the sn-2 position of diacylphospholipids and diacyglycerol, whereas it shows no positional specificity toward triacylglycerol. Exhibits also esterase activity toward p-nitrophenyl. May act on the brush border membrane to facilitate the absorption of digested lipids (By similarity)	0.9
PLIN2	ENSP00000276914	perilipin 2; May be involved in development and maintenance of adipose tissue (By similarity)	0.909
PON1	ENSP00000222381	paraoxonase 1; Hydrolyzes the toxic metabolites of a variety of organophosphorus insecticides. Capable of hydrolyzing a broad spectrum of organophosphate substrates and lactones, and a number of aromatic carboxylic acid esters. Mediates an enzymatic protection of low density lipoproteins against oxidative modification and the consequent series of events leading to atheroma formation	0.816
PPARA	ENSP00000262735	peroxisome proliferator-activated receptor alpha; Ligand-activated transcription factor. Key regulator of lipid metabolism. Activated by the endogenous ligand 1-palmitoyl- 2-oleoyl-sn-glycerol-3-phosphocholine (16-0/18-1-GPC). Activated by oleylethanolamide, a naturally occurring lipid that regulates satiety (By similarity). Receptor for peroxisome proliferators such as hypolipidemic drugs and fatty acids. Regulates the peroxisomal beta-oxidation pathway of fatty acids. Functions as transcription activator for the ACOX1 and P450 genes. Transactivation activity requires heterodimerizati [...]	0.992
PPARG	ENSP00000287820	peroxisome proliferator-activated receptor gamma	0.953
PPARGC1A	ENSP00000264867	peroxisome proliferator-activated receptor gamma, coactivator 1 alpha; Transcriptional coactivator for steroid receptors and nuclear receptors. Greatly increases the transcriptional activity of PPARG and thyroid hormone receptor on the uncoupling protein promoter. Can regulate key mitochondrial genes that contribute to the program of adaptive thermogenesis. Plays an essential role in metabolic reprogramming in response to dietary availability through coordination of the expression of a wide array of genes involved in glucose and fatty acid metabolism	0.98
PPARGC1B	ENSP00000312649	peroxisome proliferator-activated receptor gamma, coactivator 1 beta	0.9
PTGS2	ENSP00000356438	prostaglandin-endoperoxide synthase 2 (prostaglandin G/H synthase and cyclooxygenase); Mediates the formation of prostaglandins from arachidonate. May have a role as a major mediator of inflammation and/or a role for prostanoid signaling in activity-dependent plasticity	0.603
RBP1	ENSP00000232219	retinol binding protein 1, cellular; Intracellular transport of retinol	0.9
RGL1	ENSP00000303192	ral guanine nucleotide dissociation stimulator-like 1; Probable guanine nucleotide exchange factor	0.9
RORA	ENSP00000261523	RAR-related orphan receptor A; Orphan nuclear receptor. Binds DNA as a monomer to hormone response elements (HRE) containing a single core motif half-site preceded by a short A-T-rich sequence. This isomer binds to the consensus sequence 5'-[AT][TA]A[AT][CGT]TAGGTCA-3'. Regulates a number of genes involved in lipid metabolism such as apolipoproteins AI, APOA5, CIII, CYP71 and PPARgamma, in cerebellum and photoreceptor development including PCP2, OPN1SW, OPN1SM AND ARR3, in circadian rhythm with BMAL1, and skeletal muscle development with MYOD1. Possible receptor for cholesterol or one [...]	0.9
RPE65	ENSP00000262340	retinal pigment epithelium-specific protein 65kDa; Plays important roles in the production of 11-cis retinal and in visual pigment regeneration. The soluble form binds vitamin A (all-trans-retinol), making it available for LRAT processing to all-trans-retinyl ester. The membrane form, palmitoylated by LRAT, binds all-trans-retinyl esters, making them available for IMH (isomerohydrolase) processing to all-cis- retinol. The soluble form is regenerated by transferring its palmitoyl groups onto 11-cis-retinol, a reaction catalyzed by LRAT. The enzymatic activity is linearly dependent of th [...]	0.9
RXRA	ENSP00000419692	retinoid X receptor, alpha; Receptor for retinoic acid. Retinoic acid receptors bind as heterodimers to their target response elements in response to their ligands, all-trans or 9-cis retinoic acid, and regulate gene expression in various biological processes. The RAR/RXR heterodimers bind to the retinoic acid response elements (RARE) composed of tandem 5'-AGGTCA-3' sites known as DR1-DR5. The high affinity ligand for RXRs is 9-cis retinoic acid. RXRA serves as a common heterodimeric partner for a number of nuclear receptors. The RXR/RAR heterodimers bind to the retinoic acid response [...]	0.909
SCARB1	ENSP00000261693	scavenger receptor class B, member 1; Receptor for different ligands such as phospholipids, cholesterol ester, lipoproteins, phosphatidylserine and apoptotic cells. Probable receptor for HDL, located in particular region of the plasma membrane, called caveolae. Facilitates the flux of free and esterified cholesterol between the cell surface and extracellular donors and acceptors, such as HDL and to a lesser extent, apoB-containing lipoproteins and modified lipoproteins. Probably involved in the phagocytosis of apoptotic cells, via its phosphatidylserine binding activity. Receptor for h [...]	0.506
SCARB2	ENSP00000264896	scavenger receptor class B, member 2; Acts as a lysosomal receptor for glucosylceramidase (GBA) targeting	0.408

	SCD	ENSP00000359380	stearoyl-CoA desaturase (delta-9-desaturase)	0.552
	SDC1	ENSP00000254351	syndecan 1; Cell surface proteoglycan that bears both heparan sulfate and chondroitin sulfate and that links the cytoskeleton to the interstitial matrix	0.9
	SDC2	ENSP00000307046	syndecan 2; Cell surface proteoglycan that bears heparan sulfate. Regulates dendritic arbor morphogenesis (By similarity)	0.9
	SDC3	ENSP00000344468	syndecan 3; Cell surface proteoglycan that may bear heparan sulfate (By similarity). May have a role in the organization of cell shape by affecting the actin cytoskeleton, possibly by transferring signals from the cell surface in a sugar-dependent mechanism	0.9
	SDC4	ENSP00000361818	syndecan 4; Cell surface proteoglycan that bears heparan sulfate	0.979
	SELE	ENSP00000331736	selectin E; Cell-surface glycoprotein having a role in immunoadhesion. Mediates in the adhesion of blood neutrophils in cytokine-activated endothelium through interaction with PSGL1/SELPLG. May have a role in capillary morphogenesis	0.822
	SERPINE1	ENSP00000223095	serpin peptidase inhibitor, clade E (nexin, plasminogen activator inhibitor type 1), member 1; Serine protease inhibitor. This inhibitor acts as 'bait' for tissue plasminogen activator, urokinase, protein C and matriptase-3/TMPRSS7. Its rapid interaction with PLAT may function as a major control point in the regulation of fibrinolysis	0.823
	SERPINE2	ENSP00000415786	serpin peptidase inhibitor, clade E (nexin, plasminogen activator inhibitor type 1), member 2; Serine protease inhibitor with activity toward thrombin, trypsin, and urokinase. Promotes neurite extension by inhibiting thrombin. Binds heparin	0.786
	SIN3A	ENSP00000353622	SIN3 transcription regulator homolog A (yeast); Acts as a transcriptional repressor. Corepressor for REST. Interacts with MXI1 to repress MYC responsive genes and antagonize MYC oncogenic activities. Also interacts with MXD1-MAX heterodimers to repress transcription by tethering SIN3A to DNA (By similarity). Acts cooperatively with OGT to repress transcription in parallel with histone deacetylation	0.9
	SLC27A1	ENSP00000252595	solute carrier family 27 (fatty acid transporter), member 1; Involved in translocation of long-chain fatty acids (LFCA) across the plasma membrane. The LFCA import appears to be hormone-regulated in a tissue-specific manner. In adipocytes, but not myocytes, insulin induces a rapid translocation of FATP1 from intracellular compartments to the plasma membrane, paralleled by increased LFCA uptake. May act directly as a bona fide transporter, or alternatively, in a cytoplasmic or membrane- associated multimeric protein complex to trap and draw fatty acids towards accumulation. Plays a pivo [...]	0.932
	SLC27A2	ENSP00000267842	solute carrier family 27 (fatty acid transporter), member 2; Acyl-CoA synthetase probably involved in bile acid metabolism. Proposed to activate C27 precurors of bile acids to their CoA thioesters derivatives before side chain cleavage via peroxisomal beta-oxidation occurs. In vitro, activates 3-alpha,7- alpha,12-alpha-trihydroxy-5-beta-cholestanate (THCA), the C27 precursor of cholic acid deriving from the de novo synthesis from cholesterol. Does not utilize C24 bile acids as substrates. In vitro, also activates long- and branched-chain fatty acids and may have additional roles in fat [...]	0.923
	SMARCD3	ENSP00000262188	SWI/SNF related, matrix associated, actin dependent regulator of chromatin, subfamily d, member 3; Plays a role in ATP dependent nucleosome remodeling by SMARCA4 containing complexes. Stimulates nuclear receptor mediated transcription. Belongs to the neural progenitors-specific chromatin remodeling complex (npBAF complex) and the neuron- specific chromatin remodeling complex (nBAF complex). During neural development a switch from a stem/progenitor to a post- mitotic chromatin remodeling mechanism occurs as neurons exit the cell cycle and become committed to their adult state. The trans [...]	0.9
	SP1	ENSP00000329357	Sp1 transcription factor; Transcription factor that can activate or repress transcription in response to physiological and pathological stimuli. Binds with high affinity to GC-rich motifs and regulates the expression of a large number of genes involved in a variety of processes such as cell growth, apoptosis, differentiation and immune responses. Highly regulated by post-translational modifications (phosphorylations, sumoylation, proteolytic cleavage, glycosylation and acetylation). Binds also the PDGFR- alpha G-box promoter. May have a role in modulating the cellular response to DNA d [...]	0.9
	SREBF2	ENSP00000354476	sterol regulatory element binding transcription factor 2; Transcriptional activator required for lipid homeostasis. Regulates transcription of the LDL receptor gene as well as the cholesterol and to a lesser degree the fatty acid synthesis pathway (By similarity). Binds the sterol regulatory element 1 (SRE-1) (5'-ATCACCCCAC-3') found in the flanking region of the LDRL and HMG-CoA synthase genes	0.909
	SULT2A1	ENSP00000222002	sulfotransferase family, cytosolic, 2A, dehydroepiandrosterone (DHEA)-preferring, member 1; Sulfotransferase that utilizes 3'-phospho-5'-adenylyl sulfate (PAPS) as sulfonate donor to catalyze the sulfonation of steroids and bile acids in the liver and adrenal glands	0.9
	TBL1X	ENSP00000217964	transducin (beta)-like 1X-linked; F-box-like protein involved in the recruitment of the ubiquitin/19S proteasome complex to nuclear receptor-regulated transcription units. Plays an essential role in transcription activation mediated by nuclear receptors. Probably acts as integral component of corepressor complexes that mediates the recruitment of the 19S proteasome complex, leading to the subsequent proteasomal degradation of transcription repressor complexes, thereby allowing cofactor exchange	0.9
	TBL1XR1	ENSP00000405574	transducin (beta)-like 1 X-linked receptor 1; F-box-like protein involved in the recruitment of the ubiquitin/19S proteasome complex to nuclear receptor-regulated transcription units. Plays an essential role in transcription activation mediated by nuclear receptors. Probably acts as integral component of the N-Cor corepressor complex that mediates the recruitment of the 19S proteasome complex, leading to the subsequent proteasomal degradation of N-Cor complex, thereby allowing cofactor exchange, and transcription activation	0.9
	TEAD1	ENSP00000354588	TEA domain family member 1 (SV40 transcriptional enhancer factor); Transcription factor which plays a key role in the Hippo signaling pathway, a pathway involved in organ size control and tumor suppression by restricting proliferation and promoting apoptosis. The core of this pathway is composed of a kinase cascade wherein MST1/MST2, in complex with its regulatory protein SAV1, phosphorylates and activates LATS1/2 in complex with its regulatory protein MOB1, which in turn phosphorylates and inactivates YAP1 oncoprotein and WWTR1/TAZ. Acts by mediating gene expression of YAP1 and WWTR1/ [...]	0.9
	TEAD2	ENSP00000310701	TEA domain family member 2; Transcription factor which plays a key role in the Hippo signaling pathway, a pathway involved in organ size control and tumor suppression by restricting proliferation and promoting apoptosis. The core of this pathway is composed of a kinase cascade wherein MST1/MST2, in complex with its regulatory protein SAV1, phosphorylates and activates LATS1/2 in complex with its regulatory protein MOB1, which in turn phosphorylates and inactivates YAP1 oncoprotein and WWTR1/TAZ. Acts by mediating gene expression of YAP1 and WWTR1/TAZ, thereby regulating cell proliferat [...]	0.9
	TEAD3	ENSP00000345772	TEA domain family member 3; Transcription factor which plays a key role in the Hippo signaling pathway, a pathway involved in organ size control and tumor suppression by restricting proliferation and promoting apoptosis. The core of this pathway is composed of a kinase cascade wherein MST1/MST2, in complex with its regulatory protein SAV1, phosphorylates and activates LATS1/2 in complex with its regulatory protein MOB1, which in turn phosphorylates and inactivates YAP1 oncoprotein and WWTR1/TAZ. Acts by mediating gene expression of YAP1 and WWTR1/TAZ, thereby regulating cell proliferat [...]	0.9
	TEAD4	ENSP00000352926	TEA domain family member 4; Transcription factor which plays a key role in the Hippo signaling pathway, a pathway involved in organ size control and tumor suppression by restricting proliferation and promoting apoptosis. The core of this pathway is composed of a kinase cascade wherein MST1/MST2, in complex with its regulatory protein SAV1, phosphorylates and activates LATS1/2 in complex with its regulatory protein MOB1, which in turn phosphorylates and inactivates YAP1 oncoprotein and WWTR1/TAZ. Acts by mediating gene expression of YAP1 and WWTR1/TAZ, thereby regulating cell proliferat [...]	0.9
	TGS1	ENSP00000260129	trimethylguanosine synthase 1; Catalyzes the 2 serial methylation steps for the conversion of the 7-monomethylguanosine (m(7)G) caps of snRNAs and snoRNAs to a 2,2,7-trimethylguanosine (m(2,2,7)G) cap structure. The enzyme is specific for guanine, and N7 methylation must precede N2 methylation. Hypermethylation of the m7G cap of U snRNAs leads to their concentration in nuclear foci, their colocalization with coilin and the formation of canonical Cajal bodies (CBs). Plays a role in transcriptional regulation	0.9
	TIAM2	ENSP00000327315	T-cell lymphoma invasion and metastasis 2; Modulates the activity of RHO-like proteins and connects extracellular signals to cytoskeletal activities. Acts as a GDP- dissociation stimulator protein that stimulates the GDP-GTP exchange activity of RHO-like GTPases and activates them. Mediates extracellular laminin signals to activate Rac1, contributing to neurite growth. Involved in lamellipodial formation and advancement of the growth cone of embryonic hippocampal neurons. Promotes migration of neurons in the cerebral cortex. When overexpressed, induces membrane ruffling accompanied by [...]	0.9
	TNF	ENSP00000398698	tumor necrosis factor	0.848
	TNFRSF21	ENSP00000296861	tumor necrosis factor receptor superfamily, member 21; May activate NF-kappa-B and promote apoptosis. May activate JNK and be involved in T-cell differentiation. Required for both normal cell body death and axonal pruning. Trophic-factor deprivation triggers the cleavage of surface APP by beta-secretase to release sAPP-beta which is further cleaved to release an N- terminal fragment of APP (N-APP). N-APP binds TNFRSF21 triggering caspase activation and degeneration of both neuronal cell bodies (via caspase-3) and axons (via caspase-6)	0.9
	TRIB3	ENSP00000217233	tribbles homolog 3 (Drosophila); Disrupts insulin signaling by binding directly to Akt kinases and blocking their activation. May bind directly to and mask the 'Thr-308' phosphorylation site in AKT1. Binds to ATF4 and inhibits its transcriptional activation activity. Interacts with the NF-kappa-B transactivator p65 RELA and inhibits its phosphorylation and thus its transcriptional activation activity. Interacts with MAPK kinases and regulates activation of MAP kinases. May play a role in programmed neuronal cell death but does not appear to affect non-neuronal cells. Does not display k [...]	0.9
	TXNRD1	ENSP00000434516	thioredoxin reductase 1	0.9
	TYR	ENSP00000263321	tyrosinase; This is a copper-containing oxidase that functions in the formation of pigments such as melanins and other polyphenolic compounds. Catalyzes the rate-limiting conversions of tyrosine to DOPA, DOPA to DOPA-quinone and possibly 5,6-dihydroxyindole to indole-5,6 quinone	0.842
	UGT1A1	ENSP00000304845	UDP glucuronosyltransferase 1 family, polypeptide A1; UDPGT is of major importance in the conjugation and subsequent elimination of potentially toxic xenobiotics and endogenous compounds. This isoform glucuronidates bilirubin IX- alpha to form both the IX-alpha-C8 and IX-alpha-C12 monoconjugates and diconjugate. Is also able to catalyze the glucuronidation of 17beta-estradiol, 17alpha-ethinylestradiol, 1-hydroxypyrene, 4- methylumbelliferone, 1-naphthol, paranitrophenol, scopoletin, and umbelliferone	0.8
	UGT1A9	ENSP00000346768	UDP glucuronosyltransferase 1 family, polypeptide A9; UDPGT is of major importance in the conjugation and subsequent elimination of potentially toxic xenobiotics and endogenous compounds. This isoform has specificity for phenols	0.9
	VCAM1	ENSP00000294728	vascular cell adhesion molecule 1; Important in cell-cell recognition. Appears to function in leukocyte-endothelial cell adhesion. Interacts with the beta-1 integrin VLA4 on leukocytes, and mediates both adhesion and signal transduction. The VCAM1/VLA4 interaction may play a pathophysiologic role both in immune responses and in leukocyte emigration to sites of inflammation	0.828
	WWTR1	ENSP00000353847	WW domain containing transcription regulator 1; Transcriptional coactivator which acts as a downstream regulatory target in the Hippo signaling pathway that plays a pivotal role in organ size control and tumor suppression by restricting proliferation and promoting apoptosis. The core of this pathway is composed of a kinase cascade wherein STK3/MST2 and STK4/MST1, in complex with its regulatory protein SAV1, phosphorylates and activates LATS1/2 in complex with its regulatory protein MOB1, which in turn phosphorylates and inactivates YAP1 oncoprotein and WWTR1/TAZ. WWTR1 enhances PAX8 an [...]	0.9
	YAP1	ENSP00000282441	Yes-associated protein 1; Transcriptional regulator which can act both as a coactivator and a corepressor and is the critical downstream regulatory target in the Hippo signaling pathway that plays a pivotal role in organ size control and tumor suppression by restricting proliferation and promoting apoptosis. The core of this pathway is composed of a kinase cascade wherein STK3/MST2 and STK4/MST1, in complex with its regulatory protein SAV1, phosphorylates and activates LATS1/2 in complex with its regulatory protein MOB1, which in turn phosphorylates and inactivates YAP1 oncoprotein and [...]	0.9
**node1**	**Linolenic acid**	**5280934**	**Linolenic acid**	
**node2**	ABCA1	ENSP00000363868	ATP-binding cassette, sub-family A (ABC1), member 1	0.908

ABCB4	ENSP00000265723	ATP-binding cassette, sub-family B (MDR/TAP), member 4; Mediates ATP-dependent export of organic anions and drugs from the cytoplasm. Hydrolyzes ATP with low efficiency. Not capable of conferring drug resistance. Mediates the translocation of phosphatidylcholine across the canalicular membrane of the hepatocyte	0.91
ACADM	ENSP00000409612	acyl-CoA dehydrogenase, C-4 to C-12 straight chain; This enzyme is specific for acyl chain lengths of 4 to 16	0.9
ACOT1	ENSP00000311224	acyl-CoA thioesterase 1; Acyl-CoA thioesterases are a group of enzymes that catalyze the hydrolysis of acyl-CoAs to the free fatty acid and coenzyme A (CoASH), providing the potential to regulate intracellular levels of acyl-CoAs, free fatty acids and CoASH. Active towards fatty acyl-CoA with chain-lengths of C12-C16 (By similarity)	0.816
ACOT2	ENSP00000238651	acyl-CoA thioesterase 2; Acyl-CoA thioesterases are a group of enzymes that catalyze the hydrolysis of acyl-CoAs to the free fatty acid and coenzyme A (CoASH), providing the potential to regulate intracellular levels of acyl-CoAs, free fatty acids and CoASH. Displays high levels of activity on medium- and long chain acyl CoAs	0.816
ACOT4	ENSP00000323071	acyl-CoA thioesterase 4; Acyl-CoA thioesterases are a group of enzymes that catalyze the hydrolysis of acyl-CoAs to the free fatty acid and coenzyme A (CoASH), providing the potential to regulate intracellular levels of acyl-CoAs, free fatty acids and CoASH (By similarity). Succinyl-CoA thioesterase that also hydrolyzes long chain saturated and unsaturated monocarboxylic acyl-CoAs	0.816
ACOT7	ENSP00000367086	acyl-CoA thioesterase 7	0.8
ACSL1	ENSP00000281455	acyl-CoA synthetase long-chain family member 1; Activation of long-chain fatty acids for both synthesis of cellular lipids, and degradation via beta-oxidation. Preferentially uses palmitoleate, oleate and linoleate	0.909
ACSL3	ENSP00000350012	acyl-CoA synthetase long-chain family member 3; Acyl-CoA synthetases (ACSL) activates long-chain fatty acids for both synthesis of cellular lipids, and degradation via beta-oxidation. ACSL3 mediates hepatic lipogenesis (By similarity). Preferentially uses myristate, laurate, arachidonate and eicosapentaenoate as substrates (By similarity). Has mainly an anabolic role in energy metabolism. Required for the incorporation of fatty acids into phosphatidylcholine, the major phospholipid located on the surface of VLDL (very low density lipoproteins)	0.9
ACSL4	ENSP00000339787	acyl-CoA synthetase long-chain family member 4; Activation of long-chain fatty acids for both synthesis of cellular lipids, and degradation via beta-oxidation. Preferentially uses arachidonate and eicosapentaenoate as substrates	0.9
ACSL5	ENSP00000348429	acyl-CoA synthetase long-chain family member 5; Acyl-CoA synthetases (ACSL) activate long-chain fatty acids for both synthesis of cellular lipids, and degradation via beta-oxidation. ACSL5 may activate fatty acids from exogenous sources for the synthesis of triacylglycerol destined for intracellular storage (By similarity). Utilizes a wide range of saturated fatty acids with a preference for C16-C18 unsaturated fatty acids (By similarity). It was suggested that it may also stimulate fatty acid oxidation (By similarity). At the villus tip of the crypt-villus axis of the small intestine [...]	0.9
ACSL6	ENSP00000296869	acyl-CoA synthetase long-chain family member 6; Activation of long-chain fatty acids for both synthesis of cellular lipids, and degradation via beta-oxidation. Plays an important role in fatty acid metabolism in brain and the acyl-CoAs produced may be utilized exclusively for the synthesis of the brain lipid	0.9
ACSM1	ENSP00000301956	acyl-CoA synthetase medium-chain family member 1; Has medium-chain fatty acid-CoA ligase activity with broad substrate specificity (in vitro). Acts on acids from C(4) to C(11) and on the corresponding 3-hydroxy- and 2,3- or 3,4- unsaturated acids (in vitro). Functions as GTP-dependent lipoate- activating enzyme that generates the substrate for lipoyltransferase (By similarity)	0.907
ACSM3	ENSP00000289416	acyl-CoA synthetase medium-chain family member 3; Has medium-chain fatty acid-CoA ligase activity with broad substrate specificity (in vitro). Acts on acids from C(4) to C(11) and on the corresponding 3-hydroxy- and 2,3- or 3,4- unsaturated acids (in vitro) (By similarity)	0.907
ACSM4	ENSP00000382349	acyl-CoA synthetase medium-chain family member 4; Has medium-chain fatty acid-CoA ligase activity with broad substrate specificity (in vitro). Acts on acids from C(4) to C(11) and on the corresponding 3-hydroxy- and 2,3- or 3,4- unsaturated acids (in vitro) (By similarity)	0.907
ACSM5	ENSP00000327916	acyl-CoA synthetase medium-chain family member 5; Has medium-chain fatty acid-CoA ligase activity with broad substrate specificity (in vitro). Acts on acids from C(4) to C(11) and on the corresponding 3-hydroxy- and 2,3- or 3,4- unsaturated acids (in vitro) (By similarity)	0.907
AGT	ENSP00000355627	angiotensinogen (serpin peptidase inhibitor, clade A, member 8); Essential component of the renin-angiotensin system (RAS), a potent regulator of blood pressure, body fluid and electrolyte homeostasis	0.9
ALAS1	ENSP00000309259	aminolevulinate, delta-, synthase 1	0.9
ALOX12	ENSP00000251535	arachidonate 12-lipoxygenase; Oxygenase and 14,15-leukotriene A4 synthase activity	0.406
ANGPTL4	ENSP00000301455	angiopoietin-like 4; Protein with hypoxia-induced expression in endothelial cells. May act as a regulator of angiogenesis and modulate tumorigenesis. Inhibits proliferation, migration, and tubule formation of endothelial cells and reduces vascular leakage. May exert a protective function on endothelial cells through an endocrine action. It is directly involved in regulating glucose homeostasis, lipid metabolism, and insulin sensitivity. In response to hypoxia, the unprocessed form of the protein accumulates in the subendothelial extracellular matrix (ECM). The matrix-associated and imm [...]	0.9
ANKRD1	ENSP00000360762	ankyrin repeat domain 1 (cardiac muscle); May play an important role in endothelial cell activation. May act as a nuclear transcription factor that negatively regulates the expression of cardiac genes. Induction seems to be correlated with apoptotic cell death in hepatoma cells	0.9
APOA1	ENSP00000236850	apolipoprotein A-I; Participates in the reverse transport of cholesterol from tissues to the liver for excretion by promoting cholesterol efflux from tissues and by acting as a cofactor for the lecithin cholesterol acyltransferase (LCAT). As part of the SPAP complex, activates spermatozoa motility	0.912
APOA2	ENSP00000356969	apolipoprotein A-II; May stabilize HDL (high density lipoprotein) structure by its association with lipids, and affect the HDL metabolism	0.9
APOA5	ENSP00000227665	apolipoprotein A-V; Minor apolipoprotein mainly associated with HDL and to a lesser extent with VLDL. May also be associated with chylomicrons. Important determinant of plasma triglyceride (TG) levels by both being a potent stimulator of apo-CII lipoprotein lipase (LPL) TG hydrolysis and a inhibitor of the hepatic VLDL-TG production rate (without affecting the VLDL-apoB production rate) (By similarity). Activates poorly lecithin-cholesterol acyltransferase (LCAT) and does not enhance efflux of cholesterol from macrophages	0.9
APOB	ENSP00000233242	apolipoprotein B (including Ag(x) antigen)	0.833
ARNTL	ENSP00000374357	aryl hydrocarbon receptor nuclear translocator-like	0.9
BAAT	ENSP00000259407	bile acid CoA- amino acid N-acyltransferase (glycine N-choloyltransferase); Involved in bile acid metabolism. In liver hepatocytes catalyzes the second step in the conjugation of C24 bile acids (choloneates) to glycine and taurine before excretion into bile canaliculi. The major components of bile are cholic acid and chenodeoxycholic acid. In a first step the bile acids are converted to an acyl-CoA thioester, either in peroxisomes (primary bile acids deriving from the cholesterol pathway), or cytoplasmic at the endoplasmic reticulum (secondary bile acids). May catalyze the conjugation [...]	0.816
C10orf129	ENSP00000340296	chromosome 10 open reading frame 129	0.9
CARM1	ENSP00000325690	coactivator-associated arginine methyltransferase 1; Methylates (mono- and asymmetric dimethylation) the guanidino nitrogens of arginyl residues in several proteins involved in DNA packaging, transcription regulation, pre-mRNA splicing, and mRNA stability. Recruited to promoters upon gene activation together with histone acetyltransferases from EP300/P300 and p160 families, methylates histone H3 at 'Arg-17' (H3R17me), forming mainly asymmetric dimethylarginine (H3R17me2a), leading to activate transcription via chromatin remodeling. During nuclear hormone receptor activation and TCF7L2/ [...]	0.9
CCK	ENSP00000335657	cholecystokinin; This peptide hormone induces gall bladder contraction and the release of pancreatic enzymes in the gut. Its function in the brain is not clear. Binding to CCK-A receptors stimulates amylase release from the pancreas, binding to CCK-B receptors stimulates gastric acid secretion	0.786
CCNC	ENSP00000428982	cyclin C; Component of the Mediator complex, a coactivator involved in regulated gene transcription of nearly all RNA polymerase II-dependent genes. Mediator functions as a bridge to convey information from gene-specific regulatory proteins to the basal RNA polymerase II transcription machinery. Mediator is recruited to promoters by direct interactions with regulatory proteins and serves as a scaffold for the assembly of a functional preinitiation complex with RNA polymerase II and the general transcription factors. Binds to and activates cyclin-dependent kinase CDK8 that phosphorylate [...]	0.9
CD36	ENSP00000308165	CD36 molecule (thrombospondin receptor)	0.925
CDK19	ENSP00000357907	cyclin-dependent kinase 19	0.9
CDK8	ENSP00000370938	cyclin-dependent kinase 8; Component of the Mediator complex, a coactivator involved in regulated gene transcription of nearly all RNA polymerase II-dependent genes. Mediator functions as a bridge to convey information from gene-specific regulatory proteins to the basal RNA polymerase II transcription machinery. Mediator is recruited to promoters by direct interactions with regulatory proteins and serves as a scaffold for the assembly of a functional preinitiation complex with RNA polymerase II and the general transcription factors. Phosphorylates the CTD (C-terminal domain) of the lar [...]	0.9
CHD9	ENSP00000455307	chromodomain helicase DNA binding protein 9; Acts as a transcriptional coactivator for PPARA and possibly other nuclear receptors. Proposed to be a ATP-dependent chromatin remodeling protein. Has DNA-dependent ATPase activity and binds to A/T-rich DNA. Associates with A/T-rich regulatory regions in promoters of genes that participate in the differentiation of progenitors during osteogenesis (By similarity)	0.9
CLOCK	ENSP00000308741	clock homolog (mouse); ARNTL/2-CLOCK heterodimers activate E-box element (5'- CACGTG-3') transcription of a number of proteins of the circadian clock. Activates transcription of PER1 and PER2. This transcription is inhibited in a feedback loop by PER and CRY proteins. Has intrinsic histone acetyltransferase activity and this enzymatic function contributes to chromatin-remodeling events implicated in circadian control of gene expression (By similarity). Acetylates primarily histones H3 and H4 (By similarity). Acetylates also a non-histone substrate- ARNTL (By similarity). Plays a role i [...]	0.9
CPT1A	ENSP00000265641	carnitine palmitoyltransferase 1A (liver); Catalyzes the transfer of the acyl group of long-chain fatty acid-CoA conjugates onto carnitine, an essential step for the mitochondrial uptake of long-chain fatty acids and their subsequent beta-oxidation in the mitochondrion. Plays an important role in triglyceride metabolism	0.909
CPT2	ENSP00000360541	carnitine palmitoyltransferase 2	0.9
CREBBP	ENSP00000262367	CREB binding protein; Acetylates histones, giving a specific tag for transcriptional activation. Also acetylates non-histone proteins, like NCOA3 and FOXO1. Binds specifically to phosphorylated CREB and enhances its transcriptional activity toward cAMP-responsive genes. Acts as a coactivator of ALX1 in the presence of EP300	0.9

CRP	ENSP00000255030	C-reactive protein, pentraxin-related; Displays several functions associated with host defense- it promotes agglutination, bacterial capsular swelling, phagocytosis and complement fixation through its calcium-dependent binding to phosphorylcholine. Can interact with DNA and histones and may scavenge nuclear material released from damaged circulating cells	0.842
CTGF	ENSP00000356954	connective tissue growth factor; Major connective tissue mitoattractant secreted by vascular endothelial cells. Promotes proliferation and differentiation of chondrocytes. Mediates heparin- and divalent cation-dependent cell adhesion in many cell types including fibroblasts, myofibroblasts, endothelial and epithelial cells. Enhances fibroblast growth factor-induced DNA synthesis	0.9
CYP1A1	ENSP00000369050	cytochrome P450, family 1, subfamily A, polypeptide 1; Cytochromes P450 are a group of heme-thiolate monooxygenases. In liver microsomes, this enzyme is involved in an NADPH-dependent electron transport pathway. It oxidizes a variety of structurally unrelated compounds, including steroids, fatty acids, and xenobiotics	0.9
CYP1A2	ENSP00000342007	cytochrome P450, family 1, subfamily A, polypeptide 2; Cytochromes P450 are a group of heme-thiolate monooxygenases. In liver microsomes, this enzyme is involved in an NADPH-dependent electron transport pathway. It oxidizes a variety of structurally unrelated compounds, including steroids, fatty acids, and xenobiotics. Most active in catalyzing 2-hydroxylation. Caffeine is metabolized primarily by cytochrome CYP1A2 in the liver through an initial N3-demethylation. Also acts in the metabolism of aflatoxin B1 and acetaminophen. Participates in the bioactivation of carcinogenic aromatic a [...]	0.7
CYP2C19	ENSP00000360372	cytochrome P450, family 2, subfamily C, polypeptide 19; Responsible for the metabolism of a number of therapeutic agents such as the anticonvulsant drug S-mephenytoin, omeprazole, proguanil, certain barbiturates, diazepam, propranolol, citalopram and imipramine	0.7
CYP2C9	ENSP00000260682	cytochrome P450, family 2, subfamily C, polypeptide 9; Cytochromes P450 are a group of heme-thiolate monooxygenases. In liver microsomes, this enzyme is involved in an NADPH-dependent electron transport pathway. It oxidizes a variety of structurally unrelated compounds, including steroids, fatty acids, and xenobiotics. This enzyme contributes to the wide pharmacokinetics variability of the metabolism of drugs such as S- warfarin, diclofenac, phenytoin, tolbutamide and losartan	0.7
CYP2E1	ENSP00000252945	cytochrome P450, family 2, subfamily E, polypeptide 1; Metabolizes several precarcinogens, drugs, and solvents to reactive metabolites. Inactivates a number of drugs and xenobiotics and also bioactivates many xenobiotic substrates to their hepatotoxic or carcinogenic forms	0.7
CYP3A4	ENSP00000337915	cytochrome P450, family 3, subfamily A, polypeptide 4; Cytochromes P450 are a group of heme-thiolate monooxygenases. In liver microsomes, this enzyme is involved in an NADPH-dependent electron transport pathway. It performs a variety of oxidation reactions (e.g. caffeine 8-oxidation, omeprazole sulphoxidation, midazolam 1'-hydroxylation and midazolam 4- hydroxylation) of structurally unrelated compounds, including steroids, fatty acids, and xenobiotics. Acts as a 1,8-cineole 2- exo-monooxygenase. The enzyme also hydroxylates etoposide	0.7
CYP4A11	ENSP00000311095	cytochrome P450, family 4, subfamily A, polypeptide 11; Catalyzes the omega- and (omega-1)-hydroxylation of various fatty acids such as laurate, myristate and palmitate. Has little activity toward prostaglandins A1 and E1. Oxidizes arachidonic acid to 20-hydroxyeicosatetraenoic acid (20-HETE)	0.9
CYP7A1	ENSP00000301645	cytochrome P450, family 7, subfamily A, polypeptide 1; Catalyzes a rate-limiting step in cholesterol catabolism and bile acid biosynthesis by introducing a hydrophilic moiety at position 7 of cholesterol. Important for cholesterol homeostasis	0.909
ELOVL4	ENSP00000358831	ELOVL fatty acid elongase 4; Condensing enzyme that elongates saturated and monounsaturated very long chain fatty acids (VLCFAs). Elongates C24-0 and C26-0 acyl-CoAs. Seems to represent a photoreceptor- specific component of the fatty acid elongation system residing on the endoplasmic reticulum. May be implicated in docosahexaenoic acid (DHA) biosynthesis, which requires dietary consumption of the essential alpha-Linolenic acid and a subsequent series of three elongation steps. May play a critical role in early brain and skin development	0.819
ENSG00000168970	ENSP00000371886	JMJD7-PLA2G4B readthrough	0.9
EP300	ENSP00000263253	E1A binding protein p300; Functions as histone acetyltransferase and regulates transcription via chromatin remodeling. Acetylates all four core histones in nucleosomes. Histone acetylation gives an epigenetic tag for transcriptional activation. Mediates cAMP-gene regulation by binding specifically to phosphorylated CREB protein. Also functions as acetyltransferase for nonhistone targets. Acetylates 'Lys-131' of ALX1 and acts as its coactivator in the presence of CREBBP. Acetylates SIRT2 and is proposed to indirectly increase the transcriptional activity of TP53 through acetylation and [...]	0.9
EPX	ENSP00000225371	eosinophil peroxidase; Mediates tyrosine nitration of secondary granule proteins in mature resting eosinophils. Shows significant inhibitory activity towards Mycobacterium tuberculosis H37Rv by inducing bacterial fragmentation and lysis	0.57
FA2H	ENSP00000219368	fatty acid 2-hydroxylase; Required for alpha-hydroxylation of free fatty acids and the formation of alpha-hydroxylated sphingolipids	0.472
FABP1	ENSP00000295834	fatty acid binding protein 1, liver; Binds free fatty acids and their coenzyme A derivatives, bilirubin, and some other small molecules in the cytoplasm. May be involved in intracellular lipid transport	0.907
FABP4	ENSP00000256104	fatty acid binding protein 4, adipocyte; Lipid transport protein in adipocytes. Binds both long chain fatty acids and retinoic acid. Delivers long-chain fatty acids and retinoic acid to their cognate receptors in the nucleus (By similarity)	0.91
FADS1	ENSP00000322229	fatty acid desaturase 1	0.947
FADS2	ENSP00000278840	fatty acid desaturase 2; Component of a lipid metabolic pathway that catalyzes biosynthesis of highly unsaturated fatty acids (HUFA) from precursor essential polyunsaturated fatty acids (PUFA) Linoleic acid (LA) (18-2n-6) and alpha-Linolenic acid (ALA) (18-3n-3). Catalyzes the first and rate limiting step in this pathway which is the desaturation of LA (18-2n-6) and ALA (18-3n-3) into gamma- Linoleic acid (GLA) (18-3n-6) and stearidonic acid (18-4n-3) respectively and other desaturation steps. Highly unsaturated fatty acids (HUFA) play pivotal roles in many biological functions. It cat [...]	0.957
FDFT1	ENSP00000220584	farnesyl-diphosphate farnesyltransferase 1	0.907
FHL2	ENSP00000322909	four and a half LIM domains 2; May function as a molecular transmitter linking various signaling pathways to transcriptional regulation. Negatively regulates the transcriptional repressor E4F1 and may function in cell growth. Inhibits the transcriptional activity of FOXO1 and its apoptotic function by enhancing the interaction of FOXO1 with SIRT1 and FOXO1 deacetylation	0.911
FLAD1	ENSP00000292180	FAD1 flavin adenine dinucleotide synthetase homolog (S. cerevisiae); Catalyzes the adenylation of flavin mononucleotide (FMN) to form flavin adenine dinucleotide (FAD) coenzyme	0.441
G0S2	ENSP00000355996	G0/G1switch 2; Promotes apoptosis by binding to BCL2, hence preventing the formation of protective BCL2-BAX heterodimers	0.9
GCG	ENSP00000387662	glucagon; Glicentin may modulate gastric acid secretion and the gastro-pyloro-duodenal activity. May play an important role in intestinal mucosal growth in the early period of life	0.911
GCLC	ENSP00000229416	glutamate-cysteine ligase, catalytic subunit	0.7
GLIPR1	ENSP00000266659	GLI pathogenesis-related 1	0.9
GRHL1	ENSP00000324693	grainyhead-like 1 (Drosophila)	0.9
HDAC3	ENSP00000302967	histone deacetylase 3; Responsible for the deacetylation of lysine residues on the N-terminal part of the core histones (H2A, H2B, H3 and H4), and some other non-histone substrates. Histone deacetylation gives a tag for epigenetic repression and plays an important role in transcriptional regulation, cell cycle progression and developmental events. Histone deacetylases act via the formation of large multiprotein complexes. Probably participates in the regulation of transcription through its binding to the zinc-finger transcription factor YY1; increases YY1 repression activity. Required [...]	0.9
HELZ2	ENSP00000417401	helicase with zinc finger 2, transcriptional coactivator; Helicase that acts as a transcriptional coactivator for a number of nuclear receptors including PPARA, PPARG, THRA, THRB and RXRA	0.9
HIF1A	ENSP00000338018	hypoxia inducible factor 1, alpha subunit (basic helix-loop-helix transcription factor); Functions as a master transcriptional regulator of the adaptive response to hypoxia. Under hypoxic conditions, activates the transcription of over 40 genes, including erythropoietin, glucose transporters, glycolytic enzymes, vascular endothelial growth factor, HILPDA, and other genes whose protein products increase oxygen delivery or facilitate metabolic adaptation to hypoxia. Plays an essential role in embryonic vascularization, tumor angiogenesis and pathophysiology of ischemic disease. Binds to [...]	0.9
HMGCR	ENSP00000287936	3-hydroxy-3-methylglutaryl-CoA reductase; Transmembrane glycoprotein that is the rate-limiting enzyme in cholesterol biosynthesis as well as in the biosynthesis of nonsterol isoprenoids that are essential for normal cell function including ubiquinone and geranylgeranyl proteins	0.909
HMGCS1	ENSP00000322706	3-hydroxy-3-methylglutaryl-CoA synthase 1 (soluble); This enzyme condenses acetyl-CoA with acetoacetyl-CoA to form HMG-CoA, which is the substrate for HMG-CoA reductase	0.9
HMGCS2	ENSP00000358414	3-hydroxy-3-methylglutaryl-CoA synthase 2 (mitochondrial); This enzyme condenses acetyl-CoA with acetoacetyl-CoA to form HMG-CoA, which is the substrate for HMG-CoA reductase	0.9
KCNK2	ENSP00000394033	potassium channel, subfamily K, member 2	0.589
LALBA	ENSP00000301046	lactalbumin, alpha-; Regulatory subunit of lactose synthase, changes the substrate specificity of galactosyltransferase in the mammary gland making glucose a good acceptor substrate for this enzyme. This enables LS to synthesize lactose, the major carbohydrate component of milk. In other tissues, galactosyltransferase transfers galactose onto the N-acetylglucosamine of the oligosaccharide chains in glycoproteins	0.516
LPO	ENSP00000262290	lactoperoxidase; May contribute to airway host defense against infection	0.57
ME1	ENSP00000358719	malic enzyme 1, NADP(+)-dependent, cytosolic	0.911
MED1	ENSP00000300651	mediator complex subunit 1; Component of the Mediator complex, a coactivator involved in the regulated transcription of nearly all RNA polymerase II-dependent genes. Mediator functions as a bridge to convey information from gene-specific regulatory proteins to the basal RNA polymerase II transcription machinery. Mediator is recruited to promoters by direct interactions with regulatory proteins and serves as a scaffold for the assembly of a functional preinitiation complex with RNA polymerase II and the general transcription factors	0.9
MED10	ENSP00000255764	mediator complex subunit 10; Component of the Mediator complex, a coactivator involved in the regulated transcription of nearly all RNA polymerase II-dependent genes. Mediator functions as a bridge to convey information from gene-specific regulatory proteins to the basal RNA polymerase II transcription machinery. Mediator is recruited to promoters by direct interactions with regulatory proteins and serves as a scaffold for the assembly of a functional preinitiation complex with RNA polymerase II and the general transcription factors	0.9
MED11	ENSP00000293777	mediator complex subunit 11; Component of the Mediator complex, a coactivator involved in the regulated transcription of nearly all RNA polymerase II-dependent genes. Mediator functions as a bridge to convey information from gene-specific regulatory proteins to the basal RNA polymerase II transcription machinery. Mediator is recruited to promoters by direct interactions with regulatory proteins and serves as a scaffold for the assembly of a functional preinitiation complex with RNA polymerase II and the general transcription factors	0.9

MED12	ENSP00000363193	mediator complex subunit 12; Component of the Mediator complex, a coactivator involved in the regulated transcription of nearly all RNA polymerase II-dependent genes. Mediator functions as a bridge to convey information from gene-specific regulatory proteins to the basal RNA polymerase II transcription machinery. Mediator is recruited to promoters by direct interactions with regulatory proteins and serves as a scaffold for the assembly of a functional preinitiation complex with RNA polymerase II and the general transcription factors. This subunit may specifically regulate transcription [...]	0.9
MED13	ENSP00000380888	mediator complex subunit 13; Component of the Mediator complex, a coactivator involved in the regulated transcription of nearly all RNA polymerase II-dependent genes. Mediator functions as a bridge to convey information from gene-specific regulatory proteins to the basal RNA polymerase II transcription machinery. Mediator is recruited to promoters by direct interactions with regulatory proteins and serves as a scaffold for the assembly of a functional preinitiation complex with RNA polymerase II and the general transcription factors	0.9
MED13L	ENSP00000281928	mediator complex subunit 13-like; Component of the Mediator complex, a coactivator involved in the regulated transcription of nearly all RNA polymerase II-dependent genes. Mediator functions as a bridge to convey information from gene-specific regulatory proteins to the basal RNA polymerase II transcription machinery. Mediator is recruited to promoters by direct interactions with regulatory proteins and serves as a scaffold for the assembly of a functional preinitiation complex with RNA polymerase II and the general transcription factors. This subunit may specifically regulate transcri [...]	0.9
MED14	ENSP00000323720	mediator complex subunit 14; Component of the Mediator complex, a coactivator involved in the regulated transcription of nearly all RNA polymerase II-dependent genes. Mediator functions as a bridge to convey information from gene-specific regulatory proteins to the basal RNA polymerase II transcription machinery. Mediator is recruited to promoters by direct interactions with regulatory proteins and serves as a scaffold for the assembly of a functional preinitiation complex with RNA polymerase II and the general transcription factors	0.9
MED15	ENSP00000263205	mediator complex subunit 15	0.9
MED16	ENSP00000325612	mediator complex subunit 16; Component of the Mediator complex, a coactivator involved in the regulated transcription of nearly all RNA polymerase II-dependent genes. Mediator functions as a bridge to convey information from gene-specific regulatory proteins to the basal RNA polymerase II transcription machinery. Mediator is recruited to promoters by direct interactions with regulatory proteins and serves as a scaffold for the assembly of a functional preinitiation complex with RNA polymerase II and the general transcription factors	0.9
MED17	ENSP00000251871	mediator complex subunit 17; Component of the Mediator complex, a coactivator involved in the regulated transcription of nearly all RNA polymerase II-dependent genes. Mediator functions as a bridge to convey information from gene-specific regulatory proteins to the basal RNA polymerase II transcription machinery. Mediator is recruited to promoters by direct interactions with regulatory proteins and serves as a scaffold for the assembly of a functional preinitiation complex with RNA polymerase II and the general transcription factors	0.9
MED18	ENSP00000362948	mediator complex subunit 18; Component of the Mediator complex, a coactivator involved in the regulated transcription of nearly all RNA polymerase II-dependent genes. Mediator functions as a bridge to convey information from gene-specific regulatory proteins to the basal RNA polymerase II transcription machinery. Mediator is recruited to promoters by direct interactions with regulatory proteins and serves as a scaffold for the assembly of a functional preinitiation complex with RNA polymerase II and the general transcription factors	0.9
MED19	ENSP00000337340	mediator complex subunit 19; Component of the Mediator complex, a coactivator involved in the regulated transcription of nearly all RNA polymerase II-dependent genes. Mediator functions as a bridge to convey information from gene-specific regulatory proteins to the basal RNA polymerase II transcription machinery. Mediator is recruited to promoters by direct interactions with regulatory proteins and serves as a scaffold for the assembly of a functional preinitiation complex with RNA polymerase II and the general transcription factors	0.9
MED20	ENSP00000265350	mediator complex subunit 20; Component of the Mediator complex, a coactivator involved in the regulated transcription of nearly all RNA polymerase II-dependent genes. Mediator functions as a bridge to convey information from gene-specific regulatory proteins to the basal RNA polymerase II transcription machinery. Mediator is recruited to promoters by direct interactions with regulatory proteins and serves as a scaffold for the assembly of a functional preinitiation complex with RNA polymerase II and the general transcription factors	0.9
MED21	ENSP00000282892	mediator complex subunit 21; Component of the Mediator complex, a coactivator involved in the regulated transcription of nearly all RNA polymerase II-dependent genes. Mediator functions as a bridge to convey information from gene-specific regulatory proteins to the basal RNA polymerase II transcription machinery. Mediator is recruited to promoters by direct interactions with regulatory proteins and serves as a scaffold for the assembly of a functional preinitiation complex with RNA polymerase II and the general transcription factors	0.9
MED22	ENSP00000342343	mediator complex subunit 22; Component of the Mediator complex, a coactivator involved in the regulated transcription of nearly all RNA polymerase II-dependent genes. Mediator functions as a bridge to convey information from gene-specific regulatory proteins to the basal RNA polymerase II transcription machinery. Mediator is recruited to promoters by direct interactions with regulatory proteins and serves as a scaffold for the assembly of a functional preinitiation complex with RNA polymerase II and the general transcription factors	0.9
MED23	ENSP00000357047	mediator complex subunit 23	0.9
MED24	ENSP00000377686	mediator complex subunit 24; Component of the Mediator complex, a coactivator involved in the regulated transcription of nearly all RNA polymerase II-dependent genes. Mediator functions as a bridge to convey information from gene-specific regulatory proteins to the basal RNA polymerase II transcription machinery. Mediator is recruited to promoters by direct interactions with regulatory proteins and serves as a scaffold for the assembly of a functional preinitiation complex with RNA polymerase II and the general transcription factors	0.9
MED25	ENSP00000326767	mediator complex subunit 25	0.9
MED26	ENSP00000263390	mediator complex subunit 26; Component of the Mediator complex, a coactivator involved in the regulated transcription of nearly all RNA polymerase II-dependent genes. Mediator functions as a bridge to convey information from gene-specific regulatory proteins to the basal RNA polymerase II transcription machinery. Mediator is recruited to promoters by direct interactions with regulatory proteins and serves as a scaffold for the assembly of a functional preinitiation complex with RNA polymerase II and the general transcription factors	0.9
MED27	ENSP00000292035	mediator complex subunit 27; Component of the Mediator complex, a coactivator involved in the regulated transcription of nearly all RNA polymerase II-dependent genes. Mediator functions as a bridge to convey information from gene-specific regulatory proteins to the basal RNA polymerase II transcription machinery. Mediator is recruited to promoters by direct interactions with regulatory proteins and serves as a scaffold for the assembly of a functional preinitiation complex with RNA polymerase II and the general transcription factors	0.9
MED29	ENSP00000314343	mediator complex subunit 29; Component of the Mediator complex, a coactivator involved in the regulated transcription of nearly all RNA polymerase II-dependent genes. Mediator functions as a bridge to convey information from gene-specific regulatory proteins to the basal RNA polymerase II transcription machinery. Mediator is recruited to promoters by direct interactions with regulatory proteins and serves as a scaffold for the assembly of a functional preinitiation complex with RNA polymerase II and the general transcription factors	0.9
MED30	ENSP00000297347	mediator complex subunit 30; Component of the Mediator complex, a coactivator involved in the regulated transcription of nearly all RNA polymerase II-dependent genes. Mediator functions as a bridge to convey information from gene-specific regulatory proteins to the basal RNA polymerase II transcription machinery. Mediator is recruited to promoters by direct interactions with regulatory proteins and serves as a scaffold for the assembly of a functional preinitiation complex with RNA polymerase II and the general transcription factors	0.9
MED31	ENSP00000225728	mediator complex subunit 31; Component of the Mediator complex, a coactivator involved in the regulated transcription of nearly all RNA polymerase II-dependent genes. Mediator functions as a bridge to convey information from gene-specific regulatory proteins to the basal RNA polymerase II transcription machinery. Mediator is recruited to promoters by direct interactions with regulatory proteins and serves as a scaffold for the assembly of a functional preinitiation complex with RNA polymerase II and the general transcription factors	0.9
MED4	ENSP00000258648	mediator complex subunit 4; Component of the Mediator complex, a coactivator involved in the regulated transcription of nearly all RNA polymerase II-dependent genes. Mediator functions as a bridge to convey information from gene-specific regulatory proteins to the basal RNA polymerase II transcription machinery. Mediator is recruited to promoters by direct interactions with regulatory proteins and serves as a scaffold for the assembly of a functional preinitiation complex with RNA polymerase II and the general transcription factors	0.9
MED6	ENSP00000256379	mediator complex subunit 6; Component of the Mediator complex, a coactivator involved in the regulated transcription of nearly all RNA polymerase II-dependent genes. Mediator functions as a bridge to convey information from gene-specific regulatory proteins to the basal RNA polymerase II transcription machinery. Mediator is recruited to promoters by direct interactions with regulatory proteins and serves as a scaffold for the assembly of a functional preinitiation complex with RNA polymerase II and the general transcription factors (By similarity)	0.9
MED7	ENSP00000286317	mediator complex subunit 7; Component of the Mediator complex, a coactivator involved in the regulated transcription of nearly all RNA polymerase II-dependent genes. Mediator functions as a bridge to convey information from gene-specific regulatory proteins to the basal RNA polymerase II transcription machinery. Mediator is recruited to promoters by direct interactions with regulatory proteins and serves as a scaffold for the assembly of a functional preinitiation complex with RNA polymerase II and the general transcription factors	0.9
MED8	ENSP00000290663	mediator complex subunit 8; Component of the Mediator complex, a coactivator involved in the regulated transcription of nearly all RNA polymerase II-dependent genes. Mediator functions as a bridge to convey information from gene-specific regulatory proteins to the basal RNA polymerase II transcription machinery. Mediator is recruited to promoters by direct interactions with regulatory proteins and serves as a scaffold for the assembly of a functional preinitiation complex with RNA polymerase II and the general transcription factors. May play a role as a target recruitment subunit in E3 [...]	0.9
MED9	ENSP00000268711	mediator complex subunit 9; Component of the Mediator complex, a coactivator involved in the regulated transcription of nearly all RNA polymerase II-dependent genes. Mediator functions as a bridge to convey information from gene-specific regulatory proteins to the basal RNA polymerase II transcription machinery. Mediator is recruited to promoters by direct interactions with regulatory proteins and serves as a scaffold for the assembly of a functional preinitiation complex with RNA polymerase II and the general transcription factors	0.9
MPO	ENSP00000225275	myeloperoxidase; Part of the host defense system of polymorphonuclear leukocytes. It is responsible for microbicidal activity against a wide range of organisms. In the stimulated PMN, MPO catalyzes the production of hypohalous acids, primarily hypochlorous acid in physiologic situations, and other toxic intermediates that greatly enhance PMN microbicidal activity	0.57
NCOA1	ENSP00000320940	nuclear receptor coactivator 1	0.9
NCOA2	ENSP00000399968	nuclear receptor coactivator 2; Transcriptional coactivator for steroid receptors and nuclear receptors. Coactivator of the steroid binding domain (AF- 2) but not of the modulating N-terminal domain (AF-1). Required with NCOA1 to control energy balance between white and brown adipose tissues	0.9
NCOA3	ENSP00000361066	nuclear receptor coactivator 3	0.9

NCOA6	ENSP00000351894	nuclear receptor coactivator 6; Nuclear receptor coactivator that directly binds nuclear receptors and stimulates the transcriptional activities in a hormone-dependent fashion. Coactivates expression in an agonist- and AF2-dependent manner. Involved in the coactivation of different nuclear receptors, such as for steroids (GR and ERs), retinoids (RARs and RXRs), thyroid hormone (TRs), vitamin D3 (VDR) and prostanoids (PPARs). Probably functions as a general coactivator, rather than just a nuclear receptor coactivator. May also be involved in the coactivation of the NF-kappa-B pathway. M [...]	0.9
NCOR1	ENSP00000268712	nuclear receptor corepressor 1; Mediates transcriptional repression by certain nuclear receptors. Part of a complex which promotes histone deacetylation and the formation of repressive chromatin structures which may impede the access of basal transcription factors	0.9
NCOR2	ENSP00000384018	nuclear receptor corepressor 2; Transcriptional corepressor of NR4A2/NURR1 and acts through histone deacetylases (HDACs) to keep promoters of NR4A2/NURR1 target genes in a repressed deacetylated state (By similarity). Mediates the transcriptional repression activity of some nuclear receptors by promoting chromatin condensation, thus preventing access of the basal transcription. Isoform 1 and isoform 5 have different affinities for different nuclear receptors	0.9
NFYA	ENSP00000345702	nuclear transcription factor Y, alpha; Stimulates the transcription of various genes by recognizing and binding to a CCAAT motif in promoters, for example in type 1 collagen, albumin and beta-actin genes	0.909
NFYB	ENSP00000240055	nuclear transcription factor Y, beta; Stimulates the transcription of various genes by recognizing and binding to a CCAAT motif in promoters, for example in type 1 collagen, albumin and beta-actin genes	0.909
NFYC	ENSP00000396620	nuclear transcription factor Y, gamma	0.909
NPAS2	ENSP00000338283	neuronal PAS domain protein 2; BMAL1-NPAS2 heterodimers activate E-box element (5'- CACGTG-3') transcription of a number of proteins of the circadian clock. This transcription is inhibited in a feedback loop by PER, and also by CRY proteins (By similarity)	0.9
NRF1	ENSP00000223190	nuclear respiratory factor 1; Transcription factor that activates the expression of the EIF2S1 (EIF2-alpha) gene. Links the transcriptional modulation of key metabolic genes to cellular growth and development. Implicated in the control of nuclear genes required for respiration, heme biosynthesis, and mitochondrial DNA transcription and replication	0.9
O3FAR1	ENSP00000360538	omega-3 fatty acid receptor 1; Receptor for medium and long-chain free fatty acids (FFAs). Signals via a G(q)/G(11)-coupled pathway. Acts as a receptor for omega-3 fatty acids and mediates robust anti- inflammatory effects, particularly in macrophages and fat cells. The anti-inflammatory effects involve inhibition of TAK1 through a beta-arrestin 2 (ARRB2)/TAB1-dependent effect, but independent of the G(q)/G(11)-coupled pathway. Mediates potent insulin sensitizing and antidiabetic effects by repressing macrophage-induced tissue inflammation. May mediate the taste of fatty acids. Mediat [...]	0.985
PCSK1	ENSP00000308024	proprotein convertase subtilisin/kexin type 1; Involved in the processing of hormone and other protein precursors at sites comprised of pairs of basic amino acid residues. Substrates include POMC, renin, enkephalin, dynorphin, somatostatin and insulin	0.9
PEX11A	ENSP00000300056	peroxisomal biogenesis factor 11 alpha; May be involved in peroxisomal proliferation and may regulate peroxisomes division. May mediate binding of coatomer proteins to the peroxisomal membrane	0.912
PLA2G10	ENSP00000393847	phospholipase A2, group X; PA2 catalyzes the calcium-dependent hydrolysis of the 2- acyl groups in 3-sn-phosphoglycerides. Has a powerful potency for releasing arachidonic acid from cell membrane phospholipids. Prefers phosphatidylethanolamine and phosphatidylcholine liposomes to those of phosphatidylserine	0.909
PLA2G12A	ENSP00000243501	phospholipase A2, group XIIA; PA2 catalyzes the calcium-dependent hydrolysis of the 2- acyl groups in 3-sn-phosphoglycerides. Does not exhibit detectable activity toward sn-2-arachidonoyl- or linoleoyl- phosphatidylcholine or -phosphatidylethanolamine	0.9
PLA2G12B	ENSP00000362123	phospholipase A2, group XIIB; Not known; does not seem to have catalytic activity	0.9
PLA2G16	ENSP00000320337	phospholipase A2, group XVI; Exhibits PLA1/2 activity, catalyzing the calcium- independent hydrolysis of acyl groups in various phosphatidylcholines (PC) and phosphatidylethanolamine (PE). For most substrates, PLA1 activity is much higher than PLA2 activity. Specifically catalyzes the release of fatty acids from phospholipids in adipose tissue (By similarity). N- and O- acylation activity is hardly detectable. Might decrease protein phosphatase 2A (PP2A) activity	0.9
PLA2G1B	ENSP00000312286	phospholipase A2, group IB (pancreas); PA2 catalyzes the calcium-dependent hydrolysis of the 2- acyl groups in 3-sn-phosphoglycerides, this releases glycerophospholipids and arachidonic acid that serve as the precursors of signal molecules	0.92
PLA2G2A	ENSP00000364252	phospholipase A2, group IIA (platelets, synovial fluid); Thought to participate in the regulation of the phospholipid metabolism in biomembranes including eicosanoid biosynthesis. Catalyzes the calcium-dependent hydrolysis of the 2- acyl groups in 3-sn-phosphoglycerides	0.92
PLA2G2C	ENSP00000247992	phospholipase A2, group IIC; Inactive phospholipase (Probable)	0.9
PLA2G2D	ENSP00000364246	phospholipase A2, group IID; PA2 catalyzes the calcium-dependent hydrolysis of the 2- acyl groups in 3-sn-phosphoglycerides. L-alpha-1-palmitoyl-2- linoleoyl phosphatidylethanolamine is more efficiently hydrolyzed than the other phospholipids examined	0.9
PLA2G2E	ENSP00000364257	phospholipase A2, group IIE; PA2 catalyzes the calcium-dependent hydrolysis of the 2- acyl groups in 3-sn-phosphoglycerides. Has a preference for arachidonic-containing phospholipids	0.9
PLA2G2F	ENSP00000364243	phospholipase A2, group IIF; PA2 catalyzes the calcium-dependent hydrolysis of the 2- acyl groups in 3-sn-phosphoglycerides. Hydrolyzes phosphatidylglycerol versus phosphatidylcholine with a 15-fold preference	0.9
PLA2G3	ENSP00000215885	phospholipase A2, group III; PA2 catalyzes the calcium-dependent hydrolysis of the 2- acyl groups in 3-sn-phosphoglycerides. Shows an 11-fold preference for phosphatidylglycerol over phosphatidylcholine (PC). Preferential cleavage- 1-palmitoyl-2-linoleoyl-phosphatidylethanolamine (PE) > 1-palmitoyl-2-linoleoyl-PC > 1- palmitoyl-2-arachidonoyl-PC > 1-palmitoyl-2-arachidonoyl-PE. Plays a role in ciliogenesis	0.91
PLA2G4A	ENSP00000356436	phospholipase A2, group IVA (cytosolic, calcium-dependent); Selectively hydrolyzes arachidonyl phospholipids in the sn-2 position releasing arachidonic acid. Together with its lysophospholipid activity, it is implicated in the initiation of the inflammatory response	0.909
PLA2G4B	ENSP00000396045	phospholipase A2, group IVB (cytosolic); Calcium-dependent phospholipase A2 that selectively hydrolyzes glycerophospholipids in the sn-2 position with a preference for arachidonoyl phospholipids. Has a much weaker activity than PLA2G4A. Isoform 3 has calcium-dependent activity against palmitoyl-arachidonyl-phosphatidylethanolamine and low level lysophospholipase activity but no activity against phosphatidylcholine. Isoform 5 does have activity against phosphatidylcholine	0.9
PLA2G4C	ENSP00000400036	phospholipase A2, group IVC (cytosolic, calcium-independent); Has a preference for arachidonic acid at the sn-2 position of phosphatidylcholine as compared with palmitic acid	0.9
PLA2G4D	ENSP00000290472	phospholipase A2, group IVD (cytosolic); Calcium-dependent phospholipase A2 that selectively hydrolyzes glycerophospholipids in the sn-2 position. Not arachidonic acid-specific but has Linoleic acid-specific activity. May play a role in inflammation in psoriatic lesions	0.9
PLA2G4E	ENSP00000382434	phospholipase A2, group IVE; Calcium-dependent phospholipase A2 that selectively hydrolyzes glycerophospholipids in the sn-2 position (By similarity)	0.9
PLA2G4F	ENSP00000371833	phospholipase A2, group IVF; Calcium-dependent phospholipase A2 that selectively hydrolyzes glycerophospholipids in the sn-2 position. Has higher enzyme activity for phosphatidylethanolamine than phosphatidylcholine (By similarity)	0.9
PLA2G5	ENSP00000364249	phospholipase A2, group V; PA2 catalyzes the calcium-dependent hydrolysis of the 2- acyl groups in 3-sn-phosphoglycerides. This isozyme hydrolyzes more efficiently L-alpha-1-palmitoyl-2-oleoyl phosphatidylcholine than L-alpha-1-palmitoyl-2-arachidonyl phosphatidylcholine, L- alpha-1-palmitoyl-2-arachidonyl phosphatidylethanolamine, or L- alpha-1-stearoyl-2-arachidonyl phosphatidylinositol. May be involved in the production of lung surfactant, the remodeling or regulation of cardiac muscle	0.908
PLA2G6	ENSP00000333142	phospholipase A2, group VI (cytosolic, calcium-independent); Catalyzes the release of fatty acids from phospholipids. It has been implicated in normal phospholipid remodeling, nitric oxide-induced or vasopressin-induced arachidonic acid release and in leukotriene and prostaglandin production. May participate in fas mediated apoptosis and in regulating transmembrane ion flux in glucose-stimulated B-cells. Has a role in cardiolipin (CL) deacylation. Required for both speed and directionality of monocyte MCP1/CCL2-induced chemotaxis through regulation of F- actin polymerization at the pse [...]	0.921
PLB1	ENSP00000330442	phospholipase B1; Membrane-associated phospholipase. Exhibits a calcium- independent broad substrate specificity including phospholipase A2/lysophospholipase activity. Preferential hydrolysis at the sn-2 position of diacylphospholipids and diacyglycerol, whereas it shows no positional specificity toward triacylglycerol. Exhibits also esterase activity toward p-nitrophenyl. May act on the brush border membrane to facilitate the absorption of digested lipids (By similarity)	0.9
PLIN2	ENSP00000276914	perilipin 2; May be involved in development and maintenance of adipose tissue (By similarity)	0.908
PNPLA8	ENSP00000257694	patatin-like phospholipase domain containing 8	0.914
PPARA	ENSP00000262735	peroxisome proliferator-activated receptor alpha; Ligand-activated transcription factor. Key regulator of lipid metabolism. Activated by the endogenous ligand 1-palmitoyl- 2-oleoyl-sn-glycerol-3-phosphocholine (16-0/18-1-GPC). Activated by oleylethanolamide, a naturally occurring lipid that regulates satiety (By similarity). Receptor for peroxisome proliferators such as hypolipidemic drugs and fatty acids. Regulates the peroxisomal beta-oxidation pathway of fatty acids. Functions as transcription activator for the ACOX1 and P450 genes. Transactivation activity requires heterodimerizati [...]	0.939
PPARG	ENSP00000287820	peroxisome proliferator-activated receptor gamma	0.933
PPARGC1A	ENSP00000264867	peroxisome proliferator-activated receptor gamma, coactivator 1 alpha; Transcriptional coactivator for steroid receptors and nuclear receptors. Greatly increases the transcriptional activity of PPARG and thyroid hormone receptor on the uncoupling protein promoter. Can regulate key mitochondrial genes that contribute to the program of adaptive thermogenesis. Plays an essential role in metabolic reprogramming in response to dietary availability through coordination of the expression of a wide array of genes involved in glucose and fatty acid metabolism	0.9
PPARGC1B	ENSP00000312649	peroxisome proliferator-activated receptor gamma, coactivator 1 beta	0.9
PTGS1	ENSP00000354612	prostaglandin-endoperoxide synthase 1 (prostaglandin G/H synthase and cyclooxygenase); May play an important role in regulating or promoting cell proliferation in some normal and neoplastically transformed cells	0.77
PTGS2	ENSP00000356438	prostaglandin-endoperoxide synthase 2 (prostaglandin G/H synthase and cyclooxygenase); Mediates the formation of prostaglandins from arachidonate. May have a role as a major mediator of inflammation and/or a role for prostanoid signaling in activity-dependent plasticity	0.867
PXDN	ENSP00000252804	peroxidasin homolog (Drosophila); Displays low peroxidase activity and is likely to participate in H(2)O(2) metabolism and peroxidative reactions in the cardiovascular system. Plays a role in extracellular matrix formation	0.605
PXDNL	ENSP00000348645	peroxidasin homolog (Drosophila)-like; Isoform PMR1- Endonuclease selectively degrading some target mRNAs while they are engaged by translating ribosomes, among which albumin and beta-globin mRNAs	0.57
RGL1	ENSP00000303192	ral guanine nucleotide dissociation stimulator-like 1; Probable guanine nucleotide exchange factor	0.9
RORA	ENSP00000261523	RAR-related orphan receptor A; Orphan nuclear receptor. Binds DNA as a monomer to hormone response elements (HRE) containing a single core motif half-site preceded by a short A-T-rich sequence. This isomer binds to the consensus sequence 5'-[AT][TA]A[AT][CGT]TAGGTCA-3'. Regulates a number of genes involved in lipid metabolism such as apolipoproteins AI, APOA5, CIII, CYP71 and PPARgamma, in cerebellum and photoreceptor development including PCP2, OPN1SW, OPN1SM AND ARR3, in circadian rhythm with BMAL1, and skeletal muscle development with MYOD1. Possible receptor for cholesterol or one [...]	0.9

	RXRA	ENSP00000419692	retinoid X receptor, alpha; Receptor for retinoic acid. Retinoic acid receptors bind as heterodimers to their target response elements in response to their ligands, all-trans or 9-cis retinoic acid, and regulate gene expression in various biological processes. The RAR/RXR heterodimers bind to the retinoic acid response elements (RARE) composed of tandem 5'-AGGTCA-3' sites known as DR1-DR5. The high affinity ligand for RXRs is 9-cis retinoic acid. RXRA serves as a common heterodimeric partner for a number of nuclear receptors. The RXR/RAR heterodimers bind to the retinoic acid response [...]	0.9
	SCARB1	ENSP00000261693	scavenger receptor class B, member 1; Receptor for different ligands such as phospholipids, cholesterol ester, lipoproteins, phosphatidylserine and apoptotic cells. Probable receptor for HDL, located in particular region of the plasma membrane, called caveolae. Facilitates the flux of free and esterified cholesterol between the cell surface and extracellular donors and acceptors, such as HDL and to a lesser extent, apoB-containing lipoproteins and modified lipoproteins. Probably involved in the phagocytosis of apoptotic cells, via its phosphatidylserine binding activity. Receptor for h [...]	0.838
	SCD	ENSP00000359380	stearoyl-CoA desaturase (delta-9-desaturase)	0.433
	SIN3A	ENSP00000353622	SIN3 transcription regulator homolog A (yeast); Acts as a transcriptional repressor. Corepressor for REST. Interacts with MXI1 to repress MYC responsive genes and antagonize MYC oncogenic activities. Also interacts with MXD1-MAX heterodimers to repress transcription by tethering SIN3A to DNA (By similarity). Acts cooperatively with OGT to repress transcription in parallel with histone deacetylation	0.9
	SLC27A1	ENSP00000252595	solute carrier family 27 (fatty acid transporter), member 1; Involved in translocation of long-chain fatty acids (LFCA) across the plasma membrane. The LFCA import appears to be hormone-regulated in a tissue-specific manner. In adipocytes, but not myocytes, insulin induces a rapid translocation of FATP1 from intracellular compartments to the plasma membrane, paralleled by increased LFCA uptake. May act directly as a bona fide transporter, or alternatively, in a cytoplasmic or membrane- associated multimeric protein complex to trap and draw fatty acids towards accumulation. Plays a pivo [...]	0.908
	SLC8A1	ENSP00000332931	solute carrier family 8 (sodium/calcium exchanger), member 1	0.818
	SMARCD3	ENSP00000262188	SWI/SNF related, matrix associated, actin dependent regulator of chromatin, subfamily d, member 3; Plays a role in ATP dependent nucleosome remodeling by SMARCA4 containing complexes. Stimulates nuclear receptor mediated transcription. Belongs to the neural progenitors-specific chromatin remodeling complex (npBAF complex) and the neuron- specific chromatin remodeling complex (nBAF complex). During neural development a switch from a stem/progenitor to a post- mitotic chromatin remodeling mechanism occurs as neurons exit the cell cycle and become committed to their adult state. The trans [...]	0.9
	SP1	ENSP00000329357	Sp1 transcription factor; Transcription factor that can activate or repress transcription in response to physiological and pathological stimuli. Binds with high affinity to GC-rich motifs and regulates the expression of a large number of genes involved in a variety of processes such as cell growth, apoptosis, differentiation and immune responses. Highly regulated by post-translational modifications (phosphorylations, sumoylation, proteolytic cleavage, glycosylation and acetylation). Binds also the PDGFR- alpha G-box promoter. May have a role in modulating the cellular response to DNA d [...]	0.907
	SREBF2	ENSP00000354476	sterol regulatory element binding transcription factor 2; Transcriptional activator required for lipid homeostasis. Regulates transcription of the LDL receptor gene as well as the cholesterol and to a lesser degree the fatty acid synthesis pathway (By similarity). Binds the sterol regulatory element 1 (SRE-1) (5'-ATCACCCCAC-3') found in the flanking region of the LDRL and HMG-CoA synthase genes	0.9
	SULT2A1	ENSP00000222002	sulfotransferase family, cytosolic, 2A, dehydroepiandrosterone (DHEA)-preferring, member 1; Sulfotransferase that utilizes 3'-phospho-5'-adenylyl sulfate (PAPS) as sulfonate donor to catalyze the sulfonation of steroids and bile acids in the liver and adrenal glands	0.9
	TBL1X	ENSP00000217964	transducin (beta)-like 1X-linked; F-box-like protein involved in the recruitment of the ubiquitin/19S proteasome complex to nuclear receptor-regulated transcription units. Plays an essential role in transcription activation mediated by nuclear receptors. Probably acts as integral component of corepressor complexes that mediates the recruitment of the 19S proteasome complex, leading to the subsequent proteasomal degradation of transcription repressor complexes, thereby allowing cofactor exchange	0.9
	TBL1XR1	ENSP00000405574	transducin (beta)-like 1 X-linked receptor 1; F-box-like protein involved in the recruitment of the ubiquitin/19S proteasome complex to nuclear receptor-regulated transcription units. Plays an essential role in transcription activation mediated by nuclear receptors. Probably acts as integral component of the N-Cor corepressor complex that mediates the recruitment of the 19S proteasome complex, leading to the subsequent proteasomal degradation of N-Cor complex, thereby allowing cofactor exchange, and transcription activation	0.9
	TEAD1	ENSP00000354588	TEA domain family member 1 (SV40 transcriptional enhancer factor); Transcription factor which plays a key role in the Hippo signaling pathway, a pathway involved in organ size control and tumor suppression by restricting proliferation and promoting apoptosis. The core of this pathway is composed of a kinase cascade wherein MST1/MST2, in complex with its regulatory protein SAV1, phosphorylates and activates LATS1/2 in complex with its regulatory protein MOB1, which in turn phosphorylates and inactivates YAP1 oncoprotein and WWTR1/TAZ. Acts by mediating gene expression of YAP1 and WWTR1/ [...]	0.9
	TEAD2	ENSP00000310701	TEA domain family member 2; Transcription factor which plays a key role in the Hippo signaling pathway, a pathway involved in organ size control and tumor suppression by restricting proliferation and promoting apoptosis. The core of this pathway is composed of a kinase cascade wherein MST1/MST2, in complex with its regulatory protein SAV1, phosphorylates and activates LATS1/2 in complex with its regulatory protein MOB1, which in turn phosphorylates and inactivates YAP1 oncoprotein and WWTR1/TAZ. Acts by mediating gene expression of YAP1 and WWTR1/TAZ, thereby regulating cell proliferat [...]	0.9
	TEAD3	ENSP00000345772	TEA domain family member 3; Transcription factor which plays a key role in the Hippo signaling pathway, a pathway involved in organ size control and tumor suppression by restricting proliferation and promoting apoptosis. The core of this pathway is composed of a kinase cascade wherein MST1/MST2, in complex with its regulatory protein SAV1, phosphorylates and activates LATS1/2 in complex with its regulatory protein MOB1, which in turn phosphorylates and inactivates YAP1 oncoprotein and WWTR1/TAZ. Acts by mediating gene expression of YAP1 and WWTR1/TAZ, thereby regulating cell proliferat [...]	0.9
	TEAD4	ENSP00000352926	TEA domain family member 4; Transcription factor which plays a key role in the Hippo signaling pathway, a pathway involved in organ size control and tumor suppression by restricting proliferation and promoting apoptosis. The core of this pathway is composed of a kinase cascade wherein MST1/MST2, in complex with its regulatory protein SAV1, phosphorylates and activates LATS1/2 in complex with its regulatory protein MOB1, which in turn phosphorylates and inactivates YAP1 oncoprotein and WWTR1/TAZ. Acts by mediating gene expression of YAP1 and WWTR1/TAZ, thereby regulating cell proliferat [...]	0.9
	TGS1	ENSP00000260129	trimethylguanosine synthase 1; Catalyzes the 2 serial methylation steps for the conversion of the 7-monomethylguanosine (m(7)G) caps of snRNAs and snoRNAs to a 2,2,7-trimethylguanosine (m(2,2,7)G) cap structure. The enzyme is specific for guanine, and N7 methylation must precede N2 methylation. Hypermethylation of the m7G cap of U snRNAs leads to their concentration in nuclear foci, their colocalization with coilin and the formation of canonical Cajal bodies (CBs). Plays a role in transcriptional regulation	0.9
	TIAM2	ENSP00000327315	T-cell lymphoma invasion and metastasis 2; Modulates the activity of RHO-like proteins and connects extracellular signals to cytoskeletal activities. Acts as a GDP- dissociation stimulator protein that stimulates the GDP-GTP exchange activity of RHO-like GTPases and activates them. Mediates extracellular laminin signals to activate Rac1, contributing to neurite growth. Involved in lamellipodial formation and advancement of the growth cone of embryonic hippocampal neurons. Promotes migration of neurons in the cerebral cortex. When overexpressed, induces membrane ruffling accompanied by [...]	0.9
	TNFRSF21	ENSP00000296861	tumor necrosis factor receptor superfamily, member 21; May activate NF-kappa-B and promote apoptosis. May activate JNK and be involved in T-cell differentiation. Required for both normal cell body death and axonal pruning. Trophic-factor deprivation triggers the cleavage of surface APP by beta-secretase to release sAPP-beta which is further cleaved to release an N- terminal fragment of APP (N-APP). N-APP binds TNFRSF21 triggering caspase activation and degeneration of both neuronal cell bodies (via caspase-3) and axons (via caspase-6)	0.9
	TPO	ENSP00000318820	thyroid peroxidase	0.57
	TRIB3	ENSP00000217233	tribbles homolog 3 (Drosophila); Disrupts insulin signaling by binding directly to Akt kinases and blocking their activation. May bind directly to and mask the 'Thr-308' phosphorylation site in AKT1. Binds to ATF4 and inhibits its transcriptional activation activity. Interacts with the NF-kappa-B transactivator p65 RELA and inhibits its phosphorylation and thus its transcriptional activation activity. Interacts with MAPK kinases and regulates activation of MAP kinases. May play a role in programmed neuronal cell death but does not appear to affect non-neuronal cells. Does not display k [...]	0.9
	TRPV1	ENSP00000382659	transient receptor potential cation channel, subfamily V, member 1; Receptor-activated non-selective calcium permeant cation channel involved in detection of noxious chemical and thermal stimuli. Seems to mediate proton influx and may be involved in intracellular acidosis in nociceptive neurons. May be involved in mediation of inflammatory pain and hyperalgesia. Sensitized by a phosphatidylinositol second messenger system activated by receptor tyrosine kinases, which involves PKC isozymes and PCL. Acts as ionotropic endocannabinoid receptor with central neuromodulatory effects. Trigger [...]	0.815
	TXNRD1	ENSP00000434516	thioredoxin reductase 1	0.9
	UGT1A9	ENSP00000346768	UDP glucuronosyltransferase 1 family, polypeptide A9; UDPGT is of major importance in the conjugation and subsequent elimination of potentially toxic xenobiotics and endogenous compounds. This isoform has specificity for phenols	0.9
	VR1	ENSP00000459962	transient receptor potential cation channel subfamily V member 1 ; Receptor-activated non-selective calcium permeant cation channel involved in detection of noxious chemical and thermal stimuli. Seems to mediate proton influx and may be involved in intracellular acidosis in nociceptive neurons. May be involved in mediation of inflammatory pain and hyperalgesia. Sensitized by a phosphatidylinositol second messenger system activated by receptor tyrosine kinases, which involves PKC isozymes and PCL. Acts as ionotropic endocannabinoid receptor with central neuromodulatory effects. Triggers [...]	0.815
	WWTR1	ENSP00000353847	WW domain containing transcription regulator 1; Transcriptional coactivator which acts as a downstream regulatory target in the Hippo signaling pathway that plays a pivotal role in organ size control and tumor suppression by restricting proliferation and promoting apoptosis. The core of this pathway is composed of a kinase cascade wherein STK3/MST2 and STK4/MST1, in complex with its regulatory protein SAV1, phosphorylates and activates LATS1/2 in complex with its regulatory protein MOB1, which in turn phosphorylates and inactivates YAP1 oncoprotein and WWTR1/TAZ. WWTR1 enhances PAX8 an [...]	0.9
	YAP1	ENSP00000282441	Yes-associated protein 1; Transcriptional regulator which can act both as a coactivator and a corepressor and is the critical downstream regulatory target in the Hippo signaling pathway that plays a pivotal role in organ size control and tumor suppression by restricting proliferation and promoting apoptosis. The core of this pathway is composed of a kinase cascade wherein STK3/MST2 and STK4/MST1, in complex with its regulatory protein SAV1, phosphorylates and activates LATS1/2 in complex with its regulatory protein MOB1, which in turn phosphorylates and inactivates YAP1 oncoprotein and [...]	0.9
**node1**	**Oroxylin A**	**5320315**	**Oroxylin A is an O-methylated flavone, a chemical compound that can be found in the medicinal plant "Scutellaria baicalensis" and the "Oroxylum indicum" tree. It has demonstrated activity as a dopamine reuptake inhibitor, and is also a negative allosteric modulator of the benzodiazepine site of the GABAA receptor. Oroxylin A has been found to improve memory consolidation in mice by elevating brain-derived neurotrophic factor (BDNF) levels in the hippocampus.**	
node2	BDNF	ENSP00000414303	brain-derived neurotrophic factor	0.8
	CASP8	ENSP00000351273	caspase 8, apoptosis-related cysteine peptidase	0.7
	HK2	ENSP00000290573	hexokinase 2	0.8
	IL6	ENSP00000258743	interleukin 6 (interferon, beta 2); Cytokine with a wide variety of biological functions. It is a potent inducer of the acute phase response. Plays an essential role in the final differentiation of B-cells into Ig- secreting cells Involved in lymphocyte and monocyte differentiation. It induces myeloma and plasmacytoma growth and induces nerve cells differentiation Acts on B-cells, T-cells, hepatocytes, hematopoietic progenitor cells and cells of the CNS. Also acts as a myokine. It is discharged into the bloodstream after muscle contraction and acts to increase the breakdown of fats and [...]	0.8

	MAPK1	ENSP00000215832	mitogen-activated protein kinase 1; Serine/threonine kinase which acts as an essential component of the MAP kinase signal transduction pathway. MAPK1/ERK2 and MAPK3/ERK1 are the 2 MAPKs which play an important role in the MAPK/ERK cascade. They participate also in a signaling cascade initiated by activated KIT and KITLG/SCF. Depending on the cellular context, the MAPK/ERK cascade mediates diverse biological functions such as cell growth, adhesion, survival and differentiation through the regulation of transcription, translation, cytoskeletal rearrangements. The MAPK/ERK cascade plays a [...]	0.7
	MAPK3	ENSP00000263025	mitogen-activated protein kinase 3; Serine/threonine kinase which acts as an essential component of the MAP kinase signal transduction pathway. MAPK1/ERK2 and MAPK3/ERK1 are the 2 MAPKs which play an important role in the MAPK/ERK cascade. They participate also in a signaling cascade initiated by activated KIT and KITLG/SCF. Depending on the cellular context, the MAPK/ERK cascade mediates diverse biological functions such as cell growth, adhesion, survival and differentiation through the regulation of transcription, translation, cytoskeletal rearrangements. The MAPK/ERK cascade plays a [...]	0.7
	MTRR	ENSP00000264668	5-methyltetrahydrofolate-homocysteine methyltransferase reductase; Involved in the reductive regeneration of cob(I)alamin cofactor required for the maintenance of methionine synthase in a functional state	0.473
	NOS1	ENSP00000337459	nitric oxide synthase 1 (neuronal); Produces nitric oxide (NO) which is a messenger molecule with diverse functions throughout the body. In the brain and peripheral nervous system, NO displays many properties of a neurotransmitter. Probably has nitrosylase activity and mediates cysteine S-nitrosylation of cytoplasmic target proteins such SRR	0.786
	NOS2	ENSP00000327251	nitric oxide synthase 2, inducible	0.613
	NOS3	ENSP00000297494	nitric oxide synthase 3 (endothelial cell); Produces nitric oxide (NO) (By similarity)	0.613
	PARP1	ENSP00000355759	poly (ADP-ribose) polymerase 1; Involved in the base excision repair (BER) pathway, by catalyzing the poly(ADP-ribosyl)ation of a limited number of acceptor proteins involved in chromatin architecture and in DNA metabolism. This modification follows DNA damages and appears as an obligatory step in a detection/signaling pathway leading to the reparation of DNA strand breaks. Mediates the poly(ADP- ribosyl)ation of APLF and CHFR. Positively regulates the transcription of MTUS1 and negatively regulates the transcription of MTUS2/TIP150. With EEF1A1 and TXK, forms a complex that acts as a [...]	0.7
	POR	ENSP00000419970	P450 (cytochrome) oxidoreductase; This enzyme is required for electron transfer from NADP to cytochrome P450 in microsomes. It can also provide electron transfer to heme oxygenase and cytochrome B5	0.473
	PPIF	ENSP00000225174	peptidylprolyl isomerase F; PPIases accelerate the folding of proteins. It catalyzes the cis-trans isomerization of proline imidic peptide bonds in oligopeptides. Involved in regulation of the mitochondrial permeability transition pore (mPTP). It is proposed that its association with the mPTP is masking a binding site for inhibiting inorganic phosphate (Pi) and promotes the open probablity of the mPTP leading to apoptosis or necrosis; the requirement of the PPIase activity for this function is debated. In cooperation with mitochondrial TP53 is involved in activating oxidative stress- i [...]	0.8
	SIRT3	ENSP00000372191	sirtuin 3; NAD-dependent protein deacetylase. Activates mitochondrial target proteins, including ACSS1, IDH2 and GDH by deacetylating key lysine residues. Contributes to the regulation of the cellular energy metabolism. Important for regulating tissue-specific ATP levels	0.8
	SOD2	ENSP00000356022	superoxide dismutase 2, mitochondrial; Destroys radicals which are normally produced within the cells and which are toxic to biological systems (By similarity)	0.8
**nod1**	**Panicolin**	**5320399**	**skullcapflavone I**	
**nod2**	CASP3	ENSP00000311032	caspase 3, apoptosis-related cysteine peptidase; Involved in the activation cascade of caspases responsible for apoptosis execution. At the onset of apoptosis it proteolytically cleaves poly(ADP-ribose) polymerase (PARP) at a '216-Asp-|-Gly-217' bond. Cleaves and activates sterol regulatory element binding proteins (SREBPs) between the basic helix-loop- helix leucine zipper domain and the membrane attachment domain. Cleaves and activates caspase-6, -7 and -9. Involved in the cleavage of huntingtin. Triggers cell adhesion in sympathetic neurons through RET cleavage	0.8
**node1**	**Sitogluside**	**71628**	**See also eleutherosides & syringin for eleutheroside b: 118-34-3**	
**node2**	UGCG	ENSP00000363397	UDP-glucose ceramide glucosyltransferase; Catalyzes the first glycosylation step in glycosphingolipid biosynthesis, the transfer of glucose to ceramide. May also serve as a "flippase"	0.43
**node1**	**Sitosterol**	**222284**	**Sitosterol**	
**node2**	ABCA1	ENSP00000363868	ATP-binding cassette, sub-family A (ABC1), member 1	0.614
	ABCB11	ENSP00000263817	ATP-binding cassette, sub-family B (MDR/TAP), member 11; Involved in the ATP-dependent secretion of bile salts into the canaliculus of hepatocytes	0.815
	ABCG1	ENSP00000354995	ATP-binding cassette, sub-family G (WHITE), member 1	0.529
	ABCG5	ENSP00000260645	ATP-binding cassette, sub-family G (WHITE), member 5; Transporter that appears to play an indispensable role in the selective transport of the dietary cholesterol in and out of the enterocytes and in the selective sterol excretion by the liver into bile	0.879
	ABCG8	ENSP00000272286	ATP-binding cassette, sub-family G (WHITE), member 8; Transporter that appears to play an indispensable role in the selective transport of the dietary cholesterol in and out of the enterocytes and in the selective sterol excretion by the liver into bile	0.92
	APOE	ENSP00000252486	apolipoprotein E; Mediates the binding, internalization, and catabolism of lipoprotein particles. It can serve as a ligand for the LDL (apo B/E) receptor and for the specific apo-E receptor (chylomicron remnant) of hepatic tissues	0.872
	CASP3	ENSP00000311032	caspase 3, apoptosis-related cysteine peptidase; Involved in the activation cascade of caspases responsible for apoptosis execution. At the onset of apoptosis it proteolytically cleaves poly(ADP-ribose) polymerase (PARP) at a '216-Asp-|-Gly-217' bond. Cleaves and activates sterol regulatory element binding proteins (SREBPs) between the basic helix-loop- helix leucine zipper domain and the membrane attachment domain. Cleaves and activates caspase-6, -7 and -9. Involved in the cleavage of huntingtin. Triggers cell adhesion in sympathetic neurons through RET cleavage	0.818
	CYP7A1	ENSP00000301645	cytochrome P450, family 7, subfamily A, polypeptide 1; Catalyzes a rate-limiting step in cholesterol catabolism and bile acid biosynthesis by introducing a hydrophilic moiety at position 7 of cholesterol. Important for cholesterol homeostasis	0.771
	DHCR24	ENSP00000360316	24-dehydrocholesterol reductase; Catalyzes the reduction of the delta-24 double bond of sterol intermediates. Protects cells from oxidative stress by reducing caspase 3 activity during apoptosis induced by oxidative stress. Also protects against amyloid-beta peptide-induced apoptosis	0.841
	ICAM1	ENSP00000264832	intercellular adhesion molecule 1; ICAM proteins are ligands for the leukocyte adhesion protein LFA-1 (integrin alpha-L/beta-2). During leukocyte trans- endothelial migration, ICAM1 engagement promotes the assembly of endothelial apical cups through ARHGEF26/SGEF and RHOG activation. In case of rhinovirus infection acts as a cellular receptor for the virus	0.8
	NPC1L1	ENSP00000289547	NPC1-like 1; Plays a major role in cholesterol homeostasis. Is critical for the uptake of cholesterol across the plasma membrane of the intestinal enterocyte. Is the direct molecular target of ezetimibe, a drug that inhibits cholesterol absorbtion. Lack of activity leads to multiple lipid transport defects. The protein may have a function in the transport of multiple lipids and their homeostasis, and may play a critical role in regulating lipid metabolism. Acts as a negative regulator of NPC2 and down- regulates its expression and secretion by inhibiting its maturation and accelerating [...]	0.467
	NR1H2	ENSP00000253727	nuclear receptor subfamily 1, group H, member 2; Orphan receptor. Binds preferentially to double-stranded oligonucleotide direct repeats having the consensus half-site sequence 5'-AGGTCA-3' and 4-nt spacing (DR-4). Regulates cholesterol uptake through MYLIP-dependent ubiquitination of LDLR, VLDLR and LRP8 (By similarity)	0.58
	NR1H3	ENSP00000387946	nuclear receptor subfamily 1, group H, member 3; Orphan receptor. Interaction with RXR shifts RXR from its role as a silent DNA-binding partner to an active ligand- binding subunit in mediating retinoid responses through target genes defined by LXRES. LXRES are DR4-type response elements characterized by direct repeats of two similar hexanuclotide half- sites spaced by four nucleotides. Plays an important role in the regulation of cholesterol homeostasis, regulating cholesterol uptake through MYLIP-dependent ubiquitination of LDLR, VLDLR and LRP8 (By similarity)	0.576
	SREBF1	ENSP00000348069	sterol regulatory element binding transcription factor 1; Transcriptional activator required for lipid homeostasis. Regulates transcription of the LDL receptor gene as well as the fatty acid and to a lesser degree the cholesterol synthesis pathway (By similarity). Binds to the sterol regulatory element 1 (SRE-1) (5'-ATCACCCCAC-3'). Has dual sequence specificity binding to both an E-box motif (5'-ATCACGTGA-3') and to SRE-1 (5'-ATCACCCCAC-3')	0.816
	SREBF2	ENSP00000354476	sterol regulatory element binding transcription factor 2; Transcriptional activator required for lipid homeostasis. Regulates transcription of the LDL receptor gene as well as the cholesterol and to a lesser degree the fatty acid synthesis pathway (By similarity). Binds the sterol regulatory element 1 (SRE-1) (5'-ATCACCCCAC-3') found in the flanking region of the LDRL and HMG-CoA synthase genes	0.926
**node1**	**Stigmasterol**	**5280794**	**Stigmasterol**	
**node2**	ABCA1	ENSP00000363868	ATP-binding cassette, sub-family A (ABC1), member 1	0.88
	ABCG5	ENSP00000260645	ATP-binding cassette, sub-family G (WHITE), member 5; Transporter that appears to play an indispensable role in the selective transport of the dietary cholesterol in and out of the enterocytes and in the selective sterol excretion by the liver into bile	0.711
	ABCG8	ENSP00000272286	ATP-binding cassette, sub-family G (WHITE), member 8; Transporter that appears to play an indispensable role in the selective transport of the dietary cholesterol in and out of the enterocytes and in the selective sterol excretion by the liver into bile	0.811
	CTSE	ENSP00000350911	cathepsin E	0.436
	IL10	ENSP00000412237	interleukin 10; Inhibits the synthesis of a number of cytokines, including IFN-gamma, IL-2, IL-3, TNF and GM-CSF produced by activated macrophages and by helper T-cells	0.7
	IL8	ENSP00000306512	interleukin 8	0.7
	NR1H2	ENSP00000253727	nuclear receptor subfamily 1, group H, member 2; Orphan receptor. Binds preferentially to double-stranded oligonucleotide direct repeats having the consensus half-site sequence 5'-AGGTCA-3' and 4-nt spacing (DR-4). Regulates cholesterol uptake through MYLIP-dependent ubiquitination of LDLR, VLDLR and LRP8 (By similarity)	0.437
	NR1H3	ENSP00000387946	nuclear receptor subfamily 1, group H, member 3; Orphan receptor. Interaction with RXR shifts RXR from its role as a silent DNA-binding partner to an active ligand- binding subunit in mediating retinoid responses through target genes defined by LXRES. LXRES are DR4-type response elements characterized by direct repeats of two similar hexanuclotide half- sites spaced by four nucleotides. Plays an important role in the regulation of cholesterol homeostasis, regulating cholesterol uptake through MYLIP-dependent ubiquitination of LDLR, VLDLR and LRP8 (By similarity)	0.476

	SLCO1B1	ENSP00000256958	solute carrier organic anion transporter family, member 1B1; Mediates the Na(+)-independent uptake of organic anions such as pravastatin, taurocholate, methotrexate, dehydroepiandrosterone sulfate, 17-beta-glucuronosyl estradiol, estrone sulfate, prostaglandin E2, thromboxane B2, leukotriene C3, leukotriene E4, thyroxine and triiodothyronine. Involved in the clearance of bile acids and organic anions from the liver	0.7
	SREBF2	ENSP00000354476	sterol regulatory element binding transcription factor 2; Transcriptional activator required for lipid homeostasis. Regulates transcription of the LDL receptor gene as well as the cholesterol and to a lesser degree the fatty acid synthesis pathway (By similarity). Binds the sterol regulatory element 1 (SRE-1) (5'-ATCACCCCAC-3') found in the flanking region of the LDRL and HMG-CoA synthase genes	0.622
	TNF	ENSP00000398698	tumor necrosis factor	0.7
**node1**	**Supraene**	**638072**	**Supraene**	
**node2**	CCL2	ENSP00000225831	chemokine (C-C motif) ligand 2; Chemotactic factor that attracts monocytes and basophils but not neutrophils or eosinophils. Augments monocyte anti-tumor activity. Has been implicated in the pathogenesis of diseases characterized by monocytic infiltrates, like psoriasis, rheumatoid arthritis or atherosclerosis. May be involved in the recruitment of monocytes into the arterial wall during the disease process of atherosclerosis	0.7
	CCL3	ENSP00000225245	chemokine (C-C motif) ligand 3; Monokine with inflammatory and chemokinetic properties. Binds to CCR1, CCR4 and CCR5. One of the major HIV-suppressive factors produced by CD8+ T-cells. Recombinant MIP-1-alpha induces a dose-dependent inhibition of different strains of HIV-1, HIV-2, and simian immunodeficiency virus (SIV)	0.7
	CCL4	ENSP00000250151	chemokine (C-C motif) ligand 4; Monokine with inflammatory and chemokinetic properties. Binds to CCR5. One of the major HIV-suppressive factors produced by CD8+ T-cells. Recombinant MIP-1-beta induces a dose-dependent inhibition of different strains of HIV-1, HIV-2, and simian immunodeficiency virus (SIV). The processed form MIP-1-beta(3-69) retains the abilities to induce down-modulation of surface expression of the chemokine receptor CCR5 and to inhibit the CCR5- mediated entry of HIV-1 in T-cells. MIP-1-beta(3-69) is also a ligand for CCR1 and CCR2 isoform B	0.7
	CCL5	ENSP00000293272	chemokine (C-C motif) ligand 5; Chemoattractant for blood monocytes, memory T-helper cells and eosinophils. Causes the release of histamine from basophils and activates eosinophils. Binds to CCR1, CCR3, CCR4 and CCR5. One of the major HIV-suppressive factors produced by CD8+ T- cells. Recombinant RANTES protein induces a dose-dependent inhibition of different strains of HIV-1, HIV-2, and simian immunodeficiency virus (SIV). The processed form RANTES(3-68) acts as a natural chemotaxis inhibitor and is a more potent inhibitor of HIV-1-infection. The second processed form RANTES(4-68) exh [...]	0.7
	DHCR24	ENSP00000360316	24-dehydrocholesterol reductase; Catalyzes the reduction of the delta-24 double bond of sterol intermediates. Protects cells from oxidative stress by reducing caspase 3 activity during apoptosis induced by oxidative stress. Also protects against amyloid-beta peptide-induced apoptosis	0.802
	DHCR7	ENSP00000347717	7-dehydrocholesterol reductase; Production of cholesterol by reduction of C7-C8 double bond of 7-dehydrocholesterol (7-DHC)	0.698
	EBP	ENSP00000417052	emopamil binding protein (sterol isomerase); Catalyzes the conversion of Delta(8)-sterols to their corresponding Delta(7)-isomers	0.431
	FDFT1	ENSP00000220584	farnesyl-diphosphate farnesyltransferase 1	0.992
	FDPS	ENSP00000349078	farnesyl diphosphate synthase; Key enzyme in isoprenoid biosynthesis which catalyzes the formation of farnesyl diphosphate (FPP), a precursor for several classes of essential metabolites including sterols, dolichols, carotenoids, and ubiquinones. FPP also serves as substrate for protein farnesylation and geranylgeranylation. Catalyzes the sequential condensation of isopentenyl pyrophosphate with the allylic pyrophosphates, dimethylallyl pyrophosphate, and then with the resultant geranylpyrophosphate to the ultimate product farnesyl pyrophosphate	0.458
	GYPC	ENSP00000259254	glycophorin C (Gerbich blood group); This protein is a minor sialoglycoprotein in human erythrocyte membranes. The blood group Gerbich antigens and receptors for Plasmodium falciparum merozoites are most likely located within the extracellular domain. Glycophorin-C plays an important role in regulating the stability of red cells	0.57
	HMGB1	ENSP00000343040	high mobility group box 1; DNA binding proteins that associates with chromatin and has the ability to bend DNA. Binds preferentially single-stranded DNA. Involved in V(D)J recombination by acting as a cofactor of the RAG complex. Acts by stimulating cleavage and RAG protein binding at the 23 bp spacer of conserved recombination signal sequences (RSS). Heparin-binding protein that has a role in the extension of neurite-type cytoplasmic processes in developing cells (By similarity)	0.452
	HMGB2	ENSP00000296503	high mobility group box 2; DNA binding proteins that associates with chromatin and has the ability to bend DNA. Binds preferentially single-stranded DNA. Involved in V(D)J recombination by acting as a cofactor of the RAG complex. Acts by stimulating cleavage and RAG protein binding at the 23 bp spacer of conserved recombination signal sequences (RSS) (By similarity)	0.493
	HMGCR	ENSP00000287936	3-hydroxy-3-methylglutaryl-CoA reductase; Transmembrane glycoprotein that is the rate-limiting enzyme in cholesterol biosynthesis as well as in the biosynthesis of nonsterol isoprenoids that are essential for normal cell function including ubiquinone and geranylgeranyl proteins	0.65
	IL1RN	ENSP00000259206	interleukin 1 receptor antagonist; Inhibits the activity of interleukin-1 by binding to receptor IL1R1 and preventing its association with the coreceptor IL1RAP for signaling. Has no interleukin-1 like activity. Binds functional interleukin-1 receptor IL1R1 with greater affinity than decoy receptor IL1R2; however, the physiological relevance of the latter association is unsure	0.7
	IL8	ENSP00000306512	interleukin 8	0.7
	LBR	ENSP00000272163	lamin B receptor; Anchors the lamina and the heterochromatin to the inner nuclear membrane	0.43
	LSS	ENSP00000348762	lanosterol synthase (2,3-oxidoSupraene-lanosterol cyclase); Catalyzes the cyclization of (S)-2,3 oxidoSupraene to lanosterol, a reaction that forms the sterol nucleus	0.941
	NSDHL	ENSP00000359297	NAD(P) dependent steroid dehydrogenase-like	0.56
	SC5DL	ENSP00000264027	sterol-C5-desaturase (ERG3 delta-5-desaturase homolog, S. cerevisiae)-like; Catalyzes a dehydrogenation to introduce C5-6 double bond into lathosterol	0.693
	SEC14L1	ENSP00000376268	SEC14-like 1 (S. cerevisiae)	0.469
	SEC14L2	ENSP00000316203	SEC14-like 2 (S. cerevisiae); Carrier protein. Binds to some hydrophobic molecules and promotes their transfer between the different cellular sites. Binds with high affinity to alpha-tocopherol. Also binds with a weaker affinity to other tocopherols and to tocotrienols. May have a transcriptional activatory activity via its association with alpha-tocopherol. Probably recognizes and binds some Supraene structure, suggesting that it may regulate cholesterol biosynthesis by increasing the transfer of Supraene to a metabolic active pool in the cell	0.521
	SEC14L3	ENSP00000215812	SEC14-like 3 (S. cerevisiae); Probable hydrophobic ligand-binding protein; may play a role in the transport of hydrophobic ligands like tocopherol, Supraene and phospholipids	0.589
	SEC14L4	ENSP00000255858	SEC14-like 4 (S. cerevisiae); Probable hydrophobic ligand-binding protein; may play a role in the transport of hydrophobic ligands like tocopherol, Supraene and phospholipids	0.519
	SEC14L5	ENSP00000251170	SEC14-like 5 (S. cerevisiae)	0.469
	SQLE	ENSP00000265896	Supraene epoxidase; Catalyzes the first oxygenation step in sterol biosynthesis and is suggested to be one of the rate-limiting enzymes in this pathway	0.986
	TM7SF2	ENSP00000279263	transmembrane 7 superfamily member 2; Involved in the conversion of lanosterol to cholesterol	0.524
**node1**	**Wogonin**	**5281703**	**Wogonin is an O-methylated flavone, a flavonoid-like chemical compound which was found in "Scutellaria baicalensis". The glycosides of Wogonin are known as wogonosides. For example, oroxindin is a Wogonin glucuronide isolated from "Oroxylum indicum". It is one of the active ingredients of Sho-Saiko-To, a Japanese herbal supplement.**	
**node2**	BAK1	ENSP00000353878	BCL2-antagonist/killer 1; In the presence of an appropriate stimulus, accelerates programmed cell death by binding to, and antagonizing the anti- apoptotic action of BCL2 or its adenovirus homolog E1B 19k protein. Low micromolar levels of zinc ions inhibit the promotion of apoptosis	0.444
	BCL2	ENSP00000329623	B-cell CLL/lymphoma 2; Suppresses apoptosis in a variety of cell systems including factor-dependent lymphohematopoietic and neural cells. Regulates cell death by controlling the mitochondrial membrane permeability. Appears to function in a feedback loop system with caspases. Inhibits caspase activity either by preventing the release of cytochrome c from the mitochondria and/or by binding to the apoptosis-activating factor (APAF-1)	0.514
	BCL2A1	ENSP00000267953	BCL2-related protein A1; Retards apoptosis induced by IL-3 deprivation. May function in the response of hemopoietic cells to external signals and in maintaining endothelial survival during infection (By similarity)	0.444
	BCL2L1	ENSP00000302564	BCL2-like 1; Potent inhibitor of cell death. Inhibits activation of caspases (By similarity). Appears to regulate cell death by blocking the voltage-dependent anion channel (VDAC) by binding to it and preventing the release of the caspase activator, CYC1, from the mitochondrial membrane. Also acts as a regulator of G2 checkpoint and progression to cytokinesis during mitosis	0.49
	BCL2L14	ENSP00000309132	BCL2-like 14 (apoptosis facilitator); Plays a role in apoptosis	0.444
	BOK	ENSP00000314132	BCL2-related ovarian killer; May play a role in apoptosis	0.444
	CASP3	ENSP00000311032	caspase 3, apoptosis-related cysteine peptidase; Involved in the activation cascade of caspases responsible for apoptosis execution. At the onset of apoptosis it proteolytically cleaves poly(ADP-ribose) polymerase (PARP) at a '216-Asp-|-Gly-217' bond. Cleaves and activates sterol regulatory element binding proteins (SREBPs) between the basic helix-loop- helix leucine zipper domain and the membrane attachment domain. Cleaves and activates caspase-6, -7 and -9. Involved in the cleavage of huntingtin. Triggers cell adhesion in sympathetic neurons through RET cleavage	0.739
	CCL17	ENSP00000219244	chemokine (C-C motif) ligand 17; Chemotactic factor for T-lymphocytes but not monocytes or granulocytes. May play a role in T-cell development in thymus and in trafficking and activation of mature T-cells. Binds to CCR4	0.8
	CCL2	ENSP00000225831	chemokine (C-C motif) ligand 2; Chemotactic factor that attracts monocytes and basophils but not neutrophils or eosinophils. Augments monocyte anti-tumor activity. Has been implicated in the pathogenesis of diseases characterized by monocytic infiltrates, like psoriasis, rheumatoid arthritis or atherosclerosis. May be involved in the recruitment of monocytes into the arterial wall during the disease process of atherosclerosis	0.8
	CDK9	ENSP00000362361	cyclin-dependent kinase 9; Protein kinase involved in the regulation of transcription. Member of the cyclin-dependent kinase pair (CDK9/cyclin-T) complex, also called positive transcription elongation factor b (P-TEFb), which facilitates the transition from abortive to productive elongation by phosphorylating the CTD (C-terminal domain) of the large subunit of RNA polymerase II (RNAP II) POLR2A, SUPT5H and RDBP. This complex is inactive when in the 7SK snRNP complex form. Phosphorylates EP300, MYOD1, RPB1/POLR2A and AR, and the negative elongation factors DSIF and NELF. Regulates cytok [...]	0.82
	ENSG00000179066	ENSP00000325519	Uncharacterized protein	0.413
	ENSG00000258643	ENSP00000451320	BCL2L2-PABPN1 readthrough; Promotes cell survival. Blocks dexamethasone-induced apoptosis. Mediates survival of postmitotic Sertoli cells by suppressing death-promoting activity of BAX	0.444

FRAS1	ENSP00000264895	Fraser syndrome 1	0.413
GATA1	ENSP00000365858	GATA binding protein 1 (globin transcription factor 1); Transcriptional activator which probably serves as a general switch factor for erythroid development. It binds to DNA sites with the consensus sequence [AT]GATA[AG] within regulatory regions of globin genes and of other genes expressed in erythroid cells	0.8
HMGB1	ENSP00000343040	high mobility group box 1; DNA binding proteins that associates with chromatin and has the ability to bend DNA. Binds preferentially single-stranded DNA. Involved in V(D)J recombination by acting as a cofactor of the RAG complex. Acts by stimulating cleavage and RAG protein binding at the 23 bp spacer of conserved recombination signal sequences (RSS). Heparin-binding protein that has a role in the extension of neurite-type cytoplasmic processes in developing cells (By similarity)	0.8
HMOX1	ENSP00000216117	heme oxygenase (decycling) 1; Heme oxygenase cleaves the heme ring at the alpha methene bridge to form biliverdin. Biliverdin is subsequently converted to bilirubin by biliverdin reductase. Under physiological conditions, the activity of heme oxygenase is highest in the spleen, where senescent erythrocytes are sequestrated and destroyed	0.8
HRAS	ENSP00000309845	v-Ha-ras Harvey rat sarcoma viral oncogene homolog; Ras proteins bind GDP/GTP and possess intrinsic GTPase activity	0.629
KCNK10	ENSP00000310568	potassium channel, subfamily K, member 10; Outward rectifying potassium channel. Produces rapidly activating and non-inactivating outward rectifier K(+) currents. Activated by arachidonic acid and other naturally occurring unsaturated free fatty acids	0.8
KRAS	ENSP00000256078	v-Ki-ras2 Kirsten rat sarcoma viral oncogene homolog; Ras proteins bind GDP/GTP and possess intrinsic GTPase activity	0.733
MAPK1	ENSP00000215832	mitogen-activated protein kinase 1; Serine/threonine kinase which acts as an essential component of the MAP kinase signal transduction pathway. MAPK1/ERK2 and MAPK3/ERK1 are the 2 MAPKs which play an important role in the MAPK/ERK cascade. They participate also in a signaling cascade initiated by activated KIT and KITLG/SCF. Depending on the cellular context, the MAPK/ERK cascade mediates diverse biological functions such as cell growth, adhesion, survival and differentiation through the regulation of transcription, translation, cytoskeletal rearrangements. The MAPK/ERK cascade plays a [...]	0.7
MAPK3	ENSP00000263025	mitogen-activated protein kinase 3; Serine/threonine kinase which acts as an essential component of the MAP kinase signal transduction pathway. MAPK1/ERK2 and MAPK3/ERK1 are the 2 MAPKs which play an important role in the MAPK/ERK cascade. They participate also in a signaling cascade initiated by activated KIT and KITLG/SCF. Depending on the cellular context, the MAPK/ERK cascade mediates diverse biological functions such as cell growth, adhesion, survival and differentiation through the regulation of transcription, translation, cytoskeletal rearrangements. The MAPK/ERK cascade plays a [...]	0.7
MCL1	ENSP00000358022	myeloid cell leukemia sequence 1 (BCL2-related)	0.803
MDK	ENSP00000352852	midkine (neurite growth-promoting factor 2); Developmentally regulated, secreted growth factor homologous to pleiotrophin (PTN), which has heparin binding activity. Binds anaplastic lymphoma kinase (ALK) which induces ALK activation and subsequent phosphorylation of the insulin receptor substrate (IRS1), followed by the activation of mitogen-activated protein kinase (MAPK) and PI3-kinase, and the induction of cell proliferation. Involved in neointima formation after arterial injury, possibly by mediating leukocyte recruitment. Also involved in early fetal adrenal gland development (By [...]	0.786
MMP9	ENSP00000361405	matrix metallopeptidase 9 (gelatinase B, 92kDa gelatinase, 92kDa type IV collagenase); May play an essential role in local proteolysis of the extracellular matrix and in leukocyte migration. Could play a role in bone osteoclastic resorption. Cleaves KiSS1 at a Gly-|-Leu bond. Cleaves type IV and type V collagen into large C-terminal three quarter fragments and shorter N-terminal one quarter fragments. Degrades fibronectin but not laminin or Pz-peptide	0.84
MUC2	ENSP00000415183	mucin 2, oligomeric mucus/gel-forming; Coats the epithelia of the intestines, airways, and other mucus membrane-containing organs. Thought to provide a protective, lubricating barrier against particles and infectious agents at mucosal surfaces. Major constituent of both the inner and outer mucus layers of the colon and may play a role in excluding bacteria from the inner mucus layer	0.413
MUC5AC	ENSP00000435591	mucin 5AC, oligomeric mucus/gel-forming	0.684
MUC5B	ENSP00000436812	mucin 5B, oligomeric mucus/gel-forming	0.684
MUC6	ENSP00000406861	mucin 6, oligomeric mucus/gel-forming; May provide a mechanism for modulation of the composition of the protective mucus layer related to acid secretion or the presence of bacteria and noxious agents in the lumen. Plays an important role in the cytoprotection of epithelial surfaces and are used as tumor markers in a variety of cancers. May play a role in epithelial organogenesis	0.613
MYC	ENSP00000367207	v-myc myelocytomatosis viral oncogene homolog (avian); Participates in the regulation of gene transcription. Binds DNA in a non-specific manner, yet also specifically recognizes the core sequence 5'-CAC[GA]TG-3'. Seems to activate the transcription of growth-related genes	0.8
NRAS	ENSP00000358548	neuroblastoma RAS viral (v-ras) oncogene homolog; Ras proteins bind GDP/GTP and possess intrinsic GTPase activity	0.629
OTOGL	ENSP00000400895	otogelin-like	0.547
PLSCR1	ENSP00000345494	phospholipid scramblase 1; May mediate accelerated ATP-independent bidirectional transbilayer migration of phospholipids upon binding calcium ions that results in a loss of phospholipid asymmetry in the plasma membrane. May play a central role in the initiation of fibrin clot formation, in the activation of mast cells and in the recognition of apoptotic and injured cells by the reticuloendothelial system	0.8
PTGS2	ENSP00000356438	prostaglandin-endoperoxide synthase 2 (prostaglandin G/H synthase and cyclooxygenase); Mediates the formation of prostaglandins from arachidonate. May have a role as a major mediator of inflammation and/or a role for prostanoid signaling in activity-dependent plasticity	0.861
PTN	ENSP00000341170	pleiotrophin; Secreted growth factor that induces neurite outgrowth and which is mitogenic for fibroblasts, epithelial, and endothelial cells. Binds anaplastic lymphoma kinase (ALK) which induces MAPK pathway activation, an important step in the anti- apoptotic signaling of PTN and regulation of cell proliferation	0.448
TECTA	ENSP00000264037	tectorin alpha; One of the major non-collagenous components of the tectorial membrane (By similarity). The tectorial membrane is an extracellular matrix of the inner ear that covers the neuroepithelium of the cochlea and contacts the stereocilia bundles of specialized sensory hair cells. Sound induces movement of these hair cells relative to the tectorial membrane, deflects the stereocilia and leads to fluctuations in hair-cell membrane potential, transducing sound into electrical signals	0.413
TMPRSS11D	ENSP00000283916	transmembrane protease, serine 11D; May play some biological role in the host defense system on the mucous membrane independently of or in cooperation with other substances in airway mucous or bronchial secretions	0.786
VWCE	ENSP00000334186	von Willebrand factor C and EGF domains; May be a regulatory element in the beta-catenin signaling pathway and a target for chemoprevention of hapatocellular carcinoma	0.413

Table S3. Atopic dermatitis disease of human genes using GeneCards database.

No.	Symbol	Description	Category	GIFtS	GC id	Score ▼
1	FLG	Filaggrin	Protein Coding	40	GC01M152274	121.09
2	ATOD3	Dermatitis, Atopic, 3	Genetic Locus	2	GC20U990080	79.24
3	ATOD5	Dermatitis, Atopic, 5	Genetic Locus	2	GC13U900216	78.13
4	ATOD6	Dermatitis, Atopic, 6	Genetic Locus	2	GC05U990174	78.13
5	ATOD8	Dermatitis, Atopic, 8	Genetic Locus	1	GC04U902095	73.29
6	ATOD7	Dermatitis, Atopic, Susceptibility To, 7	Genetic Locus	1	GC11U901436	72.52
7	ATOD9	Dermatitis, Atopic, 9	Genetic Locus	1	GC03U902459	72.52
8	ATOD1	Dermatitis, Atopic	Genetic Locus	2	GC03U902187	71.12
9	CARD11	Caspase Recruitment Domain Family Member 11	Protein Coding	46	GC07M002906	57.73
10	MIR155	MicroRNA 155	RNA Gene	20	GC21P025573	50.08
11	FLG-AS1	FLG Antisense RNA 1	RNA Gene	17	GC01P152168	41.37
12	IL4	Interleukin 4	Protein Coding	45	GC05P132673	41.02
13	SOCS3	Suppressor Of Cytokine Signaling 3	Protein Coding	42	GC17M078356	40.66
14	IL13	Interleukin 13	Protein Coding	42	GC05P132656	40.47
15	IL4R	Interleukin 4 Receptor	Protein Coding	47	GC16P027659	38.85
16	IGHE	Immunoglobulin Heavy Constant Epsilon	Protein Coding	30	GC14M112095	38.65
17	IFNG	Interferon Gamma	Protein Coding	47	GC12M068154	36.92
18	IL5	Interleukin 5	Protein Coding	44	GC05M132541	35.91
19	MS4A2	Membrane Spanning 4-Domains A2	Protein Coding	41	GC11P060088	35.44
20	TNF	Tumor Necrosis Factor	Protein Coding	50	GC06P076864	35.26
21	IL10	Interleukin 10	Protein Coding	46	GC01M206767	34.62
22	SPINK5	Serine Peptidase Inhibitor Kazal Type 5	Protein Coding	42	GC05P148025	34.47
23	RNASE3	Ribonuclease A Family Member 3	Protein Coding	40	GC14P020891	31.41
24	CCL11	C-C Motif Chemokine Ligand 11	Protein Coding	42	GC17P034285	30.58
25	NOD2	Nucleotide Binding Oligomerization Domain Containing 2	Protein Coding	47	GC16P050693	30.18
26	IL31	Interleukin 31	Protein Coding	35	GC12M122173	29.52
27	TSLP	Thymic Stromal Lymphopoietin	Protein Coding	38	GC05P111070	28.94
28	CCL17	C-C Motif Chemokine Ligand 17	Protein Coding	38	GC16P057411	28.5
29	JAK1	Janus Kinase 1	Protein Coding	52	GC01M064833	26.67
30	FOXP3	Forkhead Box P3	Protein Coding	46	GC0XM049250	26.58
31	CCL22	C-C Motif Chemokine Ligand 22	Protein Coding	37	GC16P057359	26.41
32	CXCL8	C-X-C Motif Chemokine Ligand 8	Protein Coding	40	GC04P073740	26.29
33	IL31RA	Interleukin 31 Receptor A	Protein Coding	43	GC05P055840	25.35
34	IL2	Interleukin 2	Protein Coding	44	GC04M122451	25.26
35	PGM3	Phosphoglucomutase 3	Protein Coding	43	GC06M083161	24.98
36	IL6	Interleukin 6	Protein Coding	48	GC07P022725	24.67
37	IL2RA	Interleukin 2 Receptor Subunit Alpha	Protein Coding	49	GC10M006010	24.46
38	IL18	Interleukin 18	Protein Coding	42	GC11M112143	24.26
39	CCL5	C-C Motif Chemokine Ligand 5	Protein Coding	42	GC17M035871	23.97
40	IL17A	Interleukin 17A	Protein Coding	41	GC06P052186	23.96
41	CTLA4	Cytotoxic T-Lymphocyte Associated Protein 4	Protein Coding	44	GC02P203867	23.91
42	MSMO1	Methylsterol Monooxygenase 1	Protein Coding	42	GC04P165327	23.87
43	CCR4	C-C Motif Chemokine Receptor 4	Protein Coding	44	GC03P032951	23.75
44	TLR2	Toll Like Receptor 2	Protein Coding	50	GC04P153684	23.67
45	IL1B	Interleukin 1 Beta	Protein Coding	46	GC02M112829	23.18
46	IL6R	Interleukin 6 Receptor	Protein Coding	48	GC01P154405	22.96
47	GATA3	GATA Binding Protein 3	Protein Coding	48	GC10P008045	22.5
48	IL33	Interleukin 33	Protein Coding	38	GC09P006350	22.27
49	HLA-DRB1	Major Histocompatibility Complex, Class II, DR Beta 1	Protein Coding	46	GC06M032578	21.82
50	STAT3	Signal Transducer And Activator Of Transcription 3	Protein Coding	50	GC17M042313	21.67
51	CCL27	C-C Motif Chemokine Ligand 27	Protein Coding	35	GC09M034662	21.65
52	KLK7	Kallikrein Related Peptidase 7	Protein Coding	40	GC19M062303	21.62
53	ZNF750	Zinc Finger Protein 750	Protein Coding	36	GC17M082829	21.61
54	ICAM1	Intercellular Adhesion Molecule 1	Protein Coding	49	GC19P010310	21.59
55	IGES	Immunoglobulin E Concentration, Serum	Genetic Locus	4	GC05U990033	21.42
56	IL2RB	Interleukin 2 Receptor Subunit Beta	Protein Coding	48	GC22M037125	21.4
57	STAT1	Signal Transducer And Activator Of Transcription 1	Protein Coding	51	GC02M190908	21.24
58	IL16	Interleukin 16	Protein Coding	40	GC15P081159	21.04
59	CXCR3	C-X-C Motif Chemokine Receptor 3	Protein Coding	44	GC0XM071615	20.99
60	DEFB4A	Defensin Beta 4A	Protein Coding	34	GC08P007895	20.9
61	DSG1	Desmoglein 1	Protein Coding	43	GC18P031318	20.74
62	CSF2	Colony Stimulating Factor 2	Protein Coding	42	GC05P132073	20.74
63	FCER2	Fc Epsilon Receptor II	Protein Coding	43	GC19M007689	20.58
64	PHF11	PHD Finger Protein 11	Protein Coding	36	GC13P049495	20.4
65	LORICRIN	Loricrin Cornified Envelope Precursor Protein	Protein Coding	28	GC01P153262	20.37
66	TLR4	Toll Like Receptor 4	Protein Coding	50	GC09P117704	20
67	IL1A	Interleukin 1 Alpha	Protein Coding	43	GC02M112773	19.88
68	CDSN	Corneodesmosin	Protein Coding	42	GC06M031115	19.79
69	CCR3	C-C Motif Chemokine Receptor 3	Protein Coding	45	GC03P046276	19.56
70	SELE	Selectin E	Protein Coding	42	GC01M169722	19.55
71	IL9	Interleukin 9	Protein Coding	42	GC05M135891	19.37
72	CCL2	C-C Motif Chemokine Ligand 2	Protein Coding	47	GC17P034255	19.1
73	STAT6	Signal Transducer And Activator Of Transcription 6	Protein Coding	49	GC12M057095	18.79
74	IVL	Involucrin	Protein Coding	38	GC01P152881	18.57
75	CMA1	Chymase 1	Protein Coding	43	GC14M024506	18.56
76	HLA-DQB1	Major Histocompatibility Complex, Class II, DQ Beta 1	Protein Coding	43	GC06M061358	18.44
77	NGF	Nerve Growth Factor	Protein Coding	49	GC01M115285	18.42
78	CCL18	C-C Motif Chemokine Ligand 18	Protein Coding	35	GC17P036064	18.38
79	SELL	Selectin L	Protein Coding	42	GC01M169690	18.29
80	CCR6	C-C Motif Chemokine Receptor 6	Protein Coding	44	GC06P167111	18.14
81	CXCL10	C-X-C Motif Chemokine Ligand 10	Protein Coding	43	GC04M076021	17.94
82	IL21R	Interleukin 21 Receptor	Protein Coding	41	GC16P027663	17.92
83	PLA2G7	Phospholipase A2 Group VII	Protein Coding	49	GC06M061586	17.67

84	FOXN1	Forkhead Box N1	Protein Coding	41	GC17P028506	17.54
85	FAF2	Fas Associated Factor Family Member 2	Protein Coding	37	GC05P176447	17.53
86	TMEM79	Transmembrane Protein 79	Protein Coding	35	GC01P156252	17.45
87	TGM3	Transglutaminase 3	Protein Coding	42	GC20P002296	17.35
88	CSN1S1	Casein Alpha S1	Protein Coding	35	GC04P069932	17.15
89	PRG2	Proteoglycan 2, Pro Eosinophil Major Basic Protein	Protein Coding	40	GC11M057386	17.07
90	VCAM1	Vascular Cell Adhesion Molecule 1	Protein Coding	45	GC01P100719	17.07
91	EPX	Eosinophil Peroxidase	Protein Coding	44	GC17P058192	16.94
92	TACR1	Tachykinin Receptor 1	Protein Coding	45	GC02M075047	16.85
93	HRH1	Histamine Receptor H1	Protein Coding	45	GC03P012069	16.32
94	CAMP	Cathelicidin Antimicrobial Peptide	Protein Coding	40	GC03P048674	16.25
95	BDNF	Brain Derived Neurotrophic Factor	Protein Coding	46	GC11M027654	16.22
96	CD4	CD4 Molecule	Protein Coding	48	GC12P006786	16.18
97	CCL26	C-C Motif Chemokine Ligand 26	Protein Coding	37	GC07M075769	16.13
98	CD8A	CD8a Molecule	Protein Coding	45	GC02M086784	16.08
99	CXCL9	C-X-C Motif Chemokine Ligand 9	Protein Coding	38	GC04M076001	16.01
100	LTA	Lymphotoxin Alpha	Protein Coding	41	GC06P076862	15.94
101	S100A7	S100 Calcium Binding Protein A7	Protein Coding	40	GC01M153457	15.86
102	HAVCR1	Hepatitis A Virus Cellular Receptor 1	Protein Coding	41	GC05M157028	15.64
103	PDE4A	Phosphodiesterase 4A	Protein Coding	45	GC19P010416	15.59
104	ZNF341	Zinc Finger Protein 341	Protein Coding	36	GC20P033731	15.58
105	CYP4F22	Cytochrome P450 Family 4 Subfamily F Member 22	Protein Coding	38	GC19P015508	15.38
106	ITGAM	Integrin Subunit Alpha M	Protein Coding	46	GC16P039480	15.36
107	IL12RB1	Interleukin 12 Receptor Subunit Beta 1	Protein Coding	44	GC19M018058	15.28
108	NPSR1	Neuropeptide S Receptor 1	Protein Coding	40	GC07P034664	15.26
109	HRH4	Histamine Receptor H4	Protein Coding	45	GC18P024460	15.25
110	TLR9	Toll Like Receptor 9	Protein Coding	45	GC03M052222	15.23
111	IL7	Interleukin 7	Protein Coding	41	GC08M078689	15.18
112	PTGDR2	Prostaglandin D2 Receptor 2	Protein Coding	41	GC11M060850	15.18
113	DEFB103B	Defensin Beta 103B	Protein Coding	28	GC08M007430	15.17
114	IL7R	Interleukin 7 Receptor	Protein Coding	45	GC05P035852	14.88
115	FCGR3A	Fc Gamma Receptor IIIa	Protein Coding	44	GC01M161541	14.85
116	IL1RL1	Interleukin 1 Receptor Like 1	Protein Coding	41	GC02P102294	14.74
117	MIF	Macrophage Migration Inhibitory Factor	Protein Coding	47	GC22P023894	14.74
118	CCL20	C-C Motif Chemokine Ligand 20	Protein Coding	42	GC02P227835	14.7
119	TIMP1	TIMP Metallopeptidase Inhibitor 1	Protein Coding	43	GC0XP047583	14.67
120	CD69	CD69 Molecule	Protein Coding	41	GC12M019779	14.65
121	TMPO	Thymopoietin	Protein Coding	45	GC12P098515	14.64
122	DOCK8	Dedicator Of Cytokinesis 8	Protein Coding	43	GC09P000214	14.59
123	SELP	Selectin P	Protein Coding	45	GC01M169558	14.54
124	DOP1A	DOP1 Leucine Zipper Like Protein A	Protein Coding	25	GC06P083068	14.48
125	LALBA	Lactalbumin Alpha	Protein Coding	39	GC12M048567	14.48
126	CRLF2	Cytokine Receptor Like Factor 2	Protein Coding	38	GC0XM001190	14.3
127	F2RL1	F2R Like Trypsin Receptor 1	Protein Coding	45	GC05P076818	14.29
128	CHI3L1	Chitinase 3 Like 1	Protein Coding	44	GC01M203148	14.21
129	CYSLTR1	Cysteinyl Leukotriene Receptor 1	Protein Coding	42	GC0XM078271	14.2
130	PSIP1	PC4 And SFRS1 Interacting Protein 1	Protein Coding	39	GC09M015464	14.1
131	CCL7	C-C Motif Chemokine Ligand 7	Protein Coding	41	GC17P034270	14.08
132	TNFRSF8	TNF Receptor Superfamily Member 8	Protein Coding	42	GC01P012063	14.02
133	CD40	CD40 Molecule	Protein Coding	47	GC20P046118	13.98
134	PPBP	Pro-Platelet Basic Protein	Protein Coding	42	GC04M073986	13.96
135	CSTA	Cystatin A	Protein Coding	44	GC03P122325	13.93
136	IRAK3	Interleukin 1 Receptor Associated Kinase 3	Protein Coding	46	GC12P066242	13.92
137	IL10RA	Interleukin 10 Receptor Subunit Alpha	Protein Coding	45	GC11P117987	13.83
138	PTGDR	Prostaglandin D2 Receptor	Protein Coding	46	GC14P052267	13.81
139	DSP	Desmoplakin	Protein Coding	49	GC06P007541	13.79
140	CARMIL2	Capping Protein Regulator And Myosin 1 Linker 2	Protein Coding	29	GC16P067644	13.76
141	HLA-DQA1	Major Histocompatibility Complex, Class II, DQ Alpha 1	Protein Coding	43	GC06P076895	13.75
142	CCL4	C-C Motif Chemokine Ligand 4	Protein Coding	38	GC17P036103	13.67
143	TPMT	Thiopurine S-Methyltransferase	Protein Coding	46	GC06M018128	13.63
144	VIP	Vasoactive Intestinal Peptide	Protein Coding	44	GC06P152750	13.53
145	KITLG	KIT Ligand	Protein Coding	44	GC12M088492	13.46
146	TAC1	Tachykinin Precursor 1	Protein Coding	42	GC07P097731	13.44
147	NTF3	Neurotrophin 3	Protein Coding	42	GC12P005432	13.4
148	CCL13	C-C Motif Chemokine Ligand 13	Protein Coding	36	GC17P034356	13.25
149	SYK	Spleen Associated Tyrosine Kinase	Protein Coding	49	GC09P091849	13.23
150	CCL1	C-C Motif Chemokine Ligand 1	Protein Coding	37	GC17M034441	13.21
151	BTD	Biotinidase	Protein Coding	44	GC03P016758	13.07
152	KCNJ11	Potassium Inwardly Rectifying Channel Subfamily J Member 11	Protein Coding	47	GC11M017520	12.97
153	IL17D	Interleukin 17D	Protein Coding	37	GC13P020702	12.91
154	CX3CR1	C-X3-C Motif Chemokine Receptor 1	Protein Coding	43	GC03M039279	12.88
155	CTSE	Cathepsin E	Protein Coding	41	GC01M206009	12.85
156	IL3	Interleukin 3	Protein Coding	42	GC05P132060	12.84
157	HLA-B	Major Histocompatibility Complex, Class I, B	Protein Coding	46	GC06M061261	12.7
158	CSN3	Casein Kappa	Protein Coding	36	GC04P070242	12.51
159	CD86	CD86 Molecule	Protein Coding	42	GC03P122055	12.33
160	FCGR1A	Fc Gamma Receptor Ia	Protein Coding	41	GC01P149788	12.08
161	CD14	CD14 Molecule	Protein Coding	45	GC05M140631	11.9
162	TXK	TXK Tyrosine Kinase	Protein Coding	45	GC04M048126	11.88
163	CCR10	C-C Motif Chemokine Receptor 10	Protein Coding	36	GC17M042678	11.71
164	CFTR	CF Transmembrane Conductance Regulator	Protein Coding	50	GC07P117287	11.64
165	CD83	CD83 Molecule	Protein Coding	40	GC06P014117	11.58
166	PSMB9	Proteasome 20S Subunit Beta 9	Protein Coding	45	GC06P076897	11.52
167	LRRC32	Leucine Rich Repeat Containing 32	Protein Coding	40	GC11M076657	11.5
168	SERPINB4	Serpin Family B Member 4	Protein Coding	36	GC18M063637	11.25
169	CCR7	C-C Motif Chemokine Receptor 7	Protein Coding	44	GC17M041052	11.19
170	NAT2	N-Acetyltransferase 2	Protein Coding	42	GC08P018391	11.09

171	BRAF	B-Raf Proto-Oncogene, Serine/Threonine Kinase	Protein Coding	52	GC07M140730	11.07
172	ICOSLG	Inducible T Cell Costimulator Ligand	Protein Coding	39	GC21M044222	11.01
173	IL22	Interleukin 22	Protein Coding	41	GC12M068248	10.97
174	ICAM3	Intercellular Adhesion Molecule 3	Protein Coding	42	GC19M010388	10.92
175	KRT1	Keratin 1	Protein Coding	46	GC12M052674	10.82
176	BCL7B	BAF Chromatin Remodeling Complex Subunit BCL7B	Protein Coding	37	GC07M073536	10.62
177	CCL3	C-C Motif Chemokine Ligand 3	Protein Coding	38	GC17M036088	10.4
178	ADAM33	ADAM Metallopeptidase Domain 33	Protein Coding	38	GC20M003669	10.36
179	HLCS	Holocarboxylase Synthetase	Protein Coding	42	GC21M036750	10.35
180	TGFB1	Transforming Growth Factor Beta 1	Protein Coding	50	GC19M041301	10.12
181	DEFB103A	Defensin Beta 103A	Protein Coding	27	GC08P007881	10.09
182	KRT16	Keratin 16	Protein Coding	42	GC17M041609	10.07
183	ALOX5	Arachidonate 5-Lipoxygenase	Protein Coding	48	GC10P045374	9.91
184	IFIH1	Interferon Induced With Helicase C Domain 1	Protein Coding	45	GC02M162267	9.75
185	CD79A	CD79a Molecule	Protein Coding	46	GC19P041877	9.72
186	IL6ST	Interleukin 6 Cytokine Family Signal Transducer	Protein Coding	48	GC05M055935	9.71
187	RNASE2	Ribonuclease A Family Member 2	Protein Coding	40	GC14P030645	9.7
188	CARD11-AS1	CARD11 Antisense RNA 1	RNA Gene	10	GC07P003048	9.6
189	IL12B	Interleukin 12B	Protein Coding	43	GC05M159314	9.42
190	NACA	Nascent Polypeptide Associated Complex Subunit Alpha	Protein Coding	37	GC12M056712	9.39
191	IL17F	Interleukin 17F	Protein Coding	42	GC06M061661	9.17
192	IL1R1	Interleukin 1 Receptor Type 1	Protein Coding	45	GC02P102136	9.14
193	HNMT	Histamine N-Methyltransferase	Protein Coding	45	GC02P137964	8.79
194	COL6A5	Collagen Type VI Alpha 5 Chain	Protein Coding	33	GC03P130345	8.64
195	MMP9	Matrix Metallopeptidase 9	Protein Coding	53	GC20P046008	8.58
196	CALCA	Calcitonin Related Polypeptide Alpha	Protein Coding	44	GC11M014945	8.54
197	DSC1	Desmocollin 1	Protein Coding	39	GC18M031129	8.54
198	EGFR	Epidermal Growth Factor Receptor	Protein Coding	52	GC07P055019	8.38
199	SCNN1A	Sodium Channel Epithelial 1 Subunit Alpha	Protein Coding	47	GC12M006346	8.26
200	HLA-A	Major Histocompatibility Complex, Class I, A	Protein Coding	46	GC06P076821	8.25
201	SOCS1	Suppressor Of Cytokine Signaling 1	Protein Coding	43	GC16M011783	8.16
202	TGM1	Transglutaminase 1	Protein Coding	45	GC14M024249	8.1
203	OSMR	Oncostatin M Receptor	Protein Coding	45	GC05P038845	8.08
204	CD28	CD28 Molecule	Protein Coding	45	GC02P203706	8.05
205	GSTM1	Glutathione S-Transferase Mu 1	Protein Coding	41	GC01P109687	7.98
206	SCNN1B	Sodium Channel Epithelial 1 Subunit Beta	Protein Coding	48	GC16P023278	7.94
207	HSPA9	Heat Shock Protein Family A (Hsp70) Member 9	Protein Coding	46	GC05M138554	7.94
208	RRAS2	RAS Related 2	Protein Coding	46	GC11M014299	7.94
209	SCNN1G	Sodium Channel Epithelial 1 Subunit Gamma	Protein Coding	46	GC16P023182	7.94
210	NEK9	NIMA Related Kinase 9	Protein Coding	45	GC14M075079	7.94
211	MORC2	MORC Family CW-Type Zinc Finger 2	Protein Coding	40	GC22M030925	7.94
212	NLRP3	NLR Family Pyrin Domain Containing 3	Protein Coding	46	GC01P247415	7.9
213	FCGR3B	Fc Gamma Receptor IIIb	Protein Coding	43	GC01M161623	7.88
214	TBX21	T-Box Transcription Factor 21	Protein Coding	45	GC17P047733	7.8
215	CD1D	CD1d Molecule	Protein Coding	42	GC01P158178	7.8
216	GJB2	Gap Junction Protein Beta 2	Protein Coding	46	GC13M020187	7.75
217	GZMB	Granzyme B	Protein Coding	46	GC14M024630	7.68
218	IL15	Interleukin 15	Protein Coding	40	GC04P141636	7.67
219	KIT	KIT Proto-Oncogene, Receptor Tyrosine Kinase	Protein Coding	52	GC04P054657	7.65
220	ALB	Albumin	Protein Coding	49	GC04P073397	7.62
221	FCER1A	Fc Epsilon Receptor Ia	Protein Coding	41	GC01P159259	7.61
222	MMP1	Matrix Metallopeptidase 1	Protein Coding	51	GC11M102810	7.45
223	HLA-G	Major Histocompatibility Complex, Class I, G	Protein Coding	44	GC06P076812	7.4
224	CXCL11	C-X-C Motif Chemokine Ligand 11	Protein Coding	40	GC04M076033	7.39
225	GSTP1	Glutathione S-Transferase Pi 1	Protein Coding	49	GC11P067583	7.27
226	CD40LG	CD40 Ligand	Protein Coding	47	GC0XP136649	7.27
227	IL1RN	Interleukin 1 Receptor Antagonist	Protein Coding	48	GC02P120921	7.26
228	HLA-C	Major Histocompatibility Complex, Class I, C	Protein Coding	45	GC06M061260	7.25
229	FLG2	Filaggrin 2	Protein Coding	37	GC01M152321	7.25
230	TP63	Tumor Protein P63	Protein Coding	47	GC03P189598	7.22
231	ADRB2	Adrenoceptor Beta 2	Protein Coding	48	GC05P148825	7.22
232	AMBP	Alpha-1-Microglobulin/Bikunin Precursor	Protein Coding	42	GC09M114060	7.14
233	NCKAP1L	NCK Associated Protein 1 Like	Protein Coding	38	GC12P054497	7.14
234	IFNGR1	Interferon Gamma Receptor 1	Protein Coding	49	GC06M137197	7.13
235	ITGAX	Integrin Subunit Alpha X	Protein Coding	44	GC16P039491	7.11
236	ICOS	Inducible T Cell Costimulator	Protein Coding	42	GC02P203937	7.01
237	RMRP	RNA Component Of Mitochondrial RNA Processing Endoribonuclease	RNA Gene	26	GC09M035655	7.01
238	LCE3B	Late Cornified Envelope 3B	Protein Coding	26	GC01P152613	6.98
239	LCE3C	Late Cornified Envelope 3C	Protein Coding	27	GC01P152600	6.88
240	KRAS	KRAS Proto-Oncogene, GTPase	Protein Coding	49	GC12M025204	6.8
241	LBR	Lamin B Receptor	Protein Coding	46	GC01M225401	6.75
242	SLCO2A1	Solute Carrier Organic Anion Transporter Family Member 2A1	Protein Coding	42	GC03M133932	6.74
243	CCR5	C-C Motif Chemokine Receptor 5	Protein Coding	45	GC03P046383	6.73
244	AHR	Aryl Hydrocarbon Receptor	Protein Coding	48	GC07P016916	6.73
245	AK2	Adenylate Kinase 2	Protein Coding	47	GC01M033007	6.68
246	IL37	Interleukin 37	Protein Coding	37	GC02P120918	6.66
247	LCE5A	Late Cornified Envelope 5A	Protein Coding	25	GC01P152510	6.62
248	TAP1	Transporter 1, ATP Binding Cassette Subfamily B Member	Protein Coding	46	GC06M061365	6.62
249	TOR1B	Torsin Family 1 Member B	Protein Coding	39	GC09P129803	6.6
250	CCL24	C-C Motif Chemokine Ligand 24	Protein Coding	36	GC07M076562	6.59
251	CSF1	Colony Stimulating Factor 1	Protein Coding	43	GC01P109911	6.52
252	NCR2	Natural Cytotoxicity Triggering Receptor 2	Protein Coding	39	GC06P077045	6.5
253	TOM1	Target Of Myb1 Membrane Trafficking Protein	Protein Coding	42	GC22P035299	6.49
254	DYNLT1	Dynein Light Chain Tctex-Type 1	Protein Coding	37	GC06M158636	6.42
255	IL26	Interleukin 26	Protein Coding	34	GC12M068201	6.41
256	MAP2K1	Mitogen-Activated Protein Kinase Kinase 1	Protein Coding	52	GC15P066386	6.38
257	GFRA4	GDNF Family Receptor Alpha 4	Protein Coding	34	GC20M003659	6.38

258	DEFB125	Defensin Beta 125	Protein Coding	32	GC20P000087	6.38
259	ASAH2B	N-Acylsphingosine Amidohydrolase 2B	Protein Coding	29	GC10P050739	6.38
260	DEFB124	Defensin Beta 124	Protein Coding	29	GC20M031465	6.38
261	DEFB127	Defensin Beta 127	Protein Coding	29	GC20P000157	6.38
262	DEFB132	Defensin Beta 132	Protein Coding	28	GC20P000257	6.38
263	DEFB128	Defensin Beta 128	Protein Coding	24	GC20M000187	6.38
264	DEFB1	Defensin Beta 1	Protein Coding	39	GC08M006870	6.34
265	GBA	Glucosylceramidase Beta	Protein Coding	48	GC01M155234	6.27
266	INS	Insulin	Protein Coding	46	GC11M002159	6.26
267	MPO	Myeloperoxidase	Protein Coding	50	GC17M058269	6.22
268	HFE	Homeostatic Iron Regulator	Protein Coding	44	GC06P026087	6.21
269	CCL21	C-C Motif Chemokine Ligand 21	Protein Coding	41	GC09M034709	6.2
270	TNFRSF1B	TNF Receptor Superfamily Member 1B	Protein Coding	46	GC01P012167	6.18
271	STAT4	Signal Transducer And Activator Of Transcription 4	Protein Coding	45	GC02M191029	6.17
272	SCGB3A2	Secretoglobin Family 3A Member 2	Protein Coding	39	GC05P147870	6.11
273	CARD14	Caspase Recruitment Domain Family Member 14	Protein Coding	42	GC17P080170	6.08
274	C4A	Complement C4A (Rodgers Blood Group)	Protein Coding	44	GC06P076888	6.07
275	IL21	Interleukin 21	Protein Coding	42	GC04M122612	6.06
276	C5	Complement C5	Protein Coding	46	GC09M120952	6.01
277	UROD	Uroporphyrinogen Decarboxylase	Protein Coding	44	GC01P045270	6.01
278	ASRT3	Asthma-Related Traits, Susceptibility To, 3	Genetic Locus	1	GC02U901663	6.01
279	ASRT4	Asthma-Related Traits, Susceptibility To, 4	Genetic Locus	1	GC01U902370	6.01
280	ASRT6	Asthma-Related Traits, Susceptibility To, 6	Genetic Locus	1	GC17U901148	6.01
281	ACE	Angiotensin I Converting Enzyme	Protein Coding	50	GC17P063477	6
282	TGM2	Transglutaminase 2	Protein Coding	46	GC20M038127	5.93
283	DEL18Q	Chromosome 18q Deletion Syndrome	Genetic Locus	1	GC18U900453	5.93
284	HPGD	15-Hydroxyprostaglandin Dehydrogenase	Protein Coding	46	GC04M174490	5.89
285	LRP1	LDL Receptor Related Protein 1	Protein Coding	48	GC12P057128	5.89
286	NOD1	Nucleotide Binding Oligomerization Domain Containing 1	Protein Coding	43	GC07M030424	5.89
287	HRNR	Hornerin	Protein Coding	36	GC01M152184	5.84
288	TNFSF4	TNF Superfamily Member 4	Protein Coding	40	GC01M173183	5.81
289	MBTPS2	Membrane Bound Transcription Factor Peptidase, Site 2	Protein Coding	42	GC0XP021839	5.81
290	GSTT1	Glutathione S-Transferase Theta 1	Protein Coding	33	GC22Mi00270	5.74
291	LRBA	LPS Responsive Beige-Like Anchor Protein	Protein Coding	41	GC04M150264	5.73
292	ELN	Elastin	Protein Coding	44	GC07P074027	5.72
293	POSTN	Periostin	Protein Coding	44	GC13M037562	5.7
294	IL12RB2	Interleukin 12 Receptor Subunit Beta 2	Protein Coding	44	GC01P067307	5.69
295	NLRP10	NLR Family Pyrin Domain Containing 10	Protein Coding	38	GC11M007959	5.68
296	S100A8	S100 Calcium Binding Protein A8	Protein Coding	42	GC01M153391	5.67
297	HAVCR2	Hepatitis A Virus Cellular Receptor 2	Protein Coding	44	GC05M157063	5.67
298	C4B	Complement C4B (Chido Blood Group)	Protein Coding	44	GC06P032014	5.65
299	TAP2	Transporter 2, ATP Binding Cassette Subfamily B Member	Protein Coding	44	GC06M032821	5.61
300	EDN1	Endothelin 1	Protein Coding	48	GC06P012256	5.59
301	IDO1	Indoleamine 2,3-Dioxygenase 1	Protein Coding	44	GC08P039891	5.59
302	HAO1	Hydroxyacid Oxidase 1	Protein Coding	41	GC20M007913	5.54
303	PACRG	Parkin Coregulated	Protein Coding	38	GC06P162727	5.54
304	KIR3DL3	Killer Cell Immunoglobulin Like Receptor, Three Ig Domains And Long Cytoplasmic Tail 3	Protein Coding	35	GC19P062909	5.54
305	VEGFA	Vascular Endothelial Growth Factor A	Protein Coding	47	GC06P043770	5.52
306	DCD	Dermcidin	Protein Coding	37	GC12M054644	5.51
307	CD5	CD5 Molecule	Protein Coding	41	GC11P061114	5.46
308	IL13RA1	Interleukin 13 Receptor Subunit Alpha 1	Protein Coding	41	GC0XP118727	5.45
309	TNFAIP3	TNF Alpha Induced Protein 3	Protein Coding	47	GC06P137866	5.42
310	FBN1	Fibrillin 1	Protein Coding	46	GC15M048408	5.41
311	IL5RA	Interleukin 5 Receptor Subunit Alpha	Protein Coding	45	GC03M003066	5.39
312	GUSB	Glucuronidase Beta	Protein Coding	47	GC07M065960	5.38
313	MBL2	Mannose Binding Lectin 2	Protein Coding	46	GC10M052760	5.38
314	NLRC4	NLR Family CARD Domain Containing 4	Protein Coding	44	GC02M032224	5.35
315	NCF1	Neutrophil Cytosolic Factor 1	Protein Coding	46	GC07P074773	5.34
316	IL23A	Interleukin 23 Subunit Alpha	Protein Coding	37	GC12P057101	5.33
317	WDR61	WD Repeat Domain 61	Protein Coding	37	GC15M078277	5.31
318	OVOL1	Ovo Like Transcriptional Repressor 1	Protein Coding	35	GC11P065787	5.3
319	KIF3A	Kinesin Family Member 3A	Protein Coding	40	GC05M132689	5.27
320	TBC1D4	TBC1 Domain Family Member 4	Protein Coding	43	GC13M075284	5.27
321	PI3	Peptidase Inhibitor 3	Protein Coding	38	GC20P045174	5.27
322	PSMB8	Proteasome 20S Subunit Beta 8	Protein Coding	49	GC06M032840	5.25
323	SCGB1A1	Secretoglobin Family 1A Member 1	Protein Coding	40	GC11P062405	5.25
324	HRAS	HRas Proto-Oncogene, GTPase	Protein Coding	50	GC11M002647	5.25
325	TNFRSF18	TNF Receptor Superfamily Member 18	Protein Coding	41	GC01M001203	5.22
326	DPP4	Dipeptidyl Peptidase 4	Protein Coding	49	GC02M161992	5.18
327	AQP3	Aquaporin 3 (Gill Blood Group)	Protein Coding	46	GC09M033431	5.18
328	IRAK1	Interleukin 1 Receptor Associated Kinase 1	Protein Coding	48	GC0XM154010	5.18
329	BCL11B	BAF Chromatin Remodeling Complex Subunit BCL11B	Protein Coding	42	GC14M099169	5.17
330	IL18R1	Interleukin 18 Receptor 1	Protein Coding	42	GC02P102311	5.16
331	ITGAL	Integrin Subunit Alpha L	Protein Coding	45	GC16P030472	5.15
332	PNPLA1	Patatin Like Phospholipase Domain Containing 1	Protein Coding	37	GC06P077006	5.15
333	PLAU	Plasminogen Activator, Urokinase	Protein Coding	50	GC10P073909	5.1
334	MAPK1	Mitogen-Activated Protein Kinase 1	Protein Coding	50	GC22M021759	5.07
335	KLRB1	Killer Cell Lectin Like Receptor B1	Protein Coding	40	GC12M019777	5.07
336	KLRG1	Killer Cell Lectin Like Receptor G1	Protein Coding	37	GC12P008950	5.07
337	NRAS	NRAS Proto-Oncogene, GTPase	Protein Coding	48	GC01M114704	5.02
338	PF4	Platelet Factor 4	Protein Coding	40	GC04M073980	5.01
339	TLR6	Toll Like Receptor 6	Protein Coding	43	GC04M038828	5.01
340	EMSY	EMSY Transcriptional Repressor, BRCA2 Interacting	Protein Coding	32	GC11P077480	5.01
341	HMGB1	High Mobility Group Box 1	Protein Coding	44	GC13M030456	5.01
342	MIR148B	MicroRNA 148b	RNA Gene	21	GC12P054337	5
343	IGF1	Insulin Like Growth Factor 1	Protein Coding	48	GC12M102395	5
344	GP1BB	Glycoprotein Ib Platelet Subunit Beta	Protein Coding	42	GC22P033260	5

345	IGKC	Immunoglobulin Kappa Constant	Protein Coding	34	GC02M090708	4.99
346	IGHG2	Immunoglobulin Heavy Constant Gamma 2 (G2m Marker)	Protein Coding	30	GC14M112097	4.99
347	HLA-DMA	Major Histocompatibility Complex, Class II, DM Alpha	Protein Coding	41	GC06M061371	4.94
348	POMC	Proopiomelanocortin	Protein Coding	48	GC02M025160	4.93
349	CD1A	CD1a Molecule	Protein Coding	41	GC01P158255	4.93
350	CIITA	Class II Major Histocompatibility Complex Transactivator	Protein Coding	44	GC16P010880	4.9
351	HLA-DPB1	Major Histocompatibility Complex, Class II, DP Beta 1	Protein Coding	45	GC06P076901	4.86
352	FCGR2A	Fc Gamma Receptor IIa	Protein Coding	45	GC01P161505	4.83
353	CRH	Corticotropin Releasing Hormone	Protein Coding	44	GC08M066176	4.82
354	TLR1	Toll Like Receptor 1	Protein Coding	46	GC04M038793	4.79
355	TBXA2R	Thromboxane A2 Receptor	Protein Coding	48	GC19M003594	4.79
356	MAP2K2	Mitogen-Activated Protein Kinase Kinase 2	Protein Coding	52	GC19M004090	4.76
357	TRAF3IP2	TRAF3 Interacting Protein 2	Protein Coding	45	GC06M111555	4.75
358	MUC7	Mucin 7, Secreted	Protein Coding	37	GC04P070430	4.75
359	NCF4	Neutrophil Cytosolic Factor 4	Protein Coding	47	GC22P036860	4.74
360	IL25	Interleukin 25	Protein Coding	37	GC14P030866	4.71
361	WAS	WASP Actin Nucleation Promoting Factor	Protein Coding	47	GC0XP048676	4.71
362	VDR	Vitamin D Receptor	Protein Coding	50	GC12M047841	4.68
363	IRF2	Interferon Regulatory Factor 2	Protein Coding	43	GC04M184387	4.67
364	ORMDL3	ORMDL Sphingolipid Biosynthesis Regulator 3	Protein Coding	40	GC17M039921	4.64
365	CASP1	Caspase 1	Protein Coding	49	GC11M105025	4.62
366	CD1C	CD1c Molecule	Protein Coding	39	GC01P158289	4.61
367	CASP3	Caspase 3	Protein Coding	48	GC04M184627	4.59
368	MIR628	MicroRNA 628	RNA Gene	18	GC15M055372	4.56
369	BRCA2	BRCA2 DNA Repair Associated	Protein Coding	48	GC13P032315	4.56
370	COL7A1	Collagen Type VII Alpha 1 Chain	Protein Coding	44	GC03M048564	4.54
371	ABCC9	ATP Binding Cassette Subfamily C Member 9	Protein Coding	45	GC12M021797	4.52
372	LEP	Leptin	Protein Coding	46	GC07P128241	4.49
373	CD7	CD7 Molecule	Protein Coding	39	GC17M082314	4.48
374	ZAP70	Zeta Chain Of T Cell Receptor Associated Protein Kinase 70	Protein Coding	50	GC02P097734	4.48
375	CD207	CD207 Molecule	Protein Coding	39	GC02M070830	4.46
376	MUC5AC	Mucin 5AC, Oligomeric Mucus/Gel-Forming	Protein Coding	39	GC11P001581	4.45
377	SEMA3A	Semaphorin 3A	Protein Coding	45	GC07M083955	4.43
378	MMP12	Matrix Metallopeptidase 12	Protein Coding	45	GC11M102862	4.42
379	ADCY10	Adenylate Cyclase 10	Protein Coding	44	GC01M167809	4.41
380	TGFB2	Transforming Growth Factor Beta 2	Protein Coding	50	GC01P218345	4.41
381	HSPD1	Heat Shock Protein Family D (Hsp60) Member 1	Protein Coding	46	GC02M197486	4.4
382	TNFSF13B	TNF Superfamily Member 13b	Protein Coding	44	GC13P108251	4.4
383	PTPN22	Protein Tyrosine Phosphatase Non-Receptor Type 22	Protein Coding	47	GC01M113813	4.39
384	NGFR	Nerve Growth Factor Receptor	Protein Coding	45	GC17P049495	4.38
385	KLK5	Kallikrein Related Peptidase 5	Protein Coding	41	GC19M050943	4.38
386	MBP	Myelin Basic Protein	Protein Coding	44	GC18M076978	4.38
387	TLR3	Toll Like Receptor 3	Protein Coding	50	GC04P186059	4.33
388	TRPV3	Transient Receptor Potential Cation Channel Subfamily V Member 3	Protein Coding	43	GC17M003986	4.33
389	CSF3	Colony Stimulating Factor 3	Protein Coding	40	GC17P040015	4.32
390	CD3G	CD3g Molecule	Protein Coding	46	GC11P118344	4.32
391	ELANE	Elastase, Neutrophil Expressed	Protein Coding	48	GC19P002165	4.3
392	MALT1	MALT1 Paracaspase	Protein Coding	46	GC18P058671	4.28
393	NLRP1	NLR Family Pyrin Domain Containing 1	Protein Coding	43	GC17M005499	4.27
394	LAMA3	Laminin Subunit Alpha 3	Protein Coding	45	GC18P023689	4.25
395	KRT2	Keratin 2	Protein Coding	41	GC12M052695	4.25
396	MIR126	MicroRNA 126	RNA Gene	23	GC09P136670	4.25
397	MIR152	MicroRNA 152	RNA Gene	22	GC17M048037	4.25
398	MIR148A	MicroRNA 148a	RNA Gene	20	GC07M025993	4.25
399	ASRT8	Asthma-Related Traits, Susceptibility To, 8	Genetic Locus	1	GC09U901396	4.25
400	MMP3	Matrix Metallopeptidase 3	Protein Coding	49	GC11M102835	4.22
401	C5AR1	Complement C5a Receptor 1	Protein Coding	44	GC19P047290	4.22
402	FZD6	Frizzled Class Receptor 6	Protein Coding	47	GC08P103298	4.22
403	IPO8	Importin 8	Protein Coding	38	GC12M030628	4.22
404	KRT74	Keratin 74	Protein Coding	37	GC12M052565	4.22
405	DOCK8-AS1	DOCK8 Antisense RNA 1	RNA Gene	21	GC09M000226	4.22
406	KONDS	Kondoh Syndrome	Genetic Locus	1	GC01U903333	4.22
407	BZX	Bazex Syndrome	Genetic Locus	1	GC0XU990258	4.22
408	KRT10	Keratin 10	Protein Coding	42	GC17M040818	4.21
409	NR3C1	Nuclear Receptor Subfamily 3 Group C Member 1	Protein Coding	49	GC05M143277	4.21
410	PIK3CA	Phosphatidylinositol-4,5-Bisphosphate 3-Kinase Catalytic Subunit Alpha	Protein Coding	52	GC03P179148	4.2
411	IL1RAPL2	Interleukin 1 Receptor Accessory Protein Like 2	Protein Coding	38	GC0XP104566	4.18
412	PTGS2	Prostaglandin-Endoperoxide Synthase 2	Protein Coding	48	GC01M186640	4.18
413	IFNA1	Interferon Alpha 1	Protein Coding	40	GC09P021577	4.16
414	FECH	Ferrochelatase	Protein Coding	46	GC18M057544	4.15
415	S100A9	S100 Calcium Binding Protein A9	Protein Coding	42	GC01P153357	4.14
416	KRT5	Keratin 5	Protein Coding	46	GC12M052514	4.1
417	SPP1	Secreted Phosphoprotein 1	Protein Coding	46	GC04P087975	4.09
418	IL17RB	Interleukin 17 Receptor B	Protein Coding	41	GC03P053855	4.07
419	SLC27A4	Solute Carrier Family 27 Member 4	Protein Coding	44	GC09P128340	4.07
420	CLDN1	Claudin 1	Protein Coding	46	GC03M190305	4.05
421	AP1S3	Adaptor Related Protein Complex 1 Subunit Sigma 3	Protein Coding	38	GC02M223751	4.04
422	TPT1	Tumor Protein, Translationally-Controlled 1	Protein Coding	45	GC13M045333	4.03
423	HLA-DMB	Major Histocompatibility Complex, Class II, DM Beta	Protein Coding	41	GC06M032934	4.01
424	CD80	CD80 Molecule	Protein Coding	41	GC03M119524	3.98
425	PEPD	Peptidase D	Protein Coding	44	GC19M033386	3.98
426	BCL2A1	BCL2 Related Protein A1	Protein Coding	40	GC15M080585	3.97
427	CYSLTR2	Cysteinyl Leukotriene Receptor 2	Protein Coding	47	GC13P048653	3.95
428	NPY	Neuropeptide Y	Protein Coding	45	GC07P024290	3.95
429	CTSG	Cathepsin G	Protein Coding	45	GC14M024573	3.93
430	IFNA2	Interferon Alpha 2	Protein Coding	42	GC09M021384	3.93
431	ITGB2	Integrin Subunit Beta 2	Protein Coding	49	GC21M044885	3.93

432	CX3CL1	C-X3-C Motif Chemokine Ligand 1	Protein Coding	42	GC16P057372	3.93
433	STAT5B	Signal Transducer And Activator Of Transcription 5B	Protein Coding	48	GC17M042199	3.92
434	IRF1	Interferon Regulatory Factor 1	Protein Coding	46	GC05M132440	3.91
435	IL12A	Interleukin 12A	Protein Coding	43	GC03P159988	3.9
436	KNG1	Kininogen 1	Protein Coding	46	GC03P186717	3.9
437	PCCB	Propionyl-CoA Carboxylase Subunit Beta	Protein Coding	46	GC03P136250	3.89
438	IRF3	Interferon Regulatory Factor 3	Protein Coding	45	GC19M049659	3.88
439	TNIP1	TNFAIP3 Interacting Protein 1	Protein Coding	40	GC05M151029	3.86
440	PTPRC	Protein Tyrosine Phosphatase Receptor Type C	Protein Coding	50	GC01P198607	3.85
441	CTRL	Chymotrypsin Like	Protein Coding	39	GC16M067927	3.84
442	IL13RA2	Interleukin 13 Receptor Subunit Alpha 2	Protein Coding	39	GC0XM115003	3.83
443	HMOX1	Heme Oxygenase 1	Protein Coding	50	GC22P035380	3.83
444	ITGA6	Integrin Subunit Alpha 6	Protein Coding	49	GC02P172427	3.83
445	TNFRSF4	TNF Receptor Superfamily Member 4	Protein Coding	41	GC01M001211	3.81
446	C3	Complement C3	Protein Coding	48	GC19M006677	3.78
447	CCR8	C-C Motif Chemokine Receptor 8	Protein Coding	42	GC03P039757	3.77
448	FAS	Fas Cell Surface Death Receptor	Protein Coding	49	GC10P090723	3.74
449	KRT17	Keratin 17	Protein Coding	44	GC17M041619	3.74
450	NFKB1	Nuclear Factor Kappa B Subunit 1	Protein Coding	50	GC04P102501	3.73
451	SPRR1B	Small Proline Rich Protein 1B	Protein Coding	37	GC01P153031	3.73
452	NOS2	Nitric Oxide Synthase 2	Protein Coding	48	GC17M027756	3.71
453	FASLG	Fas Ligand	Protein Coding	46	GC01P172628	3.7
454	FLI1	Fli-1 Proto-Oncogene, ETS Transcription Factor	Protein Coding	47	GC11P128686	3.7
455	CXCL12	C-X-C Motif Chemokine Ligand 12	Protein Coding	44	GC10M044294	3.66
456	MIR146A	MicroRNA 146a	RNA Gene	23	GC05P160485	3.66
457	FCGR2B	Fc Gamma Receptor IIb	Protein Coding	46	GC01P161663	3.65
458	LDHA	Lactate Dehydrogenase A	Protein Coding	50	GC11P018394	3.64
459	CCL28	C-C Motif Chemokine Ligand 28	Protein Coding	40	GC05M043356	3.63
460	CD19	CD19 Molecule	Protein Coding	48	GC16P039277	3.6
461	CCL19	C-C Motif Chemokine Ligand 19	Protein Coding	40	GC09M034692	3.6
462	IL17RE	Interleukin 17 Receptor E	Protein Coding	39	GC03P011967	3.59
463	CXCR4	C-X-C Motif Chemokine Receptor 4	Protein Coding	50	GC02M136114	3.59
464	SREBF1	Sterol Regulatory Element Binding Transcription Factor 1	Protein Coding	45	GC17M017810	3.56
465	CLEC16A	C-Type Lectin Domain Containing 16A	Protein Coding	37	GC16P010944	3.56
466	TFRC	Transferrin Receptor	Protein Coding	47	GC03M196027	3.55
467	TLR10	Toll Like Receptor 10	Protein Coding	40	GC04M038773	3.54
468	SPRR1A	Small Proline Rich Protein 1A	Protein Coding	36	GC01P152984	3.52
469	TNFSF18	TNF Superfamily Member 18	Protein Coding	38	GC01M173009	3.49
470	MEFV	MEFV Innate Immuity Regulator, Pyrin	Protein Coding	43	GC16M006424	3.49
471	CXCR2	C-X-C Motif Chemokine Receptor 2	Protein Coding	48	GC02P218125	3.48
472	JAK3	Janus Kinase 3	Protein Coding	50	GC19M017824	3.47
473	CXCR1	C-X-C Motif Chemokine Receptor 1	Protein Coding	42	GC02M218162	3.47
474	IL18RAP	Interleukin 18 Receptor Accessory Protein	Protein Coding	38	GC02P102418	3.46
475	KATNAL1	Katanin Catalytic Subunit A1 Like 1	Protein Coding	37	GC13M030202	3.45
476	ACTL9	Actin Like 9	Protein Coding	34	GC19M008697	3.44
477	FABP5	Fatty Acid Binding Protein 5	Protein Coding	40	GC08P081282	3.43
478	PDCD1	Programmed Cell Death 1	Protein Coding	46	GC02M241849	3.41
479	PPARA	Peroxisome Proliferator Activated Receptor Alpha	Protein Coding	45	GC22P046150	3.37
480	CD209	CD209 Molecule	Protein Coding	42	GC19M007739	3.36
481	IRF7	Interferon Regulatory Factor 7	Protein Coding	47	GC11M000612	3.36
482	RETN	Resistin	Protein Coding	42	GC19P007669	3.35
483	TNFRSF1A	TNF Receptor Superfamily Member 1A	Protein Coding	48	GC12M006328	3.35
484	FADS2	Fatty Acid Desaturase 2	Protein Coding	44	GC11P061792	3.34
485	B2M	Beta-2-Microglobulin	Protein Coding	48	GC15P044711	3.34
486	MICU1	Mitochondrial Calcium Uptake 1	Protein Coding	40	GC10M072367	3.33
487	DSG4	Desmoglein 4	Protein Coding	42	GC18P031377	3.33
488	PPARG	Peroxisome Proliferator Activated Receptor Gamma	Protein Coding	51	GC03P012287	3.31
489	LCN2	Lipocalin 2	Protein Coding	43	GC09P128149	3.3
490	LELP1	Late Cornified Envelope Like Proline Rich 1	Protein Coding	32	GC01P153175	3.28
491	ERBB2	Erb-B2 Receptor Tyrosine Kinase 2	Protein Coding	53	GC17P039687	3.28
492	BCL2	BCL2 Apoptosis Regulator	Protein Coding	49	GC18M063123	3.28
493	SERPINB7	Serpin Family B Member 7	Protein Coding	41	GC18P063752	3.28
494	CYP1A1	Cytochrome P450 Family 1 Subfamily A Member 1	Protein Coding	48	GC15M074719	3.26
495	CCR1	C-C Motif Chemokine Receptor 1	Protein Coding	45	GC03M046218	3.25
496	PRL	Prolactin	Protein Coding	42	GC06M022287	3.24
497	DNMT1	DNA Methyltransferase 1	Protein Coding	50	GC19M010133	3.24
498	PLA2G4D	Phospholipase A2 Group IVD	Protein Coding	39	GC15M042067	3.24
499	MUC16	Mucin 16, Cell Surface Associated	Protein Coding	38	GC19M008848	3.24
500	BGLAP	Bone Gamma-Carboxyglutamate Protein	Protein Coding	40	GC01P156242	3.24
501	TNFSF13	TNF Superfamily Member 13	Protein Coding	44	GC17P007558	3.23
502	IL17RD	Interleukin 17 Receptor D	Protein Coding	44	GC03M057089	3.23
503	SHARPIN	SHANK Associated RH Domain Interactor	Protein Coding	40	GC08M144098	3.21
504	SST	Somatostatin	Protein Coding	41	GC03M187668	3.2
505	STAT5A	Signal Transducer And Activator Of Transcription 5A	Protein Coding	46	GC17P042287	3.19
506	TIRAP	TIR Domain Containing Adaptor Protein	Protein Coding	42	GC11P126284	3.19
507	ORAI1	ORAI Calcium Release-Activated Calcium Modulator 1	Protein Coding	43	GC12P125513	3.19
508	APOC1	Apolipoprotein C1	Protein Coding	40	GC19P044914	3.19
509	ITK	IL2 Inducible T Cell Kinase	Protein Coding	51	GC05P157158	3.19
510	KRT19	Keratin 19	Protein Coding	44	GC17M041523	3.18
511	ITGA4	Integrin Subunit Alpha 4	Protein Coding	47	GC02P181456	3.17
512	SMAD3	SMAD Family Member 3	Protein Coding	49	GC15P067063	3.17
513	ERAP1	Endoplasmic Reticulum Aminopeptidase 1	Protein Coding	45	GC05M096760	3.16
514	KLK6	Kallikrein Related Peptidase 6	Protein Coding	43	GC19M050958	3.16
515	LTA4H	Leukotriene A4 Hydrolase	Protein Coding	45	GC12M096000	3.16
516	CYP24A1	Cytochrome P450 Family 24 Subfamily A Member 1	Protein Coding	46	GC20M054153	3.15
517	DDX39B	DExD-Box Helicase 39B	Protein Coding	38	GC06M031530	3.15
518	AOC1	Amine Oxidase Copper Containing 1	Protein Coding	39	GC07P150824	3.15

519	S100A2	S100 Calcium Binding Protein A2	Protein Coding	41	GC01M153561	3.12
520	GATA1	GATA Binding Protein 1	Protein Coding	46	GC0XP048786	3.11
521	ANXA5	Annexin A5	Protein Coding	45	GC04M121667	3.11
522	TRPV1	Transient Receptor Potential Cation Channel Subfamily V Member 1	Protein Coding	46	GC17M003565	3.11
523	UBAC2	UBA Domain Containing 2	Protein Coding	38	GC13P099200	3.1
524	PDE3B	Phosphodiesterase 3B	Protein Coding	44	GC11P014643	3.1
525	REL	REL Proto-Oncogene, NF-KB Subunit	Protein Coding	45	GC02P060881	3.09
526	TNFSF12	TNF Superfamily Member 12	Protein Coding	40	GC17P010605	3.09
527	IL11	Interleukin 11	Protein Coding	40	GC19M055364	3.09
528	TMEM165	Transmembrane Protein 165	Protein Coding	38	GC04P055395	3.05
529	RAD50	RAD50 Double Strand Break Repair Protein	Protein Coding	49	GC05P132556	3.04
530	PIK3CD	Phosphatidylinositol-4,5-Bisphosphate 3-Kinase Catalytic Subunit Delta	Protein Coding	52	GC01P009629	3.04
531	SLC29A3	Solute Carrier Family 29 Member 3	Protein Coding	44	GC10P071320	3.04
532	KNSTRN	Kinetochore Localized Astrin (SPAG5) Binding Protein	Protein Coding	37	GC15P040382	3.04
533	ITGB4	Integrin Subunit Beta 4	Protein Coding	48	GC17P075721	3.03
534	S100A10	S100 Calcium Binding Protein A10	Protein Coding	44	GC01M151955	3.03
535	LCE3A	Late Cornified Envelope 3A	Protein Coding	26	GC01M152595	3.03
536	TLR7	Toll Like Receptor 7	Protein Coding	46	GC0XP012867	3.01
537	FCER1G	Fc Epsilon Receptor Ig	Protein Coding	42	GC01P161215	3.01
538	CYP3A4	Cytochrome P450 Family 3 Subfamily A Member 4	Protein Coding	49	GC07M099759	3.01
539	KRT14	Keratin 14	Protein Coding	46	GC17M041582	3.01
540	GLB1	Galactosidase Beta 1	Protein Coding	48	GC03M032963	3
541	ENO1	Enolase 1	Protein Coding	46	GC01M008861	2.99
542	PDYN	Prodynorphin	Protein Coding	44	GC20M001978	2.99
543	TOLLIP	Toll Interacting Protein	Protein Coding	43	GC11M001274	2.99
544	IL19	Interleukin 19	Protein Coding	40	GC01P206770	2.97
545	HTR1A	5-Hydroxytryptamine Receptor 1A	Protein Coding	46	GC05M063960	2.97
546	PCCA	Propionyl-CoA Carboxylase Subunit Alpha	Protein Coding	46	GC13P100089	2.97
547	FADS1	Fatty Acid Desaturase 1	Protein Coding	44	GC11M061799	2.96
548	ARG1	Arginase 1	Protein Coding	48	GC06P131473	2.96
549	CASP8	Caspase 8	Protein Coding	50	GC02P201233	2.95
550	NOS3	Nitric Oxide Synthase 3	Protein Coding	50	GC07P150990	2.94
551	FGFBP2	Fibroblast Growth Factor Binding Protein 2	Protein Coding	34	GC04M015961	2.94
552	ASAH2	N-Acylsphingosine Amidohydrolase 2	Protein Coding	41	GC10M050182	2.93
553	TGM5	Transglutaminase 5	Protein Coding	41	GC15M043609	2.93
554	CD160	CD160 Molecule	Protein Coding	39	GC01P145719	2.92
555	ANOS1	Anosmin 1	Protein Coding	37	GC0XM008528	2.92
556	PTGS1	Prostaglandin-Endoperoxide Synthase 1	Protein Coding	46	GC09P122370	2.92
557	PKP2	Plakophilin 2	Protein Coding	44	GC12M032790	2.92
558	SMPD2	Sphingomyelin Phosphodiesterase 2	Protein Coding	40	GC06P109440	2.91
559	ARTN	Artemin	Protein Coding	40	GC01P043933	2.91
560	HLA-DPA1	Major Histocompatibility Complex, Class II, DP Alpha 1	Protein Coding	41	GC06M033064	2.91
561	AREG	Amphiregulin	Protein Coding	42	GC04P074445	2.91
562	RORC	RAR Related Orphan Receptor C	Protein Coding	44	GC01M151806	2.9
563	CRNN	Cornulin	Protein Coding	36	GC01M152381	2.9
564	NLRP12	NLR Family Pyrin Domain Containing 12	Protein Coding	43	GC19M053793	2.9
565	CD2	CD2 Molecule	Protein Coding	42	GC01P116754	2.89
566	TNFRSF25	TNF Receptor Superfamily Member 25	Protein Coding	41	GC01M006460	2.88
567	JUP	Junction Plakoglobin	Protein Coding	47	GC17M041754	2.87
568	SERPINA3	Serpin Family A Member 3	Protein Coding	44	GC14P094612	2.87
569	CLEC7A	C-Type Lectin Domain Containing 7A	Protein Coding	44	GC12M019786	2.86
570	SLC6A4	Solute Carrier Family 6 Member 4	Protein Coding	48	GC17M030194	2.83
571	CD163	CD163 Molecule	Protein Coding	43	GC12M007646	2.83
572	LTF	Lactotransferrin	Protein Coding	44	GC03M046435	2.83
573	ADIPOQ	Adiponectin, C1Q And Collagen Domain Containing	Protein Coding	45	GC03P186842	2.83
574	CRHR1	Corticotropin Releasing Hormone Receptor 1	Protein Coding	44	GC17P045784	2.83
575	ANXA2	Annexin A2	Protein Coding	47	GC15M060347	2.82
576	DLX3	Distal-Less Homeobox 3	Protein Coding	41	GC17M049990	2.82
577	ADM2	Adrenomedullin 2	Protein Coding	35	GC22P050481	2.82
578	IL9R	Interleukin 9 Receptor	Protein Coding	36	GC0XP155997	2.81
579	RNASE7	Ribonuclease A Family Member 7	Protein Coding	34	GC14P021042	2.81
580	SERPINB3	Serpin Family B Member 3	Protein Coding	41	GC18M063655	2.81
581	SLC39A10	Solute Carrier Family 39 Member 10	Protein Coding	39	GC02P195575	2.8
582	AIRE	Autoimmune Regulator	Protein Coding	44	GC21P044285	2.8
583	MMP8	Matrix Metallopeptidase 8	Protein Coding	48	GC11M102617	2.79
584	IL32	Interleukin 32	Protein Coding	38	GC16P009928	2.79
585	SMPD1	Sphingomyelin Phosphodiesterase 1	Protein Coding	48	GC11P006390	2.78
586	CRP	C-Reactive Protein	Protein Coding	45	GC01M159725	2.77
587	ACKR1	Atypical Chemokine Receptor 1 (Duffy Blood Group)	Protein Coding	37	GC01P159203	2.76
588	LTC4S	Leukotriene C4 Synthase	Protein Coding	42	GC05P179793	2.76
589	ST2	Suppression Of Tumorigenicity 2	Genetic Locus	8	GC11U990127	2.75
590	MOGS	Mannosyl-Oligosaccharide Glucosidase	Protein Coding	42	GC02M074461	2.74
591	SLC35A3	Solute Carrier Family 35 Member A3	Protein Coding	41	GC01P099968	2.74
592	ALG6	ALG6 Alpha-1,3-Glucosyltransferase	Protein Coding	41	GC01P063367	2.74
593	SLC35D1	Solute Carrier Family 35 Member D1	Protein Coding	40	GC01M066999	2.74
594	ALG11	ALG11 Alpha-1,2-Mannosyltransferase	Protein Coding	40	GC13P052012	2.74
595	ALG14	ALG14 UDP-N-Acetylglucosaminyltransferase Subunit	Protein Coding	40	GC01M094974	2.74
596	SLC35C1	Solute Carrier Family 35 Member C1	Protein Coding	40	GC11P046395	2.74
597	ALG12	ALG12 Alpha-1,6-Mannosyltransferase	Protein Coding	37	GC22M055211	2.74
598	CASP14	Caspase 14	Protein Coding	45	GC19P015049	2.74
599	S100A14	S100 Calcium Binding Protein A14	Protein Coding	37	GC01M153614	2.71
600	PBX2	PBX Homeobox 2	Protein Coding	37	GC06M032184	2.69
601	GPSM3	G Protein Signaling Modulator 3	Protein Coding	33	GC06M061332	2.69
602	TSBP1	Testis Expressed Basic Protein 1	Protein Coding	25	GC06M032288	2.69
603	MIR4435-2HG	MIR4435-2 Host Gene	RNA Gene	18	GC02M111019	2.69
604	XK	X-Linked Kx Blood Group	Protein Coding	40	GC0XP037685	2.67
605	GSN	Gelsolin	Protein Coding	48	GC09P121201	2.66

606	SART1	Spliceosome Associated Factor 1, Recruiter Of U4/U6.U5 Tri-SnRNP	Protein Coding	40	GC11P069032	2.66
607	ALDH1A1	Aldehyde Dehydrogenase 1 Family Member A1	Protein Coding	46	GC09M072900	2.66
608	CST6	Cystatin E/M	Protein Coding	40	GC11P069036	2.66
609	ENO2	Enolase 2	Protein Coding	46	GC12P006913	2.66
610	PSENEN	Presenilin Enhancer, Gamma-Secretase Subunit	Protein Coding	44	GC19P051423	2.65
611	NTF4	Neurotrophin 4	Protein Coding	43	GC19M062182	2.64
612	IQGAP1	IQ Motif Containing GTPase Activating Protein 1	Protein Coding	42	GC15P090388	2.63
613	RPTN	Repetin	Protein Coding	36	GC01M152153	2.63
614	SOD2	Superoxide Dismutase 2	Protein Coding	49	GC06M159669	2.62
615	LGALS3	Galectin 3	Protein Coding	44	GC14P055124	2.61
616	C3AR1	Complement C3a Receptor 1	Protein Coding	42	GC12M008058	2.61
617	CSF2RA	Colony Stimulating Factor 2 Receptor Subunit Alpha	Protein Coding	44	GC0XP001571	2.6
618	GRP	Gastrin Releasing Peptide	Protein Coding	40	GC18P059220	2.59
619	S100A4	S100 Calcium Binding Protein A4	Protein Coding	44	GC01M153543	2.58
620	RIPK2	Receptor Interacting Serine/Threonine Kinase 2	Protein Coding	45	GC08P089786	2.57
621	FAF1	Fas Associated Factor 1	Protein Coding	42	GC01M050439	2.57
622	FUT7	Fucosyltransferase 7	Protein Coding	40	GC09M137030	2.57
623	FLT4	Fms Related Receptor Tyrosine Kinase 4	Protein Coding	50	GC05M180607	2.57
624	GRB7	Growth Factor Receptor Bound Protein 7	Protein Coding	43	GC17P050338	2.56
625	VNN3P	Vanin 3, Pseudogene	Pseudogene	26	GC06M132724	2.56
626	RELA	RELA Proto-Oncogene, NF-KB Subunit	Protein Coding	50	GC11M065653	2.56
627	ADH1B	Alcohol Dehydrogenase 1B (Class I), Beta Polypeptide	Protein Coding	44	GC04M099304	2.56
628	TNXB	Tenascin XB	Protein Coding	44	GC06M061322	2.55
629	SEC23A	SEC23 Homolog A, COPII Coat Complex Component	Protein Coding	44	GC14M039031	2.54
630	S100A12	S100 Calcium Binding Protein A12	Protein Coding	39	GC01M153373	2.53
631	MTOR	Mechanistic Target Of Rapamycin Kinase	Protein Coding	52	GC01M011106	2.53
632	TNFSF14	TNF Superfamily Member 14	Protein Coding	41	GC19M006663	2.53
633	AOC3	Amine Oxidase Copper Containing 3	Protein Coding	44	GC17P042851	2.52
634	CP	Ceruloplasmin	Protein Coding	49	GC03M149162	2.52
635	DEFA5	Defensin Alpha 5	Protein Coding	37	GC08M007057	2.51
636	IL17C	Interleukin 17C	Protein Coding	37	GC16P088638	2.5
637	S100A6	S100 Calcium Binding Protein A6	Protein Coding	42	GC01M153545	2.5
638	S100A1	S100 Calcium Binding Protein A1	Protein Coding	40	GC01P153627	2.5
639	S100A3	S100 Calcium Binding Protein A3	Protein Coding	38	GC01M153547	2.5
640	PGLYRP3	Peptidoglycan Recognition Protein 3	Protein Coding	37	GC01M153297	2.5
641	S100A5	S100 Calcium Binding Protein A5	Protein Coding	33	GC01M153537	2.5
642	EDNRB	Endothelin Receptor Type B	Protein Coding	48	GC13M077895	2.5
643	ETS1	ETS Proto-Oncogene 1, Transcription Factor	Protein Coding	46	GC11M128458	2.5
644	JAZF1	JAZF Zinc Finger 1	Protein Coding	38	GC07M027830	2.5
645	BCR	BCR Activator Of RhoGEF And GTPase	Protein Coding	50	GC22P023179	2.49
646	UBASH3A	Ubiquitin Associated And SH3 Domain Containing A	Protein Coding	38	GC21P042403	2.49
647	LAMC2	Laminin Subunit Gamma 2	Protein Coding	47	GC01P183186	2.48
648	LAMB3	Laminin Subunit Beta 3	Protein Coding	46	GC01M209614	2.48
649	EGR2	Early Growth Response 2	Protein Coding	43	GC10M062811	2.48
650	ADO	2-Aminoethanethiol Dioxygenase	Protein Coding	38	GC10P062804	2.48
651	PFDN4	Prefoldin Subunit 4	Protein Coding	38	GC20P054207	2.48
652	ZNF365	Zinc Finger Protein 365	Protein Coding	38	GC10P062374	2.48
653	CCDC80	Coiled-Coil Domain Containing 80	Protein Coding	37	GC03M112604	2.48
654	OR10A3	Olfactory Receptor Family 10 Subfamily A Member 3	Protein Coding	29	GC11M007951	2.48
655	MIR1208	MicroRNA 1208	RNA Gene	14	GC08P128150	2.48
656	LINC00824	Long Intergenic Non-Protein Coding RNA 824	RNA Gene	13	GC08M128405	2.48
657	BLMH	Bleomycin Hydrolase	Protein Coding	44	GC17M030248	2.47
658	MIR223	MicroRNA 223	RNA Gene	22	GC0XP066018	2.47
659	ATF6B	Activating Transcription Factor 6 Beta	Protein Coding	38	GC06M032115	2.47
660	SLC25A46	Solute Carrier Family 25 Member 46	Protein Coding	38	GC05P110738	2.47
661	CDH1	Cadherin 1	Protein Coding	49	GC16P068737	2.47
662	CHIA	Chitinase Acidic	Protein Coding	42	GC01P111291	2.46
663	NAMPT	Nicotinamide Phosphoribosyltransferase	Protein Coding	46	GC07M106248	2.46
664	MYD88	MYD88 Innate Immune Signal Transduction Adaptor	Protein Coding	49	GC03P038139	2.44
665	TIMD4	T Cell Immunoglobulin And Mucin Domain Containing 4	Protein Coding	37	GC05M156919	2.44
666	DEFA4	Defensin Alpha 4	Protein Coding	36	GC08M006935	2.44
667	DEFA6	Defensin Alpha 6	Protein Coding	36	GC08M006924	2.44
668	HSPB2	Heat Shock Protein Family B (Small) Member 2	Protein Coding	39	GC11P111913	2.42
669	SPRR3	Small Proline Rich Protein 3	Protein Coding	38	GC01P153001	2.42
670	ADAMTS10	ADAM Metallopeptidase With Thrombospondin Type 1 Motif 10	Protein Coding	43	GC19M008580	2.42
671	ZBTB10	Zinc Finger And BTB Domain Containing 10	Protein Coding	34	GC08P080485	2.42
672	ATP6V1G2-DDX39B	ATP6V1G2-DDX39B Readthrough (NMD Candidate)	RNA Gene	18	GC06M061303	2.42
673	KYNU	Kynureninase	Protein Coding	46	GC02P142877	2.4
674	EPO	Erythropoietin	Protein Coding	41	GC07P100720	2.4
675	MRGPRX2	MAS Related GPR Family Member X2	Protein Coding	37	GC11M019077	2.4
676	JAK2	Janus Kinase 2	Protein Coding	51	GC09P004985	2.38
677	APOA1	Apolipoprotein A1	Protein Coding	48	GC11M116835	2.36
678	CLDN4	Claudin 4	Protein Coding	41	GC07P073799	2.36
679	CYP27A1	Cytochrome P450 Family 27 Subfamily A Member 1	Protein Coding	47	GC02P218781	2.36
680	TYK2	Tyrosine Kinase 2	Protein Coding	51	GC19M010350	2.36
681	JUN	Jun Proto-Oncogene, AP-1 Transcription Factor Subunit	Protein Coding	47	GC01M058780	2.36
682	COL6A6	Collagen Type VI Alpha 6 Chain	Protein Coding	34	GC03P133405	2.35
683	TMEM232	Transmembrane Protein 232	Protein Coding	29	GC05M110289	2.34
684	ADA	Adenosine Deaminase	Protein Coding	49	GC20M044620	2.34
685	KRT9	Keratin 9	Protein Coding	41	GC17M041565	2.34
686	NELL2	Neural EGFL Like 2	Protein Coding	39	GC12M044509	2.33
687	TRPC6	Transient Receptor Potential Cation Channel Subfamily C Member 6	Protein Coding	49	GC11M101451	2.33
688	GNAI1	G Protein Subunit Alpha I1	Protein Coding	45	GC07P079769	2.32
689	TXN	Thioredoxin	Protein Coding	45	GC09M110243	2.31
690	RORA	RAR Related Orphan Receptor A	Protein Coding	48	GC15M060488	2.3
691	IRF5	Interferon Regulatory Factor 5	Protein Coding	46	GC07P128937	2.28
692	SELPLG	Selectin P Ligand	Protein Coding	42	GC12M108621	2.28

693	CD63	CD63 Molecule	Protein Coding	41	GC12M055725	2.27
694	MIR203A	MicroRNA 203a	RNA Gene	21	GC14P108823	2.26
695	NAT1	N-Acetyltransferase 1	Protein Coding	44	GC08P018179	2.26
696	ERAP2	Endoplasmic Reticulum Aminopeptidase 2	Protein Coding	40	GC05P096875	2.26
697	NTS	Neurotensin	Protein Coding	40	GC12P085876	2.25
698	IKBKB	Inhibitor Of Nuclear Factor Kappa B Kinase Subunit Beta	Protein Coding	52	GC08P042271	2.25
699	BHLHE40	Basic Helix-Loop-Helix Family Member E40	Protein Coding	40	GC03P004980	2.25
700	GJA1	Gap Junction Protein Alpha 1	Protein Coding	49	GC06P121436	2.25
701	IL2RG	Interleukin 2 Receptor Subunit Gamma	Protein Coding	46	GC0XM071108	2.24
702	PRKCA	Protein Kinase C Alpha	Protein Coding	49	GC17P066302	2.24
703	CD36	CD36 Molecule	Protein Coding	47	GC07P080369	2.24
704	HTR2A	5-Hydroxytryptamine Receptor 2A	Protein Coding	47	GC13M046831	2.24
705	MME	Membrane Metalloendopeptidase	Protein Coding	49	GC03P155024	2.23
706	IL24	Interleukin 24	Protein Coding	40	GC01P206897	2.22
707	SLC26A2	Solute Carrier Family 26 Member 2	Protein Coding	44	GC05P149944	2.22
708	TRAF6	TNF Receptor Associated Factor 6	Protein Coding	45	GC11M036467	2.22
709	CAT	Catalase	Protein Coding	49	GC11P034460	2.21
710	ABCG2	ATP Binding Cassette Subfamily G Member 2 (Junior Blood Group)	Protein Coding	49	GC04M088090	2.21
711	CRISP3	Cysteine Rich Secretory Protein 3	Protein Coding	38	GC06M049727	2.21
712	IL17RA	Interleukin 17 Receptor A	Protein Coding	44	GC22P018163	2.2
713	PECAM1	Platelet And Endothelial Cell Adhesion Molecule 1	Protein Coding	40	GC17M064319	2.19
714	ERBB4	Erb-B2 Receptor Tyrosine Kinase 4	Protein Coding	53	GC02M211375	2.19
715	MMP13	Matrix Metallopeptidase 13	Protein Coding	50	GC11M102942	2.18
716	OXA1L	OXA1L Mitochondrial Inner Membrane Protein	Protein Coding	38	GC14P022766	2.18
717	FKBP1A	FKBP Prolyl Isomerase 1A	Protein Coding	45	GC20M001369	2.18
718	ENPP3	Ectonucleotide Pyrophosphatase/Phosphodiesterase 3	Protein Coding	43	GC06P131617	2.18
719	F2R	Coagulation Factor II Thrombin Receptor	Protein Coding	46	GC05P076716	2.16
720	UROS	Uroporphyrinogen III Synthase	Protein Coding	41	GC10M125784	2.16
721	MC1R	Melanocortin 1 Receptor	Protein Coding	46	GC16P089912	2.16
722	MC3R	Melanocortin 3 Receptor	Protein Coding	42	GC20P056248	2.16
723	MUC1	Mucin 1, Cell Surface Associated	Protein Coding	48	GC01M155185	2.15
724	DPEP1	Dipeptidase 1	Protein Coding	42	GC16P089613	2.15
725	PPARD	Peroxisome Proliferator Activated Receptor Delta	Protein Coding	46	GC06P076988	2.15
726	DNAJB6	DnaJ Heat Shock Protein Family (Hsp40) Member B6	Protein Coding	41	GC07P157335	2.14
727	CLDN3	Claudin 3	Protein Coding	40	GC07M073768	2.13
728	SPINK9	Serine Peptidase Inhibitor Kazal Type 9	Protein Coding	28	GC05P148321	2.12
729	HSD11B1	Hydroxysteroid 11-Beta Dehydrogenase 1	Protein Coding	49	GC01P209686	2.12
730	NCR3	Natural Cytotoxicity Triggering Receptor 3	Protein Coding	38	GC06M031588	2.12
731	FLII	FLII Actin Remodeling Protein	Protein Coding	42	GC17M018244	2.11
732	PIP	Prolactin Induced Protein	Protein Coding	39	GC07P143132	2.1
733	TP53	Tumor Protein P53	Protein Coding	51	GC17M007661	2.1
734	ABCB1	ATP Binding Cassette Subfamily B Member 1	Protein Coding	50	GC07M087504	2.1
735	MTHFR	Methylenetetrahydrofolate Reductase	Protein Coding	48	GC01M011785	2.1
736	NR4A2	Nuclear Receptor Subfamily 4 Group A Member 2	Protein Coding	46	GC02M156324	2.09
737	F2RL2	Coagulation Factor II Thrombin Receptor Like 2	Protein Coding	40	GC05M076615	2.09
738	MMP10	Matrix Metallopeptidase 10	Protein Coding	46	GC11M102770	2.08
739	GAL	Galanin And GMAP Prepropeptide	Protein Coding	44	GC11P069367	2.07
740	GSDMB	Gasdermin B	Protein Coding	36	GC17M039904	2.07
741	ATP12A	ATPase H+/K+ Transporting Non-Gastric Alpha2 Subunit	Protein Coding	42	GC13P024680	2.05
742	TRPA1	Transient Receptor Potential Cation Channel Subfamily A Member 1	Protein Coding	46	GC08M072019	2.04
743	SGPL1	Sphingosine-1-Phosphate Lyase 1	Protein Coding	45	GC10P070815	2.03
744	NPM1	Nucleophosmin 1	Protein Coding	49	GC05P171387	2.03
745	SLC2A2	Solute Carrier Family 2 Member 2	Protein Coding	48	GC03M170996	2.03
746	AVP	Arginine Vasopressin	Protein Coding	45	GC20M003082	2.03
747	DSG2	Desmoglein 2	Protein Coding	45	GC18P031498	2.03
748	DMRTA1	DMRT Like Family A1	Protein Coding	36	GC09P022436	2.02
749	XIRP2	Xin Actin Binding Repeat Containing 2	Protein Coding	35	GC02P166888	2.02
750	NR4A1	Nuclear Receptor Subfamily 4 Group A Member 1	Protein Coding	46	GC12P052022	2.02
751	SSTR5	Somatostatin Receptor 5	Protein Coding	41	GC16P001072	2
752	COIL	Coilin	Protein Coding	40	GC17M056938	2
753	GNA11	G Protein Subunit Alpha 11	Protein Coding	47	GC19P003094	2
754	PRF1	Perforin 1	Protein Coding	45	GC10M070597	2
755	PPL	Periplakin	Protein Coding	40	GC16M006504	2
756	VPS50	VPS50 Subunit Of EARP/GARPII Complex	Protein Coding	29	GC07P093233	1.99
757	HBEGF	Heparin Binding EGF Like Growth Factor	Protein Coding	43	GC05M140332	1.99
758	OPRK1	Opioid Receptor Kappa 1	Protein Coding	45	GC08M053227	1.98
759	IFNK	Interferon Kappa	Protein Coding	38	GC09P027514	1.98
760	CABIN1	Calcineurin Binding Protein 1	Protein Coding	42	GC22P024011	1.98
761	CHIT1	Chitinase 1	Protein Coding	43	GC01M203181	1.96
762	PHB1	Prohibitin 1	Protein Coding	35	GC17M049406	1.96
763	DSC3	Desmocollin 3	Protein Coding	43	GC18M030990	1.95
764	THY1	Thy-1 Cell Surface Antigen	Protein Coding	42	GC11M119417	1.95
765	MIR151A	MicroRNA 151a	RNA Gene	20	GC08M140733	1.95
766	CTSL	Cathepsin L	Protein Coding	46	GC09P087725	1.95
767	LGALS9	Galectin 9	Protein Coding	39	GC17P027629	1.95
768	TH	Tyrosine Hydroxylase	Protein Coding	50	GC11M002163	1.95
769	S100A11	S100 Calcium Binding Protein A11	Protein Coding	41	GC01M152032	1.94
770	PNMT	Phenylethanolamine N-Methyltransferase	Protein Coding	43	GC17P039667	1.94
771	CLEC4E	C-Type Lectin Domain Family 4 Member E	Protein Coding	36	GC12M008535	1.94
772	AR	Androgen Receptor	Protein Coding	51	GC0XP067544	1.92
773	KLKB1	Kallikrein B1	Protein Coding	46	GC04P186208	1.92
774	YAP1	Yes1 Associated Transcriptional Regulator	Protein Coding	46	GC11P102110	1.92
775	CD247	CD247 Molecule	Protein Coding	48	GC01M167399	1.92
776	MAPK14	Mitogen-Activated Protein Kinase 14	Protein Coding	50	GC06P077003	1.91
777	AKR1C3	Aldo-Keto Reductase Family 1 Member C3	Protein Coding	46	GC10P005035	1.9
778	IL23R	Interleukin 23 Receptor	Protein Coding	44	GC01P067138	1.89
779	OPRM1	Opioid Receptor Mu 1	Protein Coding	48	GC06P154075	1.89

780	MDH2	Malate Dehydrogenase 2	Protein Coding	47	GC07P076048	1.89
781	ADM	Adrenomedullin	Protein Coding	44	GC11P010304	1.89
782	SLC7A9	Solute Carrier Family 7 Member 9	Protein Coding	44	GC19M032830	1.89
783	SPI1	Spi-1 Proto-Oncogene	Protein Coding	42	GC11M084329	1.89
784	PLS1	Plastin 1	Protein Coding	41	GC03P142596	1.89
785	DDX39A	DExD-Box Helicase 39A	Protein Coding	37	GC19M014408	1.89
786	FUT4	Fucosyltransferase 4	Protein Coding	36	GC11P094544	1.89
787	KPRP	Keratinocyte Proline Rich Protein	Protein Coding	30	GC01P152766	1.89
788	RPS5P5	RPS5 Pseudogene 5	Pseudogene	7	GC08P080300	1.89
789	CA3	Carbonic Anhydrase 3	Protein Coding	41	GC08P085373	1.88
790	PLA2G4A	Phospholipase A2 Group IVA	Protein Coding	48	GC01P186798	1.88
791	LTB4R	Leukotriene B4 Receptor	Protein Coding	44	GC14P024311	1.88
792	VIM	Vimentin	Protein Coding	49	GC10P017227	1.88
793	DES	Desmin	Protein Coding	46	GC02P219418	1.88
794	BCL10	BCL10 Immune Signaling Adaptor	Protein Coding	43	GC01M085265	1.88
795	SERPINA1	Serpin Family A Member 1	Protein Coding	48	GC14M094376	1.87
796	CD5L	CD5 Molecule Like	Protein Coding	38	GC01M157800	1.87
797	NFE2L2	NFE2 Like BZIP Transcription Factor 2	Protein Coding	48	GC02M177227	1.86
798	TSPO	Translocator Protein	Protein Coding	42	GC22P043151	1.85
799	KIAA1109	KIAA1109	Protein Coding	35	GC04P122152	1.85
800	IGFBP3	Insulin Like Growth Factor Binding Protein 3	Protein Coding	45	GC07M045912	1.85
801	MIR483	MicroRNA 483	RNA Gene	20	GC11M002736	1.85
802	KLRK1	Killer Cell Lectin Like Receptor K1	Protein Coding	40	GC12M019788	1.85
803	MIR21	MicroRNA 21	RNA Gene	25	GC17P059841	1.84
804	VEGFC	Vascular Endothelial Growth Factor C	Protein Coding	46	GC04M176683	1.84
805	ASPRV1	Aspartic Peptidase Retroviral Like 1	Protein Coding	35	GC02M069932	1.84
806	SHOC2	SHOC2 Leucine Rich Repeat Scaffold Protein	Protein Coding	40	GC10P110919	1.83
807	CNFN	Cornifelin	Protein Coding	32	GC19M042387	1.82
808	PLAUR	Plasminogen Activator, Urokinase Receptor	Protein Coding	44	GC19M043646	1.82
809	B3GAT1	Beta-1,3-Glucuronyltransferase 1	Protein Coding	44	GC11M134378	1.82
810	RAB7A	RAB7A, Member RAS Oncogene Family	Protein Coding	46	GC03P133360	1.82
811	CHGA	Chromogranin A	Protein Coding	44	GC14P092929	1.82
812	LGMN	Legumain	Protein Coding	41	GC14M092703	1.82
813	CTSV	Cathepsin V	Protein Coding	41	GC09M097029	1.82
814	MCC	MCC Regulator Of WNT Signaling Pathway	Protein Coding	40	GC05M113022	1.82
815	LGALS7	Galectin 7	Protein Coding	37	GC19M038770	1.82
816	CKLF	Chemokine Like Factor	Protein Coding	37	GC16P066552	1.82
817	LECT2	Leukocyte Cell Derived Chemotaxin 2	Protein Coding	36	GC05M135922	1.82
818	JCHAIN	Joining Chain Of Multimeric IgA And IgM	Protein Coding	36	GC04M070655	1.82
819	LOC110806262	Solute Carrier Family 6 Member 4 Gene Promoter	Biological Region	2	GC17P030235	1.82
820	STIN2-VNTR	Serotonin Transporter Intronic VNTR Enhancer	Biological Region	2	GC17P030221	1.82
821	ELP1	Elongator Acetyltransferase Complex Subunit 1	Protein Coding	34	GC09M108868	1.82
822	TNFRSF14	TNF Receptor Superfamily Member 14	Protein Coding	41	GC01P002555	1.81
823	RELB	RELB Proto-Oncogene, NF-KB Subunit	Protein Coding	44	GC19P052007	1.81
824	AZU1	Azurocidin 1	Protein Coding	40	GC19P000825	1.81
825	ITSN2	Intersectin 2	Protein Coding	39	GC02M024203	1.81
826	TNC	Tenascin C	Protein Coding	49	GC09M115019	1.81
827	CA2	Carbonic Anhydrase 2	Protein Coding	49	GC08P085463	1.81
828	ACKR2	Atypical Chemokine Receptor 2	Protein Coding	38	GC03P042804	1.8
829	KLK4	Kallikrein Related Peptidase 4	Protein Coding	43	GC19M062299	1.8
830	KLK14	Kallikrein Related Peptidase 14	Protein Coding	38	GC19M051077	1.8
831	MAPK3	Mitogen-Activated Protein Kinase 3	Protein Coding	48	GC16M035783	1.78
832	LTB4R2	Leukotriene B4 Receptor 2	Protein Coding	42	GC14P030885	1.78
833	LINC02676	Long Intergenic Non-Protein Coding RNA 2676	RNA Gene	9	GC10P008726	1.78
834	ENSG00000223808	Novel Transcript	RNA Gene	7	GC10P008943	1.78
835	PTK2	Protein Tyrosine Kinase 2	Protein Coding	46	GC08M140657	1.77
836	MC5R	Melanocortin 5 Receptor	Protein Coding	42	GC18P013824	1.77
837	CLDN7	Claudin 7	Protein Coding	40	GC17M007259	1.77
838	HAS1	Hyaluronan Synthase 1	Protein Coding	39	GC19M062343	1.77
839	APOE	Apolipoprotein E	Protein Coding	50	GC19P051999	1.77
840	SPINT2	Serine Peptidase Inhibitor, Kunitz Type 2	Protein Coding	43	GC19P038244	1.77
841	HAS2	Hyaluronan Synthase 2	Protein Coding	41	GC08M121594	1.77
842	VAMP3	Vesicle Associated Membrane Protein 3	Protein Coding	40	GC01P007765	1.77
843	CD180	CD180 Molecule	Protein Coding	37	GC05M067181	1.77
844	KRT75	Keratin 75	Protein Coding	36	GC12M052425	1.77
845	PKP1	Plakophilin 1	Protein Coding	41	GC01P201283	1.76
846	GAST	Gastrin	Protein Coding	39	GC17P041712	1.75
847	DSC2	Desmocollin 2	Protein Coding	45	GC18M031058	1.75
848	NCAM1	Neural Cell Adhesion Molecule 1	Protein Coding	46	GC11P112961	1.74
849	IL10RB	Interleukin 10 Receptor Subunit Beta	Protein Coding	44	GC21P033266	1.73
850	UCHL1	Ubiquitin C-Terminal Hydrolase L1	Protein Coding	49	GC04P041256	1.72
851	STIM1	Stromal Interaction Molecule 1	Protein Coding	48	GC11P003855	1.72
852	NTRK1	Neurotrophic Receptor Tyrosine Kinase 1	Protein Coding	48	GC01P156815	1.72
853	TP73	Tumor Protein P73	Protein Coding	45	GC01P003652	1.72
854	UGT1A9	UDP Glucuronosyltransferase Family 1 Member A9	Protein Coding	45	GC02P233671	1.72
855	AZGP1	Alpha-2-Glycoprotein 1, Zinc-Binding	Protein Coding	44	GC07M099967	1.72
856	ENPP2	Ectonucleotide Pyrophosphatase/Phosphodiesterase 2	Protein Coding	44	GC08M119556	1.72
857	SATB1	SATB Homeobox 1	Protein Coding	43	GC03M019799	1.72
858	SDC4	Syndecan 4	Protein Coding	43	GC20M045325	1.72
859	TIMP2	TIMP Metallopeptidase Inhibitor 2	Protein Coding	43	GC17M078852	1.72
860	FKBP8	FKBP Prolyl Isomerase 8	Protein Coding	42	GC19M018503	1.72
861	SRR	Serine Racemase	Protein Coding	42	GC17P002303	1.72
862	ESD	Esterase D	Protein Coding	42	GC13M046771	1.72
863	VDAC2	Voltage Dependent Anion Channel 2	Protein Coding	42	GC10P075210	1.72
864	HAS3	Hyaluronan Synthase 3	Protein Coding	41	GC16P069105	1.72
865	ORM1	Orosomucoid 1	Protein Coding	41	GC09P114323	1.72
866	TIMP4	TIMP Metallopeptidase Inhibitor 4	Protein Coding	40	GC03M012153	1.72

867	ESM1	Endothelial Cell Specific Molecule 1	Protein Coding	40	GC05M054977	1.72
868	CRISP2	Cysteine Rich Secretory Protein 2	Protein Coding	40	GC06M061626	1.72
869	PITPNB	Phosphatidylinositol Transfer Protein Beta	Protein Coding	40	GC22M027851	1.72
870	TTF2	Transcription Termination Factor 2	Protein Coding	40	GC01P117060	1.72
871	HS3ST2	Heparan Sulfate-Glucosamine 3-Sulfotransferase 2	Protein Coding	39	GC16P022814	1.72
872	CLIC4	Chloride Intracellular Channel 4	Protein Coding	39	GC01P024745	1.72
873	ITLN1	Intelectin 1	Protein Coding	38	GC01M160876	1.72
874	TIGIT	T Cell Immunoreceptor With Ig And ITIM Domains	Protein Coding	38	GC03P114276	1.72
875	NAT9	N-Acetyltransferase 9 (Putative)	Protein Coding	37	GC17M074770	1.72
876	APLN	Apelin	Protein Coding	37	GC0XM129645	1.72
877	RABGAP1	RAB GTPase Activating Protein 1	Protein Coding	37	GC09P122932	1.72
878	ADGRE1	Adhesion G Protein-Coupled Receptor E1	Protein Coding	36	GC19P006887	1.72
879	CALML5	Calmodulin Like 5	Protein Coding	35	GC10M005498	1.72
880	NAP1L2	Nucleosome Assembly Protein 1 Like 2	Protein Coding	34	GC0XM073212	1.72
881	ACKR4	Atypical Chemokine Receptor 4	Protein Coding	34	GC03P132597	1.72
882	ADGRE3	Adhesion G Protein-Coupled Receptor E3	Protein Coding	34	GC19M014619	1.72
883	LILRA6	Leukocyte Immunoglobulin Like Receptor A6	Protein Coding	33	GC19M062452	1.72
884	SBSN	Suprabasin	Protein Coding	33	GC19M063687	1.72
885	VSTM1	V-Set And Transmembrane Domain Containing 1	Protein Coding	33	GC19M054040	1.72
886	OR10G7	Olfactory Receptor Family 10 Subfamily G Member 7	Protein Coding	28	GC11M124038	1.72
887	KIR2DS1	Killer Cell Immunoglobulin Like Receptor, Two Ig Domains And Short Cytoplasmic Tail 1	Protein Coding	23	GC19Mr00063	1.72
888	MIR194-1	MicroRNA 194-1	RNA Gene	17	GC01M220118	1.72
889	CD27	CD27 Molecule	Protein Coding	45	GC12P018529	1.72
890	TJP1	Tight Junction Protein 1	Protein Coding	43	GC15M029699	1.7
891	SLC17A5	Solute Carrier Family 17 Member 5	Protein Coding	43	GC06M073593	1.7
892	TLR8	Toll Like Receptor 8	Protein Coding	47	GC0XP012924	1.7
893	HSPA1A	Heat Shock Protein Family A (Hsp70) Member 1A	Protein Coding	44	GC06P076883	1.69
894	RAG1	Recombination Activating 1	Protein Coding	45	GC11P036534	1.69
895	MMP2	Matrix Metallopeptidase 2	Protein Coding	52	GC16P055390	1.69
896	ARNT	Aryl Hydrocarbon Receptor Nuclear Translocator	Protein Coding	44	GC01M150809	1.68
897	DHCR7	7-Dehydrocholesterol Reductase	Protein Coding	46	GC11M071428	1.67
898	LCK	LCK Proto-Oncogene, Src Family Tyrosine Kinase	Protein Coding	51	GC01P032251	1.66
899	RAG2	Recombination Activating 2	Protein Coding	42	GC11M036575	1.65
900	PSAP	Prosaposin	Protein Coding	46	GC10M071816	1.65
901	ADH1C	Alcohol Dehydrogenase 1C (Class I), Gamma Polypeptide	Protein Coding	41	GC04M099336	1.65
902	CACNA1B	Calcium Voltage-Gated Channel Subunit Alpha1 B	Protein Coding	49	GC09P137877	1.64
903	FUT2	Fucosyltransferase 2	Protein Coding	44	GC19P048695	1.64
904	BACH2	BTB Domain And CNC Homolog 2	Protein Coding	42	GC06M089926	1.64
905	ADAD1	Adenosine Deaminase Domain Containing 1	Protein Coding	36	GC04P122378	1.64
906	CDKN1A	Cyclin Dependent Kinase Inhibitor 1A	Protein Coding	48	GC06P077011	1.63
907	SLC39A8	Solute Carrier Family 39 Member 8	Protein Coding	44	GC04M102252	1.61
908	SLC39A14	Solute Carrier Family 39 Member 14	Protein Coding	43	GC08P022367	1.61
909	SLC39A13	Solute Carrier Family 39 Member 13	Protein Coding	41	GC11P047407	1.61
910	THEMIS	Thymocyte Selection Associated	Protein Coding	37	GC06M127708	1.61
911	lnc-FLG2-1		RNA Gene	3	GC01M152303	1.61
912	CCR2	C-C Motif Chemokine Receptor 2	Protein Coding	44	GC03P046356	1.6
913	HSPG2	Heparan Sulfate Proteoglycan 2	Protein Coding	47	GC01M021822	1.6
914	GJC2	Gap Junction Protein Gamma 2	Protein Coding	41	GC01P229079	1.6
915	CLC	Charcot-Leyden Crystal Galectin	Protein Coding	40	GC19M061844	1.59
916	ITCH	Itchy E3 Ubiquitin Protein Ligase	Protein Coding	45	GC20P034363	1.59
917	CTNNA2	Catenin Alpha 2	Protein Coding	42	GC02P079185	1.59
918	ALOX5AP	Arachidonate 5-Lipoxygenase Activating Protein	Protein Coding	44	GC13P030713	1.58
919	NELFA	Negative Elongation Factor Complex Member A	Protein Coding	35	GC04M002119	1.56
920	CYP2C19	Cytochrome P450 Family 2 Subfamily C Member 19	Protein Coding	48	GC10P094762	1.56
921	PLEC	Plectin	Protein Coding	42	GC08M144096	1.56
922	AIF1	Allograft Inflammatory Factor 1	Protein Coding	38	GC06P076863	1.55
923	TPSAB1	Tryptase Alpha/Beta 1	Protein Coding	43	GC16P001240	1.54
924	CD151	CD151 Molecule (Raph Blood Group)	Protein Coding	44	GC11P001565	1.53
925	LPIN2	Lipin 2	Protein Coding	42	GC18M002916	1.52
926	RARRES2	Retinoic Acid Receptor Responder 2	Protein Coding	39	GC07M150333	1.52
927	OCLN	Occludin	Protein Coding	44	GC05P069492	1.52
928	NACA2	Nascent Polypeptide Associated Complex Subunit Alpha 2	Protein Coding	33	GC17M061590	1.51
929	BMP6	Bone Morphogenetic Protein 6	Protein Coding	43	GC06P007726	1.51
930	CYP21A2	Cytochrome P450 Family 21 Subfamily A Member 2	Protein Coding	45	GC06P076889	1.51
931	MICB	MHC Class I Polypeptide-Related Sequence B	Protein Coding	41	GC06P076856	1.51
932	HLA-DQA2	Major Histocompatibility Complex, Class II, DQ Alpha 2	Protein Coding	37	GC06P032741	1.51
933	NLRP2	NLR Family Pyrin Domain Containing 2	Protein Coding	41	GC19P054953	1.5
934	CARD8	Caspase Recruitment Domain Family Member 8	Protein Coding	40	GC19M062134	1.5
935	NELFCD	Negative Elongation Factor Complex Member C/D	Protein Coding	36	GC20P058981	1.5
936	MIR144	MicroRNA 144	RNA Gene	19	GC17M033777	1.5
937	TGFA	Transforming Growth Factor Alpha	Protein Coding	45	GC02M070447	1.49
938	ALDH2	Aldehyde Dehydrogenase 2 Family Member	Protein Coding	49	GC12P111766	1.49
939	NOS1	Nitric Oxide Synthase 1	Protein Coding	49	GC12M117208	1.49
940	ATP2A2	ATPase Sarcoplasmic/Endoplasmic Reticulum Ca2+ Transporting 2	Protein Coding	50	GC12P110280	1.48
941	ITGB3	Integrin Subunit Beta 3	Protein Coding	49	GC17P051053	1.48
942	ERCC2	ERCC Excision Repair 2, TFIIH Core Complex Helicase Subunit	Protein Coding	47	GC19M045349	1.47
943	RNASEH2C	Ribonuclease H2 Subunit C	Protein Coding	38	GC11M065714	1.47
944	NPSR1-AS1	NPSR1 Antisense RNA 1	RNA Gene	20	GC07M034387	1.47
945	ESR1	Estrogen Receptor 1	Protein Coding	52	GC06P151656	1.46
946	CLIC1	Chloride Intracellular Channel 1	Protein Coding	41	GC06M061314	1.45
947	PPIA	Peptidylprolyl Isomerase A	Protein Coding	45	GC07P044807	1.45
948	PTPN2	Protein Tyrosine Phosphatase Non-Receptor Type 2	Protein Coding	45	GC18M023298	1.45
949	IFNB1	Interferon Beta 1	Protein Coding	42	GC09M021077	1.44
950	AGT	Angiotensinogen	Protein Coding	48	GC01M230702	1.44
951	CXCL1	C-X-C Motif Chemokine Ligand 1	Protein Coding	41	GC04P073869	1.44
952	TG	Thyroglobulin	Protein Coding	44	GC08P132866	1.43
953	ANXA1	Annexin A1	Protein Coding	48	GC09P073151	1.42

954	BTNL2	Butyrophilin Like 2	Protein Coding	39	GC06M032393	1.42
955	RUNX1	RUNX Family Transcription Factor 1	Protein Coding	48	GC21M034787	1.41
956	CCND1	Cyclin D1	Protein Coding	50	GC11P069641	1.41
957	ADAM10	ADAM Metallopeptidase Domain 10	Protein Coding	51	GC15M058588	1.41
958	ITGA2	Integrin Subunit Alpha 2	Protein Coding	46	GC05P052989	1.41
959	NFKBIA	NFKB Inhibitor Alpha	Protein Coding	49	GC14M035401	1.4
960	LYN	LYN Proto-Oncogene, Src Family Tyrosine Kinase	Protein Coding	48	GC08P055879	1.4
961	NFATC1	Nuclear Factor Of Activated T Cells 1	Protein Coding	46	GC18P079395	1.4
962	NFATC2	Nuclear Factor Of Activated T Cells 2	Protein Coding	44	GC20M051386	1.4
963	MIR124-1	MicroRNA 124-1	RNA Gene	22	GC08M009903	1.4
964	PDGFRA	Platelet Derived Growth Factor Receptor Alpha	Protein Coding	53	GC04P054229	1.39
965	BCL6	BCL6 Transcription Repressor	Protein Coding	44	GC03M187721	1.39
966	NBAS	NBAS Subunit Of NRZ Tethering Complex	Protein Coding	40	GC02M014998	1.39
967	PCDH9	Protocadherin 9	Protein Coding	40	GC13M066302	1.39
968	SCAPER	S-Phase Cyclin A Associated Protein In The ER	Protein Coding	36	GC15M076347	1.39
969	LGMNP1	Legumain Pseudogene 1	Pseudogene	6	GC13M064957	1.39
970	TOR1A	Torsin Family 1 Member A	Protein Coding	45	GC09M129812	1.38
971	GHRL	Ghrelin And Obestatin Prepropeptide	Protein Coding	43	GC03M010285	1.38
972	ASGR1	Asialoglycoprotein Receptor 1	Protein Coding	40	GC17M007173	1.38
973	EDA	Ectodysplasin A	Protein Coding	42	GC0XP069618	1.38
974	ITGA1	Integrin Subunit Alpha 1	Protein Coding	43	GC05P052788	1.37
975	KIR2DL3	Killer Cell Immunoglobulin Like Receptor, Two Ig Domains And Long Cytoplasmic Tail 3	Protein Coding	37	GC19P062908	1.37
976	IKZF3	IKAROS Family Zinc Finger 3	Protein Coding	42	GC17M040974	1.37
977	PROS1	Protein S	Protein Coding	48	GC03M093873	1.37
978	CTSS	Cathepsin S	Protein Coding	44	GC01M150730	1.36
979	AGER	Advanced Glycosylation End-Product Specific Receptor	Protein Coding	44	GC06M032180	1.36
980	LIFR	LIF Receptor Subunit Alpha	Protein Coding	46	GC05M038475	1.36
981	PTAFR	Platelet Activating Factor Receptor	Protein Coding	42	GC01M028147	1.36
982	IL1RL2	Interleukin 1 Receptor Like 2	Protein Coding	42	GC02P102186	1.35
983	MICA	MHC Class I Polypeptide-Related Sequence A	Protein Coding	39	GC06P031399	1.35
984	PTGER2	Prostaglandin E Receptor 2	Protein Coding	48	GC14P052314	1.35
985	HLA-DRA	Major Histocompatibility Complex, Class II, DR Alpha	Protein Coding	46	GC06P032439	1.35
986	CNTF	Ciliary Neurotrophic Factor	Protein Coding	40	GC11P058622	1.35
987	GP9	Glycoprotein IX Platelet	Protein Coding	45	GC03P133378	1.35
988	EDNRA	Endothelin Receptor Type A	Protein Coding	49	GC04P147480	1.34
989	ACY1	Aminoacylase 1	Protein Coding	45	GC03P051983	1.34
990	SERPINE1	Serpin Family E Member 1	Protein Coding	49	GC07P101127	1.33
991	CXCL2	C-X-C Motif Chemokine Ligand 2	Protein Coding	39	GC04M074097	1.32
992	CLEC6A	C-Type Lectin Domain Containing 6A	Protein Coding	36	GC12P008455	1.32
993	SMARCC2	SWI/SNF Related, Matrix Associated, Actin Dependent Regulator Of Chromatin Subfamily C Member 2	Protein Coding	44	GC12M056531	1.31
994	PRMT7	Protein Arginine Methyltransferase 7	Protein Coding	44	GC16P068444	1.31
995	ANKRD1	Ankyrin Repeat Domain 1	Protein Coding	42	GC10M090912	1.31
996	PSORS1C1	Psoriasis Susceptibility 1 Candidate 1	Protein Coding	30	GC06P031114	1.3
997	NQO1	NAD(P)H Quinone Dehydrogenase 1	Protein Coding	47	GC16M069706	1.3
998	CTCF	CCCTC-Binding Factor	Protein Coding	46	GC16P067563	1.29
999	PUS10	Pseudouridine Synthase 10	Protein Coding	35	GC02M060940	1.29
1000	CYP1A2	Cytochrome P450 Family 1 Subfamily A Member 2	Protein Coding	46	GC15P074748	1.29
1001	TNFSF8	TNF Superfamily Member 8	Protein Coding	36	GC09M114893	1.28
1002	FGF7	Fibroblast Growth Factor 7	Protein Coding	41	GC15P049423	1.28
1003	SH2D3C	SH2 Domain Containing 3C	Protein Coding	38	GC09M127738	1.27
1004	CPA3	Carboxypeptidase A3	Protein Coding	41	GC03P148865	1.26
1005	SERPINB2	Serpin Family B Member 2	Protein Coding	43	GC18P063871	1.26
1006	GALR1	Galanin Receptor 1	Protein Coding	44	GC18P077250	1.25
1007	SERPINB8	Serpin Family B Member 8	Protein Coding	42	GC18P063969	1.25
1008	TSHZ1	Teashirt Zinc Finger Homeobox 1	Protein Coding	39	GC18P075210	1.25
1009	NETO1	Neuropilin And Tolloid Like 1	Protein Coding	37	GC18M072742	1.25
1010	SALL3	Spalt Like Transcription Factor 3	Protein Coding	36	GC18P078980	1.25
1011	TULP4	TUB Like Protein 4	Protein Coding	33	GC06P158232	1.25
1012	TTC28	Tetratricopeptide Repeat Domain 28	Protein Coding	33	GC22M027978	1.25
1013	ZNF516	Zinc Finger Protein 516	Protein Coding	33	GC18M076357	1.25
1014	ZNF236	Zinc Finger Protein 236	Protein Coding	33	GC18P076822	1.25
1015	PVALB	Parvalbumin	Protein Coding	39	GC22M036800	1.25
1016	SEMA4F	Ssemaphorin 4F	Protein Coding	38	GC02P074654	1.24
1017	VNN2	Vanin 2	Protein Coding	41	GC06M132743	1.24
1018	CD44	CD44 Molecule (Indian Blood Group)	Protein Coding	46	GC11P035139	1.24
1019	CHRM3	Cholinergic Receptor Muscarinic 3	Protein Coding	48	GC01P239386	1.24
1020	AIM2	Absent In Melanoma 2	Protein Coding	41	GC01M159062	1.24
1021	IL36G	Interleukin 36 Gamma	Protein Coding	37	GC02P112973	1.23
1022	FURIN	Furin, Paired Basic Amino Acid Cleaving Enzyme	Protein Coding	45	GC15P090868	1.23
1023	KRT7	Keratin 7	Protein Coding	42	GC12P052232	1.23
1024	ATP4A	ATPase H+/K+ Transporting Subunit Alpha	Protein Coding	41	GC19M062663	1.23
1025	CMKLR1	Chemerin Chemokine-Like Receptor 1	Protein Coding	40	GC12M108288	1.23
1026	EMCN	Endomucin	Protein Coding	37	GC04M100395	1.23
1027	HCG27	HLA Complex Group 27	RNA Gene	20	GC06P031197	1.22
1028	ITGB1	Integrin Subunit Beta 1	Protein Coding	49	GC10M032932	1.22
1029	DPP10	Dipeptidyl Peptidase Like 10	Protein Coding	41	GC02P114442	1.22
1030	IFN1@	Interferon, Type 1, Cluster	Gene Cluster	5	GC09U990039	1.22
1031	ANAPC1	Anaphase Promoting Complex Subunit 1	Protein Coding	40	GC02M111611	1.21
1032	MIP	Major Intrinsic Protein Of Lens Fiber	Protein Coding	41	GC12M056449	1.21
1033	FOS	Fos Proto-Oncogene, AP-1 Transcription Factor Subunit	Protein Coding	50	GC14P075278	1.2
1034	SRC	SRC Proto-Oncogene, Non-Receptor Tyrosine Kinase	Protein Coding	50	GC20P037344	1.2
1035	GPNMB	Glycoprotein Nmb	Protein Coding	44	GC07P023238	1.2
1036	ABCC6	ATP Binding Cassette Subfamily C Member 6	Protein Coding	46	GC16M016148	1.2
1037	TTR	Transthyretin	Protein Coding	48	GC18P031557	1.19
1038	FGFR1	Fibroblast Growth Factor Receptor 1	Protein Coding	53	GC08M038400	1.19
1039	PDGFRB	Platelet Derived Growth Factor Receptor Beta	Protein Coding	53	GC05M150113	1.19
1040	NF1	Neurofibromin 1	Protein Coding	48	GC17P031094	1.19

1041	KCNJ1	Potassium Inwardly Rectifying Channel Subfamily J Member 1	Protein Coding	48	GC11M128741	1.19
1042	CHD7	Chromodomain Helicase DNA Binding Protein 7	Protein Coding	45	GC08P060678	1.19
1043	PON1	Paraoxonase 1	Protein Coding	47	GC07M095297	1.18
1044	TIMELESS	Timeless Circadian Regulator	Protein Coding	38	GC12M056416	1.18
1045	MAP3K7	Mitogen-Activated Protein Kinase Kinase Kinase 7	Protein Coding	50	GC06M090513	1.18
1046	SMAD2	SMAD Family Member 2	Protein Coding	49	GC18M047809	1.17
1047	SFTPD	Surfactant Protein D	Protein Coding	43	GC10M079937	1.17
1048	LZTR1	Leucine Zipper Like Transcription Regulator 1	Protein Coding	42	GC22P033344	1.16
1049	IL1F10	Interleukin 1 Family Member 10	Protein Coding	38	GC02P113067	1.16
1050	FN1	Fibronectin 1	Protein Coding	49	GC02M215360	1.16
1051	SMS	Spermine Synthase	Protein Coding	43	GC0XP021958	1.15
1052	ALAS2	5'-Aminolevulinate Synthase 2	Protein Coding	44	GC0XM055009	1.15
1053	CPOX	Coproporphyrinogen Oxidase	Protein Coding	43	GC03M098576	1.15
1054	PRKCB	Protein Kinase C Beta	Protein Coding	46	GC16P024142	1.15
1055	CTSD	Cathepsin D	Protein Coding	50	GC11M001752	1.15
1056	CASP7	Caspase 7	Protein Coding	48	GC10P113679	1.15
1057	MAOA	Monoamine Oxidase A	Protein Coding	48	GC0XP043654	1.15
1058	ELOVL5	ELOVL Fatty Acid Elongase 5	Protein Coding	42	GC06M053267	1.15
1059	CD53	CD53 Molecule	Protein Coding	38	GC01P110871	1.15
1060	TSPAN32	Tetraspanin 32	Protein Coding	36	GC11P002302	1.15
1061	CXCR5	C-X-C Motif Chemokine Receptor 5	Protein Coding	40	GC11P118912	1.14
1062	DUSP1	Dual Specificity Phosphatase 1	Protein Coding	46	GC05M172768	1.14
1063	CXCL16	C-X-C Motif Chemokine Ligand 16	Protein Coding	39	GC17M004733	1.14
1064	IFNL1	Interferon Lambda 1	Protein Coding	34	GC19P039296	1.13
1065	ERV9-1	Endogenous Retrovirus Group 9 Member 1	Uncategorized	7	GC11U901873	1.13
1066	PTGDS	Prostaglandin D2 Synthase	Protein Coding	45	GC09P137026	1.13
1067	CAMK4	Calcium/Calmodulin Dependent Protein Kinase IV	Protein Coding	45	GC05P111223	1.13
1068	HLA-DRB5	Major Histocompatibility Complex, Class II, DR Beta 5	Protein Coding	40	GC06M061346	1.13
1069	SKIV2L	Ski2 Like RNA Helicase	Protein Coding	42	GC06P076887	1.12
1070	SLC6A15	Solute Carrier Family 6 Member 15	Protein Coding	41	GC12M084859	1.12
1071	MDC1	Mediator Of DNA Damage Checkpoint 1	Protein Coding	41	GC06M061228	1.12
1072	PSORS1C2	Psoriasis Susceptibility 1 Candidate 2	Protein Coding	31	GC06M031137	1.12
1073	SP1	Sp1 Transcription Factor	Protein Coding	45	GC12P053380	1.12
1074	ALOX15	Arachidonate 15-Lipoxygenase	Protein Coding	45	GC17M004630	1.12
1075	PIK3CG	Phosphatidylinositol-4,5-Bisphosphate 3-Kinase Catalytic Subunit Gamma	Protein Coding	47	GC07P106865	1.12
1076	CXCL5	C-X-C Motif Chemokine Ligand 5	Protein Coding	40	GC04M073995	1.12
1077	SOS1	SOS Ras/Rac Guanine Nucleotide Exchange Factor 1	Protein Coding	47	GC02M039016	1.12
1078	PCDH1	Protocadherin 1	Protein Coding	38	GC05M141900	1.11
1079	ACP1	Acid Phosphatase 1	Protein Coding	43	GC02P000293	1.11
1080	WIF1	WNT Inhibitory Factor 1	Protein Coding	44	GC12M065050	1.1
1081	COL10A1	Collagen Type X Alpha 1 Chain	Protein Coding	42	GC06M116118	1.1
1082	CRY2	Cryptochrome Circadian Regulator 2	Protein Coding	40	GC11P046397	1.1
1083	COL8A1	Collagen Type VIII Alpha 1 Chain	Protein Coding	39	GC03P099638	1.1
1084	CDHR3	Cadherin Related Family Member 3	Protein Coding	36	GC07P105876	1.1
1085	CTNNA3	Catenin Alpha 3	Protein Coding	40	GC10M065912	1.09
1086	CST3	Cystatin C	Protein Coding	44	GC20M023646	1.09
1087	CASR	Calcium Sensing Receptor	Protein Coding	50	GC03P122183	1.09
1088	POLE	DNA Polymerase Epsilon, Catalytic Subunit	Protein Coding	48	GC12M132666	1.09
1089	ERCC3	ERCC Excision Repair 3, TFIIH Core Complex Helicase Subunit	Protein Coding	47	GC02M127257	1.09
1090	TAF1	TATA-Box Binding Protein Associated Factor 1	Protein Coding	45	GC0XP071366	1.09
1091	GTF2E2	General Transcription Factor IIE Subunit 2	Protein Coding	42	GC08M030578	1.09
1092	SRP54	Signal Recognition Particle 54	Protein Coding	42	GC14P034981	1.09
1093	NUP107	Nucleoporin 107	Protein Coding	42	GC12P068686	1.09
1094	SIK3	SIK Family Kinase 3	Protein Coding	42	GC11M116843	1.09
1095	SBDS	SBDS Ribosome Maturation Factor	Protein Coding	41	GC07M066987	1.09
1096	TBCK	TBC1 Domain Containing Kinase	Protein Coding	40	GC04M106041	1.09
1097	GTF2H5	General Transcription Factor IIH Subunit 5	Protein Coding	38	GC06P158168	1.09
1098	RNF113A	Ring Finger Protein 113A	Protein Coding	37	GC0XM119870	1.09
1099	MPLKIP	M-Phase Specific PLK1 Interacting Protein	Protein Coding	36	GC07M040126	1.09
1100	TARS1	Threonyl-TRNA Synthetase 1	Protein Coding	35	GC05P033441	1.09
1101	DNAJC21	DnaJ Heat Shock Protein Family (Hsp40) Member C21	Protein Coding	35	GC05P034929	1.09
1102	EFL1	Elongation Factor Like GTPase 1	Protein Coding	32	GC15M082131	1.09
1103	RNU4ATAC	RNA, U4atac Small Nuclear (U12-Dependent Splicing)	RNA Gene	21	GC02P121601	1.09
1104	MIR31	MicroRNA 31	RNA Gene	21	GC09M021705	1.09
1105	GP1BA	Glycoprotein Ib Platelet Subunit Alpha	Protein Coding	46	GC17P004932	1.08
1106	PLA2G2A	Phospholipase A2 Group IIA	Protein Coding	46	GC01M019975	1.08
1107	FKBP5	FKBP Prolyl Isomerase 5	Protein Coding	45	GC06M061437	1.08
1108	CCN2	Cellular Communication Network Factor 2	Protein Coding	38	GC06M131948	1.08
1109	SMAD7	SMAD Family Member 7	Protein Coding	44	GC18M048919	1.08
1110	PLS3	Plastin 3	Protein Coding	42	GC0XP115560	1.08
1111	PTGER4	Prostaglandin E Receptor 4	Protein Coding	45	GC05P040679	1.07
1112	EGR1	Early Growth Response 1	Protein Coding	44	GC05P138465	1.07
1113	MYLK	Myosin Light Chain Kinase	Protein Coding	51	GC03M123610	1.07
1114	ARG2	Arginase 2	Protein Coding	44	GC14P067619	1.07
1115	MMEL1	Membrane Metalloendopeptidase Like 1	Protein Coding	37	GC01M002590	1.07
1116	SLC22A4	Solute Carrier Family 22 Member 4	Protein Coding	44	GC05P132294	1.06
1117	CALB2	Calbindin 2	Protein Coding	40	GC16P071358	1.06
1118	STEAP2-AS1	STEAP2 Antisense RNA 1	RNA Gene	15	GC07M089882	1.06
1119	POU2F1	POU Class 2 Homeobox 1	Protein Coding	43	GC01P167190	1.05
1120	KRT8	Keratin 8	Protein Coding	46	GC12M052897	1.04
1121	DENND1B	DENN Domain Containing 1B	Protein Coding	35	GC01M197473	1.04
1122	REN	Renin	Protein Coding	48	GC01M204154	1.04
1123	APTX	Aprataxin	Protein Coding	44	GC09M032886	1.04
1124	FERMT3	FERM Domain Containing Kindlin 3	Protein Coding	43	GC11P064286	1.04
1125	HRH2	Histamine Receptor H2	Protein Coding	44	GC05P175659	1.04
1126	TET2	Tet Methylcytosine Dioxygenase 2	Protein Coding	45	GC04P105145	1.03
1127	AIP	Aryl Hydrocarbon Receptor Interacting Protein	Protein Coding	44	GC11P067468	1.03

1128	ASXL1	ASXL Transcriptional Regulator 1	Protein Coding	43	GC20P032585	1.03
1129	CISH	Cytokine Inducible SH2 Containing Protein	Protein Coding	45	GC03M050974	1.03
1130	LIF	LIF Interleukin 6 Family Cytokine	Protein Coding	41	GC22M030240	1.03
1131	MAP3K11	Mitogen-Activated Protein Kinase Kinase Kinase 11	Protein Coding	46	GC11M084684	1.03
1132	NEU1	Neuraminidase 1	Protein Coding	44	GC06M031857	1.02
1133	PLCD1	Phospholipase C Delta 1	Protein Coding	47	GC03M038008	1.02
1134	HACE1	HECT Domain And Ankyrin Repeat Containing E3 Ubiquitin Protein Ligase 1	Protein Coding	42	GC06M104728	1.02
1135	LIN28B	Lin-28 Homolog B	Protein Coding	41	GC06P104949	1.02
1136	SQSTM1	Sequestosome 1	Protein Coding	47	GC05P179806	1.02
1137	SEMA4D	Semaphorin 4D	Protein Coding	45	GC09M089360	1.02
1138	TNFSF15	TNF Superfamily Member 15	Protein Coding	43	GC09M114784	1.02
1139	RAC1	Rac Family Small GTPase 1	Protein Coding	48	GC07P006377	1.01
1140	TNFRSF13B	TNF Receptor Superfamily Member 13B	Protein Coding	46	GC17M016929	1.01
1141	APCS	Amyloid P Component, Serum	Protein Coding	42	GC01P159587	1.01
1142	FGFR2	Fibroblast Growth Factor Receptor 2	Protein Coding	53	GC10M121478	1
1143	AKT3	AKT Serine/Threonine Kinase 3	Protein Coding	52	GC01M243488	1
1144	BCL3	BCL3 Transcription Coactivator	Protein Coding	41	GC19P044742	1
1145	RAF1	Raf-1 Proto-Oncogene, Serine/Threonine Kinase	Protein Coding	52	GC03M012583	1
1146	PTPN11	Protein Tyrosine Phosphatase Non-Receptor Type 11	Protein Coding	51	GC12P112418	1
1147	IL18BP	Interleukin 18 Binding Protein	Protein Coding	40	GC11P071998	1
1148	CHST8	Carbohydrate Sulfotransferase 8	Protein Coding	41	GC19P033621	0.99
1149	KIR2DL2	Killer Cell Immunoglobulin Like Receptor, Two Ig Domains And Long Cytoplasmic Tail 2	Protein Coding	25	GC19Mr00108	0.99
1150	MIR142	MicroRNA 142	RNA Gene	21	GC17M058331	0.98
1151	MIRLET7E	MicroRNA Let-7e	RNA Gene	21	GC19P053332	0.98
1152	MIR197	MicroRNA 197	RNA Gene	21	GC01P109549	0.98
1153	MIR381	MicroRNA 381	RNA Gene	18	GC14P109079	0.98
1154	MIR518B	MicroRNA 518b	RNA Gene	17	GC19P062500	0.98
1155	CBL	Cbl Proto-Oncogene	Protein Coding	50	GC11P119206	0.98
1156	HAMP	Hepcidin Antimicrobial Peptide	Protein Coding	44	GC19P051384	0.97
1157	VWF	Von Willebrand Factor	Protein Coding	48	GC12M005917	0.97
1158	NR1I2	Nuclear Receptor Subfamily 1 Group I Member 2	Protein Coding	45	GC03P119780	0.97
1159	MMP7	Matrix Metallopeptidase 7	Protein Coding	47	GC11M102425	0.97
1160	H2AX	H2A.X Variant Histone	Protein Coding	35	GC11M119155	0.97
1161	MIR145	MicroRNA 145	RNA Gene	23	GC05P149430	0.96
1162	ADRB1	Adrenoceptor Beta 1	Protein Coding	48	GC10P114044	0.96
1163	ADORA3	Adenosine A3 Receptor	Protein Coding	45	GC01M111499	0.96
1164	HLX	H2.0 Like Homeobox	Protein Coding	40	GC01P220879	0.96
1165	OSM	Oncostatin M	Protein Coding	42	GC22M030262	0.95
1166	SETDB2	SET Domain Bifurcated Histone Lysine Methyltransferase 2	Protein Coding	36	GC13P049444	0.94
1167	FOXJ1	Forkhead Box J1	Protein Coding	37	GC17M076136	0.94
1168	KMO	Kynurenine 3-Monooxygenase	Protein Coding	44	GC01P241532	0.94
1169	PRKCD	Protein Kinase C Delta	Protein Coding	52	GC03P053156	0.93
1170	PTGER3	Prostaglandin E Receptor 3	Protein Coding	46	GC01M070852	0.93
1171	SH2B3	SH2B Adaptor Protein 3	Protein Coding	46	GC12P111405	0.92
1172	HLA-E	Major Histocompatibility Complex, Class I, E	Protein Coding	42	GC06P076836	0.92
1173	PRDM1	PR/SET Domain 1	Protein Coding	44	GC06P105993	0.91
1174	ATG5	Autophagy Related 5	Protein Coding	43	GC06M106045	0.91
1175	AFF4	AF4/FMR2 Family Member 4	Protein Coding	41	GC05M132875	0.91
1176	SNRNP70	Small Nuclear Ribonucleoprotein U1 Subunit 70	Protein Coding	38	GC19P049085	0.91
1177	OTULINL	OTU Deubiquitinase With Linear Linkage Specificity Like	Protein Coding	27	GC05P014582	0.91
1178	RARA	Retinoic Acid Receptor Alpha	Protein Coding	48	GC17P040309	0.91
1179	CCHCR1	Coiled-Coil Alpha-Helical Rod Protein 1	Protein Coding	38	GC06M061251	0.91
1180	LIG1	DNA Ligase 1	Protein Coding	45	GC19M048115	0.9
1181	RELCH	RAB11 Binding And LisH Domain, Coiled-Coil And HEAT Repeat Containing	Protein Coding	26	GC18P062188	0.9
1182	S100A7A	S100 Calcium Binding Protein A7A	Protein Coding	34	GC01P153416	0.9
1183	NCR3LG1	Natural Killer Cell Cytotoxicity Receptor 3 Ligand 1	Protein Coding	32	GC11P017351	0.9
1184	SLPI	Secretory Leukocyte Peptidase Inhibitor	Protein Coding	40	GC20M045252	0.9
1185	ICAM2	Intercellular Adhesion Molecule 2	Protein Coding	44	GC17M064002	0.9
1186	TNFRSF9	TNF Receptor Superfamily Member 9	Protein Coding	42	GC01M007915	0.9
1187	TRPV4	Transient Receptor Potential Cation Channel Subfamily V Member 4	Protein Coding	48	GC12M109783	0.88
1188	CYCS	Cytochrome C, Somatic	Protein Coding	47	GC07M025118	0.88
1189	MC2R	Melanocortin 2 Receptor	Protein Coding	47	GC18M023304	0.88
1190	NEDD4L	NEDD4 Like E3 Ubiquitin Protein Ligase	Protein Coding	46	GC18P058044	0.88
1191	NR3C2	Nuclear Receptor Subfamily 3 Group C Member 2	Protein Coding	46	GC04M148078	0.88
1192	ETV6	ETS Variant Transcription Factor 6	Protein Coding	46	GC12P011649	0.88
1193	CASP9	Caspase 9	Protein Coding	46	GC01M015491	0.88
1194	RASA1	RAS P21 Protein Activator 1	Protein Coding	46	GC05P087267	0.88
1195	HSD3B2	Hydroxy-Delta-5-Steroid Dehydrogenase, 3 Beta- And Steroid Delta-Isomerase 2	Protein Coding	45	GC01P119414	0.88
1196	MRAS	Muscle RAS Oncogene Homolog	Protein Coding	45	GC03P138347	0.88
1197	ETFDH	Electron Transfer Flavoprotein Dehydrogenase	Protein Coding	45	GC04P158672	0.88
1198	PPP1CB	Protein Phosphatase 1 Catalytic Subunit Beta	Protein Coding	45	GC02P028752	0.88
1199	SOS2	SOS Ras/Rho Guanine Nucleotide Exchange Factor 2	Protein Coding	44	GC14M050117	0.88
1200	ATP6V1B1	ATPase H+ Transporting V1 Subunit B1	Protein Coding	44	GC02P070935	0.88
1201	ARAF	A-Raf Proto-Oncogene, Serine/Threonine Kinase	Protein Coding	44	GC0XP047562	0.88
1202	KAT6B	Lysine Acetyltransferase 6B	Protein Coding	43	GC10P074860	0.88
1203	SYNGAP1	Synaptic Ras GTPase Activating Protein 1	Protein Coding	43	GC06P033419	0.88
1204	PDSS1	Decaprenyl Diphosphate Synthase Subunit 1	Protein Coding	42	GC10P026697	0.88
1205	RHBDF2	Rhomboid 5 Homolog 2	Protein Coding	42	GC17M076470	0.88
1206	RRAS	RAS Related	Protein Coding	42	GC19M049635	0.88
1207	ASIC1	Acid Sensing Ion Channel Subunit 1	Protein Coding	42	GC12P050057	0.88
1208	VAPA	VAMP Associated Protein A	Protein Coding	42	GC18P009904	0.88
1209	PDSS2	Decaprenyl Diphosphate Synthase Subunit 2	Protein Coding	41	GC06M107152	0.88
1210	SPRED1	Sprouty Related EVH1 Domain Containing 1	Protein Coding	41	GC15P038252	0.88
1211	SPRED2	Sprouty Related EVH1 Domain Containing 2	Protein Coding	41	GC02M065307	0.88
1212	SPRY1	Sprouty RTK Signaling Antagonist 1	Protein Coding	41	GC04P123396	0.88
1213	TMEM43	Transmembrane Protein 43	Protein Coding	41	GC03P014124	0.88
1214	COQ2	Coenzyme Q2, Polyprenyltransferase	Protein Coding	41	GC04M083261	0.88

1215	A2ML1	Alpha-2-Macroglobulin Like 1	Protein Coding	41	GC12P008822	0.88
1216	RALGDS	Ral Guanine Nucleotide Dissociation Stimulator	Protein Coding	41	GC09M133097	0.88
1217	KCND1	Potassium Voltage-Gated Channel Subfamily D Member 1	Protein Coding	40	GC0XM049014	0.88
1218	PDPN	Podoplanin	Protein Coding	40	GC01P013583	0.88
1219	RHBG	Rh Family B Glycoprotein	Protein Coding	40	GC01P156366	0.88
1220	RASA2	RAS P21 Protein Activator 2	Protein Coding	40	GC03P141487	0.88
1221	FIP1L1	Factor Interacting With PAPOLA And CPSF1	Protein Coding	39	GC04P053418	0.88
1222	ASIC2	Acid Sensing Ion Channel Subunit 2	Protein Coding	39	GC17M033013	0.88
1223	KRT6C	Keratin 6C	Protein Coding	38	GC12M052468	0.88
1224	CCRL2	C-C Motif Chemokine Receptor Like 2	Protein Coding	38	GC03P046407	0.88
1225	ARMC9	Armadillo Repeat Containing 9	Protein Coding	38	GC02P231198	0.88
1226	KLHDC2	Kelch Domain Containing 2	Protein Coding	37	GC14P049767	0.88
1227	CFC1	Cripto, FRL-1, Cryptic Family 1	Protein Coding	37	GC02M130592	0.88
1228	ASZ1	Ankyrin Repeat, SAM And Basic Leucine Zipper Domain Containing 1	Protein Coding	37	GC07M117363	0.88
1229	SLC26A11	Solute Carrier Family 26 Member 11	Protein Coding	37	GC17P080219	0.88
1230	FEM1C	Fem-1 Homolog C	Protein Coding	36	GC05M115520	0.88
1231	SPRED3	Sprouty Related EVH1 Domain Containing 3	Protein Coding	36	GC19P038388	0.88
1232	TOR3A	Torsin Family 3 Member A	Protein Coding	36	GC01P179081	0.88
1233	ERAS	ES Cell Expressed Ras	Protein Coding	36	GC0XP050318	0.88
1234	COQ8A	Coenzyme Q8A	Protein Coding	36	GC01P226939	0.88
1235	SPATA22	Spermatogenesis Associated 22	Protein Coding	35	GC17M003440	0.88
1236	CTTNBP2	Cortactin Binding Protein 2	Protein Coding	35	GC07M117710	0.88
1237	KLHDC3	Kelch Domain Containing 3	Protein Coding	34	GC06P043014	0.88
1238	TENM4	Teneurin Transmembrane Protein 4	Protein Coding	34	GC11M078652	0.88
1239	ASIC5	Acid Sensing Ion Channel Subunit Family Member 5	Protein Coding	34	GC04M155829	0.88
1240	YIPF2	Yip1 Domain Family Member 2	Protein Coding	34	GC19M011046	0.88
1241	KIAA0895	KIAA0895	Protein Coding	33	GC07M036324	0.88
1242	ACTRT1	Actin Related Protein T1	Protein Coding	32	GC0XM128050	0.88
1243	RMDN1	Regulator Of Microtubule Dynamics 1	Protein Coding	32	GC08M086407	0.88
1244	PLAAT2	Phospholipase A And Acyltransferase 2	Protein Coding	25	GC11M063552	0.88
1245	DNAI7	Dynein Axonemal Intermediate Chain 7	Protein Coding	24	GC12M025110	0.88
1246	MIR499A	MicroRNA 499a	RNA Gene	22	GC20P034990	0.88
1247	USP8	Ubiquitin Specific Peptidase 8	Protein Coding	47	GC15P050424	0.88
1248	CDH5	Cadherin 5	Protein Coding	46	GC16P066366	0.87
1249	MIR143	MicroRNA 143	RNA Gene	24	GC05P149410	0.87
1250	NCSTN	Nicastrin	Protein Coding	46	GC01P160343	0.87
1251	SDHC	Succinate Dehydrogenase Complex Subunit C	Protein Coding	44	GC01P161314	0.87
1252	RFX5	Regulatory Factor X5	Protein Coding	41	GC01M151340	0.87
1253	PRKCQ	Protein Kinase C Theta	Protein Coding	48	GC10M006393	0.87
1254	PFKFB3	6-Phosphofructo-2-Kinase/Fructose-2,6-Biphosphatase 3	Protein Coding	44	GC10P006144	0.87
1255	TLR5	Toll Like Receptor 5	Protein Coding	46	GC01M223643	0.86
1256	HJV	Hemojuvelin BMP Co-Receptor	Protein Coding	36	GC01M146576	0.85
1257	RET	Ret Proto-Oncogene	Protein Coding	52	GC10P043121	0.85
1258	HDAC2	Histone Deacetylase 2	Protein Coding	50	GC06M113933	0.85
1259	KRT18	Keratin 18	Protein Coding	48	GC12P052948	0.85
1260	PDE4D	Phosphodiesterase 4D	Protein Coding	48	GC05M058969	0.85
1261	KLK3	Kallikrein Related Peptidase 3	Protein Coding	46	GC19P050854	0.85
1262	FOLH1	Folate Hydrolase 1	Protein Coding	46	GC11M084355	0.85
1263	ADORA1	Adenosine A1 Receptor	Protein Coding	46	GC01P203090	0.85
1264	CYP3A5	Cytochrome P450 Family 3 Subfamily A Member 5	Protein Coding	46	GC07M099648	0.85
1265	ADORA2B	Adenosine A2b Receptor	Protein Coding	46	GC17P015927	0.85
1266	ADORA2A	Adenosine A2a Receptor	Protein Coding	45	GC22P024417	0.85
1267	ADCY9	Adenylate Cyclase 9	Protein Coding	45	GC16M003953	0.85
1268	ARRB2	Arrestin Beta 2	Protein Coding	45	GC17P004711	0.85
1269	EPHX1	Epoxide Hydrolase 1	Protein Coding	45	GC01P225810	0.85
1270	TGFBI	Transforming Growth Factor Beta Induced	Protein Coding	45	GC05P136027	0.85
1271	SFTPB	Surfactant Protein B	Protein Coding	44	GC02M085657	0.85
1272	PDE4B	Phosphodiesterase 4B	Protein Coding	44	GC01P065792	0.85
1273	SERPINA6	Serpin Family A Member 6	Protein Coding	44	GC14M099728	0.85
1274	TNFRSF13C	TNF Receptor Superfamily Member 13C	Protein Coding	43	GC22M055099	0.85
1275	ADAM8	ADAM Metallopeptidase Domain 8	Protein Coding	42	GC10M133262	0.85
1276	TACR2	Tachykinin Receptor 2	Protein Coding	42	GC10M069403	0.85
1277	SLC7A2	Solute Carrier Family 7 Member 2	Protein Coding	42	GC08P017497	0.85
1278	ALOX15B	Arachidonate 15-Lipoxygenase Type B	Protein Coding	41	GC17P008039	0.85
1279	CLCA1	Chloride Channel Accessory 1	Protein Coding	41	GC01P086468	0.85
1280	GLCCI1	Glucocorticoid Induced 1	Protein Coding	38	GC07P007974	0.85
1281	RASGRP4	RAS Guanyl Releasing Protein 4	Protein Coding	37	GC19M038409	0.85
1282	DCAF8	DDB1 And CUL4 Associated Factor 8	Protein Coding	37	GC01M160215	0.85
1283	EPRS1	Glutamyl-Prolyl-TRNA Synthetase 1	Protein Coding	36	GC01M219969	0.85
1284	METRNL	Meteorin Like, Glial Cell Differentiation Regulator	Protein Coding	35	GC17P083079	0.85
1285	SPATS2L	Spermatogenesis Associated Serine Rich 2 Like	Protein Coding	34	GC02P200305	0.85
1286	COL26A1	Collagen Type XXVI Alpha 1 Chain	Protein Coding	34	GC07P101362	0.85
1287	ZGRF1	Zinc Finger GRF-Type Containing 1	Protein Coding	32	GC04M112539	0.85
1288	C5orf46	Chromosome 5 Open Reading Frame 46	Protein Coding	30	GC05M147850	0.85
1289	PVT1	Pvt1 Oncogene	RNA Gene	26	GC08P127849	0.85
1290	CXCR6	C-X-C Motif Chemokine Receptor 6	Protein Coding	39	GC03P046271	0.85
1291	CDKN2B	Cyclin Dependent Kinase Inhibitor 2B	Protein Coding	47	GC09M022002	0.84
1292	FGF8	Fibroblast Growth Factor 8	Protein Coding	46	GC10M101770	0.84
1293	TNFSF11	TNF Superfamily Member 11	Protein Coding	46	GC13P042562	0.84
1294	PIGT	Phosphatidylinositol Glycan Anchor Biosynthesis Class T	Protein Coding	40	GC20P045416	0.84
1295	PIGG	Phosphatidylinositol Glycan Anchor Biosynthesis Class G	Protein Coding	38	GC04P000727	0.84
1296	GTF2IRD2	GTF2I Repeat Domain Containing 2	Protein Coding	34	GC07M074796	0.84
1297	MX1	MX Dynamin Like GTPase 1	Protein Coding	41	GC21P041420	0.84
1298	SAT1	Spermidine/Spermine N1-Acetyltransferase 1	Protein Coding	44	GC0XP023784	0.84
1299	CHST6	Carbohydrate Sulfotransferase 6	Protein Coding	44	GC16M075472	0.84
1300	MBTPS1	Membrane Bound Transcription Factor Peptidase, Site 1	Protein Coding	44	GC16M084053	0.84
1301	KRT3	Keratin 3	Protein Coding	41	GC12M052789	0.84

1302	KRT12	Keratin 12	Protein Coding	40	GC17M040861	0.84
1303	PAOX	Polyamine Oxidase	Protein Coding	40	GC10P133379	0.84
1304	DOHH	Deoxyhypusine Hydroxylase	Protein Coding	34	GC19M004513	0.84
1305	SCARNA23	Small Cajal Body-Specific RNA 23	RNA Gene	17	GC0XP024744	0.84
1306	CADM1	Cell Adhesion Molecule 1	Protein Coding	42	GC11M115169	0.84
1307	CLEC4D	C-Type Lectin Domain Family 4 Member D	Protein Coding	38	GC12P008509	0.83
1308	FGA	Fibrinogen Alpha Chain	Protein Coding	48	GC04M154583	0.8
1309	BCHE	Butyrylcholinesterase	Protein Coding	49	GC03M165772	0.8
1310	F2RL3	F2R Like Thrombin Or Trypsin Receptor 3	Protein Coding	45	GC19P016888	0.8
1311	SMOC1	SPARC Related Modular Calcium Binding 1	Protein Coding	41	GC14P069854	0.8
1312	IRAK2	Interleukin 1 Receptor Associated Kinase 2	Protein Coding	40	GC03P012054	0.8
1313	UCN	Urocortin	Protein Coding	36	GC02M027308	0.8
1314	TBXT	T-Box Transcription Factor T	Protein Coding	34	GC06M166158	0.8
1315	CCL23	C-C Motif Chemokine Ligand 23	Protein Coding	34	GC17M036013	0.8
1316	UCN2	Urocortin 2	Protein Coding	33	GC03M048561	0.8
1317	MIR424	MicroRNA 424	RNA Gene	18	GC0XM134685	0.8
1318	ERCC6	ERCC Excision Repair 6, Chromatin Remodeling Factor	Protein Coding	45	GC10M049454	0.79
1319	SETBP1	SET Binding Protein 1	Protein Coding	41	GC18P044680	0.79
1320	CD48	CD48 Molecule	Protein Coding	38	GC01M160648	0.79
1321	ZMPSTE24	Zinc Metallopeptidase STE24	Protein Coding	41	GC01P040258	0.79
1322	BSG	Basigin (Ok Blood Group)	Protein Coding	43	GC19P000571	0.78
1323	SDAD1	SDA1 Domain Containing 1	Protein Coding	33	GC04M075940	0.78
1324	AKT1	AKT Serine/Threonine Kinase 1	Protein Coding	52	GC14M104769	0.77
1325	STK4	Serine/Threonine Kinase 4	Protein Coding	48	GC20P044966	0.76
1326	MPL	MPL Proto-Oncogene, Thrombopoietin Receptor	Protein Coding	46	GC01P043337	0.76
1327	MIR34A	MicroRNA 34a	RNA Gene	23	GC01M009151	0.76
1328	XRCC3	X-Ray Repair Cross Complementing 3	Protein Coding	42	GC14M103697	0.75
1329	TGIF1	TGFB Induced Factor Homeobox 1	Protein Coding	45	GC18P003411	0.75
1330	TOP2A	DNA Topoisomerase II Alpha	Protein Coding	49	GC17M040388	0.75
1331	CEBPA	CCAAT Enhancer Binding Protein Alpha	Protein Coding	46	GC19M033299	0.75
1332	CREB1	CAMP Responsive Element Binding Protein 1	Protein Coding	48	GC02P207529	0.73
1333	PTPN6	Protein Tyrosine Phosphatase Non-Receptor Type 6	Protein Coding	48	GC12P018571	0.71
1334	GSK3B	Glycogen Synthase Kinase 3 Beta	Protein Coding	48	GC03M119821	0.71
1335	FASN	Fatty Acid Synthase	Protein Coding	48	GC17M082078	0.71
1336	YWHAE	Tyrosine 3-Monooxygenase/Tryptophan 5-Monooxygenase Activation Protein Epsilon	Protein Coding	48	GC17M002492	0.71
1337	CD81	CD81 Molecule	Protein Coding	45	GC11P002505	0.71
1338	CTNND1	Catenin Delta 1	Protein Coding	45	GC11P058081	0.71
1339	MCL1	MCL1 Apoptosis Regulator, BCL2 Family Member	Protein Coding	45	GC01M151420	0.71
1340	IL1RAP	Interleukin 1 Receptor Accessory Protein	Protein Coding	44	GC03P190514	0.71
1341	PARK7	Parkinsonism Associated Deglycase	Protein Coding	44	GC01P008078	0.71
1342	SEMA4A	Semaphorin 4A	Protein Coding	44	GC01P156147	0.71
1343	TCF7	Transcription Factor 7	Protein Coding	44	GC05P134114	0.71
1344	CD9	CD9 Molecule	Protein Coding	42	GC12P018518	0.71
1345	SCN7A	Sodium Voltage-Gated Channel Alpha Subunit 7	Protein Coding	41	GC02M166403	0.71
1346	CLCA2	Chloride Channel Accessory 2	Protein Coding	41	GC01P086424	0.71
1347	MARCO	Macrophage Receptor With Collagenous Structure	Protein Coding	41	GC02P118942	0.71
1348	SLC25A17	Solute Carrier Family 25 Member 17	Protein Coding	38	GC22M055093	0.71
1349	PLXNB2	Plexin B2	Protein Coding	37	GC22M050274	0.71
1350	LOC107880064	IVL Promoter Region	Biological Region	1	GC01P152906	0.71
1351	HSPA4	Heat Shock Protein Family A (Hsp70) Member 4	Protein Coding	41	GC05P133051	0.71
1352	TEC	Tec Protein Tyrosine Kinase	Protein Coding	45	GC04M048137	0.7
1353	THBD	Thrombomodulin	Protein Coding	45	GC20M023026	0.7
1354	MITF	Melanocyte Inducing Transcription Factor	Protein Coding	47	GC03P069788	0.69
1355	SOX10	SRY-Box Transcription Factor 10	Protein Coding	45	GC22M056089	0.69
1356	NGLY1	N-Glycanase 1	Protein Coding	44	GC03M025718	0.69
1357	SNRPN	Small Nuclear Ribonucleoprotein Polypeptide N	Protein Coding	44	GC15P024823	0.69
1358	HECW2	HECT, C2 And WW Domain Containing E3 Ubiquitin Protein Ligase 2	Protein Coding	41	GC02M196194	0.69
1359	ALPL	Alkaline Phosphatase, Biomineralization Associated	Protein Coding	51	GC01P021508	0.69
1360	GATA4	GATA Binding Protein 4	Protein Coding	48	GC08P011676	0.69
1361	TNFRSF11B	TNF Receptor Superfamily Member 11b	Protein Coding	48	GC08M118923	0.69
1362	RUNX2	RUNX Family Transcription Factor 2	Protein Coding	46	GC06P077087	0.69
1363	HBB	Hemoglobin Subunit Beta	Protein Coding	45	GC11M006229	0.69
1364	SLC7A7	Solute Carrier Family 7 Member 7	Protein Coding	45	GC14M022773	0.69
1365	AP2S1	Adaptor Related Protein Complex 2 Subunit Sigma 1	Protein Coding	41	GC19M046838	0.69
1366	STX3	Syntaxin 3	Protein Coding	41	GC11P059713	0.69
1367	ANKH	ANKH Inorganic Pyrophosphate Transport Regulator	Protein Coding	41	GC05M014706	0.69
1368	GCM2	Glial Cells Missing Transcription Factor 2	Protein Coding	40	GC06M010873	0.69
1369	HSPB1	Heat Shock Protein Family B (Small) Member 1	Protein Coding	50	GC07P076302	0.68
1370	TGFBR3	Transforming Growth Factor Beta Receptor 3	Protein Coding	46	GC01M091680	0.68
1371	BDKRB1	Bradykinin Receptor B1	Protein Coding	42	GC14P096290	0.68
1372	TRB	T Cell Receptor Beta Locus	Protein Coding	18	GC07P147873	0.67
1373	TYRP1	Tyrosinase Related Protein 1	Protein Coding	46	GC09P012683	0.66
1374	PRKACA	Protein Kinase CAMP-Activated Catalytic Subunit Alpha	Protein Coding	50	GC19M014403	0.66
1375	ENPP1	Ectonucleotide Pyrophosphatase/Phosphodiesterase 1	Protein Coding	48	GC06P131808	0.66
1376	SLC4A1	Solute Carrier Family 4 Member 1 (Diego Blood Group)	Protein Coding	48	GC17M044786	0.66
1377	ACP5	Acid Phosphatase 5, Tartrate Resistant	Protein Coding	46	GC19M011574	0.66
1378	ERCC4	ERCC Excision Repair 4, Endonuclease Catalytic Subunit	Protein Coding	45	GC16P013920	0.66
1379	TCIRG1	T Cell Immune Regulator 1, ATPase H+ Transporting V0 Subunit A3	Protein Coding	44	GC11P069343	0.66
1380	CDH23	Cadherin Related 23	Protein Coding	44	GC10P071396	0.66
1381	EBP	EBP Cholestenol Delta-Isomerase	Protein Coding	43	GC0XP048521	0.66
1382	POLR3A	RNA Polymerase III Subunit A	Protein Coding	42	GC10M078393	0.66
1383	GFI1	Growth Factor Independent 1 Transcriptional Repressor	Protein Coding	41	GC01M092474	0.66
1384	COX4I2	Cytochrome C Oxidase Subunit 4I2	Protein Coding	41	GC20P031637	0.66
1385	FAT4	FAT Atypical Cadherin 4	Protein Coding	40	GC04P125315	0.66
1386	SH3PXD2B	SH3 And PX Domains 2B	Protein Coding	40	GC05M172325	0.66
1387	SRSF2	Serine And Arginine Rich Splicing Factor 2	Protein Coding	40	GC17M076734	0.66
1388	SLC11A1	Solute Carrier Family 11 Member 1	Protein Coding	46	GC02P218382	0.65

1389	CHRNA7	Cholinergic Receptor Nicotinic Alpha 7 Subunit	Protein Coding	45	GC15P031923	0.65
1390	EFHD2	EF-Hand Domain Family Member D2	Protein Coding	36	GC01P015409	0.65
1391	GPX1	Glutathione Peroxidase 1	Protein Coding	46	GC03M050895	0.65
1392	MAPK10	Mitogen-Activated Protein Kinase 10	Protein Coding	49	GC04M085990	0.64
1393	MPZ	Myelin Protein Zero	Protein Coding	44	GC01M161304	0.64
1394	PRKRA	Protein Activator Of Interferon Induced Protein Kinase EIF2AK2	Protein Coding	41	GC02M178431	0.64
1395	CYP19A1	Cytochrome P450 Family 19 Subfamily A Member 1	Protein Coding	48	GC15M051208	0.64
1396	TEK	TEK Receptor Tyrosine Kinase	Protein Coding	50	GC09P027109	0.62
1397	AANAT	Aralkylamine N-Acetyltransferase	Protein Coding	39	GC17P076453	0.62
1398	CYP27B1	Cytochrome P450 Family 27 Subfamily B Member 1	Protein Coding	47	GC12M057757	0.62
1399	RGCC	Regulator Of Cell Cycle	Protein Coding	35	GC13P041457	0.61
1400	MIR210	MicroRNA 210	RNA Gene	22	GC11M002651	0.61
1401	ACE2	Angiotensin Converting Enzyme 2	Protein Coding	48	GC0XM015494	0.61
1402	S100B	S100 Calcium Binding Protein B	Protein Coding	45	GC21M050156	0.61
1403	SH3KBP1	SH3 Domain Containing Kinase Binding Protein 1	Protein Coding	40	GC0XM019552	0.6
1404	BCL2L1	BCL2 Like 1	Protein Coding	45	GC20M031664	0.59
1405	ELOVL2	ELOVL Fatty Acid Elongase 2	Protein Coding	40	GC06M010980	0.59
1406	MIR30E	MicroRNA 30e	RNA Gene	23	GC01P040754	0.59
1407	MIR133B	MicroRNA 133b	RNA Gene	22	GC06P052148	0.59
1408	MIR10A	MicroRNA 10a	RNA Gene	22	GC17M048579	0.59
1409	MIR122	MicroRNA 122	RNA Gene	22	GC18P058451	0.59
1410	MIR141	MicroRNA 141	RNA Gene	22	GC12P018574	0.59
1411	MIR200A	MicroRNA 200a	RNA Gene	22	GC01P003359	0.59
1412	MIR100	MicroRNA 100	RNA Gene	22	GC11M122152	0.59
1413	MIR125B1	MicroRNA 125b-1	RNA Gene	22	GC11M122100	0.59
1414	MIR326	MicroRNA 326	RNA Gene	22	GC11M075335	0.59
1415	MIR22	MicroRNA 22	RNA Gene	21	GC17M001713	0.59
1416	MIR146B	MicroRNA 146b	RNA Gene	21	GC10P102436	0.59
1417	MIR17	MicroRNA 17	RNA Gene	21	GC13P091350	0.59
1418	MIR20A	MicroRNA 20a	RNA Gene	20	GC13P091533	0.59
1419	MIR99B	MicroRNA 99b	RNA Gene	20	GC19P051692	0.59
1420	MIR106A	MicroRNA 106a	RNA Gene	20	GC0XM134219	0.59
1421	MIR215	MicroRNA 215	RNA Gene	20	GC01M220117	0.59
1422	RAD51	RAD51 Recombinase	Protein Coding	51	GC15P040694	0.59
1423	CETP	Cholesteryl Ester Transfer Protein	Protein Coding	47	GC16P056961	0.59
1424	IGF2	Insulin Like Growth Factor 2	Protein Coding	48	GC11M002733	0.57
1425	HIF1A	Hypoxia Inducible Factor 1 Subunit Alpha	Protein Coding	46	GC14P061695	0.55
1426	DDT	D-Dopachrome Tautomerase	Protein Coding	38	GC22M023971	0.55
1427	CASP6	Caspase 6	Protein Coding	47	GC04M109688	0.55
1428	NR1H2	Nuclear Receptor Subfamily 1 Group H Member 2	Protein Coding	46	GC19P050329	0.55
1429	HDC	Histidine Decarboxylase	Protein Coding	44	GC15M050241	0.55
1430	BIRC2	Baculoviral IAP Repeat Containing 2	Protein Coding	44	GC11P102347	0.55
1431	RABGEF1	RAB Guanine Nucleotide Exchange Factor 1	Protein Coding	41	GC07P069527	0.54
1432	PLD1	Phospholipase D1	Protein Coding	48	GC03M171600	0.54
1433	MRC1	Mannose Receptor C-Type 1	Protein Coding	40	GC10P017809	0.54
1434	LMNA	Lamin A/C	Protein Coding	48	GC01P156082	0.54
1435	DDX58	DExD/H-Box Helicase 58	Protein Coding	46	GC09M032455	0.54
1436	FMR1	FMRP Translational Regulator 1	Protein Coding	43	GC0XP147933	0.54
1437	ABCB7	ATP Binding Cassette Subfamily B Member 7	Protein Coding	42	GC0XM075053	0.54
1438	ESR2	Estrogen Receptor 2	Protein Coding	48	GC14M064084	0.54
1439	BIRC3	Baculoviral IAP Repeat Containing 3	Protein Coding	45	GC11P102317	0.51
1440	KLHDC1	Kelch Domain Containing 1	Protein Coding	34	GC14P049740	0.51
1441	FCN3	Ficolin 3	Protein Coding	44	GC01M027729	0.5
1442	FCN2	Ficolin 2	Protein Coding	43	GC09P134864	0.5
1443	CD244	CD244 Molecule	Protein Coding	42	GC01M160830	0.5
1444	NEAT1	Nuclear Paraspeckle Assembly Transcript 1	RNA Gene	24	GC11P068984	0.5
1445	STS	Steroid Sulfatase	Protein Coding	46	GC0XP007146	0.49
1446	EPHB2	EPH Receptor B2	Protein Coding	50	GC01P022710	0.48
1447	PROC	Protein C, Inactivator Of Coagulation Factors Va And VIIIa	Protein Coding	49	GC02P127418	0.48
1448	CNTNAP2	Contactin Associated Protein 2	Protein Coding	44	GC07P146116	0.48
1449	DOCK2	Dedicator Of Cytokinesis 2	Protein Coding	44	GC05P169637	0.48
1450	KLB	Klotho Beta	Protein Coding	41	GC04P039408	0.48
1451	ACER1	Alkaline Ceramidase 1	Protein Coding	33	GC19M006306	0.48
1452	KIR2DS2	Killer Cell Immunoglobulin Like Receptor, Two Ig Domains And Short Cytoplasmic Tail 2	Protein Coding	25	GC19MR00122	0.48
1453	IGHV1OR21-1	Immunoglobulin Heavy Variable 1/OR21-1 (Non-Functional)	Pseudogene	13	GC21M010649	0.48
1454	RNASE2CP	Ribonuclease A Family Member 2C, Pseudogene	Pseudogene	9	GC14P031379	0.48
1455	SOD1	Superoxide Dismutase 1	Protein Coding	50	GC21P031659	0.47
1456	XIAP	X-Linked Inhibitor Of Apoptosis	Protein Coding	47	GC0XP123859	0.46
1457	HSP90AA1	Heat Shock Protein 90 Alpha Family Class A Member 1	Protein Coding	47	GC14M102080	0.46
1458	NOTCH1	Notch Receptor 1	Protein Coding	49	GC09M137332	0.46
1459	PRKCH	Protein Kinase C Eta	Protein Coding	49	GC14P061187	0.46
1460	STARD7	StAR Related Lipid Transfer Domain Containing 7	Protein Coding	37	GC02M096184	0.46
1461	CPQ	Carboxypeptidase Q	Protein Coding	36	GC08P096645	0.46
1462	CXCL17	C-X-C Motif Chemokine Ligand 17	Protein Coding	32	GC19M042428	0.46
1463	SPN	Sialophorin	Protein Coding	39	GC16P029662	0.45
1464	NOX5	NADPH Oxidase 5	Protein Coding	37	GC15P092249	0.45
1465	FCGR2C	Fc Gamma Receptor IIc (Gene/Pseudogene)	Protein Coding	35	GC01P161647	0.45
1466	SPINK6	Serine Peptidase Inhibitor Kazal Type 6	Protein Coding	32	GC05P148202	0.45
1467	EGF	Epidermal Growth Factor	Protein Coding	50	GC04P109912	0.41
1468	MAPK8	Mitogen-Activated Protein Kinase 8	Protein Coding	48	GC10P048306	0.39
1469	VNN1	Vanin 1	Protein Coding	44	GC06M132680	0.39
1470	LCP1	Lymphocyte Cytosolic Protein 1	Protein Coding	42	GC13M046132	0.39
1471	RBBP7	RB Binding Protein 7, Chromatin Remodeling Factor	Protein Coding	41	GC0XM016839	0.39
1472	PLA2G2E	Phospholipase A2 Group IIE	Protein Coding	38	GC01M019920	0.39
1473	PLA2G2F	Phospholipase A2 Group IIF	Protein Coding	34	GC01P020139	0.39
1474	PADI3	Peptidyl Arginine Deiminase 3	Protein Coding	41	GC01P017249	0.39
1475	SLURP1	Secreted LY6/PLAUR Domain Containing 1	Protein Coding	40	GC08M142740	0.37

1476	IL3RA	Interleukin 3 Receptor Subunit Alpha	Protein Coding	42	GC0XP001336	0.37
1477	CSN2	Casein Beta	Protein Coding	34	GC04M069955	0.37
1478	GAPDH	Glyceraldehyde-3-Phosphate Dehydrogenase	Protein Coding	48	GC12P018545	0.36
1479	NR1H3	Nuclear Receptor Subfamily 1 Group H Member 3	Protein Coding	46	GC11P047248	0.36
1480	LGALS1	Galectin 1	Protein Coding	42	GC22P037675	0.34
1481	LY96	Lymphocyte Antigen 96	Protein Coding	42	GC08P073991	0.34
1482	SERPINB13	Serpin Family B Member 13	Protein Coding	36	GC18P063586	0.34
1483	GLI3	GLI Family Zinc Finger 3	Protein Coding	48	GC07M041960	0.32
1484	TTN	Titin	Protein Coding	46	GC02M178525	0.32
1485	COL6A3	Collagen Type VI Alpha 3 Chain	Protein Coding	45	GC02M237324	0.32
1486	ABL2	ABL Proto-Oncogene 2, Non-Receptor Tyrosine Kinase	Protein Coding	44	GC01M179126	0.32
1487	EPS15	Epidermal Growth Factor Receptor Pathway Substrate 15	Protein Coding	44	GC01M051354	0.32
1488	CEACAM1	CEA Cell Adhesion Molecule 1	Protein Coding	43	GC19M042507	0.32
1489	ENTPD6	Ectonucleoside Triphosphate Diphosphohydrolase 6	Protein Coding	42	GC20P025196	0.32
1490	DNAH5	Dynein Axonemal Heavy Chain 5	Protein Coding	40	GC05M013693	0.32
1491	GRHL2	Grainyhead Like Transcription Factor 2	Protein Coding	40	GC08P101492	0.32
1492	COL6A4P1	Collagen Type VI Alpha 4 Pseudogene 1	Pseudogene	13	GC03M015151	0.32
1493	KLK11	Kallikrein Related Peptidase 11	Protein Coding	41	GC19M062307	0.31
1494	GGT1	Gamma-Glutamyltransferase 1	Protein Coding	49	GC22P024583	0.31
1495	ANPEP	Alanyl Aminopeptidase, Membrane	Protein Coding	49	GC15M089784	0.31
1496	SPTLC2	Serine Palmitoyltransferase Long Chain Base Subunit 2	Protein Coding	47	GC14M077505	0.31
1497	SPTLC1	Serine Palmitoyltransferase Long Chain Base Subunit 1	Protein Coding	45	GC09M092007	0.31
1498	CDH3	Cadherin 3	Protein Coding	47	GC16P068637	0.27
1499	CNR1	Cannabinoid Receptor 1	Protein Coding	46	GC06M088139	0.27
1500	MAG	Myelin Associated Glycoprotein	Protein Coding	46	GC19P035292	0.27
1501	HMGCR	3-Hydroxy-3-Methylglutaryl-CoA Reductase	Protein Coding	45	GC05P075336	0.27
1502	PDE7A	Phosphodiesterase 7A	Protein Coding	44	GC08M065720	0.27
1503	ADPRH	ADP-Ribosylarginine Hydrolase	Protein Coding	35	GC03P119579	0.27
1504	RXRA	Retinoid X Receptor Alpha	Protein Coding	48	GC09P134317	0.27
1505	LAG3	Lymphocyte Activating 3	Protein Coding	38	GC12P018557	0.27
1506	MANF	Mesencephalic Astrocyte Derived Neurotrophic Factor	Protein Coding	38	GC03P051385	0.27
1507	IL27	Interleukin 27	Protein Coding	37	GC16M028645	0.27
1508	SETDB2-PHF11	SETDB2-PHF11 Readthrough	Protein Coding	8	GC13P049834	0.27
1509	FZD5	Frizzled Class Receptor 5	Protein Coding	47	GC02M207762	0.23
1510	FMO3	Flavin Containing Dimethylaniline Monoxygenase 3	Protein Coding	46	GC01P171090	0.23
1511	BACE1	Beta-Secretase 1	Protein Coding	46	GC11M117285	0.23
1512	GLO1	Glyoxalase I	Protein Coding	45	GC06M061455	0.23
1513	PPP3CB	Protein Phosphatase 3 Catalytic Subunit Beta	Protein Coding	45	GC10M073436	0.23
1514	FZD9	Frizzled Class Receptor 9	Protein Coding	44	GC07P073433	0.23
1515	FZD10	Frizzled Class Receptor 10	Protein Coding	44	GC12P130162	0.23
1516	FAP	Fibroblast Activation Protein Alpha	Protein Coding	44	GC02M162170	0.23
1517	PEBP1	Phosphatidylethanolamine Binding Protein 1	Protein Coding	44	GC12P118135	0.23
1518	S1PR2	Sphingosine-1-Phosphate Receptor 2	Protein Coding	44	GC19M010223	0.23
1519	BID	BH3 Interacting Domain Death Agonist	Protein Coding	44	GC22M017734	0.23
1520	ABHD5	Abhydrolase Domain Containing 5, Lysophosphatidic Acid Acyltransferase	Protein Coding	44	GC03P043707	0.23
1521	PPM1A	Protein Phosphatase, Mg2+/Mn2+ Dependent 1A	Protein Coding	44	GC14P060245	0.23
1522	CRABP2	Cellular Retinoic Acid Binding Protein 2	Protein Coding	43	GC01M156701	0.23
1523	CANX	Calnexin	Protein Coding	43	GC05P179678	0.23
1524	PLSCR1	Phospholipid Scramblase 1	Protein Coding	43	GC03M146515	0.23
1525	UGCG	UDP-Glucose Ceramide Glucosyltransferase	Protein Coding	42	GC09P111896	0.23
1526	DPP9	Dipeptidyl Peptidase 9	Protein Coding	42	GC19M004675	0.23
1527	CHSY1	Chondroitin Sulfate Synthase 1	Protein Coding	42	GC15M101175	0.23
1528	PTGER1	Prostaglandin E Receptor 1	Protein Coding	41	GC19M014444	0.23
1529	UBR5	Ubiquitin Protein Ligase E3 Component N-Recognin 5	Protein Coding	41	GC08M102252	0.23
1530	TOP3A	DNA Topoisomerase III Alpha	Protein Coding	41	GC17M024647	0.23
1531	ETS2	ETS Proto-Oncogene 2, Transcription Factor	Protein Coding	41	GC21P038805	0.23
1532	CD164	CD164 Molecule	Protein Coding	41	GC06M109366	0.23
1533	NPTN	Neuroplastin	Protein Coding	40	GC15M073560	0.23
1534	SEMA6A	Semaphorin 6A	Protein Coding	40	GC05M116443	0.23
1535	SERPINB1	Serpin Family B Member 1	Protein Coding	40	GC06M002833	0.23
1536	CD300A	CD300a Molecule	Protein Coding	40	GC17P074466	0.23
1537	DDX17	DEAD-Box Helicase 17	Protein Coding	40	GC22M038483	0.23
1538	TOX	Thymocyte Selection Associated High Mobility Group Box	Protein Coding	39	GC08M058791	0.23
1539	CD200R1	CD200 Receptor 1	Protein Coding	39	GC03M112921	0.23
1540	GOLPH3	Golgi Phosphoprotein 3	Protein Coding	38	GC05M032124	0.23
1541	SYTL4	Synaptotagmin Like 4	Protein Coding	38	GC0XM100674	0.23
1542	DNAH8	Dynein Axonemal Heavy Chain 8	Protein Coding	38	GC06P077030	0.23
1543	SENP5	SUMO Specific Peptidase 5	Protein Coding	37	GC03P196869	0.23
1544	CDX1	Caudal Type Homeobox 1	Protein Coding	37	GC05P150166	0.23
1545	CD300LB	CD300 Molecule Like Family Member B	Protein Coding	37	GC17M074520	0.23
1546	NOL8	Nucleolar Protein 8	Protein Coding	36	GC09M092297	0.23
1547	DHRS7	Dehydrogenase/Reductase 7	Protein Coding	36	GC14M060144	0.23
1548	UCN3	Urocortin 3	Protein Coding	36	GC10P005396	0.23
1549	RNF39	Ring Finger Protein 39	Protein Coding	35	GC06M061207	0.23
1550	CD300E	CD300e Molecule	Protein Coding	35	GC17M074609	0.23
1551	ELOF1	Elongation Factor 1	Protein Coding	34	GC19M011551	0.23
1552	PDZK1IP1	PDZK1 Interacting Protein 1	Protein Coding	34	GC01M047183	0.23
1553	CD300C	CD300c Molecule	Protein Coding	34	GC17M074544	0.23
1554	CROCC	Ciliary Rootlet Coiled-Coil, Rootletin	Protein Coding	34	GC01P017623	0.23
1555	MAP6	Microtubule Associated Protein 6	Protein Coding	34	GC11M075586	0.23
1556	TMEM108	Transmembrane Protein 108	Protein Coding	34	GC03P133038	0.23
1557	WFDC12	WAP Four-Disulfide Core Domain 12	Protein Coding	33	GC20M045123	0.23
1558	CD300LD	CD300 Molecule Like Family Member D	Protein Coding	32	GC17M074579	0.23
1559	NWD1	NACHT And WD Repeat Domain Containing 1	Protein Coding	30	GC19P016719	0.23
1560	MIR29A	MicroRNA 29a	RNA Gene	22	GC07M130876	0.23
1561	MIR375	MicroRNA 375	RNA Gene	21	GC02M219001	0.23
1562	MIR19A	MicroRNA 19a	RNA Gene	20	GC13P091532	0.23

1563	MIR323A	MicroRNA 323a	RNA Gene	18	GC14P109066	0.23
1564	RPL21P119	Ribosomal Protein L21 Pseudogene 119	Pseudogene	10	GC16M009255	0.23
1565	RPS14P8	Ribosomal Protein S14 Pseudogene 8	Pseudogene	9	GC05P116562	0.23
1566	RPL17P2	Ribosomal Protein L17 Pseudogene 2	Pseudogene	8	GC14M060212	0.23
1567	ALRH	Allergic Rhinitis	Genetic Locus	2	GC00U922352	0.23
